# Floristic inventory and distribution characteristics of vascular plants in forest wetlands of South Korea

**DOI:** 10.3897/BDJ.10.e85848

**Published:** 2022-09-15

**Authors:** Jong-Won Lee, Ho-Geun Yun, Tae Young Hwang, Jong-Bin An

**Affiliations:** 1 Korea National Arboretum, DMZ Forest Biological Conservation, Yanggu-gun, Republic of Korea Korea National Arboretum, DMZ Forest Biological Conservation Yanggu-gun Republic of Korea

**Keywords:** orest wetland, Korea endemic plants, rare plants, floristic target plants, invasive alien plants, wetland preference

## Abstract

This study surveyed the vascular flora present in 455 forest wetlands in South Korea to provide baseline data for conservation, management and monitoring and to support preparation for climate change. The survey period was from April 2015 to November 2019. The vascular flora of 455 forest wetlands were identified and were found to consist of 143 families, 582 genera, 1,246 species, 26 subsp., 104 var., 12 f. and 1,388 individual taxa. Eight taxa were identified as Grade II endangered wild plants, 220 taxa were identified as northern lineage plants of the Korean Peninsula, 66 taxa were endemic to the Korean Peninsula and 94 taxa were rare plants as designated by the Korea Forest Service. Amongst them, eight taxa were Critically Endangered species, 10 taxa were Endangered species, 31 taxa were Vulnerable species, 31 taxa were Least Concern species and 14 taxa were Data Deficient species. Further, 411 taxa were floristic target plants, of which 17 taxa were classified as grade V, 70 taxa as grade IV, 110 taxa as grade III, 90 taxa as grade II and 29 taxa as grade I. There were 64 invasive alien plant taxa. Eighteen of these species were classed as Widespread species, nine taxa were Serious Spread species, 13 taxa were Spread Risk species, 18 taxa were Spread Concern species and six taxa were Continuing Spread species. According to wetland preference, 152 taxa (11.0%) were obligate wetland plants, 138 taxa (9.9%) were facultative wetland plants and 177 (12.77%) taxa were facultative plants. Additionally, 198 taxa (14.3%) were facultative upland plants and 723 taxa (52.1%) were obligate upland plants.

## Introduction

A wetland can include swamps, marshes, peatlands, or any area with water, including areas where the depth of water does not exceed 6 m at low tide, whether natural or artificial and permanent or temporary and whether their waters are purified, running, fresh, brackish or salty (Ramsar 2021). A wetland is also a depression (Wells and Mundkur 1996) or lowland (Federal Geographic Data Committee 2013) that has been covered with water at various intervals of time. Wetlands are flooded or saturated areas with a frequency and duration that can be sustained by the ground or groundwater (Griffin et al. 2016). Wetlands are lands transitional between terrestrial and aqualic systems where the water table is usually at or near the surface or the land is covered by shallow water (Dahl and Johnson 1991).

The Wetlands Conservation Act (Korea Ministry of Environment 2021b) defines inland and coastal wetlands as areas where freshwater, brackish water or saltwater covers the surface permanently or temporarily. According to the topography, inland wetlands are classified as river, lake or hill types. Hill-type wetlands are divided into those that are high moor, low moor, lowland wetland and marsh, according to their vegetation, soil and hydrological regime. These wetlands have the following functions: a hydrological function enabling land moisture regulation through naturally-formed drainage and irrigation; a biodiversity maintenance function by providing habitats for various organisms; a carbon storage function, for example, peatlands absorb carbon through the remains of emerged plants and aquatic plants around wetlands while accounting for only 3% of the world's land area, but containing approximately 30% of all carbon on land; trapping and fixing sediment with the roots of the plants that make up the wetlands; and moderating damage from disasters such as flooding and storms (Ramsar Convention Secretariat 2013).

However, wetlands are widely recognised as one of the most vulnerable ecosystems to climate change and especially the increased severity of drought (Zedler and Kercher 2005, Intergovernmental Panel on Climate Change 2007, Johnson et al. 2010). This is especially true for wetlands in mountainous and alpine areas, where climate change, causing effects such as reduced snow cover, is accelerating (Burkett and Kusler 2000, Carpenter et al. 2007, Erwin 2008). However, despite their importance and sensitivity to climate change, the vulnerability of montane wetlands has not yet been quantified, mainly owing to a lack of research. Montane wetlands are refugia and hotspots of biodiversity (Chatterjee et al. 2010, Son et al. 2014) because they have a relatively low species diversity, but provide unique habitats for specific plant species (Omar et al. 2016).

Korea Forest Service has been conducting research on forest wetlands since 2005. According to the Korea Forest Service, forest wetlands are defined as all wetlands (such as swamps, marshes and peatlands) that are found in areas classified as forests, as well as swamps that are home to woody plants, such as tall trees, shrubs and bushes, even in those areas not classified as forests (Korea Forest Service 2006). Over an eight-year period from 2006 to 2014, the first national survey of forest wetlands covered 6.37 million hectares of forests within national and private forest areas, which represented one third of the nation's land. Consequently, 1,264 sites (390 ha) of forest wetlands and 1,260 plant taxa (135 families, 545 genera, 1,101 species, seven subspecies, 136 varieties and 16 f. were identified (Korea National Arboretum 2016). This represents 27.15% of the total abundance of vascular flora on the entire Korean Peninsula (Korea National Arboretum 2020b). The large number of taxa identified relative to the area confirms that forest wetlands are an important site of biodiversity and must be preserved.

A total of 1,280 forest wetlands were precisely surveyed for five years between 2015 and 2019, which included 1,264 sites derived from the first survey and 16 new target sites. A second survey of forest wetlands was conducted to grade the sites following evaluation. Consequently, 455 sites functioning as forest wetlands were identified and classified by grade. The 455 sites were categorised as either A, B, C or D grades with 30, 201, 184 and 40 sites belonging to each grade, respectively. Twenty three sites were reclassified as modified wetlands due to desiccation and a complete clearing of the sites. Consequently, forest genetic resource reserves (FGRRs) and other effective area-based conservation measures (OECMs) should be established as soon as possible to achieve a more systematic management strategy for forest wetlands, which are areas rich in biodiversity.

Additionally, the mountainous terrain accounts for approximately 63% of South Korea's land and is home to a wide range of forest species. The forest wetlands (montane wetlands) also serve as sanctuaries for flora and fauna, buffer zones against climate change and provide excellent carbon storage, amongst other functions. Nevertheless, the importance of forest wetlands has been overlooked. On the Korean Peninsula, forest wetlands are a hotspot for biodiversity conservation and they represent ecosystems that must be protected and managed, but to effectively do this, it is crucial to have a better understanding of their current state. The purpose of the present study was to investigate the vascular flora that must be surveyed to conserve and utilise forest wetlands in the long term, as well as to use this information as the basis for the restoration of forest wetlands in preparation for future climate changes.

## Method

### Target area

**Figure 1.**
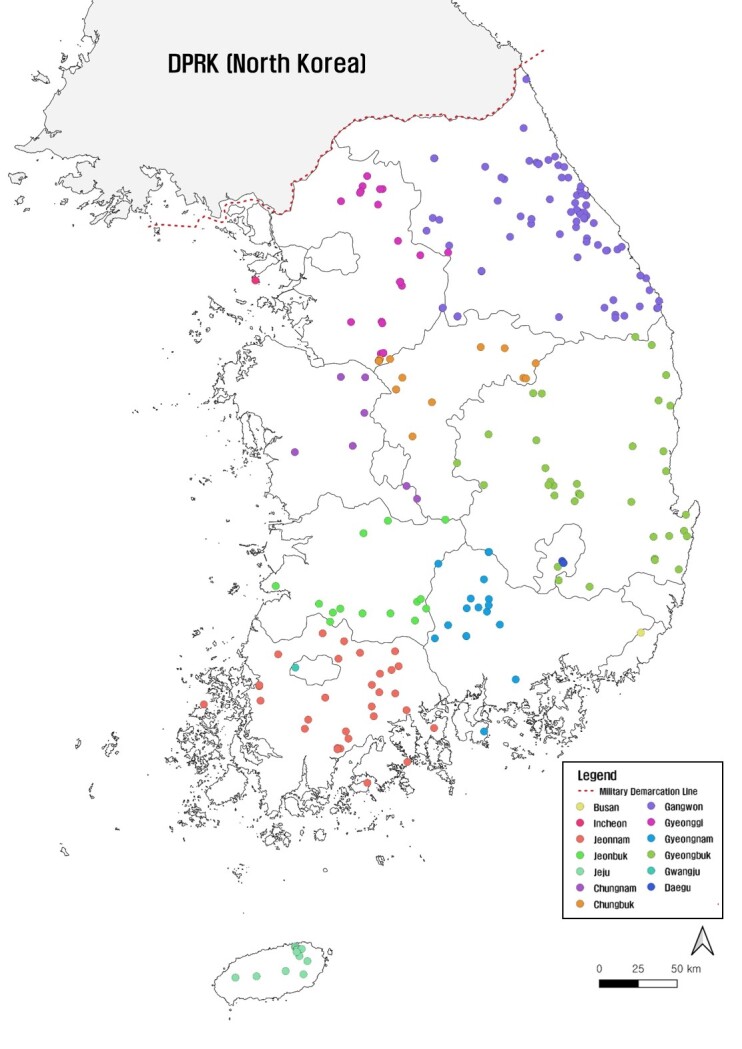
Map showing the location of forest wetlands by region.

As part of the study, 455 forest wetlands of grades A-D, which were identified in a second survey in 2015-2019, were selected from 1,264 national forest wetlands first surveyed from 2006 to 2014 and 16 newly-discovered forest wetlands (Fig. 1, Suppl. material 1). The 455 forest wetlands were classified into grades A, B, C and D, with 30, 201, 184 and 40 sites accounting for each grade, respectively. The 455 forest wetlands are distributed across 14 administrative districts, excluding Seoul Metropolitan City, Daejeon Metropolitan City and Sejong Metropolitan Autonomous City. The region with the greatest number of forest wetlands is Gangwon-do with 132 sites (29.0%) covering an area of 875,322 m^2^ (31.7%), followed by Gyeongsangbuk-do with 68 sites (14.9%) covering an area of 711,792 m^2^ (25.8%) and Jeollanam-do with 56 sites (12.3%) covering an area of 216,910 m^2^ (7.9%). Additionally, 132 sites (29%) were state-owned forests, while 323 sites (71%) were privately-owned forests. Altitudes range from 20 m to 1,560 m above sea level. However, 342 forest wetlands (approximately 75%), were found to be distributed at elevations below 500 m, 104 (approximately 23%) at elevations between 501 m and 1,000 m and nine (approximately 2%) at elevations above 1,001 m (Korea National Arboretum 2019b).

Forest wetlands are classified into four categories: natural forest wetlands, abandoned paddy-field forest wetlands, man-made forest wetlands and modified wetlands (Korea National Arboretum 2020a). The 455 forest wetlands include 193 natural forest wetlands, 237 abandoned paddy-field forest wetlands, two man-made forest wetlands and 23 modified forest wetlands.

### Research method

Field notes were used for the survey of flora. The field note includes survey number, survey date, investigators, survey site, altitude, survey route, GPS coordinates, according to the order of plant numbers, plant name, image data and specimen collection items. A field survey of vascular flora was conducted between April 2015 and November 2019. Plant species were identified in the field as much as possible to prevent disturbance to the plants and, in the case of plants that were difficult to identify in the field, only individuals with reproductive organs were collected for laboratory identification. Identifying plants was based on literature from Lee (1996), Lee (2006), Lee (2014a) and Lee (2014b). Taxonomy of the occurring plants was based on Engler's taxonomy (Melchior 1964) and the list was arranged accordingly (Suppl. material 18). These scientific names and Korean names were derived from the Korea National Plant List (Korea National Arboretum 2020b). The plant list was arranged by family and the classifications of the genera and below taxa were arranged alphabetically. Listing northern lineage plants followed Gantsetseg et al. (2020), North Korean plants followed Korea National Arboretum (2019c), endemic plants followed Chung et al. (2017) and Korea National Arboretum (2020b), rare plants and endangered wild species followed Korea National Arboretum (2009) and Korea Ministry of Environment (2021a), respectively, floristic target plants followed Kim (2000) and National Institute of Ecology (2018), plants in the limestone zone followed Korea National Arboretum (2010) and invasive alien species followed Chung et al. (2017) and Korea National Arboretum (2019a). The classification of wetland plants by vascular plant type followed Choung et al. (2012), Choung et al. (2020), Choung et al. (2021).

## Results

### Distribution characteristics of the total flora of forest wetlands

#### Total vascular flora

The vascular plants identified in 455 forest wetlands in South Korea belonged to 143 families, 582 genera, 1,246 species, 26 subsp., 104 var., 12 f., and 1,388 taxa (Suppl. material 2). Ferns accounted for 72 taxa, belonging to 19 families, 32 genera, 69 species and three var., gymnosperms for five families, eight genera and 14 species, angiosperms for 925 taxa, belonging to 101 families, 398 genera, 819 species, 23 subsp., 75 var. and 8 f. and monocotyledons for 377 taxa, belonging to 18 families, 145 genera, 344 species, three subsp., 26 var. and 4 f. This accounted for about 29.91% of the 4,641 taxa of vascular plants in South Korea (Korea National Arboretum 2020b). The plant families with the highest number of species recorded were Asteraceae (137 taxa, 9.9% of species recorded), Poaceae (117 taxa, 8.4%), Cyperaceae (113 taxa, 8.1% of species recorded), Rosaceae (63 taxa, 4.5% of species recorded) and Liliaceae (61 taxa, 4.4% of species recorded).

The most frequently occurring species were (in descending order): *Persicariathunbergii* at 315 sites (69.2%), *Salixpierotii* at 311 sites (68.4%), *Oplismenusundulatifolius* at 269 sites (59.1%), *Artemisiaindica* at 249 sites (54.7%), *Impatienstextorii* at 235 sites (51.7%) and *Clematisapiifolia* at 235 sites (50.6%). However, amongst the 1,388 taxa, 891 taxa including *Syringareticulata* were observed at only ten or fewer sites and 295 taxa including Viciaangustifoliavar.minor were recorded at only one site.

#### Northern lineage plants and North Korean plants of the Korean Peninsula

**Figure 2.**
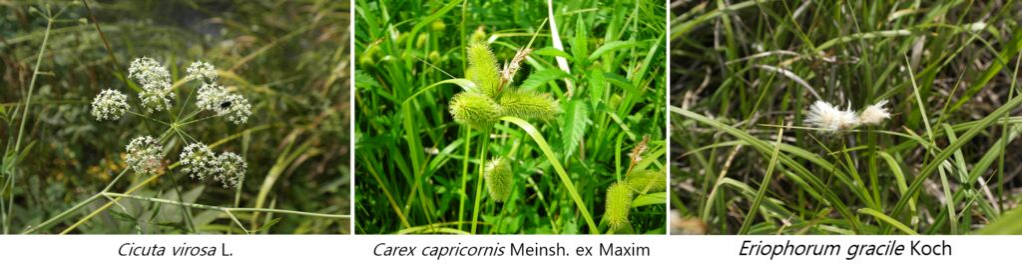
The images show northern lineage plants in forest wetlands of Korea.

In total, 220 taxa of plants from the northern Korean Peninsula were identified and these taxa belonged to 65 families, 141 genera, 208 species and four subspecies (Suppl. material 18). This number represents approximately 35.7% of the 616 taxa of northern lineage plants that occur on the Korean Peninsula, which is 15.9% of the total vascular plant taxa (1,388) observed in the entire forest wetlands. These data can be used as a basis for future research on ecosystem changes related to climate change. The remarkable plants identified as northern lineage plants included *Eleutheroccussenticosus*, *Trigonotisradicans*, *Cicutavirosa*, *Loniceracaerulea*, *Carexcapricornis*, *Scorzoneraalbicaulis* and *Eriophorumgracile* (Fig. 2).

Northern lineage plants are those that migrated to the Korean Peninsula from the north during the Last Inter-Glacial (LIG) of the Last Glacial Maximum (LGM). They are widespread in northern East Asia, including China and Russia, but very limited or remnant in the Korean Peninsula. However, if the growing environment becomes poor as a result of global warming or other factors, there will be severe challenges to their growth and a great danger of extinction. Therefore, it is imperative to develop conservation measures both within and outside the local area, as well as active management measures to prevent anthropogenic threats.

North Korean plants are native only to the North Korean region of the Korean Peninsula. A total of 753 taxa of species distributed exclusively in North Korea were selected from the 3,182 taxa included in the North Korean Vascular Plant Checklist (Korea National Arboretum 2019c). Korea National Plant List (Korea National Arboretum 2020b) and others were consulted for the screening of the data selected above, resulting in a North Korean plant list of 497 taxa including *Lycopodiumalpinum* and *Equisetumfluviatile*. As with northern plants, the habitats of North Korean plants will be damaged by climate change, which will destroy forest wetland ecosystems as well.

Fourteen North Korean plants were observed in forest wetlands in South Korea, including *Calamagrostispseudophragmites*, *Equisetumsylvaticum*, *Ranunculuscrucilobus*, *Prunusjaponica*, *Ligulariaintermedia*, *Agrostiscanina*, *Stachysoblongifolia*, *Carexidzuroei*, *Juncusfiliformis*, *Carexschmidtii*, *Platantherasachalinensis*, *Elymusgmelinii*, *Lonicerasubhispida* and *Artemisiasacrorum*. It has been determined that additional detailed research on North Korean plants is required as part of conservation measures, amongst other considerations.

#### Endemic plants of the Korean Peninsula

**Figure 3.**
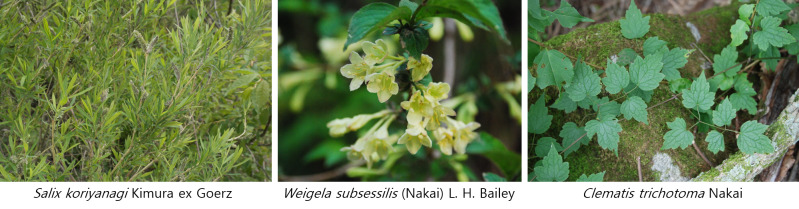
Selected Korean endemic plants in forest wetlands of Korea.

A total of 66 taxa of endemic plants belonging to 27 families, 57 genera, 59 species, six varieties, and one f. were identified in 455 forest wetlands (Suppl. material 3). This accounts for 16.6% of the 398 taxa of endemic plants of the Korean Peninsula (Chung et al. 2017, Korea National Arboretum 2020b). In the 455 forest wetlands, the most frequently occurring endemic plants of the Korean Peninsula were *Weigelasubsessilis* and *Salixkoriyanagi* (*Fig. 3*), which were found in 111 sites, followed by *Clematistrichotoma* occurring in 29 sites, Rhododendronyedoensef.poukhanense in 26 sites, Populus×tomentiglandulosa in 23 sites and *Hemerocallishakuunensis*, *Asterkoraiensis* and *Carexerythrobasis* in 16 sites.

#### Rare plants on the IUCN Red List

**Figure 4.**
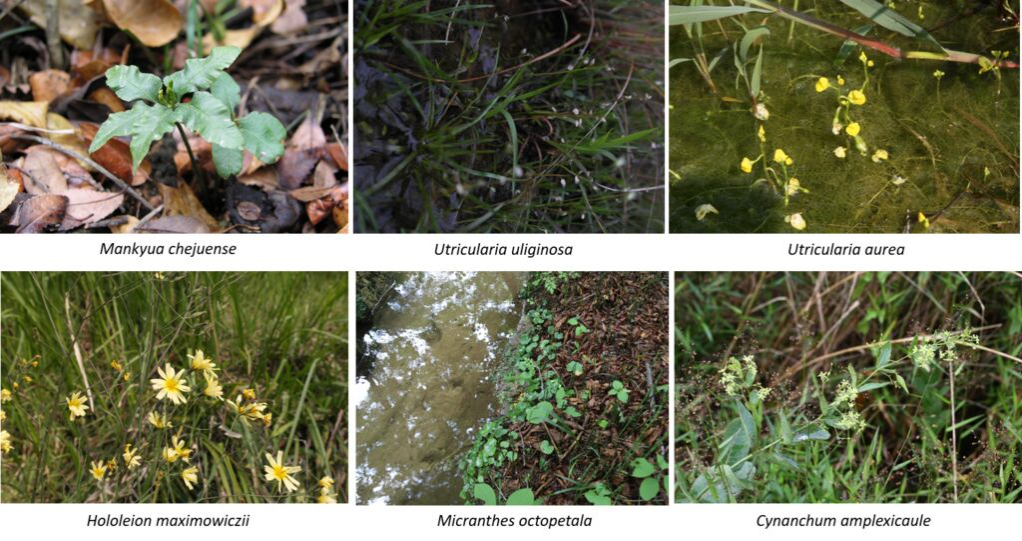
A selection of some rare plants by IUCN identified during the survey.

Ninety-four taxa were identified as rare plants and they belonged to 46 families, 81 genera, 86 species, one subspecies, five varieties and two f. (Suppl. material 4). This is approximately 16.5% of the 571 taxa on the rare plant list of South Korea (Korea National Arboretum 2009), which was re-evaluated by the Korea National Arboretum according to the IUCN Criteria. Eight Critically Endangered (CR) taxa, ten Endangered (EN) taxa, 31 Vulnerable (VU) taxa, 31 Least Concern (LC) taxa and 14 Data Deficient (DD) taxa were identified (Suppl. material 4, Fig. 4).

Irisensatavar.spontanea, an LC species, was found most frequently of all the rare plants, with 67 occurrences, followed by the LC species *Aristolochiacontorta* with 23 occurrences, Gentianatrifloravar.japonica with 18 occurrences, *Utriculariacaerulea* with 16 occurrences and the VU species *Droserarotundifolia* with 14 occurrences. The frequency of occurrence of rare plants by grade amongst the CR species was seven for *Mankyuachejuense*, six for *Utriculariauliginosa* and five for *Cicutavirosa*. Amongst the EN species, *Hololeionmaximowiczii* occurred six times and *Micranthesoctopetala* and *Cynanchumamplexicaule* occurred five times each. Amongst the VU species, *Droserarotundifolia* was recorded 14 times, *Utriculariabifida* 13 times and *Pogoniajaponica* 11 times. Amongst the LC species, Irisensatavar.spontanea occurred 67 times, *Aristolochiacontorta* 23 times and Gentianatrifloravar.japonica 18 times. Amongst the DD species, *Sagittariatrifolia* occurred 13 times, *Scrophulariakoraiensis* 10 times and Eleutherococcusdivaricatusvar.chiisanensis eight times.

#### Endangered wild plants

The term endangered wild organisms refer to species in danger of extinction in the near future, with a very small number of individuals remaining whose population has been severely reduced by natural or human factors (Korea Ministry of Environment 2021a). Eight taxa were identified as endangered wild plants of grade II, including *Menyanthestrifoliata* (Suppl. material 5). Frequency of occurrence of these species can be found in Suppl. material 5. The record of *Eleutherococcussenticosus* from Goseong-gun, Gyeongsangnam-do needs to be re-examined. As this species is also known to mainly grow in northern regions (Lee 2014a).

#### Floristic target plants

**Figure 5.**
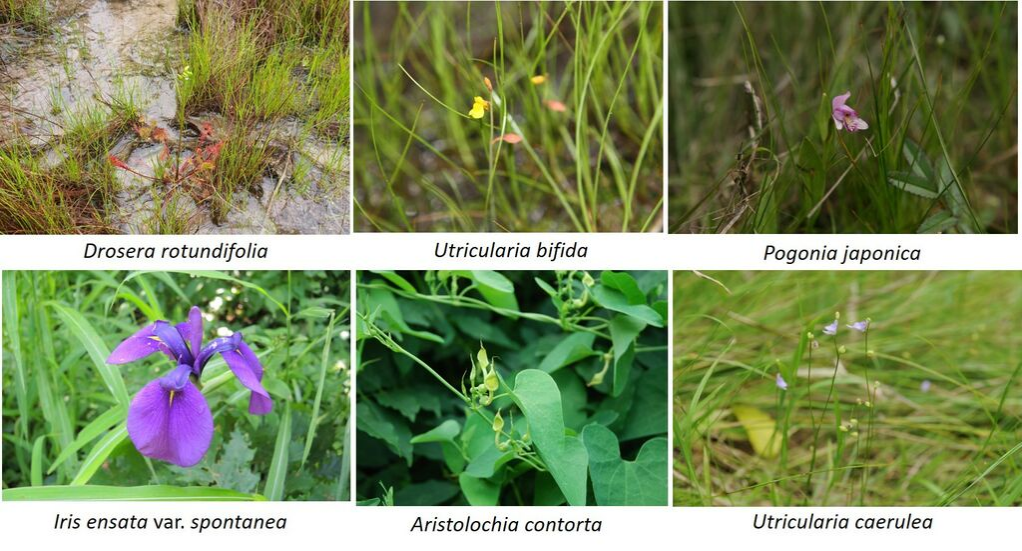
The picitures show floristic target plants in forest wetlands of Korea.

A total of 411 taxa were identified as floristic target plants. These belonged to 103 families, 268 genera, 381 species, five subspecies, 24 varieties and one f. (Suppl. material 18, Fig. 5), representing about 29.6% of the 1,388 taxa of emergent plants and 27.8% of the 1,476 taxa of floristic target plants.

Grade V plants have discontinuous distributions, such as northern or southern plants that were introduced during glacial periods or are isolated in a limited geographical area. A total of 17 taxa including *Nephrolepiscordifolia*, Trientaliseuropaeavar.arctica and *Platantherahologlottis* were identified, accounting for 6.6% of the 258 taxa belonging to grade V (Suppl. material 6). Amongst the grade V plants, the most frequently occurring taxa were *Actaeaheracleifolia* (11 recods), followed by *Braseniaschreberi* eight records), *Mankyuachejuense* (seven records) and *Asperulalasiantha* and *Utriculariauliginosa* with six records each.

Grade IV plants are distributed in one of the four floristic subregions, the Middle, Jeju, Southern Coast and Southern Subregions and to this group belong taxa that grow sporadically and have few individuals or grow in groups and have a relatively large number of individuals. A total of 70 taxa of grade IV floristic regional indicator plants were identified, including *Rhododendronmicranthum*, *Hanabusayaasiatica* and *Trilliumcamschatcense*. Approximately 15.9% of the total 440 taxa of grade IV plants were observed in the forest wetlands. The most frequently occurring grade IV plants were *Toxicodendronsuccedaneum* (22 records), *Hydrocotylesibthorpioides* (18 records), *Utriculariacaerulea* (16 records) and *Utriculariabifida* (13 records) (Suppl. material 7).

Grade III plants occurred in two of the four subregions and include taxa that grow infrequently and have small populations, as well as taxa that grow in groups and have relatively large populations. A total of 110 grade III taxa were identified, including Lysimachiavulgarisvar.davurica, *Cynanchumamplexicaule* and *Scutellariainsignis*. Approximately 29.6% of the 371 grade III floristic target plant taxa were present. The most frequently occurring grade III taxa were Lysimachiavulgarisvar.davurica (59 records), *Prunussargentii* (51 records), *Betuladavurica* (29 records), *Vitiscoignetiae* (28 records), *Spiraeafritschiana* (22 records) and *Cynanchumnipponicum* (21 records) (Suppl. material 8).

Plants of grade II are either unique to specific environments or are distributed relatively nationwide. However, they include taxa that are generally associated with large populations of 1,000 individuals or more. A total of 90 grade II taxa were identified, including *Bolboschoenusmaritimus*, *Maianthemumbifolium* and *Lobeliasessilifolia*. Approximately 43.8% of the 207 grade II taxa were observed in the area. *Alnusjaponica* and *Glycerialeptolepis* were recorded 84 times each, *Scutellariadependens* 68 times, Irisensatavar.spontanea 67 times and *Tripterygiumregelii* 53 times.

Taxa in grade I occur in three of the four subregions and become established sporadically and have relatively small populations or become established in groups and have relatively large populations. For grade I plants, 129 taxa were observed, including *Lycopusmaackianus*, *Tricyrtismacropod*a and *Arisaemaheterophyllum*. Of the 200 grade I taxa, 64.5% were recorded. The most frequently recorded were *Carexdispalata* (101 records), *Onocleainterrupta* (100 records), *Linderaerythrocarpa* (88 records), *Salixchaenomeloides* (69 records) and *Eleutherococcussessiliflorus* (68 records).

#### Limestone area plants

Limestone area plants include northern alpine flora occurring in lowland areas, such as riversides, new species emerging in isolated areas and isolated distributions of coastal plants (Korea National Arboretum 2010). A total of 31 limestone area taxa were identified, including *Spiraeatrichocarpa* and *Stemmacanthauniflora* (Suppl. material 9). The most frequently occurring plants in the limestone area were *Smilaxsieboldii* (124 records), followed by *Quercusvariabilis* (51 records), *Juniperusrigida* (22 records) and *Euonymusalatus* (16 records). However, *Smilaxsieboldii* and *Quercusvariabilis* are distributed nationwide and additional research on plants in limestone areas is needed.

#### Invasive alien plants

A total of 64 invasive alien plant taxa were identified, belonging to 17 families, 54 genera, 63 species and one subspecies (Suppl. material 10). This represented approximately 17.1% of the 375 taxa in the invasive alien plant inventory (Korea National Arboretum 2019a). The 375 taxa of invasive alien plants in the country were divided into five classes according to the degree of spread: widespread species (WS), serious spread species (SS), spread risk species (SR), spread concern species (SC) and continuously spreading species (CS) (Jung et al. 2017). Of these, WS plants are those recorded at 101 sites or more and 231 (72.2%) of the 375 taxa fall into this category. A total of 18 taxa, including *Robiniapseudoacacia* and *Erigeronannuus*, were identified as WS plants during the survey. SS species are those with 75 to 100 distribution sites and 38 (11.9%) of the 375 taxa fall into this category. A total of nine taxa were identified as SS in the survey, including *Symphyotrichumpilosum* and *Festucaarundinacea*. SR species are those with 50 to 74 distribution sites and 18 (5.6%) out of 375 taxa fall under this category. A total of 13 taxa were identified as SR, including *Quamoclitangulata* and *Crassocephalumcrepidioides*. SC species are those with 25 to 49 distribution sites and 15 (4.7%) of 375 taxa fall into this category. A total of 18 taxa were identified as SC, including *Sicyosangulatus* and *Ambrosiatrifida*. The rate of spread of *Sicyosangulatus* and *Ambrosiatrifida* is very fast. Therefore, the Ministry of Environment designated them as invasive plants for which management measures should be developed as soon as possible. CS species are those with less than 24 distribution sites and 18 (5.6%) out of 375 taxa fall into this category and six taxa, including *Erigeronphiladelphicus* and *Ageratinaaltissima*, were identified as CS.

The most frequently recorded invasive alien plants were *Erigeronannuus* (202 records), followed by *Bidensfrondosa* (153 records), *Robiniapseudoacacia* (111 records), *Phytolaccaamericana* (61 records) and *Conyzacanadensis* (54 records). A total of 21 taxa, including *Sicyosangulatus* and *Solanumcarolinense*, appeared at only one site and 14 taxa, including *Ambrosiatrifida*, *Erigeronphiladelphicus* and *Cerastiumglomeratum*, appeared in two or three places. The frequency of occurrence of all 35 taxa was low, but most taxa identified tend to spread rapidly; therefore, further monitoring is required to determine the extent of spread and formulate medium- to long-term management measures to control these species.

### Categorisation according to wetland preference of vascular plants

Based on Choung et al. (2012), Choung et al. (2020), Choung et al. (2021), the plants identified in the survey were assigned to the following categories according to the frequency of appearance: obligate wetland plant (OBW), facultative wetland plant (FACW), facultative plant (FAC), facultative upland plant (FACU) and obligate upland plant (OBU). Consequently, OBW accounted for 152 taxa (11.0%), FACW 138 taxa (9.9%), FAC 177 taxa (12.7%), FACU 198 taxa (14.3%) and OBU 723 taxa (52.1%) (Suppl. material 11). For each category, the plants with the highest occurrence frequency in forest wetlands were examined. For OBW, *Persicariathunbergii* appeared 315 times, followed by Juncuseffuususvar.decipiens 226 times, *Phragmitesjaponica* 162 times, *Lycopuslucidus* 154 times and *Isachneglobosa* and *Aneilemakeisak* each 124 times. For FACW, *Salixkoreensis* appeared 311 times, followed by *Impatienstextorii* 235 times, *Thelypterispalustris* 189 times, *Persicariasagittata* 186 times and *Bidensfrondosa* 153 times. For FAC, *Oplismenusundulatifolius* appeared 269 times, followed by *Rosamultiflora* 239 times, *Equisetumarvense* 224 times, Amphicarpaeabracteatasubsp.edgeworthii 202 times and Acertataricumsubsp.Ginnala 192 times. For FACU, *Clematisapiifolia* appeared 230 times, followed by *Erigeronannuus* 202 times, *Fraxinusrhynchophylla* 170 times, *Commelinacommunis* 161 times and *Microstegiumvimineum* 152 times. For OBU, *Artemisiaprinceps* appeared 249 times, followed by *Zanthoxylumschinifolium* 224 times, *Ligustrumobtusifolium* 209 times, *Linderaobtusiloba* 158 times and *Rubuscrataegifolius* 156 times.

### Vascular flora by region

The flora of the 455 forest wetland sites in South Korea belonged to 143 families, 582 genera, 1,245 species, 26 subspecies, 104 varieties and 13 f., with a total of 1,388 taxa recorded. Amongst them, the flora of 132 sites in the Gangwon area belonged to 927 taxa (66.8%), including 113 families, 423 genera, 819 species, 23 subsp., 78 var. and seven f. and 151 taxa (about 10.9%), such as *Equisetumramosissimum*, were found to the Gangwon area. The flora of forest wetlands in 38 locations in the Gyeonggi region (including Incheon) was identified to consist of 555 taxa (about 40%), belonging to 98 families, 305 genera, 491 species, 14 subsp., 45 varieties and five f., amongst which 18 taxa (1.3%) including *Scirpusorientalis* were found to be endemic to the Gyeonggi area. The flora of the 116 forest wetlands in the Gyeongsang region belonged to 121 families, 417 genera, 719 species, 21 subspecies, 64 varieties and seven f., with a total of 811 taxa (about 58.4%). Of these, 71 taxa (about 5.1%), including *Parathelypterisbeddomei*, were identified to have appeared in the forest wetlands of the Gyeongsang area. The flora of the 50 forest wetlands in the Chungcheong region consisted of 667 taxa (about 48%) belonging to 104 families, 357 genera, 19 subspecies, 49 varieties and seven f. and 43 taxa, including *Reynoutriaforbesii*, were endemic to the forest wetlands of the Chungcheong region. A total of 770 taxa (about 55.5%) of 117 families, 397 genera, 686 species, 16 subspecies, 58 varieties and 10 f. were identified in the flora of 90 forest wetlands in the Jeolla region. In the Jeolla region alone, 93 taxa (about 6.7%) of endemic plants were identified, including *Lapsanastrumapogonoides*. Finally, the flora of the 29 forest wetlands in the Jeju area belonged to 81 families, 186 genera, 260 species, four subspecies, 13 varieties and two f., with a total of 279 taxa (about 20.1%) and 65 taxa (4.7%) including *Potamogetoncristatus* were identified to have appeared in the forest wetlands of the Jeju area. Many of the taxa distributed in each region were considered to be region-specific indicator species because their frequency of occurrence was as low as one to five locations and additional research on forest wetland region-specific indicator species is considered necessary.

#### Vascular flora in forest wetlands of Gangwon region

A total of 927 taxa, belonging to 113 families, 423 genera, 819 species, 23 subspecies, 78 varieties and seven f. were identified as vascular flora of the 132 forest wetlands in the Gangwon area (Suppl. material 18). *Persicariathunbergii* had the highest frequency of occurrence at 114, followed by *Salixpierotii* Miq. at 103, *Impatienstextorii* at 85 and *Fraxinusrhynchophylla* at 83. A total of 38 endemic plant taxa were identified, including *Pseudostellariasetulosa* and *Angelicapurpuraefolia*. Forty seven rare plant taxa were also recorded. Of these, three taxa are classified as CR: *Cicutavirosa*, *Eriophorumgracile* and *Magnoliakobus*. However, the *M.kobus*, as a species native to the Jeju-South region, is considered to have been planted. A total of four taxa were EN, including *Micranthesoctopetala*, *Menyanthestrifoliata*, Trientaliseuropaeavar.arctica and *Megaleranthissaniculifolia*; 15 taxa were VU, including *Galeariscyclochila* and *Tylophorafloribunda*; 20 taxa were LC, including *Aristolochiamanshuriensis* and Syringavillosasubsp.wolfii and five taxa were DD, including *Scrophulariakoraiensis*. Additionally, 245 taxa were floristic target plants. There were nine grade V taxa, including *Braseniaschreberi* and *Platantherahologlottis*; 41 grade IV taxa, including *Actaeabifida* and *Hanabusayaasiatica*; and 61 grade III taxa, including *Megaleranthissaniculifolia*, *Viciachosenensis* and *Carexlasiolepis*; 36 taxa were invasive alien plants, including *Chenopodiumalbum*. and *Dactylisglomerate*.

Of the 927 taxa identified in the forest wetlands of the Gangwon region, 151 taxa (about 16.3%) were confirmed to the forest wetlands of the Gangwon region (Suppl. material 12). Of the species found only inGangwon-do, the taxon that appeared most frequently was *Carpinuscordata* (19 records), followed by *Tiliaamurensis* (18 records), *Acerbarbinerve* (10 records), Impatienstextorivar.koreana (nine records) and *Salixrorida* (eight records). The following trees were documented to be distributed in areas considered to be south of central South Korea: *Dryopteriserythrosora* (Seo-myeon, Yangyang-gun), *Carpinusturczaninovii* (Daegwallyeong-myeon, Pyeongchang-gun), *Sinomeniumacutum* (Budong-myeon, Wonju-si), Viciaangustifoliavar.minor (Inje-eup, Inje-gun), *Erigeronfloribundus* (Wondeok-eup, Samcheok-si), *Arundodonax* (Sacheon-myeon, Gangneung-city) and *Carexlenta* (Gwirae-myeon, Wonju-si). In particular, as *Sinomeniumacutum* grows naturally around Jeju, the southern islands and the coasts, it was considered to have been either planted for medicinal purposes or originated from seeds transported with the sand for snow removal in winter. Viciaangustifoliavar.minor is another species that is distributed mainly along the coast and is likely to have spread along with the snow removal sand. *Sicyosangulatus*, designated as an invasive plant and other invasive alien plants, such as *Cerastiumglomeratum* and *Erigeronphiladelphicus*, have been introduced to one or two forest wetlands. A decrease in water supply due to climate change and terrestrialisation of forest wetlands pose a threat to them and will accelerate the spread of invasive alien plants and management measures are urgently required.

#### Vascular flora in forest wetlands of Gyeonggi region

A total of 555 taxa belonging to 98 families, 305 genera, 491 species, 14 subspecies, 45 varieties and five f. were identified within the 38 forest wetlands in the Gyeonggi region, which includes Gyeonggi-do and Incheon Metropolitan City. The most frequently occurring taxon was *Salixpierotii* (34 records), followed by *Oplismenusundulatifolius* (33 records), Acertataricumsubsp.Ginnala (30 records) and *Persicariathunbergii* and *Ligustrumobtusifolium* with 29 records each. There were 19 endemic taxa, including Carexsabynensisvar.leiosperma and *Polygonatuminfundiflorum*. Additionally, 17 rare plant taxa were identified, amongst which, six including *Irisminutoaurea* and *Rhododendronmicranthum* were VU, seven including *Arisaemaheterophyllum* and *Utriculariacaerulea* were LC and four including *Scirpusorientalis* were DD. A total of 97 floristic target plant taxa were recorded. One grade V taxon was identified, while 13 grade IV taxa, including *Rhamnusdavurica* and *Epimediumkoreanum*, 21 grade III taxa including *Cynanchumnipponicum* and *Centellaasiatica* and 16 invasive alien plants including *Bidensfrondosa* and *Phytolaccaamericana*, were recorded.

Of the 555 taxa that appeared in the forest wetlands of the Gyeonggi region, 18 (approximately 3.2%) were documented to be endemic (Suppl. material 14). *Elaeagnusglabra* was recorded three times, *Prunusjaponica*, *Magnoliaobovata*, *Hostaclausa* and *Galiumtokyoense* were recorded twice each and the remaining 14 taxa were recorded only once. *Elaeagnusglabra* was observed in Gyeonggi in study site 2019-3 (Yulgil-ri, Sang-myeon, Gapyeong-gun, Gyeonggi-do), Gyeonggi 2019-36 (Sineup-dong, Pocheon-si, Gyeonggi-do) and Gyeonggi 2019-37 (Hwahyeon-ri, Hwahyeon-myeon, Pocheon-si, Gyeonggi-do). In the Gyeonggi 2019-37 area, five taxonomic groups were surveyed, including *Elaeagnusglabra*, *Mukdeniarossii*, *Prunusjaponica*, Carexsabynensisvar.leiosperma,*Arabishirsuta*. and *Calamagrostispseudophragmites* (forest genetic resource reserves, Muui-dong, Jung-gu, Incheon), *Scirpusorientalis* and *Echinochloaoryzicola* appeared in the mountain valley wetland of Gyeonggi 2019-24 (Dowon-ri, Cheongun-myeon, Yangpyeong-gun, Gyeonggi-do). *Epimediumkoreanum* and Veratrummaackiivar.parviflorum were found in Gyeonggi 2019-25 (Sillon-ri, Cheongun-myeon, Yangpyeong-gun, Gyeonggi-do) and *Clematisbrachyura* in Gyeonggi 2019-05 (Wigok-ri, Seorak-myeon, Gapyeong-gun, Gyeonggi-do). The designation of forest genetic resource reserves, such as the one in Muui-dong, Incheon Metropolitan City and other effective regional infrastructure-based conservation measures must be promoted to systematically monitor and manage prime forest wetlands, such as Gyeonggi 2019-36.

#### Vascular flora of Gyeongsang region

A total of 811 taxa belonging to 121 families, 417 genera, 719 species, 21 subspecies, 64 varieties and seven f. were identified as the flora of the 116 forest wetlands in the Gyeongsang region. The most frequently occurring species were *Persicariathunbergii* and *Salixpierotii* (79 records each), followed by *Artemisiaindica* (73 records) and *Juncusdecipiens* with 72 records. A total of 27 taxa, including *Lespedezamaritima* and *Hostaminor*, were endemic plants. As for rare plants, two taxa *Utriculariauliginosa* and *Eriophorumgracile* were CR, two taxa *Hololeionmaximowiczii* and *Trigonotisradicans* were EN, 12 taxa including *Utriculariabifida* and *Inulasalicina* were VU, 12 taxa including *Penthorumchinense* and *Otteliaalismoides* were LC and five taxa, including *Chloranthusfortunei*, were DD. Additionally, 157 taxa were floristic target plants. Five grade V taxa, including *Eriophorumgracile* and *Asperulalasiantha*, were recorded, along with 17 grade IV taxa, including *Mimulustenellus* and *Cardamineyezoensis* and 30 grade III taxa, including *Cardaminekomarovii* and *Parathelypterisbeddomei*. Forty-two invasive alien plant taxa, including *Brizaminor* and *Trifoliumpratense*, were identified.

Of the 811 taxa identified in the forest wetlands of the Gyeongsang region, 71 taxa (about 8.8%), including *Parathelypterisbeddomei*, were endemic (Suppl. material 13). The most frequently occurring taxon was *Brizaminor* (four records), followed by *Viburnumcarlesii*, Euonymushamiltonianusvar.maackii and *Lespedezamaritima* with three records each. *Brizaminor* is a CS invasive alien plant which was recorded in four locations: Gyeongsangnam-do 2015-49 (Seon-ri, Wondong-myeon, Yangsan-si, Gyeongsangnam-do), Busan 2015-1 (Imgi-ri, Cheolma-myeon, Gijang-gun, Busan), Busan 2015-4 (Oeseok-ri, Sangbuk-myeon, Yangsan-si, Gyeongsangnam-do) and Ulsan 2015-11 (Jungsan-dong, Buk-gu, Ulsan). Each of these four forest wetlands is considered to have high conservation value because numerous notable plants, such as *Eriophorumgracile* and *Utriculariauliginosa*, are found there. In particular, the Gyeongsangnam-do 2015-49 area is a high-altitude forest wetland located at 954 m above sea level. The northern lineage plant *Eriophorumgracile* has been documented to occur there, although more precise monitoring is required. *Viburnumcarlesii* was recorded in forest wetlands located in three areas in Gunwi-gun, Gyeongsangbuk-do: Gyeongsangbuk-do 2017-9.10 (Unsan-ri, Sanseong-myeon, Gunwi-eup, Gyeongsangbuk-do), Gyeongsangbuk-do 2017-11 (Hwajeon-ri, Sanseong-myeon, Gunwi-gun, Gyeongsangbuk-do) and Gyeongsangbuk-do 2017-15 (Geumyang-ri, Uiheung-myeon, Gunwi-gun, Gyeongsangbuk-do). *Geraniumkrameri* and Euonymushamiltonianusvar.maackii appeared in two forest wetlands: Gyeongsangbuk-do 2017-16 (Subuk-ri, Uiheung-myeon, Gunwi-gun, Gyeongsangbuk-do) and Gyeongsangbuk-do 2017 47 (Ibam-ri, Donghae-myeon, Nam-gu, Pohang-si, Gyeongsangbuk-do). *Lespedezamaritima* was observed in three locations: Gyeongsangbuk-do 2017-2 (Nogok-ri, Naenam-myeon, Gyeongju-si, Gyeongsangbuk-do), Gyeongsangbuk-do 2017-9·10 (Unsan-ri, Sanseong-myeon, Gunwi-eup, Gyeongsangbuk-do) and Ulsan 2015-3 (Oegwang-ri, Onyang-eup, Ulju-gun, Ulsan).

A total of ten invasive alien plant taxa were found to be endemic to the Gyeongsang region. Of these, two taxa, *Lepidiumvirginicum* and *Loliumperenne* were SS, two taxa *Rudbeckiabicolor* and *Cosmosbipinnatus* were SR, four taxa, including *Medicagopolymorpha* and *Hibiscustrionum*, were SC and two taxa *Elymusrepens* and *Brizaminor* were CS. Thus, the development of management measures for preventing their spread is urgent.

#### Vascular flora of Chungcheong region

A total of 667 taxa belonging to 104 families, 357 genera, 592 species, 19 subspecies, 49 varieties and seven f. were identified as the flora of the forest wetlands at 50 sites in the Chungcheong region. The frequency of occurrence from highest to lowest was as follows: *Artemisiaindica* (41 records), *Persicariathunbergii* (40 records), *Salixpierotii* (40 records), *Oplismenusundulatifolius* (39 records) and *Equisetumarvense* (37 records). A total of 25 taxa of endemic plants were observed, including *Fraxinuschiisanensis* and Irisrossiivar.latifolia J. As for rare plants, two taxa *Carexcapricornis* and Prunus×yedoensis were CR. However, additional research is considered necessary for Prunus×yedoensis. Four taxa, including *Utriculariajaponica* were VU, seven taxa including Gentianatrifloravar.japonica and *Tricyrtismacropoda* were LC and five taxa, including Eleutherococcusdivaricatusvar.chiisanensis (Nakai), were DD. A total of 113 taxa were floristic target plants, including two grade V taxa *Utriculariajaponica* and *Asperulalasiantha*, ten grade IV taxa, including *Rodgersiapodophylla* and *Aegopodiumalpestre* and 26 grade III taxa, including *Catolobuspendulus* and Veratrummaackiivar.japonicum. Twenty-nine invasive alien plant taxa, including *Rumexobtusifolius* and *Carduuscrispus*, were also recorded.

Of the 667 taxa identified in the Chungcheong region, 43 taxa (about 6.4%), including *Dryopterissacrosancta*, *Fallopiaforbesii* and *Loniceraharae* were endemic to the Chungcheong region (Suppl. material 15) and were recorded only once or twice. Four taxa including *Indigoferapseudotinctoria*, *Dryopterissacrosancta* and *Primulasieboldii* were recorded twice and 39 taxa including *Broussonetiapapyrifera* and *Triporadivaricata* were recorded only once. The current distribution status of the 43 taxa that were endemic to the Chungcheong region showed that eight taxa were recorded in Chungcheongnam-do 2016 12/13/14 (Daedong-ri, Yeomchi-eup, Asan-si, Chungcheongnam-do), including *Ixerisdebilis* and *Solidagogigantea*. Therefore, management measures for *Solidagogigantea* are required. Six taxa, including *Themedatriandra*. and Viciaunijugavar.ouensanensis, were recorded in Chungcheongnam-do 2016-05 (Cheongnae-ri, Jewon-myeon, Geumsan-gun, Chungcheongnam-do) and Chungcheongbuk-do 2017-34 (Jidong-ri, Dongnyang-myeon, Chungju-si, Chungcheongbuk-do), respectively.

#### Vascular flora of Jeolla region

A total of 770 taxa belonging to 117 families, 397 genera, 686 species, 16 subspecies, 58 varieties and ten f. were recorded in 90 forest wetlands in the Jeolla region. The most frequently occurring taxa were: *Oplismenusundulatifolius* (65 records), *Rosamultiflora* (57 records), *Salixpierotii* (53 records), *Ligustrumobtusifolium* (52 records) and *Styraxjaponicus* (51 records). Twenty-eight taxa, including *Stewartiakoreana* and *Carexerythrobasis*, were identified as endemic to the region. A total of 38 rare plant taxa were recorded, of which five, including *Utriculariaaurea* and *Habenariaradiata*. were CR, two including *Hololeionmaximowiczii* and *Ophioglossumvulgatum* were EN, 12 including *Pogoniaminor* and *Sparganiumstoloniferum* were VU, 14 including *Melothriajaponica* and *Linderasericea* were LC and five including *Hypericumattenuatum* were DD. Additionally, 162 floristic target plant taxa were recorded, including eight grade V taxa including *Arundinariamunsuensis* and *Nephrolepiscordifolia*, 18 grade IV taxa including *Dryopteristokyoensis* and *Loniceracaerulea* and 29 grade III taxa including *Salviajaponica* and *Teucriumveronicoides*. Twenty-two invasive alien plant taxa, including *Erechtiteshieraciifolius* and *Tagetesminuta*, were recorded.

Of the 770 taxa found in the Jeolla region, 90 taxa (about 11.7%), including *Cephalotaxusharringtonia* and *Ajugadecumbens*, were found to be endemic. *Scleriaparvula*, *Hemerocallisthunbergii*, *Vacciniumoldhamii* and *Hypericumattenuatum* were recorded five times, *Ischaemumanthephoroides*, *Pogoniaminor*, and *Melothriajaponica* four times and *Agrostiscanina* and *Euscaphisjaponica* three times. All taxa recorded three, four or five times were found to be endemic to the Jeollanam-do region (Suppl. material 16). Eleven taxa, including *Carexscabrifolia*, were recorded in Jeollanam-do 2016-25 (Hanwoon-ri, Jaeun-myeon, Sinan-gun, Jeollanam-do), nine including *Chionanthusretusus* in Jeollanam-do 2016-15 (Cheongcheon-ri, Cheonggye-myeon, Muan-gun, Jeollanam-do) and seven including *Lophatherumgracile* in Jeollanam-do 2016-01 (Deoknyeon-ri, Doam-myeon, Gangjin-gun, Jeollanam-do).

#### Vascular flora of Jeju region

A total of 279 taxa belonging to 81 families, 186 genera, 260 species, four subsp., 13 var. and two f. were identified as the flora of the 29 forest wetlands in the Jeju region. The most frequently recorded taxa were: *Schoenoplectiellatriangulata* (15 records), *Persicariapraetermissa* (15 records), *Rosamultiflora* (14 records), *Carexdimorpholepis* (13 records) and *Isachneglobosa* (11 records). Eight taxa, including *Maackiafauriei* and *Cirsiumrhinoceros*, were identified as endemic plants. Fourteen rare plant taxa were recorded, with two taxa *Mankyuachejuensis*, and *Utriculariauliginosa* classified as CR, four taxa including *Cynanchumamplexicaule* and *Nymphoidescoreana* as EN, four taxa including Swertiadilutavar.tosaensis and *Tripterospermumjaponicum* as VU, one taxon *Acoruscalamus* as LC and three taxa including *Apocynumcannabinum* as DD. Sixty-eight floristic target plant taxa were recorded, of which five grade V taxa, including *Braseniaschreberi* were recorded, as well as nine grade IV taxa including *Persicariataquetii* and *Eleocharisdulcis* and 17 grade III taxa including *Ilexcrenata* and *Daphniphyllummacropodum*. Twelve invasive alien plant taxa were identified, including *Ambrosiaartemisiifolia* and *Sisyrinchiumrosulatum*.

Of the 279 taxa recorded in the forest wetlands of the Jeju region, 65 taxa (about 23.3%) including *Potamogetoncristatus* and Rubus buergeri appeared to be endemic (Suppl. material 17). About 23% of taxa endemic to the Jeju region were considered to be floristic target plants. The most frequently occurring of these taxa were: *Mankyuachejuensis*, (seven records), *Ludwigiaovalis* and *Violalactiflora*, *Maackiafauriei*, *Cynanchumamplexicaule* and *Nymphoidesindica* (five records each), *Cirsiumrhinoceros* (four records) and *Potamogetoncristatus*, *Marsileaquadrifolia*, *Sasaquelpaertensis* and *Alliumtaquetii* (three records each). By region, 31 taxa, including *Viburnumfurcatum*, were identified in Jeju 2015-28 (Sumeunmulbaengdui, Gwangnyeong-ri, Aewol-eup, Jeju-si), nine taxa including *Potamogetoncristatus* in Jeju 2015-25 (Geumoreum, Geumak-ri, Hanlim-eup, Jeju-si) and eight taxa including *Gentianasquarrosa* in Jeju 2015-29 (Bongseong-ri, Aewol-eup, Jeju-si).

## Disscussion

### Plant distribution characteristics of forest wetlands

The most frequently appearing endemic plants in the 455 wetlands were *Weigelasubsessilis* and *Salixkoriyanagi*, which appeared in 111 wetlands. Wetlands with the most frequent occurrence of endemic plants were Jeollanam-do 2016-32, Chungcheongbuk-do 2017-16, Gangwon-do 2019-30 and Gangwon-do 2019-33, with eight taxa recorded in each region. In Jeollanam-do 2016-32, *Cirsiumsetidens*, *Scutellariainsignis*, *Weigelasubsessilis*, *Thalictrumactaeifolium*, *Lysimachiacoreana*, *Salixkoriyanagi*, *Scrophulariakoraiensis* and *Chrysospleniumbarbatum* were recorded and in Chungcheongbuk-do 2017-16, *Cirsiumsetidens*, *Stewartiakoreana*, *Fraxinuschiisanensis*, *Weigelasubsessilis*, *Violaseoulensis*, *Thalictrumactaeifolium*, *Aconitumpseudolaeve* and *Carexerythrobasis* were recorded. In Gangwon-do 2019-30, *Asperulalasiantha*, *Angelicareflexa*, *Cirsiumsetidens*, *Fraxinuschiisanensis*, *Pseudostellariasetulosa*, *Angelicapurpuraefolia*, *Lysimachiacoreana* and *Saussureagrandicapitula* were recorded and in Gangwon-do 2019-33, *Asperulalasiantha*, *Cirsiumsetidens*, *Pseudostellariasetulosa*, *Angelicapurpuraefolia*, *Aconogononmicrocarpum*, *Lysimachiacoreana*, *Carexerythrobasis* and *Chrysospleniumbarbatum* were recorded.

The wetlands where the greatest number of rare plants were identified were in Chungcheongbuk-do 2017-23 and Gangwon-do 2018-53. In Chungcheongbuk-do 2017-23, *Carexcapricornis*. (CR), *Mimulustenellus* (VU), Gentianatrifloravar.japonica (LC) and *Rumexlongifolius* (DD) were found. In Gangwon-do 2018-53, a total of eight taxa appeared: *Megaleranthissaniculifolia* and *Micranthesoctopetala* (EN), *Galeariscyclochila* (VU), *Violaalbida*, Gentianatrifloravar.japonica, *Parasenecioauriculatus*, *Liliumdistichum* and *Trilliumcamschatcense*.

Analysis of the distribution characteristics of the rare plants by region and altitude showed that *Mankyuachejuense*, classified as CR, appeared only in seven forest wetlands at 160 m alt. or below in Jeju-do and *Cicutavirosa* appeared only in six forest wetlands in Gangwon-do, although there was no altitude restriction. *Utriculariauliginosa* appeared only in the southern regions of Gyeongsangnam-do and Jeollanam-do. *Eriophorumgracile* appeared only in the forest wetlands of Gangwon-do, which were located at high altitudes. As wetlands with high OBW occurrence rates relative to the occurrence of other species, three forest wetland sites in Jeju-do showed the presence of OBW: Jeju 2015-07, Jeju 2015-10 and Jeju 2015-17. In Jeju 2015-07, ten taxa were identified: *Potamogetondistinctus*, *Trapajaponica*, *Triadenumjaponicum*, Monochoriavaginalisvar.plantaginea, *Typhaorientalis*, *Schoenoplectiellatriangulata*, *Braseniaschreberi*, *Nymphoidesindica*, *Schoenoplectustabernaemontani* and *Utriculariajaponica*. In Jeju 2015-10, two taxa were identified: *Trapajaponica* and *Schoenoplectiellatriangulata*. In Jeju 2015-17, three taxa were identified: *Spirodelapolyrrhiza*, *Trapajaponica* and *Wolffiaarrhiza*. In contrast, there were eight wetlands with high OBU occurrence rates relative to the occurrence rates of other species sites, where only OBU were recorded: Gyeongsangbuk-do 2017-44 (20 taxa), Gyeongsangbuk-do 2018-09 (24 taxa), Jeollanam-do 2016-07 (14 taxa), Jeollabuk-do 2016-10 (11 taxa), Jeollabuk-do 2016-20 (18 taxa), Jeollabuk-do 2016-21 (36 taxa), Chungcheongbuk-do 2017-02 (47 taxa) and Chungcheongbuk-do 2017-18 (16 taxa). These forest wetlands were located at altitudes of up to 500 m above sea level, which made them highly accessible and they are considered to be wetlands that are undergoing terrestrialisation and losing their function as forest wetlands.

### Forest wetland conservation measures

The notable plants recorded in the 455 forest wetlands included eight taxa of grade II endangered wild plants, 202 taxa of northern plants in the Korean Peninsula, 66 taxa of endemic plants of the Korean Peninsula, 94 taxa of IUCN-designated rare plants and 411 taxa of floristic regional indicator plants. In forest wetlands where eight taxa of native endangered wild plants (grade II) occur (*Mankyuachejuense*, *Cicutavirosa*, *Utriculariauliginosa*, *Habenariaradiata*, *Menyanthestrifoliata*, Trientaliseuropaeavar.arctica, *Braseniaschreberi* and *Eleutherococcussenticosus*), it is necessary to designate the area as a conservation area through an immediate review of its feasibility or to establish a conservation plan by installing information boards and training local observers. In forest wetlands where native rare plants classified as VU, EN and CR occur, 1-14 forest wetlands should be designated by the Korea Forest Service as Forest Genetic Resources Reserves (FGRR) to be monitored for a cycle of three years. For 197 taxa of floristic target plants of grade III and above, precise monitoring around the native habitat should be conducted periodically.

A total of 64 invasive alien plant taxa were identified in 455 forest wetland sites. The species with the highest frequency of occurrence was *Erigeronannuus* (202 records), followed by *Bidensfrondosa* (153 records), *Robiniapseudoacacia* (111 records), *Phytolaccaamericana* (61 records) and *Conyzacanadensis* (54 records). *Erigeronannuus* was identified at 65 (56%) of the 116 sites in the Gyeongsang region, but it was recorded most frequently in the Gyeongsangbuk-do region, occurring at 50 (73.5%) of the 68 sites. In the Chungcheong region, it appeared at 26 (52%) of the 50 sites, 63 (47.7%) of the 132 sites in the Gangwon-do region and 18 (47.3%) of the 38 sites in the captal area. *Erigeronannuus* was mostly observed around Gyeongsangbuk-do, Chungcheongnam-do, Chungcheongbuk-do, Gyeonggi-do and Gangwon-do. In the southern regions of Jeju, Jeollanam-do and Gyeongsangnam-do, *Erigeronannuus* as FACU was considered to have spread slowly owing to the ease of water supply even in winter in southern regions, such as Jeju, Jeollanam-do and Gyeongsangnam-do. However, for forest wetlands in the northern part of the country, the spread is expected to be more active because the moisture supply is insufficient in winter. *Bidensfrondosa* had a similar distribution pattern. It was found in 18 (48.6%) of 37 sites in the Gyeonggi region and in 48 (41.4%) of 116 sites in the Gyeongsang region and had spread to 42 sites (61.8%) in the Gyeongsangbuk-do region. It also occurred in the Gangwon-do and Chungcheong-do regions at 36.4% and 30% of the sites in each region, respectively. *Robiniapseudoacacia* was found in the following regions, excluding Jeju: Chungcheong (38%), Gangwon (31.1%), capital area (26.3%), Gyeongsang (25%) and Jeolla (13.3%). *Robiniapseudoacacia* is an OBU that often appears at the boundaries of forest wetlands. As it is known not to spread into the interior of forest wetlands, it can serve as an indicator plant for changes in the size of forest wetland areas. In contrast, *Sicyosangulatus* (FACW) was recorded in one location in Gangwon 2019-36 area during the second survey of forest wetlands conducted from 2015 to 2019, but its spreading speed is very fast and it is necessary to pre-empt its spread through monitoring and control measures.

Amongst 455 forest wetlands, 23 were classified as deformed wetlands due to natural disasters and terrestrialisation; 323 sites (approximately 71%) are privately owned and difficult to manage. Additionally, only 20% (91 sites) are currently designated as forest genetic resource conservation areas (Korea National Arboretum 2019b). Therefore, further designation of FGRRs is required. Application of other effective area-based conservation measures (OECMs) does not involve designating conservation areas, but is defined as the management of geographically limited areas over long periods, intending to maintain positive and sustainable in situ conservation of biodiversity, along with relevant ecosystem functions and services and cultural, spiritual, socio-economic and other locally relevant values (IUCN World Commission on Protected Areas (WCPA) 2019). In South Korea, seven areas of use, including forest wetlands and algific slopes, were reported to be suitable for OECMs. Therefore, it is necessary to pre-emptively designate 140 of the remaining 364 sites as FGRRs and apply OECMs and to seek an integrated management plan with systematic and regular monitoring under the administration of government agencies.

## Conclusions

This study was carried out to investigate the customary vascular flora of 455 forest wetlands in South Korea, to survey for mid- and long-term conservation and utilisation and to prepare basic data necessary for the restoration of the forest wetlands in the future under climate change. The vascular flora of 455 sites was identified and included 1,388 taxa belonging to 143 families, 582 genera, 1,246 species, 26 subspecies, 104 varieties and 12 forma. The most frequently occurring species in South Korea's forest wetlands were: *Persicariathunbergii* (315 records), *Salixpierotii* (311 records), *Oplismenusundulatifolius* (269 records), *Artemisiaindica* (249 records), *Impatienstextorii* (235 records) and *Clematisapiifolia* (235 records).

A range of notable plants was also recorded. Eight taxa of grade II endangered wild plants were identified, including *Menyanthestrifoliata* and *Mankyuachejuense*. A total of 220 taxa were identified as northern lineage plants, North Korean plants of the Korean Peninsula. A total of 66 taxa were identified as endemic plants of the Korean Peninsula. *Salixkoriyanagi* and *Weigelasubsessilis* each had 111 records. The rare plants designated by the Korea Forest Service recorded during the survey consisted of 94 taxa. A total of eight taxa, including *Carexcapricornis* were CR, 10 taxa including *Micranthesoctopetala* were EN, 31 taxa including *Utriculariabifida* were VU.

A total of 411 floristic target plant taxa were recorded. Thirty-one limestone area plant taxa were recorded including *Stemmacanthauniflora* and 64 invasive alien plant taxa were also recorded. Eighteen taxa, including *Erigeronannuus* were WS, nine taxa including *Symphyotrichumpilosum* were SS, 13 taxa including *Quamoclitangulate* were SR, 18 taxa including *Sicyosangulatus* were SC and six taxa including *Ageratinaaltissima* were CS. The most frequently occurring invasive alien plants in the 455 forest wetlands were *Erigeronannuus* with 202 records, followed by *Bidensfrondosa* with 153 records, *Robiniapseudoacacia* with 111 records, *Phytolaccaamericana* L. with 61 records and *Conyzacanadensis* with 54 records.

All vascular plants recorded were classified according to their wetland preference: 152 taxa (11.0%) were OBW, 138 taxa (9.9%) were FACW, 177 taxa (12.7%) were FAC, 198 taxa (14.3%) were FACU and 723 (52.1%) were OBU.

The distribution of vascular plants by region was also recorded. The flora at 132 sites in the Gangwon region consisted of 927 taxa. At 38 sites in the Gyeonggi region, 555 taxa were identified. At 116 sites in the Gyeongsang region, 811 taxa were identified. At 50 sites in the Chungcheong region, 667 taxa were identified. At 90 sites in the Jeolla region, 770 taxa were identified. At 29 sites in the Jeju region, 279 taxa were identified. At the 140 sites assessed to be of much higher degree amongst the 364 sites, pre-emptive designation as FGRRs and application of OECMs are required. Integrated management measures for the OECMs must be sought through systematic and regular monitoring under the administration of government agencies.

## Supplementary Material

3C74F015-FBA7-5D1C-A41C-493B53CBFA33 10.3897/BDJ.10.e85848.suppl1 Supplementary material 1 Survey site distribution by region according to grades of forest wetland Data type etc. File: oo_667188.docx https://binary.pensoft.net/file/667188 Lee, Jong-Won, Ho-Geun Yun, Tae Young Hwang, Jong Bin An

58BF75D0-4088-59D4-843E-67B55DCF2827 10.3897/BDJ.10.e85848.suppl2 Supplementary material 2 The list of vascular plants in forest wetlands of Korea. Data type etc. File: oo_667190.docx https://binary.pensoft.net/file/667190 Lee, Jong-Won, Ho-Geun Yun, Tae Young Hwang, Jong Bin An

4DA0F22C-C960-5CAF-9C7C-68CED36E3C15 10.3897/BDJ.10.e85848.suppl3 Supplementary material 3 The list of Korean endemic plants in forest wetlands of Korea. Data type etc. File: oo_667194.docx https://binary.pensoft.net/file/667194 Lee, Jong-Won, Ho-Geun Yun, Tae Young Hwang, Jong Bin An

701268E5-B0A1-5CC2-8468-876DC183D6FD 10.3897/BDJ.10.e85848.suppl4 Supplementary material 4 Rare plants by IUCN identified during the survey. Data type etc. File: oo_667192.docx https://binary.pensoft.net/file/667192 Lee, Jong-Won, Ho-Geun Yun, Tae Young Hwang, Jong Bin An

87C22692-2C7F-52CE-A762-DC20B039758F 10.3897/BDJ.10.e85848.suppl5 Supplementary material 5 The list of endangered wild plants in forest wetlands of Korea. Data type etc. File: oo_667193.docx https://binary.pensoft.net/file/667193 Lee, Jong-Won, Ho-Geun Yun, Tae Young Hwang, Jong Bin An

292A99A9-AEAE-581F-A991-F22E26BA4659 10.3897/BDJ.10.e85848.suppl6 Supplementary material 6 Floristic tartget plants of grade V plants identified in the survey. Data type etc. File: oo_667195.docx https://binary.pensoft.net/file/667195 Lee, Jong-Won, Ho-Geun Yun, Tae Young Hwang, Jong Bin An

DFF2ED98-EA2B-56BE-B526-1A627EBF272E 10.3897/BDJ.10.e85848.suppl7 Supplementary material 7 Floristic target plants of grade IV plants identified in the survey. Data type etc. File: oo_667198.docx https://binary.pensoft.net/file/667198 Lee, Jong-Won, Ho-Geun Yun, Tae Young Hwang, Jong Bin An

E303C006-C0AA-5827-A63C-8AFB622957B9 10.3897/BDJ.10.e85848.suppl8 Supplementary material 8 Floristic target plants of grade III identified in the survey. Data type etc. File: oo_667197.docx https://binary.pensoft.net/file/667197 Lee, Jong-Won, Ho-Geun Yun, Tae Young Hwang, Jong Bin An

EF10CFA9-5214-5BF6-A805-590901A25108 10.3897/BDJ.10.e85848.suppl9 Supplementary material 9 Limestone area plants identified in the survey. Data type etc. File: oo_667199.docx https://binary.pensoft.net/file/667199 Lee, Jong-Won, Ho-Geun Yun, Tae Young Hwang, Jong Bin An

4BF3687F-BBC2-517B-BC0D-9576D57BBDB3 10.3897/BDJ.10.e85848.suppl10 Supplementary material 10 Invasive alien plants identified in the survey. Data type etc. File: oo_667200.docx https://binary.pensoft.net/file/667200 Lee, Jong-Won, Ho-Geun Yun, Tae Young Hwang, Jong Bin An

BA3FA469-4B7B-5E9C-A60C-8EA293F3599A 10.3897/BDJ.10.e85848.suppl11 Supplementary material 11 Categorizing vascular plant species occurring in wetland ecosystems of the Korean Peninsula by frequency of occurrence in the study area. Data type etc. Brief description Choung et al. (2012) File: oo_667201.docx https://binary.pensoft.net/file/667201 Lee, Jong-Won, Ho-Geun Yun, Tae Young Hwang, Jong Bin An

D51B80F1-640B-5308-80BD-B7E2C0E615C7 10.3897/BDJ.10.e85848.suppl12 Supplementary material 12 Vascular plants recorded only in forest wetlands of Gangwon region, Korea. Data type etc File: oo_667202.docx https://binary.pensoft.net/file/667202 Lee, Jong-Won, Ho-Geun Yun, Tae Young Hwang, Jong Bin An

5EAC8D5D-45E6-5ECF-B9E7-679D5FCBA126 10.3897/BDJ.10.e85848.suppl13 Supplementary material 13 Vascular plants recorded only in forest wetlands of Gyeonggi region, Korea. Data type etc File: oo_667203.docx https://binary.pensoft.net/file/667203 Lee, Jong-Won, Ho-Geun Yun, Tae Young Hwang, Jong Bin An

2E85E28E-95CA-5498-93D9-86359DCAB181 10.3897/BDJ.10.e85848.suppl14 Supplementary material 14 Vascular plants recorded only in forest wetlands of Gyeongsang region Data type etc File: oo_667204.docx https://binary.pensoft.net/file/667204 Lee, Jong-Won, Ho-Geun Yun, Tae Young Hwang, Jong Bin An

90FF6725-3C90-501E-AD5E-4A3CAD225D1A 10.3897/BDJ.10.e85848.suppl15 Supplementary material 15 Vascular plants recorded only in forest wetlands of Chungcheong region, Korea. Data type etc File: oo_667206.docx https://binary.pensoft.net/file/667206 Lee, Jong-Won, Ho-Geun Yun, Tae Young Hwang, Jong Bin An

41AA926D-B8E7-5830-A420-E51263FE89EF 10.3897/BDJ.10.e85848.suppl16 Supplementary material 16 Vascular plants that appeared only in forest wetlands of Jeolla region, Korea. Data type etc File: oo_667207.docx https://binary.pensoft.net/file/667207 Lee, Jong-Won, Ho-Geun Yun, Tae Young Hwang, Jong Bin An

36948FDF-7B22-535B-9397-09997F9B7EB0 10.3897/BDJ.10.e85848.suppl17 Supplementary material 17 Vascular plants recorded only in forest wetlands of Jeju region, Korea. Data type etc. File: oo_667208.docx https://binary.pensoft.net/file/667208 Lee, Jong-Won, Ho-Geun Yun, Tae Young Hwang, Jong Bin An

83A74D28-2400-50EE-A1E4-A9866B945F77 10.3897/BDJ.10.e85848.suppl18 Supplementary material 18 The total list of vascular plants of forest wetlands in Korea. Data type etc File: oo_667209.docx https://binary.pensoft.net/file/667209 Lee, Jong-Won, Ho-Geun Yun, Tae Young Hwang, Jong Bin An
