# Supplementary material for: Floristic inventory and distribution characteristics of vascular plants in forest wetlands of South Korea
Source: Biodivers Data J. 2022 Sep 15;10:e85848. doi: 10.3897/BDJ.10.e85848 (PMC9848468; doi:10.3897/BDJ.10.e85848)
Supplement: Supplementary material 1 — Survey site distribution by region according to grades of forest wetland [file bdj-10-e85848-s001.docx]

Table 1. Survey site distribution by region according to grades of forest wetland

| **Location** | **A** | **B** | **C** | **D** | **Total** |
| --- | --- | --- | --- | --- | --- |
| Gyeonggi | · | 20 | 12 | 5 | 37 |
| Gangwon | 8 | 64 | 52 | 8 | 132 |
| Chungbuk | 2 | 14 | 14 | 4 | 34 |
| Chungnam | · | 7 | 9 | · | 16 |
| Gyeongbuk | 1 | 33 | 30 | 4 | 68 |
| Gyeongnam | 3 | 15 | 15 | 1 | 34 |
| Jeonbuk | 1 | 13 | 9 | 9 | 32 |
| Jeonnam | 5 | 23 | 24 | 4 | 56 |
| Jeju | 10 | 7 | 11 | 1 | 29 |
| Busan | · | · | 3 | · | 3 |
| Incheon | · | 1 | · | · | 1 |
| Daegu | · | 2 | 1 | · | 3 |
| Gwangju | · | 1 | 1 | · | 2 |
| Ulsan | · | 1 | 3 | 4 | 8 |
| Total | 30 | 201 | 184 | 40 | 455 |

*A~D Grade.
