# Supplementary material for: Floristic inventory and distribution characteristics of vascular plants in forest wetlands of South Korea
Source: Biodivers Data J. 2022 Sep 15;10:e85848. doi: 10.3897/BDJ.10.e85848 (PMC9848468; doi:10.3897/BDJ.10.e85848)
Supplement: Supplementary material 2 — The list of vascular plants in forest wetlands of Korea. [file bdj-10-e85848-s002.docx]

**Table 2.** The list of vascular plants in forest wetlands of Korea.

| System/Taxa | Fam. | Gen. | Sp. | Sub. | Var. | For. | Total(%) |
| --- | --- | --- | --- | --- | --- | --- | --- |
| Pteridophyta | 19 | 32 | 69 | 0 | 3 | 0 | 72(5.2) |
| Gymnospermae | 5 | 8 | 14 | 0 | 0 | 0 | 14(1.0) |
| Angiospermae | 119 | 543 | 1163 | 26 | 101 | 12 | 1,302(93.8) |
| Dicotyledons | 101 | 398 | 819 | 23 | 75 | 8 | 925(66.6) |
| Monocotyledons | 18 | 145 | 344 | 3 | 26 | 4 | 377(27.2) |
| Total | 143 | 582 | 1246 | 26 | 104 | 12 | 1,388 |
