# Supplementary material for: Floristic inventory and distribution characteristics of vascular plants in forest wetlands of South Korea
Source: Biodivers Data J. 2022 Sep 15;10:e85848. doi: 10.3897/BDJ.10.e85848 (PMC9848468; doi:10.3897/BDJ.10.e85848)
Supplement: Supplementary material 3 — The list of Korean endemic plants in forest wetlands of Korea. [file bdj-10-e85848-s003.docx]

Table 6. Floristic tartget plants of grade V plants identified in the survey.

| Family name | Scientific name / Korean name | IUCN category | Frequency |
| --- | --- | --- | --- |
| Ophioglossaceae | *Mankyua chejuense* B. Y. Sun, M. H. Kim & C. H. Kim 제주고사리삼 | CR | 7 |
| Magnoliaceae | *Magnolia kobus* DC. 목련 | CR | 2 |
| Apiaceae | *Cicuta virosa* L. 독미나리 | CR | 5 |
| Dryopteridaceae | *Dryopsis maximowicziana* (Miq.) C. Chr. 흰비늘고사리 |  | 1 |
| Lentibulariaceae | *Utricularia aurea* Lour. 들통발 | CR | 1 |
| Nephrolepidaceae | *Nephrolepis cordifolia* (L.) C. Presl 줄고사리 |  | 1 |
| Lentibulariaceae | *Utricularia uliginosa* Vahl 자주땅귀개 | CR | 6 |
| Cyperaceae | *Eriophorum gracile* Koch 작은황새풀 | CR | 2 |
| Orchidaceae | *Habenaria radiata* (Thunb.) Spreng. 해오라비난초 | CR | 1 |
| Ranunculaceae | *Actaea heracleifolia* (Kom.) J. Compton 승마 |  | 11 |
| Primulaceae | *Trientalis europaea* L. var. *arctica* (Fisch. ex Hook.) Ledeb. 기생꽃 | EN | 1 |
| Menyanthaceae | *Menyanthes trifoliata* L. 조름나물 | EN | 2 |
| Rubiaceae | *Asperula lasiantha* Nakai 갈퀴아재비 |  | 6 |
| Cabombaceae | *Brasenia schreberi* J. F. Gmel. 순채 | VU | 8 |
| Lentibulariaceae | *Utricularia japonica* Makino 통발 | VU | 5 |
| Poaceae | *Arundinaria munsuensis* Y. N. Lee 문수조릿대 |  | 1 |
| Orchidaceae | *Platanthera hologlottis* Maxim. 흰제비란 |  | 5 |
