# Supplementary material for: Floristic inventory and distribution characteristics of vascular plants in forest wetlands of South Korea
Source: Biodivers Data J. 2022 Sep 15;10:e85848. doi: 10.3897/BDJ.10.e85848 (PMC9848468; doi:10.3897/BDJ.10.e85848)
Supplement: Supplementary material 4 — Rare plants by IUCN identified during the survey. [file bdj-10-e85848-s004.docx]

Table 4. Rare plants by IUCN identified during the survey.

| Family name | Scientific name / Korean name | Grade | Frequency |
| --- | --- | --- | --- |
| Ophioglossaceae | *Mankyua chejuense* B. Y. Sun, M. H. Kim & C. H. Kim 제주고사리삼 | CR | 7 |
| Magnoliaceae | *Magnolia kobus* DC. 목련 | CR | 2 |
| Apiaceae | *Cicuta virosa* L. 독미나리 | CR | 5 |
| Lentibulariaceae | *Utricularia aurea* Lour. 들통발 | CR | 1 |
| Lentibulariaceae | *Utricularia uliginosa* Vahl 자주땅귀개 | CR | 6 |
| Cyperaceae | *Carex capricornis* Meinsh. ex Maxim. 양뿔사초 | CR | 1 |
| Cyperaceae | *Eriophorum gracile* Koch 작은황새풀 | CR | 2 |
| Orchidaceae | *Habenaria radiata* (Thunb.) Spreng. 해오라비난초 | CR | 1 |
| Ophioglossaceae | *Ophioglossum vulgatum* L. 나도고사리삼 | EN | 2 |
| Ranunculaceae | *Megaleranthis saniculifolia* Ohwi 모데미풀 | EN | 1 |
| Saxifragaceae | *Micranthes octopetala* (Nakai) Y. I. Kim & Y. D. Kim 구실바위취 | EN | 5 |
| Primulaceae | *Trientalis europaea* L. var. *arctica* (Fisch. ex Hook.) Ledeb. 기생꽃 | EN | 1 |
| Menyanthaceae | *Menyanthes trifoliata* L. 조름나물 | EN | 2 |
| Menyanthaceae | *Nymphoides coreana* (H. Lév.) H. Hara 좀어리연꽃 | EN | 1 |
| Apocynaceae | *Cynanchum amplexicaule* (Siebold & Zucc.) Hemsl. 솜아마존 | EN | 5 |
| Boraginaceae | *Trigonotis radicans* (Turcz.) Steven 거센털꽃마리 | EN | 1 |
| Asteraceae | *Cirsium rhinoceros* (H. Lév. & Vaniot) Nakai 바늘엉겅퀴 | EN | 4 |
| Asteraceae | *Hololeion maximowiczii* Kitam. 께묵 | EN | 6 |
| Dryopteridaceae | *Dryopteris tokyoensis* (Matsum. ex Makino) C.Chr. 느리미고사리 | VU | 3 |
| Taxaceae | *Taxus cuspidata* Siebold & Zucc. 주목 | VU | 1 |
| Ranunculaceae | *Actaea bifida* (Nakai) J. Compton 세잎승마 | VU | 4 |
| Berberidaceae | *Epimedium koreanum* Nakai 삼지구엽초 | VU | 1 |
| Cabombaceae | *Brasenia schreberi* J. F. Gmel. 순채 | VU | 8 |
| Paeoniaceae | *Paeonia japonica* (Makino) Miyabe & Takeda 백작약 | VU | 1 |
| Droseraceae | *Drosera rotundifolia* L. 끈끈이주걱 | VU | 14 |
| Araliaceae | *Eleutherococcus senticosus* (Rupr. & Maxim.) Maxim. 가시오갈피 | VU | 2 |
| Ericaceae | *Rhododendron micranthum* Turcz. 꼬리진달래 | VU | 3 |
| Gentianaceae | *Swertia diluta* (Turcz.) Benth. & Hook. f. var. *tosaensis* (Makino) H. Hara 개쓴풀 | VU | 5 |
| Gentianaceae | *Tripterospermum japonicum* (Siebold & Zucc.) Maxim. 덩굴용담 | VU | 1 |
| Apocynaceae | *Tylophora floribunda* Miq. 왜박주가리 | VU | 6 |
| Lamiaceae | *Mosla japonica* (Benth. ex Oliv.) Maxim. 산들깨 | VU | 1 |
| Lamiaceae | *Nepeta cataria* L. 개박하 | VU | 3 |
| Scrophulariaceae | *Mimulus tenellus* Bunge 애기물꽈리아재비 | VU | 2 |
| Lentibulariaceae | *Utricularia bifida* L. 땅귀개 | VU | 13 |
| Lentibulariaceae | *Utricularia japonica* Makino 통발 | VU | 5 |
| Caprifoliaceae | *Lonicera caerulea* L. 댕댕이나무 | VU | 1 |
| Campanulaceae | *Hanabusaya asiatica* (Nakai) Nakai 금강초롱꽃 | VU | 1 |
| Asteraceae | *Aster fastigiatus* Fisch. 옹굿나물 | VU | 2 |
| Asteraceae | *Inula salicina* L. 버들금불초 | VU | 2 |
| Asteraceae | *Prenanthes ochroleuca* (Maxim.) Hemsl. 왕씀배 | VU | 1 |
| Asteraceae | *Scorzonera albicaulis* Bunge 쇠채 | VU | 1 |
| Asteraceae | *Senecio argunensis* Turcz. 쑥방망이 | VU | 2 |
| Liliaceae | *Trillium tschonoskii* Maxim. 큰연영초 | VU | 1 |
| Iridaceae | *Iris minutoaurea* Makino 금붓꽃 | VU | 2 |
| Typhaceae | *Sparganium stoloniferum* (Graebn.) Buch.- Ham. ex Juz. 흑삼릉 | VU | 2 |
| Orchidaceae | *Cephalanthera erecta* (Thunb.) Blume f. *subaphylla* (Miyabe & Kudô) Hiroë 꼬마은난초 | VU | 2 |
| Orchidaceae | *Galearis cyclochila* (Franch. & Sav.) Soó 나도제비란 | VU | 5 |
| Orchidaceae | *Pogonia japonica* Rchb.f. 큰방울새란 | VU | 11 |
| Orchidaceae | *Pogonia minor* (Makino) Makino 방울새란 | VU | 4 |
| Ophioglossaceae | *Botrychium virginianum* (L.) Sw. 늦고사리삼 | LC | 1 |
| Pinaceae | *Abies koreana* E. H. Wilson 구상나무 | LC | 2 |
| Ulmaceae | *Celtis choseniana* Nakai 검팽나무 | LC | 2 |
| Lauraceae | *Lindera sericea* (Siebold &Zucc.) Blume 털조장나무 | LC | 1 |
| Aristolochiaceae | *Aristolochia contorta* Bunge 쥐방울덩굴 | LC | 23 |
| Aristolochiaceae | *Aristolochia manshuriensis* Kom. 등칡 | LC | 6 |
| Papaveraceae | *Coreanomecon hylomeconoides* Nakai 매미꽃 | LC | 1 |
| Penthoraceae | *Penthorum chinense* Pursh 낙지다리 | LC | 8 |
| Saxifragaceae | *Rodgersia podophylla* A. Gray 도깨비부채 | LC | 1 |
| Violaceae | *Viola albida* Palib. 태백제비꽃 | LC | 10 |
| Violaceae | *Viola diamantiaca* Nakai 금강제비꽃 | LC | 3 |
| Cucurbitaceae | *Melothria japonica* (Thunb.) Maxim. ex Cogn. 새박 | LC | 4 |
| Primulaceae | *Lysimachia coreana* Nakai 참좁쌀풀 | LC | 8 |
| Oleaceae | *Chionanthus retusus* Lindl. & Paxton 이팝나무 | LC | 1 |
| Oleaceae | *Syringa villosa* Vahl subsp. *wolfii* (C. K. Schneid.) Y. Chen & D. Y. Hong 꽃개회나무 | LC | 5 |
| Gentianaceae | *Gentiana triflora* Pall. var. *japonica* (Kusn.) H. Hara 과남풀 | LC | 18 |
| Boraginaceae | *Trigonotis icumae* (Maxim.) Makino 덩굴꽃마리 | LC | 10 |
| Lamiaceae | *Scutellaria insignis* Nakai 광릉골무꽃 | LC | 4 |
| Lentibulariaceae | *Utricularia caerulea* L. 이삭귀개 | LC | 16 |
| Valerianaceae | *Patrinia saniculifolia* Hemsl. 금마타리 | LC | 1 |
| Asteraceae | *Parasenecio auriculatus* (DC.) J.R. Grant 귀박쥐나물 | LC | 1 |
| Hydrocharitaceae | *Ottelia alismoides* (L.) Pers. 물질경이 | LC | 3 |
| Liliaceae | *Lilium distichum* Nakai ex Kamib. 말나리 | LC | 5 |
| Liliaceae | *Streptopus ovalis* (Ohwi) F. T. Wang & Y. C. Tang 금강애기나리 | LC | 3 |
| Liliaceae | *Tricyrtis macropoda* Miq. 뻐꾹나리 | LC | 13 |
| Liliaceae | *Trillium camschatcense* Ker Gawl. 연영초 | LC | 4 |
| Iridaceae | *Iris ensata* Thunb. var. *spontanea* (Makino) Nakai꽃창포 | LC | 67 |
| Poaceae | *Phacelurus latifolius* (Steud.) Ohwi 모새달 | LC | 1 |
| Araceae | *Acorus calamus* L. 창포 | LC | 12 |
| Araceae | *Arisaema heterophyllum* Blume 두루미천남성 | LC | 11 |
| Orchidaceae | *Goodyera schlechtendaliana* Rchb.f. 사철란 | LC | 1 |
| Polygonaceae | *Rumex longifolius* DC. 개대황 | DD | 1 |
| Chloranthaceae | *Chloranthus fortunei* (A. Gray) Solms 옥녀꽃대 | DD | 3 |
| Clusiaceae | *Hypericum attenuatum* Fisch. ex Choisy 채고추나물 | DD | 5 |
| Onagraceae | *Epilobium palustre* L. 버들바늘꽃 | DD | 2 |
| Alangiaceae | *Alangium platanifolium* (Siebold & Zucc.) Harms 단풍박쥐나무 | DD | 1 |
| Araliaceae | *Eleutherococcus divaricatus* var. chiisanensis (Nakai) C. H. Kim & B. Y. Sun 지리산오갈피 | DD | 8 |
| Scrophulariaceae | *Scrophularia koraiensis* Nakai 토현삼 | DD | 10 |
| Alismataceae | *Caldesia parnassifolia* (Bassi ex L.) Parl. 둥근잎택사 | DD | 2 |
| Alismataceae | *Sagittaria trifolia* L. 벗풀 | DD | 13 |
| Liliaceae | *Polygonatum infundiflorum* Y. S. Kim, B. U. Oh & C. G. Jang 늦둥굴레 | DD | 7 |
| Typhaceae | *Sparganium japonicum* Rothert 긴흑삼릉 | DD | 1 |
| Cyperaceae | *Carex idzuroei* Franch. & Sav. 좀도깨비사초 | DD | 3 |
| Cyperaceae | *Carex pseudochinensis* H. Lév. & Vaniot 햇사초 | DD | 1 |
| Cyperaceae | *Scirpus orientalis* Ohwi 검은도루박이 | DD | 1 |
