# Supplementary material for: Floristic inventory and distribution characteristics of vascular plants in forest wetlands of South Korea
Source: Biodivers Data J. 2022 Sep 15;10:e85848. doi: 10.3897/BDJ.10.e85848 (PMC9848468; doi:10.3897/BDJ.10.e85848)
Supplement: Supplementary material 5 — The list of endangered wild plants in forest wetlands of Korea. [file bdj-10-e85848-s005.docx]

Table 5. The list of endangered wild plants in forest wetlands of Korea.

| **Family name** | **Scientific name / Korean name** | **Region** | **Site (Frequency)** |
| --- | --- | --- | --- |
| Ophioglossaceae | *Mankyua chejuense* B. Y. Sun, M. H. Kim & C. H. Kim  제주고사리삼 | Jeju | Jeju-si (7) |
| Apiaceae | *Cicuta virosa* L.  독미나리 | Gangwon | Yangyang-gun (2) |
|  |  |  | Taebaek-si (2) |
|  |  |  | Pyeongchang-gun (1) |
| Lentibulariaceae | *Utricularia uliginosa* Vahl  자주땅귀개 | Busan | Gijang-gun (1) |
|  |  | Ulsan | Buk-gu (1) |
|  |  | Jeonnam | Goheung-gun (2) |
|  |  | Jeju | Jeju-si (2) |
| Orchidaceae | *Habenaria radiata* (Thunb.) Spreng.  해오라비난초 | Jeonnam | Goheung-gun (1) |
| Primulaceae | *Trientalis europaea* L. var. *arctica* (Fisch. ex Hook.) Ledeb.  기생꽃 | Gangwon | Pyeongchang-gun (1) |
| Menyanthaceae | *Menyanthes trifoliata* L.  조름나물 | Gangwon | Taebaek-si (2) |
| Cabombaceae | *Brasenia schreberi* J. F. Gmel.  순채 | Gangwon | Samcheok-si (1) |
|  |  | Jeju | Jeju-si (7) |
| Araliaceae | *Eleutherococcus senticosus* (Rupr. & Maxim.) Maxim.  가시오갈피 | Gangwon | Taebaek-si (1) |
|  |  | Gyeongnam | Goseong-gun (1) |
