# Supplementary material for: Floristic inventory and distribution characteristics of vascular plants in forest wetlands of South Korea
Source: Biodivers Data J. 2022 Sep 15;10:e85848. doi: 10.3897/BDJ.10.e85848 (PMC9848468; doi:10.3897/BDJ.10.e85848)
Supplement: Supplementary material 7 — Floristic target plants of grade IV plants identified in the survey. [file bdj-10-e85848-s007.docx]

Table 9. Limestone area plants identified in the survey.

| Family name | Scientific name / Korean name | Frequency |
| --- | --- | --- |
| Aspleniaceae | *Asplenium ruprechtii* Sa. Kurata 거미고사리 | 1 |
| Cupressaceae | *Juniperus rigida* Siebold & Zucc. 노간주나무 | 22 |
| Betulaceae | *Carpinus turczaninovii* Hance 소사나무 | 1 |
| Fagaceae | *Quercus variabilis* Blume 굴참나무 | 51 |
| Ulmaceae | *Ulmus macrocarpa* Hance 왕느릅나무 | 1 |
| Moraceae | *Morus cathayana* Hemsl. 돌뽕나무 | 1 |
| Ranunculaceae | *Clematis brachyura* Maxim. 외대으아리 | 1 |
| Brassicaceae | *Berteroella maximowiczii* (Palib.) O. E. Schulz 장대냉이 | 1 |
| Brassicaceae | *Sisymbrium luteum* (Maxim.) O. E. Schulz 노란장대 | 2 |
| Rosaceae | *Spiraea blumei* G. Don 산조팝나무 | 5 |
| Rosaceae | *Spiraea trichocarpa* Nakai 갈기조팝나무 | 1 |
| Euphorbiaceae | *Euphorbia sieboldiana* Morren & Decne. 개감수 | 2 |
| Celastraceae | *Euonymus alatus* (Thunb.) Siebold 화살나무 | 16 |
| Celastraceae | *Euonymus pauciflorus* Maxim. 회목나무 | 1 |
| Gentianaceae | *Swertia pseudochinensis* H. Hara 자주쓴풀 | 2 |
| Lamiaceae | *Isodon japonicus* (Burm.f.) H. Hara 방아풀 | 11 |
| Scrophulariaceae | *Siphonostegia chinensis* Benth. 절국대 | 1 |
| Caprifoliaceae | *Lonicera subsessilis* Rehder 청괴불나무 | 4 |
| Caprifoliaceae | *Viburnum carlesii* Hemsl. 분꽃나무 | 3 |
| Caprifoliaceae | *Weigela praecox* (Lemoine) L. H. Bailey 소영도리나무 | 9 |
| Ranunculaceae | *Actaea bifida* (Nakai) J. Compton 세잎승마 | 4 |
| Asteraceae | *Artemisia sacrorum* Ledeb. var. *iwayomogi* (Kitam.) M. S. Park & G. Y. Chung 더위지기 | 8 |
| Asteraceae | *Aster maackii* Regel 좀개미취 | 1 |
| Asteraceae | *Dendranthema oreastrum* (Hance) Y. Ling 바위구절초 | 1 |
| Ericaceae | *Rhododendron micranthum* Turcz. 꼬리진달래 | 3 |
| Asteraceae | *Stemmacantha uniflora* (L.) Dittrich 뻐꾹채 | 1 |
| Liliaceae | *Asparagus oligoclonos* Maxim. 방울비짜루 | 1 |
| Liliaceae | *Smilax sieboldii* Miq. 청가시덩굴 | 124 |
| Liliaceae | *Veratrum nigrum* L. var. *ussuriense* O. Loes. 참여로 | 1 |
| Cyperaceae | *Scirpus orientalis* Ohwi 검은도루박이 | 1 |
| Orchidaceae | *Epipactis thunbergii* A. Gray 닭의난초 | 12 |
