# Supplementary material for: Floristic inventory and distribution characteristics of vascular plants in forest wetlands of South Korea
Source: Biodivers Data J. 2022 Sep 15;10:e85848. doi: 10.3897/BDJ.10.e85848 (PMC9848468; doi:10.3897/BDJ.10.e85848)
Supplement: Supplementary material 8 — Floristic target plants of grade III identified in the survey. [file bdj-10-e85848-s008.docx]

Table 8. Floristic target plants of grade III identified in the survey.

| Family name | Scientific name / Korean name | IUCN category | Frequency |
| --- | --- | --- | --- |
| Selaginellaceae | *Selaginella tamariscina* (P. Beauv.) Spring 바위손 |  | 1 |
| Equisetaceae | *Equisetum hyemale* L. 속새 |  | 8 |
| Osmundaceae | *Osmunda claytoniana* L. 음양고비 |  | 4 |
| Gleicheniaceae | *Dicranopteris linearis* (Burm. f.) Underw. 발풀고사리 |  | 1 |
| Pteridaceae | *Coniogramme japonica* (Thunb.) Diels 가지고비고사리 |  | 1 |
| Thelypteridaceae | *Metathelypteris laxa* (Franch. & Sav.) Ching 드문고사리 |  | 1 |
| Thelypteridaceae | *Parathelypteris beddomei* (Baker) Ching 가는잎처녀고사리 |  | 1 |
| Salicaceae | *Salix rorida* Laksch. 분버들 |  | 8 |
| Salicaceae | *Salix xerophila* Flod. 여우버들 |  | 1 |
| Betulaceae | *Betula costata* Trautv. 거제수나무 |  | 7 |
| Betulaceae | *Betula davurica* Pall. 물박달나무 |  | 29 |
| Betulaceae | *Betula schmidtii* Regel 박달나무 |  | 7 |
| Fagaceae | *Quercus glauca* Thunb. 종가시나무 |  | 2 |
| Ulmaceae | *Ulmus laciniata* (Trautv.) Mayr 난티나무 |  | 2 |
| Moraceae | *Morus cathayana* Hemsl. 돌뽕나무 |  | 1 |
| Urticaceae | *Boehmeria pannosa* Nakai & Satake ex Oka 왕모시풀 |  | 1 |
| Urticaceae | *Urtica angustifolia* Fisch. ex Hornem. 가는잎쐐기풀 |  | 1 |
| Urticaceae | *Urtica thunbergiana* Siebold & Zucc. 쐐기풀 |  | 5 |
| Polygonaceae | *Persicaria erectominor* (Makino) Nakai var. *koreensis* (Nakai) I. Ito 대동여뀌 |  | 2 |
| Ophioglossaceae | *Ophioglossum vulgatum* L. 나도고사리삼 | EN | 2 |
| Lauraceae | *Litsea japonica* (Thunb.) Juss. 까마귀쪽나무 |  | 1 |
| Ranunculaceae | *Actaea asiatica* H. Hara 노루삼 |  | 9 |
| Ranunculaceae | *Megaleranthis saniculifolia* Ohwi 모데미풀 | EN | 1 |
| Ranunculaceae | *Aquilegia buergeriana* var. *oxysepala* (Trautv. & A. Mey.) Kitam. 매발톱 |  | 2 |
| Ranunculaceae | *Clematis brachyura* Maxim. 외대으아리 |  | 1 |
| Ranunculaceae | *Clematis fusca* Turcz. var. *flabellata* (Nakai) J. S. Kim 요강나물 |  | 2 |
| Ranunculaceae | *Clematis fusca* Turcz. var. *violacea* Maxim. 종덩굴 |  | 3 |
| Ranunculaceae | *Clematis urticifolia* Nakai ex Kitag. 병조희풀 |  | 3 |
| Ranunculaceae | *Thalictrum rochebrunnianum* Franch. & Sav. 금꿩의다리 |  | 7 |
| Menispermaceae | *Sinomenium acutum* (Thunb.) Rehder & E. H. Wilson 방기 |  | 1 |
| Apocynaceae | *Cynanchum amplexicaule* (Siebold & Zucc.) Hemsl. 솜아마존 | EN | 5 |
| Boraginaceae | *Trigonotis radicans* (Turcz.) Steven 거센털꽃마리 | EN | 1 |
| Actinidiaceae | *Actinidia kolomikta* (Maxim. & Rupr.) Maxim. 쥐다래 |  | 7 |
| Theaceae | *Stewartia koreana* Nakai ex Rehder 노각나무 |  | 3 |
| Clusiaceae | *Triadenum japonicum* (Blume) Makino 물고추나물 |  | 10 |
| Asteraceae | *Cirsium rhinoceros* (H. Lév. & Vaniot) Nakai 바늘엉겅퀴 | EN | 4 |
| Papaveraceae | *Dicentra spectabilis* (L.) Lem. 금낭화 |  | 2 |
| Brassicaceae | *Cardamine komarovii* Nakai 는쟁이냉이 |  | 9 |
| Brassicaceae | *Catolobus pendulus* (L.) Al-Shehbaz 느러진장대 |  | 1 |
| Pinaceae | *Abies koreana* E. H. Wilson 구상나무 | LC | 2 |
| Hydrangeaceae | *Hydrangea petiolaris* Siebold & Zucc. 등수국 |  | 1 |
| Hydrangeaceae | *Philadelphus schrenkii* Rupr. 고광나무 |  | 15 |
| Grossulariaceae | *Ribes mandshuricum* (Maxim.) Kom. 까치밥나무 |  | 3 |
| Rosaceae | *Aruncus dioicus* (Walter) Fernald 눈개승마 |  | 4 |
| Rosaceae | *Potentilla centigrana* Maxim. 좀딸기 |  | 4 |
| Rosaceae | *Potentilla cryptotaeniae* Maxim. 물양지꽃 |  | 15 |
| Rosaceae | *Prunus maackii* Rupr. 개벚지나무 |  | 1 |
| Rosaceae | *Prunus mandshurica* (Maxim.) Koehne 개살구나무 |  | 1 |
| Rosaceae | *Prunus sargentii* Rehder 산벚나무 |  | 51 |
| Rosaceae | *Rubus buergeri* Miq. 겨울딸기 |  | 1 |
| Rosaceae | *Sorbaria sorbifolia* (L.) A. Braun var. *stellipila* Maxim. 쉬땅나무 |  | 6 |
| Rosaceae | *Spiraea fritschiana* C. K. Schneid. 참조팝나무 |  | 22 |
| Rosaceae | *Spiraea microgyna* Nakai 좀조팝나무 |  | 1 |
| Fabaceae | *Indigofera pseudotinctoria* Matsum. 낭아초 |  | 2 |
| Fabaceae | *Lespedeza davurica* (Laxm.) Schindl. 호비수리 |  | 1 |
| Fabaceae | *Vicia chosenensis* Ohwi 노랑갈퀴 |  | 2 |
| Oxalidaceae | *Oxalis acetosella* L. 애기괭이밥 |  | 1 |
| Daphniphyllaceae | *Daphniphyllum macropodum* Miq. 굴거리나무 |  | 2 |
| Aceraceae | *Acer barbinerve* Maxim. 청시닥나무 |  | 10 |
| Aceraceae | *Acer komarovii* Pojark. 시닥나무 |  | 10 |
| Aceraceae | *Acer mandshuricum* Maxim. 복장나무 |  | 7 |
| Aceraceae | *Acer palmatum* Thunb. 단풍나무 |  | 10 |
| Aceraceae | *Acer triflorum* Kom. 복자기 |  | 9 |
| Aceraceae | *Acer ukurunduense* Trautv. & C. A. Mey. 부게꽃나무 |  | 1 |
| Aquifoliaceae | *Ilex crenata* Thunb. 꽝꽝나무 |  | 8 |
| Vitaceae | *Vitis coignetiae* Pulliat ex Planch. 머루 |  | 28 |
| Elaeagnaceae | *Elaeagnus glabra* Thunb. 보리장나무 |  | 3 |
| Onagraceae | *Ludwigia ovalis* Miq. 눈여뀌바늘 |  | 6 |
| Araliaceae | *Dendropanax trifidus* (Thunb.) Makino ex H. Hara 황칠나무 |  | 1 |
| Araliaceae | *Eleutherococcus divaricatus* var. *chiisanensis* (Nakai) C. .H. . Kim & B. Y. . Sun 지리산오갈피 | DD | 8 |
| Apiaceae | *Centella asiatica* (L.) Urb. 병풀 |  | 7 |
| Primulaceae | *Lysimachia vulgaris* L. var. *davurica* (Ledeb.) R. Knuth 좁쌀풀 |  | 59 |
| Violaceae | *Viola diamantiaca* Nakai 금강제비꽃 | LC | 3 |
| Oleaceae | *Fraxinus chiisanensis* Nakai 물들메나무 |  | 11 |
| Oleaceae | *Ligustrum ovalifolium* Hassk. 왕쥐똥나무 |  | 3 |
| Oleaceae | *Syringa reticulata* (Blume) H. Hara 개회나무 |  | 10 |
| Oleaceae | *Chionanthus retusus* Lindl. & Paxton 이팝나무 | LC | 1 |
| Lamiaceae | *Scutellaria insignis* Nakai 광릉골무꽃 | LC | 4 |
| Apocynaceae | *Cynanchum nipponicum* Matsum. 덩굴박주가리 |  | 21 |
| Apocynaceae | *Cynanchum nipponicum* Matsum. var. *glabrum* (Nakai) H. Hara 흑박주가리 |  | 1 |
| Valerianaceae | *Patrinia saniculifolia* Hemsl. 금마타리 | LC | 1 |
| Rubiaceae | *Galium odoratum* (L.) Scop. 선갈퀴 |  | 6 |
| Rubiaceae | *Neanotis hirsuta* (L.f.) W. H. Lewis 탐라풀 |  | 1 |
| Boraginaceae | *Brachybotrys paridiformis* Maxim. ex Oliv. 당개지치 |  | 13 |
| Verbenaceae | *Callicarpa dichotoma* (Lour.) Raeusch. ex K. Koch 좀작살나무 |  | 1 |
| Verbenaceae | *Callicarpa mollis* Siebold & Zucc. 새비나무 |  | 1 |
| Liliaceae | *Lilium distichum* Nakai ex Kamib. 말나리 | LC | 5 |
| Lamiaceae | *Salvia japonica* Thunb. 둥근배암차즈기 |  | 3 |
| Lamiaceae | *Teucrium veronicoides* Maxim. 곽향 |  | 1 |
| Scrophulariaceae | *Euphrasia maximowiczii* Wettst. ex Palib. 앉은좁쌀풀 |  | 1 |
| Caprifoliaceae | *Viburnum furcatum* Blume ex Maxim. 분단나무 |  | 1 |
| Asteraceae | *Aster maackii* Regel 좀개미취 |  | 1 |
| Asteraceae | *Cirsium schantarense* Trautv. & C. A. Mey. 도깨비엉겅퀴 |  | 2 |
| Droseraceae | *Drosera rotundifolia* L. 끈끈이주걱 | VU | 14 |
| Asteraceae | *Parasenecio auriculatus* (DC.) J. R. Grant var. *kamtschatica* (Maxim.) H. Koyama 나래박쥐나물 |  | 3 |
| Asteraceae | *Parasenecio hastatus* (L.) H. Koyama var. *orientalis* (Kitam.) H. Koyama 민박쥐나물 |  | 3 |
| Liliaceae | *Hosta clausa* Nakai 주걱비비추 |  | 1 |
| Liliaceae | *Polygonatum falcatum* A. Gray 진황정 |  | 1 |
| Liliaceae | *Veratrum maackii* Regel 긴잎여로 |  | 8 |
| Liliaceae | *Veratrum maackii* Regel var. *japonicum* (Baker) Shimizu 여로 |  | 13 |
| Liliaceae | *Veratrum nigrum* L. var. *ussuriense* O. Loes. 참여로 |  | 1 |
| Poaceae | *Agrostis scabra* Willd. 긴겨이삭 |  | 3 |
| Poaceae | *Lophatherum gracile* Brongn. 조릿대풀 |  | 2 |
| Araceae | *Acorus gramineus* Aiton 석창포 |  | 1 |
| Araceae | *Symplocarpus nipponicus* Makino 애기앉은부채 |  | 4 |
| Cyperaceae | *Carex lasiolepis* Franch. 난사초 |  | 1 |
| Cyperaceae | *Carex pilosa* Scop. 털사초 |  | 4 |
| Cyperaceae | *Carex tegulata* H. Lév. & Vaniot 구슬사초 |  | 1 |
| Orchidaceae | *Cephalanthera falcata* (Thunb.) Blume 금난초 |  | 2 |
| Typhaceae | *Sparganium stoloniferum* (Graebn.) Buch.- Ham. ex Juz. 흑삼릉 | VU | 2 |
