# Supplementary material for: Floristic inventory and distribution characteristics of vascular plants in forest wetlands of South Korea
Source: Biodivers Data J. 2022 Sep 15;10:e85848. doi: 10.3897/BDJ.10.e85848 (PMC9848468; doi:10.3897/BDJ.10.e85848)
Supplement: Supplementary material 10 — Invasive alien plants identified in the survey. [file bdj-10-e85848-s010.docx]

Table 10. Invasive alien plants identified in the survey.

| **Family name** | **Scientific name / Korean name** | **Grade** | **Frequency** |
| --- | --- | --- | --- |
| Polygonaceae | *Persicaria orientalis* (L.) Spach 털여뀌 | SR | 2 |
| Polygonaceae | *Rumex acetosella* L. 애기수영 | WS | 7 |
| Polygonaceae | *Rumex crispus* L. 소리쟁이 | WS | 9 |
| Polygonaceae | *Rumex obtusifolius* L. 돌소리쟁이 | SC | 2 |
| Phytolaccaceae | *Phytolacca americana* L. 미국자리공 | WS | 61 |
| Caryophyllaceae | *Cerastium glomeratum* Thuill. 유럽점나도나물 | SC | 2 |
| Chenopodiaceae | *Chenopodium album* L. 흰명아주 | SR | 6 |
| Chenopodiaceae | *Chenopodium ficifolium* Sm. 좀명아주 | WS | 5 |
| Amaranthaceae | *Amaranthus blitum* L. subsp. *oleraceus* (L.) Costea 개비름 | SR | 2 |
| Brassicaceae | *Barbarea vulgaris* R.Br. 유럽나도냉이 | SC | 7 |
| Brassicaceae | *Lepidium virginicum* L. 콩다닥냉이 | SS | 1 |
| Fabaceae | *Amorpha fruticosa* L. 족제비싸리 | WS | 40 |
| Fabaceae | *Medicago polymorpha* L. 개자리 | SC | 1 |
| Fabaceae | *Robinia pseudoacacia* L. 아까시나무 | WS | 111 |
| Fabaceae | *Trifolium pratense* L. 붉은토끼풀 | WS | 3 |
| Fabaceae | *Trifolium repens* L. 토끼풀 | WS | 28 |
| Fabaceae | *Vicia villosa* Roth 벳지 | SC | 2 |
| Simaroubaceae | *Ailanthus altissima* (Mill.) Swingle 가죽나무 | SR | 22 |
| Malvaceae | *Hibiscus trionum* L. 수박풀 | SC | 1 |
| Cucurbitaceae | *Sicyos angulatus* L. 가시박 | SC | 1 |
| Onagraceae | *Oenothera biennis* L. 달맞이꽃 | WS | 37 |
| Convolvulaceae | *Cuscuta pentagona* Engelm. 미국실새삼 | SR | 2 |
| Convolvulaceae | *Quamoclit angulata* (Lam.) Bojer 둥근잎유홍초 | SR | 1 |
| Boraginaceae | *Symphytum officinale* L. 컴프리 | SC | 1 |
| Solanaceae | *Solanum carolinense* L. 도깨비가지 | SC | 1 |
| Scrophulariaceae | *Veronica arvensis* L. 선개불알풀 | WS | 3 |
| Asteraceae | *Achillea millefolium* L. 서양톱풀 | SC | 1 |
| Asteraceae | *Ageratina altissima* (L.) R. M. King & H.Rob. 서양등골나물 | CS | 5 |
| Asteraceae | *Ambrosia artemisiifolia* L. 돼지풀 | WS | 25 |
| Asteraceae | *Ambrosia trifida* L. 단풍잎돼지풀 | SC | 3 |
| Asteraceae | *Bidens frondosa* L. 미국가막사리 | WS | 153 |
| Asteraceae | *Bidens pilosa* L. 울산도깨비바늘 | SC | 2 |
| Asteraceae | *Carduus crispus* L. 지느러미엉겅퀴 | SS | 8 |
| Asteraceae | *Conyza bonariensis* (L.) Cronquist 실망초 | SS | 1 |
| Asteraceae | *Conyza canadensis* (L.) Cronquist 망초 | WS | 54 |
| Asteraceae | *Coreopsis lanceolata* L. 큰금계국 | SR | 3 |
| Asteraceae | *Cosmos bipinnatus* Cav. 코스모스 | SR | 1 |
| Asteraceae | *Crassocephalum crepidioides* (Benth.) S. Moore 주홍서나물 | SR | 5 |
| Asteraceae | *Erechtites hieraciifolius* (L.) Raf. ex DC.붉은서나물 | WS | 33 |
| Asteraceae | *Erigeron annuus* (L.) Pers. 개망초 | WS | 202 |
| Asteraceae | *Erigeron floribundus* (Kunth) Sch. Bip. 큰망초 | CS | 1 |
| Asteraceae | *Erigeron philadelphicus* L. 봄망초 | CS | 2 |
| Asteraceae | *Erigeron strigosus* Muhl. ex Willd. 주걱개망초 | SC | 9 |
| Asteraceae | *Galinsoga quadriradiata* Ruiz & Pav. 털별꽃아재비 | WS | 3 |
| Asteraceae | *Helianthus tuberosus* L. 뚱딴지 | SC | 6 |
| Asteraceae | *Rudbeckia bicolor* Nutt. 원추천인국 | SR | 1 |
| Asteraceae | *Solidago gigantea* Aiton 미국미역취 | SR | 1 |
| Asteraceae | *Symphyotrichum pilosum* (Willd.) G. L. Nesom 미국쑥부쟁이 | SS | 36 |
| Asteraceae | *Tagetes minuta* L. 만수국아재비 | SC | 1 |
| Asteraceae | *Taraxacum officinale* F. H. Wigg. 서양민들레 | WS | 11 |
| Asteraceae | *Xanthium strumarium* L. 도꼬마리 | SS | 1 |
| Iridaceae | *Sisyrinchium rosulatum* E. P. Bicknell 등심붓꽃 | CS | 1 |
| Poaceae | *Avena fatua* L. 메귀리 | SC | 1 |
| Poaceae | *Briza minor* L. 방울새풀 | CS | 4 |
| Poaceae | *Bromus tectorum* L. 털빕새귀리 | SC | 2 |
| Poaceae | *Dactylis glomerata* L. 오리새 | WS | 20 |
| Poaceae | *Elymus repens* (L.) Gould 구주개밀 | CS | 1 |
| Poaceae | *Festuca arundinacea* Schreb. 큰김의털 | SS | 12 |
| Poaceae | *Lolium multiflorum* Lam. 쥐보리 | SR | 1 |
| Poaceae | *Lolium perenne* L. 호밀풀 | SS | 1 |
| Poaceae | *Panicum dichotomiflorum* Michx. 미국개기장 | SS | 9 |
| Poaceae | *Phleum pratense* L. 큰조아재비 | SR | 7 |
| Poaceae | *Poa pratensis* L. 왕포아풀 | SS | 8 |
| Poaceae | *Vulpia myuros* (L.) C. C. Gmel. 들묵새 | SC | 1 |
