# Supplementary material for: Floristic inventory and distribution characteristics of vascular plants in forest wetlands of South Korea
Source: Biodivers Data J. 2022 Sep 15;10:e85848. doi: 10.3897/BDJ.10.e85848 (PMC9848468; doi:10.3897/BDJ.10.e85848)
Supplement: Supplementary material 11 — Categorizing vascular plant species occurring in wetland ecosystems of the Korean Peninsula by frequency of occurrence in the study area. [file bdj-10-e85848-s011.docx]

Table 11. Categorizing vascular plant species occurring in wetland ecosystems of the Korean Peninsula by frequency of occurrence in study area

| Frequency of occurrence | Abbreviation | Explanation | Frequency (% of recorded taxa) | |
| --- | --- | --- | --- | --- |
| Obligate wetland plant | OBW | Occurs almost always in wetlands under natural conditions (estimated ＞ 98% probability in wetlands) | | 152  (11.0) |
| Facultative wetland plant | FACW | Usually occurs in wetlands  but occasionally found in non-wetlands  (estimated ∼71–98% probability in wetlands) | | 138  (9.9) |
| Facultative plant | FAC | Equally likely to occur in wetlands or non-wetlands  (estimated ∼31–70% probability in wetlands) | | 177  (12.7) |
| Facultative upland plant | FACU | Occasionally occurs in wetlands,  but usually occurs in non-wetlands  (estimated ∼3–30% probability in wetlands) | | 198  (14.3) |
| Obligate upland plant | OBU | Almost never occurs in wetlands under natural conditions  (estimated ＜ 3% probability in wetlands) | | 723  (52.1) |
