# Supplementary material for: Floristic inventory and distribution characteristics of vascular plants in forest wetlands of South Korea
Source: Biodivers Data J. 2022 Sep 15;10:e85848. doi: 10.3897/BDJ.10.e85848 (PMC9848468; doi:10.3897/BDJ.10.e85848)
Supplement: Supplementary material 12 — Vascular plants recorded only in forest wetlands of Gangwon region, Korea. [file bdj-10-e85848-s012.docx]

Table 12. Vascular plants recorded only in forest wetlands of Gangwon region, Korea.

| Family name | Scientific name / Korean name | Fre. | RP. |
| --- | --- | --- | --- |
| Equisetaceae | *Equisetum ramosissimum* Desf. 개속새 | 1 | Ⅱ |
| Ophioglossaceae | *Botrychium virginianum* (L.) Sw. 늦고사리삼 | 1 | LC, Ⅱ |
| Athyriaceae | *Cornopteris crenulato-serrulata* (Makino) Nakai 응달고사리 | 6 |  |
| Dryopteridaceae | *Arachniodes borealis* Seriz. 왁살고사리 | 3 |  |
| Dryopteridaceae | *Dryopteris erythrosora* (D. C. Eaton) Kuntze 홍지네고사리 | 1 | Ⅰ |
| Taxaceae | *Taxus cuspidata* Siebold & Zucc. 주목 | 1 | VU, Ⅱ |
| Salicaceae | *Populus suaveolens* Fisch. Ex Poit. & A. Vilm. 황철나무 | 1 | Ⅳ |
| Salicaceae | *Salix babylonica* L. 수양버들 | 2 |  |
| Salicaceae | *Salix rorida* Laksch. 분버들 | 8 | Ⅲ |
| Salicaceae | *Salix siuzevii* Seem. 참오글잎버들 | 3 |  |
| Salicaceae | *Salix xerophila* Flod. 여우버들 | 1 | Ⅲ |
| Betulaceae | *Carpinus cordata* Blume 까치박달 | 19 |  |
| Betulaceae | *Carpinus turczaninovii* Hance 소사나무 | 1 | Ⅰ |
| Ulmaceae | *Ulmus laciniata* (Trautv.) Mayr 난티나무 | 2 | Ⅲ |
| Ulmaceae | *Ulmus macrocarpa* Hance 왕느릅나무 | 1 | Ⅳ |
| Urticaceae | *Urtica angustifolia* Fisch. Ex Hornem. 가는잎쐐기풀 | 1 | Ⅲ |
| Polygonaceae | *Aconogonon divaricatum* (L.) Nakai ex T. Mori 왜개싱아 | 1 |  |
| Polygonaceae | *Aconogonon microcarpum* (Kitag.) H. Hara 참개싱아 | 1 | ED, Ⅳ |
| Polygonaceae | *Bistorta incana* (Nakai) Nakai ex T. Mori 흰범꼬리 | 1 |  |
| Polygonaceae | *Reynoutria ciliinervis* (Nakai) Moldenke 나도하수오 | 1 |  |
| Portulacaceae | *Portulaca oleracea* L. 쇠비름 | 1 |  |
| Caryophyllaceae | *Cerastium glomeratum* Thuill. 유럽점나도나물 | 2 | SC |
| Magnoliaceae | *Magnolia denudata* Desr. 백목련 | 1 |  |
| Ranunculaceae | *Aquilegia buergeriana* var. *oxysepala* (Trautv. &. A. Mey.) Kitam. 매발톱 | 2 | Ⅲ |
| Ranunculaceae | *Clematis fusca* Turcz. var. *flabellata* (Nakai) J.S. Kim 요강나물 | 2 | ED, Ⅲ |
| Ranunculaceae | *Clematis fusca* Turcz. var. *violacea* Maxim. 종덩굴 | 3 | Ⅲ |
| Ranunculaceae | *Megaleranthis saniculifolia* Ohwi 모데미풀 | 1 | EN, ED, Ⅲ |
| Menispermaceae | *Sinomenium acutum* (Thunb.) Rehder & E. H. Wilson 방기 | 1 | Ⅲ |
| Aristolochiaceae | *Aristolochia manshuriensis* Kom. 등칡 | 6 | LC, Ⅱ |
| Paeoniaceae | *Paeonia japonica* (Makino) Miyabe & Takeda 백작약 | 1 | VU, Ⅱ |
| Papaveraceae | *Dicentra spectabilis* (L.) Lem. 금낭화 | 2 | Ⅲ |
| Brassicaceae | *Barbarea thunbergia* Ledeb. 나도냉이 | 1 |  |
| Brassicaceae | *Capsella bursa-pastoris* (L.) Medik. 냉이 | 5 |  |
| Brassicaceae | *Cardamine fallax* (O. E. Schulz) Nakai 좁쌀냉이 | 2 |  |
| Brassicaceae | *Cardamine scutata* Thunb. 큰황새냉이 | 3 |  |
| Brassicaceae | *Sisymbrium luteum* (Maxim.) O. E. Schulz 노란장대 | 2 | Ⅰ |
| Crassulaceae | *Hylotelephium erythrostictum* (Miq.) H. Ohba 꿩의비름 | 1 |  |
| Saxifragaceae | *Chrysosplenium pilosum* Maxim. 털괭이눈 | 1 | Ⅱ |
| Saxifragaceae | *Chrysosplenium pseudofauriei* H. Lév. 선괭이눈 | 2 | Ⅰ |
| Saxifragaceae | *Chrysosplenium ramosum* Maxim. 가지괭이눈 | 1 | Ⅳ |
| Saxifragaceae | *Micranthes octopetala* (Nakai) Y. I. Kim & Y. D. Kim 구실바위취 | 5 | EN, ED, Ⅳ |
| Saxifragaceae | *Saxifraga fortunei* Hook. 바위떡풀 | 4 |  |
| Saxifragaceae | *Saxifraga stolonifera* Curtis 바위취 | 3 |  |
| Rosaceae | *Aruncus dioicus* (Walter) Fernald 눈개승마 | 4 | Ⅲ |
| Rosaceae | *Malus mandshurica* (Maxim.) Kom. Ex Skvortsov 털야광나무 | 2 | Ⅰ |
| Rosaceae | *Prunus armeniaca* L. 살구나무 | 1 |  |
| Rosaceae | *Prunus davidiana* (Carrière) Franch. 산복사나무 | 1 |  |
| Rosaceae | *Rosa davurica* Pall. 생열귀나무 | 1 | Ⅳ |
| Rosaceae | *Sorbaria sorbifolia* (L.) A. Braun var. *stellipila* Maxim. 쉬땅나무 | 6 | Ⅲ |
| Rosaceae | *Sorbus commixta* Hedl. 마가목 | 1 | Ⅱ |
| Rosaceae | *Spiraea trichocarpa* Nakai 갈기조팝나무 | 1 | Ⅳ |
| Rosaceae | *Waldsteinia ternata* (Stephan) Fritsch 나도양지꽃 | 1 | Ⅳ |
| Fabaceae | *Lotus corniculatus* L. var. *japonica* Regel 벌노랑이 | 1 |  |
| Fabaceae | *Vicia angustifolia* L. var. *minor* (Bertol.) Ohwi 가는갈퀴 | 1 |  |
| Fabaceae | *Vicia cracca* L. 등갈퀴나물 | 2 |  |
| Oxalidaceae | *Oxalis obtriangulata* Maxim. 큰괭이밥 | 3 |  |
| Rutaceae | *Phellodendron amurense* Rupr. 황벽나무 | 1 | Ⅱ |
| Aceraceae | *Acer barbinerve* Maxim. 청시닥나무 | 10 | Ⅲ |
| Aceraceae | *Acer mandshuricum* Maxim. 복장나무 | 7 | Ⅲ |
| Aceraceae | *Acer tegmentosum* Maxim. 산겨릅나무 | 3 | Ⅳ |
| Aceraceae | *Acer ukurunduense* Trautv. & C. A. Mey. 부게꽃나무 | 1 | Ⅲ |
| Balsaminaceae | *Impatiens textori* Miq. var. *koreana* (Nakai) Nakai 흰물봉선 | 9 |  |
| Celastraceae | *Euonymus macropterus* Rupr. 나래회나무 | 3 | Ⅱ |
| Celastraceae | *Euonymus pauciflorus* Maxim. 회목나무 | 1 | Ⅱ |
| Tiliaceae | *Tilia amurensis* Rupr. 피나무 | 18 | Ⅱ |
| Violaceae | *Viola diamantiaca* Nakai 금강제비꽃 | 3 | LC, Ⅲ |
| Violaceae | *Viola orientalis* (Maxim.) W. Becker 노랑제비꽃 | 3 | Ⅱ |
| Violaceae | *Viola patrinii* DC. Ex Ging. 흰제비꽃 | 1 |  |
| Violaceae | *Viola variegata* Fisch. Ex Link 알록제비꽃 | 2 |  |
| Cucurbitaceae | *Schizopepon bryoniifolius* Maxim. 산외 | 3 | Ⅳ |
| Cucurbitaceae | *Sicyos angulatus* L. 가시박 | 1 | SC |
| Onagraceae | *Epilobium palustre* L. 버들바늘꽃 | 2 | DD, Ⅳ |
| Apiaceae | *Angelica anomala* Avé-Lall. 개구릿대 | 4 | Ⅰ |
| Apiaceae | *Angelica cartilaginomarginata* var. *distans* (Nakai) Kitag. 흰바디나물 | 3 |  |
| Apiaceae | *Cicuta virosa* L. 독미나리 | 5 | CR, Ⅴ |
| Apiaceae | *Ostericum maximowiczii* (F. Schmidt) Kitag. 가는바디 | 1 | Ⅳ |
| Primulaceae | *Trientalis europaea* L. var. *arctica* (Fisch. Ex Hook.) Ledeb. 기생꽃 | 1 | EN, Ⅴ |
| Oleaceae | *Syringa reticulata* (Blume) H. Hara 개회나무 | 10 | Ⅲ |
| Oleaceae | *Syringa villosa* Vahl subsp. *wolfii* (C. K. Schneid.) Y. Chen & D. Y. Hong 꽃개회나무 | 5 | LC, Ⅳ |
| Menyanthaceae | *Menyanthes trifoliata* L. 조름나물 | 2 | EN, Ⅴ |
| Convolvulaceae | *Calystegia sepium* (L.) R. Br. 큰메꽃 | 1 |  |
| Lamiaceae | *Scutellaria strigillosa* Hemsl. 참골무꽃 | 1 | Ⅰ |
| Scrophulariaceae | *Pedicularis resupinata* L. var. *umbrosa* (Kom.) Nakai 그늘송이풀 | 1 | ED |
| Scrophulariaceae | *Pseudolysimachion rotundum* var. *subintegrum* (Nakai) T. Yamaz. 산꼬리풀 | 2 |  |
| Scrophulariaceae | *Scrophularia kakudensis* Franch. 큰개현삼 | 2 | Ⅱ |
| Caprifoliaceae | *Lonicera nigra* L. var. *barbinervis* (Kom.) Nakai 암괴불나무 | 1 |  |
| Valerianaceae | *Patrinia saniculifolia* Hemsl. 금마타리 | 1 | LC, ED, Ⅲ |
| Campanulaceae | *Hanabusaya asiatica* (Nakai) Nakai 금강초롱꽃 | 1 | VU, ED, Ⅳ |
| Asteraceae | *Achillea alpina* L. var*. discoidea* (Regel) Kitam. 산톱풀 | 1 |  |
| Asteraceae | *Artemisia sacrorum* Ledeb. 털산쑥 | 1 |  |
| Asteraceae | *Carpesium triste* Maxim. 두메담배풀 | 5 |  |
| Asteraceae | *Cirsium schantarense* Trautv. & C. A. Mey. 도깨비엉겅퀴 | 2 | Ⅲ |
| Asteraceae | *Crepidiastrum chelidoniifolium* (Makino) J. H. Pak & Kawano 까치고들빼기 | 1 |  |
| Asteraceae | *Dendranthema oreastrum* (Hance) Y. Ling 바위구절초 | 1 |  |
| Asteraceae | *Erigeron floribundus* (Kunth) Sch. Bip. 큰망초 | 1 | CS |
| Asteraceae | *Erigeron philadelphicus* L. 봄망초 | 2 | CS |
| Asteraceae | *Galinsoga quadriradiata* Ruiz & Pav. 털별꽃아재비 | 3 | WS |
| Asteraceae | *Ixeris strigosa* (H.Lév. & Vaniot) J. H. Pak & Kawano 선씀바귀 | 1 |  |
| Asteraceae | *Lactuca triangulata* Maxim. 두메고들빼기 | 2 |  |
| Asteraceae | *Ligularia intermedia* Nakai 어리곤달비 | 1 |  |
| Asteraceae | *Parasenecio auriculatus* (DC.) J. R. Grant 귀박쥐나물 | 2 | LC, Ⅳ |
| Asteraceae | *Parasenecio auriculatus* var. *kamtschatica* (Maxim.) H. Koyama 나래박쥐나물 | 3 | Ⅲ |
| Asteraceae | *Parasenecio hastatus* (L.) H. Koyama var. *orientalis* (Kitam.) H. Koyama 민박쥐나물 | 3 | Ⅲ |
| Asteraceae | *Rudbeckia laciniata* L. 삼잎국화 | 1 |  |
| Asteraceae | *Saussurea gracilis* Maxim. 은분취 | 1 |  |
| Asteraceae | *Saussurea grandicapitula* W. T. Lee & H. T. Im 태백취 | 1 | ED, Ⅳ |
| Asteraceae | *Saussurea grandifolia* Maxim. 서덜취 | 4 |  |
| Asteraceae | *Saussurea macrolepis* (Nakai) Kitam. 각시서덜취 | 4 | ED |
| Asteraceae | *Saussurea tanakae* Franch. & Sav. Ex Maxim. 당분취 | 1 |  |
| Asteraceae | *Scorzonera albicaulis* Bunge 쇠채 | 1 | VU |
| Asteraceae | *Synurus excelsus* (Makino) Kitam. 큰수리취 | 4 |  |
| Liliaceae | *Asparagus oligoclonos* Maxim. 방울비짜루 | 1 | Ⅰ |
| Liliaceae | *Lilium distichum* Nakai ex Kamib. 말나리 | 5 | LC, Ⅲ |
| Liliaceae | *Lilium lancifolium* Thunb. 참나리 | 2 |  |
| Liliaceae | *Maianthemum bifolium* (L.) F. W. Schmidt 두루미꽃 | 4 | Ⅱ |
| Liliaceae | *Polygonatum humile* Fisch. Ex Maxim. 각시둥굴레 | 2 |  |
| Liliaceae | *Polygonatum* × *desoulavyi* Kom. 안면용둥굴레 | 1 |  |
| Liliaceae | *Streptopus ovalis* (Ohwi) F. T. Wang & Y. C. Tang 금강애기나리 | 3 | LC |
| Liliaceae | *Trillium camschatcense* Ker Gawl. 연영초 | 4 | LC, Ⅳ |
| Liliaceae | *Trillium tschonoskii* Maxim. 큰연영초 | 1 | VU, Ⅳ |
| Liliaceae | *Veratrum dolichopetalum* O. Loes. 푸른박새 | 2 |  |
| Liliaceae | *Veratrum nigrum* L. var. *ussuriense* O. Loes. 참여로 | 1 | Ⅲ |
| Poaceae | *Arundo donax* L. 물대 | 1 |  |
| Poaceae | *Diarrhena mandshurica* Maxim. 껍질용수염 | 7 |  |
| Poaceae | *Festuca ovina* L. var. *koreanoalpina* Ohwi 두메김의털 | 1 | ED |
| Poaceae | *Melica nutans* L. 왕쌀새 | 1 | Ⅱ |
| Poaceae | *Microstegium japonicum* (Miq.) Koidz. 민바랭이새 | 4 |  |
| Araceae | *Acorus gramineus* Aiton 석창포 | 1 | Ⅲ |
| Araceae | *Symplocarpus nipponicus* Makino 애기앉은부채 | 4 | Ⅲ |
| Typhaceae | *Sparganium japonicum* Rothert 긴흑삼릉 | 1 | DD, Ⅳ |
| Cyperaceae | *Bolboschoenus fluviatilis* (Torr.) Soják 큰매자기 | 1 |  |
| Cyperaceae | *Bolboschoenus maritimus* (L.) Palla 매자기 | 1 | Ⅱ |
| Cyperaceae | *Carex arenicola* F.Schmidt 진퍼리사초 | 1 | Ⅳ |
| Cyperaceae | *Carex canescens* L. 산사초 | 1 | Ⅳ |
| Cyperaceae | *Carex fernaldiana* H. Lév. & Vaniot 실사초 | 2 |  |
| Cyperaceae | *Carex hakonensis* Franch. & Sav. 애기바늘사초 | 3 |  |
| Cyperaceae | *Carex lasiocarpa* Ehrh. 벌사초 | 1 | Ⅳ |
| Cyperaceae | *Carex lasiolepis* Franch. 난사초 | 1 | Ⅲ |
| Cyperaceae | *Carex lenta* D. Don 줄사초 | 1 | Ⅰ |
| Cyperaceae | *Carex pilosa* Scop. 털사초 | 4 | Ⅲ |
| Cyperaceae | *Carex pumila* Thunb. 좀보리사초 | 1 |  |
| Cyperaceae | *Carex quadriflora* (Kük.) Ohwi 녹빛사초 | 1 | Ⅱ |
| Cyperaceae | *Carex rhynchophysa* Fisch., C. A. Mey. & Avé-Lall. 왕삿갓사초 | 1 | Ⅳ |
| Cyperaceae | *Carex stipata* Muhl. Ex Willd. 양덕사초 | 3 | Ⅳ |
| Cyperaceae | *Carex tenuiflora* Wahlenb. 별사초 | 2 | Ⅳ |
| Cyperaceae | *Cyperus exaltatus* Retz. var. *iwasakii* (Makino) T. Koyama 왕골 | 1 |  |
| Cyperaceae | *Cyperus nipponicus* Franch. & Sav. 푸른방동사니 | 1 |  |
| Orchidaceae | *Galearis cyclochila* (Franch. & Sav.) Soó 나도제비란 | 5 | VU, Ⅱ |
| Orchidaceae | *Liparis makinoana* Schltr. 나리난초 | 2 | Ⅰ |
| Orchidaceae | *Oreorchis patens* (Lindl.) Lindl. 감자난초 | 1 |  |
| Orchidaceae | *Platanthera sachalinensis* F. Schmidt 큰제비란 | 2 | Ⅳ |

**^*^Fre: Frequency, RP.: Remarkable plants (Rare plants: CR, EN, VU, LC, DD), ED: Endemic plants, Floristic target plants: Ⅰ~Ⅴ, Invasive alien plants: WS, SS, SR, SC, CS**
