# Supplementary material for: Floristic inventory and distribution characteristics of vascular plants in forest wetlands of South Korea
Source: Biodivers Data J. 2022 Sep 15;10:e85848. doi: 10.3897/BDJ.10.e85848 (PMC9848468; doi:10.3897/BDJ.10.e85848)
Supplement: Supplementary material 13 — Vascular plants recorded only in forest wetlands of Gyeonggi region, Korea. [file bdj-10-e85848-s013.docx]

Table 13. Vascular plants recorded only in forest wetlands of Gyeonggi region, Korea.

| Family name | Scientific name / Korean name | Fre. | RP. |
| --- | --- | --- | --- |
| Poaceae | *Calamagrostis pseudophragmites* (Haller f.) Koeler 갯조풀 | 1 |  |
| Cyperaceae | *Scirpus orientalis* Ohwi 검은도루박이 | 1 | DD, Ⅳ |
| Poaceae | *Echinochloa oryzicola* (Vasinger) Vasinger 논피 | 1 |  |
| Saxifragaceae | *Mukdenia rossii* (Oliv.) Koidz. 돌단풍 | 1 | Ⅱ |
| Berberidaceae | *Berberis amurensis* Rupr. 매발톱나무 | 1 | Ⅱ |
| Elaeagnaceae | *Elaeagnus glabra* Thunb. 보리장나무 | 3 | Ⅲ |
| Rosaceae | *Prunus japonica* Thunb. 산이스라지 | 2 | ED |
| Berberidaceae | *Epimedium koreanum* Nakai 삼지구엽초 | 1 | VU, Ⅳ |
| Juncaceae | *Juncus bufonius* L. 애기골풀 | 1 |  |
| Liliaceae | *Hemerocallis minor* Mill. 애기원추리 | 1 |  |
| Ranunculaceae | *Clematis brachyura* Maxim. 외대으아리 | 1 | ED, Ⅲ |
| Magnoliaceae | *Magnolia obovata* Thunb. 일본목련 | 2 |  |
| Liliaceae | *Hosta clausa* Nakai 주걱비비추 | 1 | Ⅲ |
| Cyperaceae | *Carex sabynensis* Less. ex Kunth var. *leiosperma* Ohwi 지리실청사초 | 2 | ED |
| Brassicaceae | *Arabis hirsuta* (L.) Scop. 털장대 | 1 |  |
| Liliaceae | *Veratrum maackii* Regel var. *parviflorum* (Maxim. ex Miq.) H. Hara 파란여로 | 1 | Ⅳ |
| Rubiaceae | *Galium tokyoense* Makino 흰갈퀴 | 2 |  |
| Violaceae | *Viola hirtipes* S. Moore 흰털제비꽃 | 1 |  |
| Polygonaceae | *Bistorta incana* (Nakai) Nakai ex T. Mori 흰범꼬리 | 1 |  |

**^*^Fre: Frequency, RP.: Remarkable plants (Rare plants: CR, EN, VU, LC, DD), ED: Endemic plants, Floristic target plants: Ⅰ~Ⅴ, Invasive alien plants: WS, SS, SR, SC, CS**
