# Supplementary material for: Floristic inventory and distribution characteristics of vascular plants in forest wetlands of South Korea
Source: Biodivers Data J. 2022 Sep 15;10:e85848. doi: 10.3897/BDJ.10.e85848 (PMC9848468; doi:10.3897/BDJ.10.e85848)
Supplement: Supplementary material 14 — Vascular plants recorded only in forest wetlands of Gyeongsang region [file bdj-10-e85848-s014.docx]

Table 14. Vascular plants recorded only in forest wetlands of Gyeongsang region

| Family name | Scientific name / Korean name | Fre. | RP. |
| --- | --- | --- | --- |
| Thelypteridaceae | *Parathelypteris beddomei* (Baker) Ching 가는잎처녀고사리 | 1 | Ⅲ |
| Liliaceae | *Hemerocallis dumortieri* C. Morren 각시원추리 | 1 |  |
| Rosaceae | *Prunus mandshurica* (Maxim.) Koehne 개살구나무 | 1 | Ⅲ |
| Fabaceae | *Medicago polymorpha* L. 개자리 | 1 | SC |
| Boraginaceae | *Trigonotis radicans* (Turcz.) Steven 거센털꽃마리 | 1 | EN, Ⅲ |
| Eriocaulaceae | *Eriocaulon parvum* Körn. 검은개수염 | 1 |  |
| Oleaceae | *Osmanthus heterophyllus* (G. Don) P. S. Green. 구골나무 | 1 | Ⅳ |
| Cyperaceae | *Carex tegulata* H. Lév. & Vaniot 구슬사초 | 1 | Ⅲ |
| Poaceae | *Elymus repens* (L.) Gould 구주개밀 | 1 | CS |
| Rosaceae | *Sanguisorba longifolia* Bertol. 긴오이풀 | 1 | Ⅳ |
| Sabiaceae | *Meliosma myriantha* Siebold & Zucc. 나도밤나무 | 1 | Ⅰ |
| Apiaceae | *Pimpinella komarovii* (Kitag.) R. H. Shan & F. T. Pu 노루참나물 | 1 |  |
| Salicaceae | *Salix pseudolasiogyne* H. Lév. 능수버들 | 1 |  |
| Alangiaceae | *Alangium platanifolium* (Siebold & Zucc.) Harms 단풍박쥐나무 | 1 | DD |
| Ulmaceae | *Ulmus davidiana* Planch. ex DC. 당느릅나무 | 1 | Ⅰ |
| Polygonaceae | *Persicaria erecto-minor* (Makino) Nakai var. *koreensis* (Nakai) I. Ito 대동여뀌 | 2 | Ⅲ |
| Eucommiaceae | *Eucommia ulmoides* Oliv. 두충 | 1 |  |
| Euphorbiaceae | *Euphorbia humifusa* Willd. ex Schltdl. 땅빈대 | 2 |  |
| Fagaceae | *Quercus mongolica* Fisch. ex Ledeb. var. *crispula* (Blume) H. Ohashi 물참나무 | 2 |  |
| Selaginellaceae | *Selaginella tamariscina* (P. Beauv.) Spring 바위손 | 1 | Ⅲ |
| Gleicheniaceae | *Dicranopteris linearis* (Burm.f.) Underw. 발풀고사리 | 1 | Ⅲ |
| Poaceae | *Briza minor* L. 방울새풀 | 4 | CS |
| Asteraceae | *Inula salicina* L. 버들금불초 | 2 | VU |
| Balsaminaceae | *Impatiens balsamina* L. 봉선화 | 2 |  |
| Asteraceae | *Saussurea mongolica* (Franch.) Franch. 북분취 | 1 |  |
| Caprifoliaceae | *Viburnum carlesii* Hemsl. 분꽃나무 | 3 |  |
| Ceratophyllaceae | *Ceratophyllum demersum* L. 붕어마름 | 1 |  |
| Asteraceae | *Stemmacantha uniflora* (L.) Dittrich 뻐꾹채 | 1 | Ⅰ |
| Asteraceae | *Sonchus brachyotus* DC. 사데풀 | 1 |  |
| Orchidaceae | *Goodyera schlechtendaliana* Rchb.f. 사철란 | 1 | LC |
| Asteraceae | *Artemisia capillaris* Thunb. 사철쑥 | 2 |  |
| Liliaceae | *Polygonatum thunbergii* C. Morren & Decne. 산둥굴레 | 1 |  |
| Dryopteridaceae | *Dryopteris polylepis* (Franch. & Sav.) C.Chr. 산비늘고사리 | 1 |  |
| Thymelaeaceae | *Edgeworthia chrysantha* Lindl. 삼지닥나무 | 1 |  |
| Geraniaceae | *Geranium krameri* Franch. & Sav. 선이질풀 | 3 |  |
| Cyperaceae | *Scirpus mitsukurianus* Makino 솔방울골 | 1 |  |
| Asteraceae | *Leibnitzia anandria* (L.) Turcz. 솜나물 | 2 |  |
| Onagraceae | *Circaea cordata* Royle 쇠털이슬 | 1 | Ⅰ |
| Malvaceae | *Hibiscus trionum* L. 수박풀 | 1 | SC |
| Asteraceae | *Senecio argunensis* Turcz. 쑥방망이 | 2 | VU, Ⅰ |
| Scrophulariaceae | *Euphrasia maximowiczii* Wettst. ex Palib. 앉은좁쌀풀 | 1 | Ⅲ |
| Cyperaceae | *Cyperus difformis* L. 알방동사니 | 1 |  |
| Rosaceae | *Prunus tomentosa* Thunb. 앵도나무 | 1 |  |
| Salicaceae | *Populus nigra* L. 양버들 | 2 |  |
| Asteraceae | *Aster fastigiatus* Fisch. 옹굿나물 | 2 | VU |
| Poaceae | *Eleusine indica* (L.) Gaertn. 왕바랭이 | 2 |  |
| Brassicaceae | *Cardamine yezoensis* Maxim. 왜갓냉이 | 2 | Ⅳ |
| Lamiaceae | *Stachys oblongifolia* Benth. 우단석잠풀 | 1 |  |
| Asteraceae | *Bidens pilosa* L. 울산도깨비바늘 | 2 | SC |
| Asteraceae | *Rudbeckia bicolor* Nutt. 원추천인국 | 1 | SR |
| Asteraceae | *Artemisia koidzumii* Nakai 율무쑥 | 1 |  |
| Apiaceae | *Angelica czernaevia* (Fisch. & C. A. Mey.) Kitag. 잔잎바디 | 1 |  |
| Brassicaceae | *Berteroella maximowiczii* (Palib.) O. E. Schulz 장대냉이 | 1 |  |
| Scrophulariaceae | *Siphonostegia chinensis* Benth. 절국대 | 1 |  |
| Poaceae | *Setaria chondrachne* (Steud.) Honda 조아재비 | 2 | Ⅰ |
| Fabaceae | *Lespedeza virgata* (Thunb.) DC. 좀싸리 | 2 |  |
| Celastraceae | *Euonymus hamiltonianus* Wall. var. *maackii* (Rupr.) Kom. 좀참빗살나무 | 3 |  |
| Cyperaceae | *Carex okamotoi* Ohwi 지리대사초 | 2 | ED, Ⅰ |
| Rosaceae | *Filipendula formosa* Nakai 지리터리풀 | 1 | ED, Ⅳ |
| Alismataceae | *Alisma orientale* (Sam.) Juz. 질경이택사 | 2 | Ⅱ |
| Theaceae | *Camellia sinensis* (L.) Kuntze 차나무 | 1 |  |
| Boraginaceae | *Symphytum officinale* L. 컴프리 | 1 | SC |
| Asteraceae | *Cosmos bipinnatus* Cav. 코스모스 | 1 | SR |
| Brassicaceae | *Lepidium virginicum* L. 콩다닥냉이 | 1 | SS |
| Fabaceae | *Vicia pseudoorobus* Fisch. & C. A. Mey. 큰등갈퀴 | 1 | Ⅰ |
| Poaceae | *Elymus gmelinii* (Ledeb.) Tzvelev 털개밀 | 2 |  |
| Fabaceae | *Lespedeza maritima* Nakai 해변싸리 | 3 | ED, Ⅰ |
| Rhamnaceae | *Hovenia dulcis* Thunb. 헛개나무 | 1 | Ⅰ |
| Poaceae | *Lolium perenne* L. 호밀풀 | 1 | SS |
| Fabaceae | *Lespedeza davurica* (Laxm.) Schindl. 호비수리 | 1 | Ⅲ |
| Poaceae | *Schizachne purpurascens* (Torr.) Swallen subsp. *callosa* (Turcz. ex Griseb.) T. Koyama & Kawano 호오리새 | 1 | Ⅱ |

**^*^Fre: Frequency, RP.: Remarkable plants (Rare plants: CR, EN, VU, LC, DD), ED: Endemic plants, Floristic target plants: Ⅰ~Ⅴ, Invasive alien plants: WS, SS, SR, SC, CS**
