# Supplementary material for: Floristic inventory and distribution characteristics of vascular plants in forest wetlands of South Korea
Source: Biodivers Data J. 2022 Sep 15;10:e85848. doi: 10.3897/BDJ.10.e85848 (PMC9848468; doi:10.3897/BDJ.10.e85848)
Supplement: Supplementary material 15 — Vascular plants recorded only in forest wetlands of Chungcheong region, Korea. [file bdj-10-e85848-s015.docx]

Table 15. Vascular plants recorded only in forest wetlands of Chungcheong region, Korea.

| Family name | Scientific name / Korean name | Fre. | RP. |
| --- | --- | --- | --- |
| Polygonaceae | *Reynoutria forbesii* (Hance) T. Yamaz. 감절대 | 1 |  |
| Polygonaceae | *Rumex longifolius* DC. 개대황 | 1 | DD |
| Rubiaceae | *Galium trifloriforme* Kom. 개선갈퀴 | 1 |  |
| Aspleniaceae | *Asplenium ruprechtii* Sa. Kurata 거미고사리 | 1 |  |
| Caprifoliaceae | *Lonicera harae* Makino 길마가지나무 | 1 |  |
| Moraceae | *Broussonetia papyrifera* (L.) L’ Hér. ex Vent. 꾸지나무 | 1 |  |
| Poaceae | *Sporobolus piliferus* (Trin.) Kunth 나도잔디 | 1 |  |
| Fabaceae | *Indigofera pseudotinctoria* Matsum. 낭아초 | 2 | Ⅲ |
| Iridaceae | *Iris pseudacorus* L. 노랑꽃창포 | 1 |  |
| Verbenaceae | *Tripora divaricata* (Maxim.) P. D. Cantino 누린내풀 | 1 |  |
| Brassicaceae | *Catolobus pendulus* (L.) Al-Shehbaz 느러진장대 | 1 | Ⅲ |
| Saxifragaceae | *Rodgersia podophylla* A. Gray 도깨비부채 | 1 | LC, Ⅳ |
| Poaceae | *Koeleria macrantha* (Ledeb.) Schult. 도랭이피 | 1 |  |
| Moraceae | *Morus cathayana* Hemsl. 돌뽕나무 | 1 | Ⅲ |
| Poaceae | *Vulpia myuros* (L.) C.C. Gmel. 들묵새 | 1 | SC |
| Cucurbitaceae | *Actinostemma lobatum* (Maxim.) Maxim. ex Franch. & Sav. 뚜껑덩굴 | 1 | Ⅰ |
| Cupressaceae | *Metasequoia glyptostroboides* Hu & W. C. Cheng 메타세쿼이아 | 1 |  |
| Asteraceae | *Solidago gigantea* Aiton 미국미역취 | 1 | SR |
| Poaceae | *Digitaria violascens* Link 민바랭이 | 1 |  |
| Cyperaceae | *Fimbristylis squarrosa* Vahl 민하늘지기 | 1 |  |
| Platanaceae | *Platanus orientalis* L. 버즘나무 | 1 |  |
| Asteraceae | *Ixeris japonica* (Burm.f.) Nakai 벋음씀바귀 | 1 |  |
| Asteraceae | *Saussurea seoulensis* Nakai 분취 | 1 | ED |
| Cyperaceae | *Carex heterolepis* Bunge 산비늘사초 | 1 |  |
| Ulmaceae | *Celtis aurantiaca* Nakai 산팽나무 | 1 |  |
| Poaceae | *Poa annua* L. 새포아풀 | 1 |  |
| Poaceae | *Themeda triandra* Forssk. 솔새 | 1 |  |
| Poaceae | *Schizachyrium brevifolium* (Sw.) Nees ex Büse 쇠풀 | 1 |  |
| Rutaceae | *Tetradium daniellii* (Benn.) T. G. Hartley 쉬나무 | 1 |  |
| Dryopteridaceae | *Dryopteris sacrosancta* Koidz. 애기족제비고사리 | 2 |  |
| Primulaceae | *Primula sieboldii* E. Morren 앵초 | 2 | Ⅱ |
| Cyperaceae | *Carex capricornis* Meinsh. ex Maxim. 양뿔사초 | 1 | CR, Ⅳ |
| Apiaceae | *Aegopodium alpestre* Ledeb. 왜방풍 | 1 | Ⅳ |
| Aceraceae | *Acer saccharinum* L. 은단풍 | 2 |  |
| Verbenaceae | *Callicarpa dichotoma* (Lour.) Raeusch. ex K. Koch 좀작살나무 | 1 | Ⅲ |
| Rosaceae | *Spiraea microgyna* Nakai 좀조팝나무 | 1 | ED, Ⅲ |
| Rubiaceae | *Galium koreanum* (Nakai) Nakai 참갈퀴덩굴 | 1 | ED |
| Cyperaceae | *Cyperus iria* L. 참방동사니 | 1 |  |
| Fabaceae | *Vicia unijuga* A. Braun var. *ouensanensis* H. Lév. 큰나비나물 | 1 |  |
| Caryophyllaceae | *Silene firma* Siebold & Zucc. f. *pubescens* (Makino) Ohwi & H. Ohashi 털장구채 | 1 |  |
| Liliaceae | *Polygonatum odoratum* (Mill.) Druce 풍도둥굴레 | 1 |  |
| Apocynaceae | *Cynanchum nipponicum* Matsum. var. *glabrum* (Nakai) H. Hara 흑박주가리 | 1 | Ⅲ |

**^*^Fre: Frequency, RP.: Remarkable plants (Rare plants: CR, EN, VU, LC, DD), ED: Endemic plants, Floristic target plants: Ⅰ~Ⅴ, Invasive alien plants: WS, SS, SR, SC, CS**
