# Supplementary material for: Floristic inventory and distribution characteristics of vascular plants in forest wetlands of South Korea
Source: Biodivers Data J. 2022 Sep 15;10:e85848. doi: 10.3897/BDJ.10.e85848 (PMC9848468; doi:10.3897/BDJ.10.e85848)
Supplement: Supplementary material 16 — Vascular plants that appeared only in forest wetlands of Jeolla region, Korea. [file bdj-10-e85848-s016.docx]

Table 16. Vascular plants that appeared only in forest wetlands of Jeolla region, Korea.

| Family name | Scientific name / Korean name | Fre. | RP. |
| --- | --- | --- | --- |
| Asteraceae | *Lactuca indica* L. var. *laciniata* (Houtt.) H. Hara f. *indivisa* (Maxim.) H. Hara 가는잎왕고들빼기 | 1 |  |
| Pteridaceae | *Coniogramme japonica* (Thunb.) Diels 가지고비고사리 | 1 | Ⅲ |
| Asteraceae | *Chrysanthemum indicum* L. 감국 | 1 |  |
| Asteraceae | *Lapsanastrum apogonoides* (Maxim.) J. H. Pak & K. Bremer 개보리뺑이 | 1 | Ⅰ |
| Cephalotaxaceae | *Cephalotaxus harringtonia* (Knight ex J. Forbes) K. Koch 개비자나무 | 1 | Ⅰ |
| Rutaceae | *Zanthoxylum armatum* DC. 개산초 | 1 | Ⅰ |
| Poaceae | *Eulalia speciosa* (Debeaux) Kuntze 개억새 | 1 |  |
| Apiaceae | *Cnidium japonicum* Miq. 갯사상자 | 1 | Ⅰ |
| Poaceae | *Ischaemum anthephoroides* (Steud.) Miq. 갯쇠보리 | 4 | Ⅰ |
| Poaceae | *Zoysia sinica* Hance 갯잔디 | 1 |  |
| Plumbaginaceae | *Limonium tetragonum* (Thunb.) Bullock 갯질경 | 1 |  |
| Poaceae | *Agrostis canina* L. 검은겨이삭 | 3 |  |
| Cornaceae | *Cornus macrophylla* Wall. 곰의말채나무 | 2 |  |
| Lamiaceae | *Teucrium veronicoides* Maxim. 곽향 | 1 | Ⅲ |
| Fabaceae | *Lespedeza pilosa* (Thunb.) Siebold & Zucc. 괭이싸리 | 2 |  |
| Orchidaceae | *Cephalanthera falcata* (Thunb.) Blume 금난초 | 2 | Ⅲ |
| Lamiaceae | *Ajuga decumbens* Thunb. 금창초 | 1 | Ⅰ |
| Polygonaceae | *Persicaria breviochreata* (Makino) Ohki 긴화살여뀌 | 1 | Ⅰ |
| Convolvulaceae | *Ipomoea nil* (L.) Roth 나팔꽃 | 1 |  |
| Juncaceae | *Juncus alatus* Franch. & Sav. 날개골풀 | 2 |  |
| Cyperaceae | *Scleria parvula* Steud. 너도고랭이 | 5 |  |
| Asteraceae | *Ixeris chinensis* (Thunb.) Nakai 노랑선씀바귀 | 1 |  |
| Liliaceae | *Hemerocallis thunbergii* Baker 노랑원추리 | 5 |  |
| Brassicaceae | *Cardamine lyrata* Bunge 논냉이 | 2 |  |
| Caprifoliaceae | *Lonicera caerulea* L. 댕댕이나무 | 1 | VU, Ⅳ |
| Convolvulaceae | *Quamoclit angulata* (Lam.) Bojer 둥근잎유홍초 | 1 | SR |
| Thelypteridaceae | *Metathelypteris laxa* (Franch. & Sav.) Ching 드문고사리 | 1 | Ⅲ |
| Lentibulariaceae | *Utricularia aurea* Lour. 들통발 | 1 | CR, Ⅴ |
| Crassulaceae | *Sedum oryzifolium* Makino 땅채송화 | 1 |  |
| Asteraceae | *Tagetes minuta* L. 만수국아재비 | 1 | SC |
| Crassulaceae | *Sedum bulbiferum* Makino 말똥비름 | 2 |  |
| Staphyleaceae | *Euscaphis japonica* (Thunb.) Kanitz 말오줌때 | 3 | Ⅰ |
| Papaveraceae | *Coreanomecon hylomeconoides* Nakai 매미꽃 | 1 | LC, ED, Ⅳ |
| Poaceae | *Avena fatua* L. 메귀리 | 1 | SC |
| Poaceae | *Phacelurus latifolius* (Steud.) Ohwi 모새달 | 1 | LC, Ⅰ |
| Poaceae | *Arundinaria munsuensis* Y. N. Lee 문수조릿대 | 1 | ED, Ⅴ |
| Cyperaceae | *Schoenoplectus nipponicus* (Makino) Soják 물고랭이 | 1 | Ⅱ |
| Cyperaceae | *Carex boottiana* Hook. & Arn. 밀사초 | 1 |  |
| Crassulaceae | *Sedum polytrichoides Hemsl. 바위채송화* | 1 |  |
| Orchidaceae | *Pogonia minor (Makino) Makino 방울새란* | 4 | VU |
| Cyperaceae | *Cyperus hakonensis* Franch. & Sav. 병아리방동사니 | 1 |  |
| Euphorbiaceae | *Neoshirakia japonica* (Siebold & Zucc.) Esser 사람주나무 | 2 | Ⅰ |
| Lamiaceae | *Mosla japonica* (Benth. ex Oliv.) Maxim. 산들깨 | 1 | VU |
| Rutaceae | *Orixa japonica* Thunb. 상산 | 1 | Ⅰ |
| Cucurbitaceae | *Melothria japonica* (Thunb.) Maxim. ex Cogn. 새박 | 4 | LC, Ⅰ |
| Verbenaceae | *Callicarpa mollis* Siebold & Zucc. 새비나무 | 1 | Ⅲ |
| Asteraceae | *Achillea millefolium* L. 서양톱풀 | 1 | SC |
| Araliaceae | *Hedera rhombea* (Miq.) Siebold & Zucc. ex Bean 송악 | 2 | Ⅰ |
| Verbenaceae | *Vitex rotundifolia* L.f. 순비기나무 | 1 | Ⅱ |
| Caryophyllaceae | *Dianthus longicalyx* Miq. 술패랭이꽃 | 1 |  |
| Lygodiaceae | *Lygodium japonicum* (Thunb.) Sw. 실고사리 | 1 | Ⅰ |
| Potamogetonaceae | *Potamogeton pusillus* L. 실말 | 1 |  |
| Asteraceae | *Conyza bonariensis* (L.) Cronquist 실망초 | 1 | SS |
| Asteraceae | *Artemisia angustissima* Nakai 실제비쑥 | 1 |  |
| Dryopteridaceae | *Dryopteris kinkiensis* Koidz. ex Tagawa 엷은잎지네고사리 | 1 |  |
| Poaceae | *Phyllostachys reticulata* (Rupr.) K. Koch. 왕대 | 1 |  |
| Urticaceae | *Boehmeria pannosa* Nakai & Satake ex Oka 왕모시풀 | 1 | Ⅲ |
| Asteraceae | *Prenanthes ochroleuca* (Maxim.) Hemsl. 왕씀배 | 1 | VU |
| Verbenaceae | *Callicarpa japonica* Thunb. var. *luxurians* Rehder 왕작살나무 | 1 |  |
| Scrophulariaceae | *Lindernia crustacea* (L.) F. Muell. 외풀 | 1 |  |
| Cyperaceae | *Cyperus tenuispica* Steud. 우산방동사니 | 2 |  |
| Oleaceae | *Chionanthus retusus* Lindl. & Paxton 이팝나무 | 1 | LC, Ⅲ |
| Cyperaceae | *Carex capillacea* Boott 잔솔잎사초 | 1 |  |
| Ericaceae | *Vaccinium oldhamii* Miq. 정금나무 | 5 | Ⅰ |
| Poaceae | *Lophatherum gracile* Brongn. 조릿대풀 | 2 | Ⅲ |
| Asteraceae | *Aster maackii* Regel 좀개미취 | 1 | Ⅲ |
| Asteraceae | *Carpesium cernuum* L. 좀담배풀 | 1 |  |
| Fabaceae | *Indigofera koreana* Ohwi 좀땅비싸리 | 1 | ED |
| Liliaceae | *Polygonatum lasianthum* Maxim. 죽대 | 1 |  |
| Nephrolepidaceae | *Nephrolepis cordifolia* (L.) C. Presl 줄고사리 | 1 | Ⅴ |
| Juncaginaceae | *Triglochin maritima* L. 지채 | 1 | Ⅰ |
| Poaceae | *Glyceria ischyroneura* Steud. 진들피 | 1 |  |
| Liliaceae | *Polygonatum falcatum* A. Gray 진황정 | 1 | Ⅲ |
| Juncaceae | *Juncus filiformis* L. 참골풀 | 1 |  |
| Ranunculaceae | *Clematis terniflora* DC. 참으아리 | 1 |  |
| Clusiaceae | *Hypericum attenuatum* Fisch. ex Choisy 채고추나물 | 5 | DD, Ⅱ |
| Cyperaceae | *Carex scabrifolia* Steud. 천일사초 | 1 |  |
| Verbenaceae | *Caryopteris incana* (Thunb. ex Houtt.) Miq. 층꽃나무 | 1 | Ⅰ |
| Cyperaceae | *Cladium chinense* Nees 층층고랭이 | 1 | Ⅳ |
| Lauraceae | *Lindera sericea* (Siebold & Zucc.) Blume 털조장나무 | 1 | LC, Ⅳ |
| Ulmaceae | *Aphananthe aspera* (Thunb.) Planch. 푸조나무 | 1 | Ⅰ |
| Fabaceae | *Lespedeza thunbergii* (DC.) Nakai 풀싸리 | 1 |  |
| Poaceae | *Echinochloa esculenta* (A. Braun) H. Scholz 피 | 2 |  |
| Asteraceae | *Eclipta prostrata* (L.) L. 한련초 | 1 |  |
| Sabiaceae | *Meliosma pinnata* var. *oldhamii* (Miq. ex Maxim.) Beusekom 합다리나무 | 2 | Ⅰ |
| Orchidaceae | *Habenaria radiata* (Thunb.) Spreng. 해오라비난초 | 1 | CR, Ⅴ |
| Chenopodiaceae | *Suaeda maritima* (L.) Dumort. 해홍나물 | 1 | Ⅱ |
| Cyperaceae | *Carex pseudochinensis* H. Lév. & Vaniot 햇사초 | 1 | DD, ED |
| Asteraceae | *Eupatorium tripartitum* (Makino) Murata & H. Koyama 향등골나물 | 1 |  |
| Juglandaceae | *Juglans regia* L. 호두나무 | 1 |  |
| Dryopteridaceae | *Dryopteris maximowicziana* (Miq.) C.Chr. 흰비늘고사리 | 1 | Ⅴ |
| Asteraceae | *Ixeridium dentatum* (Thunb.) Tzvelev f. *albiflora* (Makino) H. Hara 흰씀바귀 | 1 |  |
| Liliaceae | *Veratrum versicolor* Nakai 흰여로 | 1 |  |

**^*^Fre: Frequency, RP.: Remarkable plants (Rare plants: CR, EN, VU, LC, DD), ED: Endemic plants, Floristic target plants: Ⅰ~Ⅴ, Invasive alien plants: WS, SS, SR, SC, CS**
