# Supplementary material for: Floristic inventory and distribution characteristics of vascular plants in forest wetlands of South Korea
Source: Biodivers Data J. 2022 Sep 15;10:e85848. doi: 10.3897/BDJ.10.e85848 (PMC9848468; doi:10.3897/BDJ.10.e85848)
Supplement: Supplementary material 17 — Vascular plants recorded only in forest wetlands of Jeju region, Korea. [file bdj-10-e85848-s017.docx]

Table 17. Vascular plants recorded only in forest wetlands of Jeju region, Korea.

| Family name | Scientific name / Korean name | Fre. | RP. |
| --- | --- | --- | --- |
| Potamogetonaceae | *Potamogeton cristatus* Regel & Maack 가는가래 | 3 | Ⅰ |
| Isoetaceae | *Isoetes sinensis* Palmer 가는물부추 | 1 |  |
| Asteraceae | *Cirsium japonicum* Fisch. ex DC. var. *spinossimum* Kitam. 가시엉겅퀴 | 1 |  |
| Rosaceae | *Prunus maackii* Rupr. 개벚지나무 | 1 | Ⅲ |
| Rosaceae | *Rubus buergeri* Miq. 겨울딸기 | 1 | Ⅲ |
| Polygonaceae | *Persicaria taquetii* (H. Lév.) Koidz. 겨이삭여뀌 | 1 | Ⅳ |
| Dryopteridaceae | *Dryopteris saxifragi-varia* Nakai 광릉족제비고사리 | 1 |  |
| Gentianaceae | *Gentiana squarrosa* Ledeb. 구슬붕이 | 1 |  |
| Scrophulariaceae | *Limnophila sessiliflora* (Vahl) Blume 구와말 | 1 | Ⅱ |
| Daphniphyllaceae | *Daphniphyllum macropodum* Miq. 굴거리나무 | 2 | Ⅲ |
| Cyperaceae | *Carex mitrata* Franch. var. *aristata* Ohwi 까락겨사초 | 1 | Ⅰ |
| Lauraceae | *Litsea japonica* (Thunb.) Juss. 까마귀쪽나무 | 1 | Ⅲ |
| Asteraceae | *Bidens parviflora* Willd. 까치발 | 1 |  |
| Cyperaceae | *Bulbostylis densa* (Wall.) Hand. -Mazz. 꽃하늘지기 | 1 |  |
| Ranunculaceae | *Anemone raddeana* Regel 꿩의바람꽃 | 1 |  |
| Marsileaceae | *Marsilea quadrifolia* L. 네가래 | 3 | Ⅰ |
| Onagraceae | *Ludwigia ovalis* Miq. 눈여뀌바늘 | 6 | Ⅲ |
| Lycopodiaceae | *Huperzia miyoshiana* (Makino) Ching 다람쥐꼬리 | 1 | Ⅱ |
| Liliaceae | *Allium monanthum* Maxim. 달래 | 1 |  |
| Gentianaceae | *Tripterospermum japonicum* (Siebold & Zucc.) Maxim. 덩굴용담 | 1 | VU, Ⅳ |
| Solanaceae | *Solanum carolinense* L. 도깨비가지 | 1 | SC |
| Asteraceae | *Xanthium strumarium* L. 도꼬마리 | 1 | SS |
| Theaceae | *Camellia japonica* L. 동백나무 | 2 | Ⅰ |
| Alismataceae | *Caldesia parnassifolia* (Bassi ex L.) Parl. 둥근잎택사 | 2 | DD, Ⅳ |
| Hydrangeaceae | *Hydrangea petiolaris* Siebold & Zucc. 등수국 | 1 | Ⅲ |
| Iridaceae | *Sisyrinchium rosulatum* E. P. Bicknell 등심붓꽃 | 1 | CS |
| Cyperaceae | *Carex maculata* Boott 무늬사초 | 1 | Ⅰ |
| Asteraceae | *Cirsium rhinoceros* (H. Lév. & Vaniot) Nakai 바늘엉겅퀴 | 4 | EN, ED, Ⅲ |
| Ranunculaceae | *Ranunculus crucilobus* H. Lév. 바위미나리아재비 | 1 | ED, Ⅳ |
| Hydrangeaceae | *Schizophragma hydrangeoides* Siebold & Zucc. 바위수국 | 1 | Ⅳ |
| Lycopodiaceae | *Huperzia serrata* (Thunb.) Trevis. 뱀톱 | 2 |  |
| Ranunculaceae | *Ranunculus natans* C. A. Mey. 북미나리아재비 | 1 |  |
| Lemnaceae | *Wolffia arrhiza* (L.) Horkel ex Wimm. 분개구리밥 | 2 |  |
| Caprifoliaceae | *Viburnum furcatum* Blume ex Maxim. 분단나무 | 1 | Ⅲ |
| Polypodiaceae | *Lepisorus ussuriensis* (Regel & Maack) Ching 산일엽초 | 1 |  |
| Liliaceae | *Tulipa edulis* (Miq.) Baker 산자고 | 1 |  |
| Lycopodiaceae | *Lycopodium clavatum* L. 석송 | 1 |  |
| Apiaceae | *Hydrocotyle maritima* Honda 선피막이 | 1 | Ⅰ |
| Fabaceae | *Maackia fauriei* (H. Lév.) Takeda 솔비나무 | 5 | ED, Ⅳ |
| Apocynaceae | *Cynanchum amplexicaule* (Siebold & Zucc.) Hemsl. 솜아마존 | 5 | EN, Ⅲ |
| Apocynaceae | *Apocynum cannabinum* L. 수궁초 | 1 | DD |
| Nymphaeaceae | *Nymphaea tetragona* Georgi 수련 | 1 |  |
| Oxalidaceae | *Oxalis acetosella* L. 애기괭이밥 | 1 | Ⅲ |
| Menyanthaceae | *Nymphoides indica* (L.) Kuntze 어리연꽃 | 5 |  |
| Myrsinaceae | *Ardisia japonica* (Thunb.) Blume 자금우 | 2 | Ⅰ |
| Ophioglossaceae | *Ophioglossum petiolatum* Hook. 자루나도고사리삼 | 1 | Ⅱ |
| Ophioglossaceae | *Mankyua chejuensis* B.-Y. Sun, M. H. Kim & C. H. Kim 제주고사리삼 | 7 | CR, ED, Ⅴ |
| Poaceae | *Sasa quelpaertensis* Nakai 제주조릿대 | 3 | ED |
| Apiaceae | *Hydrocotyle yabei* Makino, 제주피막이 | 1 | Ⅳ |
| Eriocaulaceae | *Eriocaulon decemflorum* Maxim. 좀개수염 | 2 |  |
| Asteraceae | *Ainsliaea apiculata* Sch. Bip. 좀딱취 | 1 | Ⅰ |
| Asteraceae | *Ixeris stolonifera* A. Gray 좀씀바귀 | 2 |  |
| Menyanthaceae | *Nymphoides coreana* (H. Lév.) H. Hara 좀어리연꽃 | 1 | EN |
| Fagaceae | *Quercus glauca* Thunb. 종가시나무 | 2 | Ⅲ |
| Poaceae | *Lolium multiflorum* Lam. 쥐보리 | 1 | SR |
| Scrophulariaceae | *Microcarpaea minima* (K. D. Koenig ex Retz.) Merr. 진흙풀 | 1 | Ⅱ |
| Thelypteridaceae | *Parathelypteris nipponica* (Franch. & Sav.) Ching 키다리처녀고사리 | 1 |  |
| Rubiaceae | *Neanotis hirsuta* (L.f.) W. H. Lewis 탐라풀 | 1 | Ⅲ |
| Liliaceae | *Allium taquetii* H. Lév. & Vaniot 한라부추 | 3 | ED |
| Poaceae | *Hierochloe odorata* (L.) P. Beauv. 향모 | 1 |  |
| Papaveraceae | *Corydalis remota* Fisch. ex Maxim. 현호색 | 1 |  |
| Violaceae | *Viola philippica* Cav. 호제비꽃 | 1 |  |
| Cyperaceae | *Carex transversa* Boott 화살사초 | 1 |  |
| Araliaceae | *Dendropanax trifidus* (Thunb.) Makino ex H. Hara 황칠나무 | 1 | Ⅲ |
| Violaceae | *Viola lactiflora* Nakai 흰젖제비꽃 | 6 |  |

**^*^Fre: Frequency, RP.: Remarkable plants (Rare plants: CR, EN, VU, LC, DD), ED: Endemic plants, Floristic target plants: Ⅰ~Ⅴ, Invasive alien plants: WS, SS, SR, SC, CS**
