# Supplementary material for: Floristic inventory and distribution characteristics of vascular plants in forest wetlands of South Korea
Source: Biodivers Data J. 2022 Sep 15;10:e85848. doi: 10.3897/BDJ.10.e85848 (PMC9848468; doi:10.3897/BDJ.10.e85848)
Supplement: Supplementary material 18 — The total list of vascular plants of forest wetlands in Korea. [file bdj-10-e85848-s018.docx]

Table 18 . The list of vascular plants of forest wetlands in Korea.

| **Family name** | **Scientific name and Korean name** | **A** | **B** | **C** | **D** | **E** | **F** | **G** | **H** | **I** |
| --- | --- | --- | --- | --- | --- | --- | --- | --- | --- | --- |
| Lycopodiaceae | *Huperzia miyoshiana* (Makino) Ching 다람쥐꼬리 |  | Ⅱ | FACU | 0 | 0 | 0 | 0 | 0 | 1 |
| Lycopodiaceae | *Huperzia serrata* (Thunb.) Trevis. 뱀톱 |  |  | FACW | 0 | 0 | 0 | 0 | 0 | 2 |
| Lycopodiaceae | *Lycopodium clavatum* L. 석송 |  |  | OBW | 0 | 0 | 0 | 0 | 0 | 1 |
| Isoetaceae | *Isoetes sinensis* Palmer 가는물부추 |  |  | OBW | 0 | 0 | 0 | 0 | 0 | 1 |
| Selaginellaceae | *Selaginella tamariscina* (P. Beauv.) Spring 바위손 |  | Ⅲ | OBU | 0 | 0 | 1 | 0 | 0 | 0 |
| Equisetaceae | *Equisetum arvense* L. 쇠뜨기 |  |  | OBU | 71 | 21 | 67 | 37 | 28 | 0 |
| Equisetaceae | *Equisetum hyemale* L. 속새 | ○ | Ⅲ | OBU | 7 | 0 | 0 | 0 | 1 | 0 |
| Equisetaceae | *Equisetum ramosissimum* Desf. 개속새 |  | Ⅱ | FAC | 1 | 0 | 0 | 0 | 0 | 0 |
| Equisetaceae | *Equisetum sylvaticum* L. 능수쇠뜨기 | ○ |  | FACW | 2 | 0 | 0 | 0 | 8 | 0 |
| Ophioglossaceae | *Botrychium ternatum* (Thunb.) Sw. 고사리삼 |  |  | OBU | 8 | 0 | 3 | 3 | 3 | 2 |
| Ophioglossaceae | *Botrychium virginianum* (L.) Sw. 늦고사리삼 |  | Ⅱ | FACU | 1 | 0 | 0 | 0 | 0 | 0 |
| Ophioglossaceae | *Mankyua chejuense* B. Y. Sun, M. H. Kim & C. H. Kim 제주고사리삼 |  | Ⅴ | FACU | 0 | 0 | 0 | 0 | 0 | 7 |
| Ophioglossaceae | *Ophioglossum petiolatum* Hook. 자루나도고사리삼 |  | Ⅱ | OBU | 0 | 0 | 0 | 0 | 0 | 1 |
| Ophioglossaceae | *Ophioglossum vulgatum* L. 나도고사리삼 |  | Ⅲ | FAC | 0 | 0 | 0 | 0 | 1 | 1 |
| Osmundaceae | *Osmunda cinnamomea* L. 꿩고비 |  | Ⅱ | OBU | 32 | 2 | 4 | 1 | 1 | 0 |
| Osmundaceae | *Osmunda claytoniana* L. 음양고비 |  | Ⅲ | FACU | 1 | 2 | 0 | 1 | 0 | 0 |
| Osmundaceae | *Osmunda japonica* Thunb. 고비 |  |  | FAC | 20 | 7 | 9 | 10 | 33 | 0 |
| Gleicheniaceae | *Dicranopteris linearis* (Burm. f.) Underw. 발풀고사리 |  | Ⅲ | OBU | 0 | 0 | 1 | 0 | 0 | 0 |
| Lygodiaceae | *Lygodium japonicum* (Thunb.) Sw. 실고사리 |  | Ⅰ | FAC | 0 | 0 | 0 | 0 | 1 | 0 |
| Marsileaceae | *Marsilea quadrifolia* L. 네가래 |  | Ⅰ | OBW | 0 | 0 | 0 | 0 | 0 | 3 |
| Dennstaedtiaceae | *Dennstaedtia hirsuta* (Sw.) Mett. ex Miq. 잔고사리 |  |  | OBU | 1 | 0 | 1 | 0 | 0 | 0 |
| Dennstaedtiaceae | *Dennstaedtia wilfordii* (T. Moore) Christ 황고사리 |  |  | OBU | 24 | 11 | 11 | 9 | 6 | 0 |
| Dennstaedtiaceae | *Pteridium aquilinum* (L.) Kuhn var. *latiusculum* (Desv.) Underw. ex A. Heller 고사리 |  |  | OBU | 25 | 10 | 19 | 18 | 33 | 3 |
| Pteridaceae | *Coniogramme japonica* (Thunb.) Diels 가지고비고사리 |  | Ⅲ | FACW | 0 | 0 | 0 | 0 | 1 | 0 |
| Aspleniaceae | *Asplenium incisum* Thunb. 꼬리고사리 |  |  | OBU | 6 | 0 | 8 | 3 | 6 | 0 |
| Aspleniaceae | *Asplenium ruprechtii* Sa. Kurata 거미고사리 | ○ |  | FAC | 0 | 0 | 0 | 1 | 0 | 0 |
| Thelypteridaceae | *Metathelypteris laxa* (Franch. & Sav.) Ching 드문고사리 |  | Ⅲ | OBU | 0 | 0 | 0 | 0 | 1 | 0 |
| Thelypteridaceae | *Parathelypteris beddomei* (Baker) Ching 가는잎처녀고사리 |  | Ⅲ | FAC | 0 | 0 | 1 | 0 | 0 | 0 |
| Thelypteridaceae | *Parathelypteris glanduligera* (Kunze) Ching 사다리고사리 |  |  | FAC | 1 | 0 | 0 | 0 | 3 | 0 |
| Thelypteridaceae | *Parathelypteris japonica* (Baker) Ching 지네고사리 |  |  | FAC | 6 | 2 | 4 | 2 | 19 | 0 |
| Thelypteridaceae | *Parathelypteris nipponica* (Franch. & Sav.) Ching 키다리처녀고사리 |  |  | FAC | 0 | 0 | 0 | 0 | 0 | 1 |
| Thelypteridaceae | *Phegopteris connectilis* (Michx.) D. Watt 가래고사리 |  | Ⅱ | OBU | 5 | 0 | 1 | 1 | 1 | 0 |
| Thelypteridaceae | *Phegopteris decursive-pinnata* (H. C. Hall) Fée 설설고사리 |  |  | OBU | 1 | 1 | 0 | 0 | 0 | 0 |
| Thelypteridaceae | *Thelypteris palustris* (A. Gray) Schott 처녀고사리 |  |  | OBU | 74 | 26 | 44 | 21 | 24 | 0 |
| Woodsiaceae | *Woodsia manchuriensis* Hook. 만주우드풀 |  |  | FACU | 1 | 0 | 0 | 0 | 1 | 0 |
| Onocleaceae | *Matteuccia struthiopteris* (L.) Tod. 청나래고사리 | ○ | Ⅱ | OBW | 6 | 0 | 1 | 1 | 0 | 0 |
| Onocleaceae | *Onoclea interrupta* (Maxim.) Ching & P. C. Chiu 야산고비 |  | Ⅰ | OBW | 51 | 22 | 12 | 12 | 3 | 0 |
| Onocleaceae | *Pentarhizidium orientale* (Hook.) Hayata 개면마 |  |  | FACU | 34 | 0 | 3 | 1 | 3 | 0 |
| Athyriaceae | *Athyrium brevifrons* Nakai ex Kitag. 참새발고사리 |  |  | OBU | 24 | 2 | 4 | 5 | 2 | 0 |
| Athyriaceae | *Athyrium niponicum* (Mett.) Hance 개고사리 |  |  | FAC | 25 | 8 | 7 | 9 | 7 | 0 |
| Athyriaceae | *Athyrium vidalii* (Franch. & Sav.) Nakai 산개고사리 |  | Ⅰ | OBU | 3 | 4 | 8 | 0 | 3 | 0 |
| Athyriaceae | *Athyrium yokoscense* (Franch. & Sav.) Christ 뱀고사리 |  |  | FAC | 49 | 15 | 29 | 20 | 22 | 2 |
| Athyriaceae | *Cornopteris crenulatoserrulata* (Makino) Nakai 응달고사리 | ○ |  | OBU | 6 | 0 | 0 | 0 | 0 | 0 |
| Athyriaceae | *Deparia conilii* (Franch. & Sav.) M. Kato 좀진고사리 |  |  | OBU | 13 | 7 | 8 | 7 | 7 | 0 |
| Athyriaceae | *Deparia coreana* (Christ) M. Kato 곱새고사리 |  |  | OBU | 3 | 0 | 0 | 1 | 0 | 0 |
| Athyriaceae | *Deparia japonica* (Thunb.) M. Kato 진고사리 |  |  | OBU | 3 | 1 | 3 | 3 | 5 | 0 |
| Athyriaceae | *Deparia pycnosora* (Christ) M. Kato 털고사리 |  |  | OBU | 7 | 1 | 0 | 0 | 0 | 0 |
| Athyriaceae | *Deparia pycnosora* (Christ) M. Kato var. *albosquamata* M. Kato 흰털고사리 |  |  | FACU | 2 | 1 | 0 | 0 | 0 | 0 |
| Dryopteridaceae | *Arachniodes borealis* Seriz. 왁살고사리 |  |  | FACW | 3 | 0 | 0 | 0 | 0 | 0 |
| Dryopteridaceae | *Cyrtomium fortunei* J. Sm. 쇠고비 |  |  | OBU | 0 | 0 | 2 | 0 | 2 | 0 |
| Dryopteridaceae | *Dryopsis maximowicziana* (Miq.) C. Chr. 흰비늘고사리 |  | Ⅴ | FAC | 0 | 0 | 0 | 0 | 1 | 0 |
| Dryopteridaceae | *Dryopteris bissetiana* (Baker) C. Chr. 산족제비고사리 |  |  | OBU | 1 | 0 | 3 | 1 | 1 | 0 |
| Dryopteridaceae | *Dryopteris chinensis* (Baker) Koidz. 가는잎족제비고사리 |  |  | OBU | 15 | 6 | 11 | 6 | 3 | 0 |
| Dryopteridaceae | *Dryopteris crassirhizoma* Nakai 관중 | ○ |  | FAC | 54 | 17 | 2 | 0 | 2 | 0 |
| Dryopteridaceae | *Dryopteris erythrosora* (D. C. Eaton) Kuntze 홍지네고사리 |  | Ⅰ | OBU | 1 | 0 | 0 | 0 | 0 | 0 |
| Dryopteridaceae | *Dryopteris expansa* (C. Presl) Fraser-Jenk. & Jermy 퍼진고사리 | ○ | Ⅱ | OBU | 3 | 0 | 0 | 0 | 1 | 0 |
| Dryopteridaceae | *Dryopteris kinkiensis* Koidz. ex Tagawa 엷은잎지네고사리 |  |  | OBU | 0 | 0 | 0 | 0 | 1 | 0 |
| Dryopteridaceae | *Dryopteris lacera* (Thunb.) Kuntze 비늘고사리 |  |  | FACW | 4 | 1 | 3 | 2 | 3 | 0 |
| Dryopteridaceae | *Dryopteris monticola* (Makino) C. Chr. 왕지네고사리 |  |  | OBU | 1 | 0 | 2 | 0 | 0 | 0 |
| Dryopteridaceae | *Dryopteris polylepis* (Franch. & Sav.) C. Chr. 산비늘고사리 |  |  | OBW | 0 | 0 | 1 | 0 | 0 | 0 |
| Dryopteridaceae | *Dryopteris sacrosancta* Koidz. 애기족제비고사리 |  |  | OBU | 0 | 0 | 0 | 2 | 0 | 0 |
| Dryopteridaceae | *Dryopteris saxifragivaria* Nakai 광릉족제비고사리 |  |  | FACU | 0 | 0 | 0 | 0 | 0 | 1 |
| Dryopteridaceae | *Dryopteris tokyoensis* (Matsum. ex Makino) C. Chr. 느리미고사리 |  | Ⅳ | OBU | 1 | 1 | 0 | 0 | 1 | 0 |
| Dryopteridaceae | *Dryopteris uniformis* (Makino) Makino 곰비늘고사리 |  |  | FAC | 1 | 0 | 0 | 0 | 0 | 1 |
| Dryopteridaceae | *Dryopteris varia* (L.) Kuntze 족제비고사리 |  |  | OBU | 3 | 3 | 1 | 1 | 7 | 0 |
| Dryopteridaceae | *Polystichum braunii* (Spenn.) Fée 좀나도히초미 |  | Ⅱ | FACU | 1 | 0 | 0 | 0 | 1 | 0 |
| Dryopteridaceae | *Polystichum ovatopaleaceum* (Kodama) Sa. Kurata  var. *coraiense* (Christ) Sa. Kurata 참나도히초미 |  | Ⅰ | OBU | 5 | 3 | 0 | 1 | 0 | 0 |
| Dryopteridaceae | *Polystichum polyblepharum* (Roem. ex Kunze) C. Presl 나도히초미 |  | Ⅰ | OBU | 1 | 0 | 0 | 0 | 0 | 1 |
| Dryopteridaceae | *Polystichum tripteron* (Kunze) C. Presl 십자고사리 |  |  | OBU | 14 | 3 | 1 | 1 | 4 | 0 |
| Nephrolepidaceae | *Nephrolepis cordifolia* (L.) C. Presl 줄고사리 |  | Ⅴ | OBU | 0 | 0 | 0 | 0 | 1 | 0 |
| Polypodiaceae | *Lepisorus thunbergianus* (Kaulf.) Ching 일엽초 |  | Ⅰ | OBU | 1 | 0 | 0 | 0 | 1 | 0 |
| Polypodiaceae | *Lepisorus ussuriensis* (Regel & Maack) Ching 산일엽초 |  |  | OBU | 0 | 0 | 0 | 0 | 0 | 1 |
| Ginkgoaceae | *Ginkgo biloba* L. 은행나무 |  |  | FACW | 2 | 1 | 1 | 0 | 0 | 0 |
| Pinaceae | *Abies holophylla* Maxim. 전나무 | ○ |  | OBU | 7 | 3 | 0 | 0 | 1 | 0 |
| Pinaceae | *Abies koreana* E. H. Wilson 구상나무 |  | Ⅲ | FACW | 1 | 0 | 1 | 0 | 0 | 0 |
| Pinaceae | *Larix kaempferi* (Lamb.) Carrière 일본잎갈나무 |  |  | FAC | 28 | 7 | 4 | 7 | 3 | 0 |
| Pinaceae | *Pinus densiflora* Siebold & Zucc. 소나무 | ○ |  | OBU | 49 | 8 | 40 | 12 | 27 | 1 |
| Pinaceae | *Pinus koraiensis* Siebold & Zucc. 잣나무 | ○ | Ⅱ | OBU | 14 | 8 | 3 | 2 | 6 | 0 |
| Pinaceae | *Pinus rigida* Mill. 리기다소나무 |  |  | OBU | 4 | 2 | 4 | 3 | 16 | 0 |
| Pinaceae | *Pinus thunbergii* Parl. 곰솔 |  |  | OBU | 0 | 1 | 5 | 0 | 11 | 1 |
| Cupressaceae | *Chamaecyparis obtusa* (Siebold & Zucc.) Endl. 편백 |  |  | OBU | 0 | 0 | 2 | 0 | 5 | 0 |
| Cupressaceae | *Cryptomeria japonica* (Thunb. ex L. f.) D. Don 삼나무 |  |  | OBU | 0 | 0 | 0 | 0 | 9 | 3 |
| Cupressaceae | *Juniperus rigida* Siebold & Zucc. 노간주나무 |  |  | OBU | 2 | 0 | 4 | 3 | 13 | 0 |
| Cupressaceae | *Metasequoia glyptostroboides* Hu & W. C. Cheng 메타세쿼이아 |  |  | FAC | 0 | 0 | 0 | 1 | 0 | 0 |
| Cephalotaxaceae | *Cephalotaxus harringtonii* (Knight ex J. Forbes) K. Koch 개비자나무 |  | Ⅰ | OBU | 0 | 0 | 0 | 0 | 1 | 0 |
| Taxaceae | *Taxus cuspidata* Siebold & Zucc. 주목 | ○ | Ⅱ | OBU | 1 | 0 | 0 | 0 | 0 | 0 |
| Juglandaceae | *Juglans mandshurica* Maxim. 가래나무 |  | Ⅰ | OBU | 18 | 1 | 3 | 2 | 5 | 0 |
| Juglandaceae | *Juglans regia* L. 호두나무 |  |  | FACU | 0 | 0 | 0 | 0 | 1 | 0 |
| Juglandaceae | *Platycarya strobilacea* Siebold & Zucc. 굴피나무 |  |  | FACW | 2 | 4 | 18 | 10 | 13 | 0 |
| Salicaceae | *Populus deltoides* Marsh. 미루나무 |  |  | OBU | 0 | 0 | 1 | 1 | 0 | 0 |
| Salicaceae | *Populus nigra* L. 양버들 |  |  | OBU | 0 | 0 | 2 | 0 | 0 | 0 |
| Salicaceae | *Populus suaveolens* Fisch. ex Loudon 황철나무 |  | Ⅳ | OBU | 1 | 0 | 0 | 0 | 0 | 0 |
| Salicaceae | *Populus tremula* L. var. *davidiana* (Dode) C. K. Schneid. 사시나무 |  |  | FACU | 3 | 0 | 0 | 0 | 1 | 0 |
| Salicaceae | *Populus* ⨉ *anadensis* Moench 이태리포플라 |  |  | OBU | 2 | 1 | 5 | 1 | 3 | 0 |
| Salicaceae | *Populus* ⨉ *tomentiglandulosa* T. B. Lee ex M. Kim 은사시나무 |  |  | OBU | 5 | 3 | 6 | 4 | 5 | 0 |
| Salicaceae | *Salix babylonica* L. 수양버들 |  |  | FAC | 2 | 0 | 0 | 0 | 0 | 0 |
| Salicaceae | *Salix caprea* L. 호랑버들 |  |  | FACU | 13 | 1 | 3 | 6 | 2 | 0 |
| Salicaceae | *Salix chaenomeloides* Kimura 왕버들 |  | Ⅰ | FACW | 4 | 3 | 33 | 14 | 15 | 0 |
| Salicaceae | *Salix gracilistyla* Miq. 갯버들 | ○ |  | OBW | 29 | 11 | 16 | 14 | 24 | 0 |
| Salicaceae | *Salix hallaisanensis* H. Lév. 떡버들 |  |  | OBU | 1 | 0 | 1 | 1 | 0 | 0 |
| Salicaceae | *Salix integra* Thunb. 개키버들 | ○ |  | FACW | 3 | 0 | 3 | 0 | 1 | 0 |
| Salicaceae | *Salix koriyanagi* Kimura ex Goerz 키버들 |  |  | OBU | 38 | 15 | 34 | 10 | 14 | 0 |
| Salicaceae | *Salix pierotii* Miq. 버드나무 |  |  | FAC | 103 | 34 | 79 | 40 | 53 | 2 |
| Salicaceae | *Salix pseudolasiogyne* H. Lév. 능수버들 |  |  | FACW | 0 | 0 | 1 | 0 | 0 | 0 |
| Salicaceae | *Salix rorida* Laksch. 분버들 | ○ | Ⅲ | FACW | 8 | 0 | 0 | 0 | 0 | 0 |
| Salicaceae | *Salix siuzevii* Seem. 참오글잎버들 |  |  | OBU | 3 | 0 | 0 | 0 | 0 | 0 |
| Salicaceae | *Salix triandra* L. subsp. *nipponica* (Franch. & Sav.) A. K. Skvortsov 선버들 |  |  | FACU | 8 | 1 | 4 | 7 | 7 | 0 |
| Salicaceae | *Salix xerophila* Flod. 여우버들 |  | Ⅲ | FACU | 1 | 0 | 0 | 0 | 0 | 0 |
| Betulaceae | *Alnus firma* Siebold & Zucc. 사방오리 |  |  | FACW | 2 | 1 | 3 | 2 | 4 | 0 |
| Betulaceae | *Alnus incana* (L.) Moench subsp. *hirsuta* (Turcz. ex Spach) Á. Löve & D. Löve 물오리나무 | ○ |  | OBW | 44 | 11 | 52 | 15 | 17 | 0 |
| Betulaceae | *Alnus japonica* (Thunb.) Steud. 오리나무 |  | Ⅱ | FACU | 18 | 12 | 23 | 9 | 22 | 0 |
| Betulaceae | *Betula costata* Trautv. 거제수나무 | ○ | Ⅲ | FACU | 6 | 1 | 0 | 0 | 0 | 0 |
| Betulaceae | *Betula davurica* Pall. 물박달나무 | ○ | Ⅲ | FACW | 21 | 1 | 6 | 1 | 0 | 0 |
| Betulaceae | *Betula ermanii* Cham. 사스래나무 | ○ | Ⅱ | OBU | 3 | 1 | 1 | 0 | 0 | 0 |
| Betulaceae | *Betula pendula* Roth 자작나무 | ○ |  | OBU | 13 | 0 | 0 | 3 | 0 | 0 |
| Betulaceae | *Betula schmidtii* Regel 박달나무 | ○ | Ⅲ | FAC | 4 | 1 | 1 | 0 | 1 | 0 |
| Betulaceae | *Carpinus cordata* Blume 까치박달 |  |  | FAC | 19 | 0 | 0 | 0 | 0 | 0 |
| Betulaceae | *Carpinus laxiflora* (Siebold & Zucc.) Blume 서어나무 |  |  | OBU | 4 | 1 | 7 | 0 | 7 | 1 |
| Betulaceae | *Carpinus tschonoskii* Maxim. 개서어나무 |  | Ⅰ | OBU | 0 | 0 | 1 | 0 | 3 | 1 |
| Betulaceae | *Carpinus turczaninovii* Hance 소사나무 |  | Ⅰ | OBU | 1 | 0 | 0 | 0 | 0 | 0 |
| Betulaceae | *Corylus heterophylla* Fisch. ex Trautv. 개암나무 |  |  | OBU | 19 | 9 | 4 | 6 | 9 | 0 |
| Betulaceae | *Corylus sieboldiana* Blume 참개암나무 | ○ |  | OBW | 14 | 2 | 1 | 6 | 6 | 0 |
| Betulaceae | *Corylus sieboldiana* Blume var. *mandshurica* (Maxim.) C. K. Schneid. 물개암나무 |  |  | OBW | 4 | 0 | 0 | 1 | 2 | 0 |
| Fagaceae | *Castanea crenata* Siebold & Zucc. 밤나무 |  |  | OBU | 37 | 13 | 34 | 19 | 35 | 0 |
| Fagaceae | *Quercus acutissima* Carruth. 상수리나무 |  |  | FACU | 3 | 5 | 17 | 13 | 23 | 0 |
| Fagaceae | *Quercus aliena* Blume 갈참나무 |  |  | OBU | 13 | 9 | 29 | 16 | 20 | 0 |
| Fagaceae | *Quercus dentata* Thunb. 떡갈나무 |  |  | FACW | 9 | 3 | 5 | 1 | 4 | 0 |
| Fagaceae | *Quercus glauca* Thunb. 종가시나무 |  | Ⅲ | FAC | 0 | 0 | 0 | 0 | 0 | 2 |
| Fagaceae | *Quercus mongolica* Fisch. ex Ledeb. 신갈나무 |  |  | FAC | 60 | 4 | 15 | 8 | 9 | 2 |
| Fagaceae | *Quercus mongolica* Fisch. ex Ledeb. var. *crispula* (Blume) H. Ohashi 물참나무 |  |  | FACU | 0 | 0 | 2 | 0 | 0 | 0 |
| Fagaceae | *Quercus serrata* Murray 졸참나무 |  |  | OBW | 36 | 7 | 34 | 15 | 40 | 1 |
| Fagaceae | *Quercus variabilis* Blume 굴참나무 |  |  | OBU | 13 | 7 | 11 | 7 | 13 | 0 |
| Fagaceae | *Quercus* ⨉ *urticifolia* Blume 갈졸참나무 |  |  | OBU | 2 | 0 | 1 | 0 | 0 | 0 |
| Eucommiaceae | *Eucommia ulmoides* Oliv. 두충 |  |  | OBU | 0 | 0 | 1 | 0 | 0 | 0 |
| Ulmaceae | *Aphananthe aspera* (Thunb.) Planch. 푸조나무 |  | Ⅰ | OBU | 0 | 0 | 0 | 0 | 1 | 0 |
| Ulmaceae | *Celtis aurantiaca* Nakai 산팽나무 |  |  | OBU | 0 | 0 | 0 | 1 | 0 | 0 |
| Ulmaceae | *Celtis bungeana* Blume 좀풍게나무 |  |  | FACW | 1 | 1 | 0 | 0 | 0 | 0 |
| Ulmaceae | *Celtis choseniana* Nakai 검팽나무 |  | Ⅰ | OBU | 0 | 0 | 1 | 0 | 1 | 0 |
| Ulmaceae | *Celtis jessoensis* Koidz. 풍게나무 |  |  | OBU | 0 | 0 | 2 | 1 | 0 | 0 |
| Ulmaceae | *Celtis sinensis* Pers. 팽나무 |  |  | OBU | 0 | 0 | 18 | 4 | 15 | 0 |
| Ulmaceae | *Hemiptelea davidii* (Hance) Planch. 시무나무 |  | Ⅰ | OBU | 1 | 0 | 1 | 0 | 0 | 0 |
| Ulmaceae | *Ulmus davidiana* Planch. ex DC. 당느릅나무 | ○ | Ⅰ | OBU | 0 | 0 | 1 | 0 | 0 | 0 |
| Ulmaceae | *Ulmus davidiana* Planch. ex DC. var. *japonica* (Rehder) Nakai 느릅나무 |  | Ⅰ | FACU | 35 | 5 | 17 | 6 | 2 | 0 |
| Ulmaceae | *Ulmus laciniata* (Trautv.) Mayr 난티나무 | ○ | Ⅲ | OBW | 2 | 0 | 0 | 0 | 0 | 0 |
| Ulmaceae | *Ulmus macrocarpa* Hance 왕느릅나무 | ○ | Ⅳ | FACU | 1 | 0 | 0 | 0 | 0 | 0 |
| Ulmaceae | *Ulmus parvifolia* Jacq. 참느릅나무 |  | Ⅰ | OBW | 1 | 2 | 10 | 4 | 5 | 6 |
| Ulmaceae | *Zelkova serrata* (Thunb.) Makino 느티나무 |  |  | FACW | 3 | 2 | 8 | 3 | 6 | 0 |
| Moraceae | *Broussonetia papyrifera* (L.) L’ Hér. ex Vent. 꾸지나무 |  |  | OBU | 0 | 0 | 0 | 1 | 0 | 0 |
| Moraceae | *Broussonetia* ⨉ *hanjiana* M. Kim 닥나무 |  |  | OBU | 0 | 1 | 0 | 0 | 1 | 0 |
| Moraceae | *Cudrania tricuspidata* (Carrière) Bureau ex Lavallée 꾸지뽕나무 |  |  | FACU | 0 | 0 | 1 | 0 | 5 | 6 |
| Moraceae | *Fatoua villosa* (Thunb.) Nakai 뽕모시풀 |  |  | OBU | 1 | 0 | 3 | 1 | 0 | 0 |
| Moraceae | *Morus alba* L. 뽕나무 |  |  | OBU | 15 | 4 | 12 | 2 | 7 | 0 |
| Moraceae | *Morus australis* Poir. 산뽕나무 |  |  | OBU | 49 | 20 | 21 | 21 | 22 | 0 |
| Moraceae | *Morus cathayana* Hemsl. 돌뽕나무 |  | Ⅲ | FACU | 0 | 0 | 0 | 1 | 0 | 0 |
| Cannabaceae | *Humulus scandens* (Lour.) Merr. 환삼덩굴 |  |  | OBU | 36 | 14 | 42 | 23 | 19 | 1 |
| Urticaceae | *Achudemia japonica* Maxim. 산물통이 |  | Ⅰ | OBU | 7 | 2 | 4 | 0 | 0 | 0 |
| Urticaceae | *Boehmeria japonica* (L.f.) Miq. 왜모시풀 |  |  | FAC | 1 | 0 | 2 | 2 | 4 | 0 |
| Urticaceae | *Boehmeria nivea* (L.) Gaudich. 모시풀 |  |  | OBU | 1 | 1 | 3 | 1 | 8 | 0 |
| Urticaceae | *Boehmeria pannosa* Nakai & Satake ex Oka 왕모시풀 |  | Ⅲ | FACU | 0 | 0 | 0 | 0 | 1 | 0 |
| Urticaceae | *Boehmeria platanifolia* (Franch. & Sav.) C. H. Wright 개모시풀 |  |  | FACU | 5 | 4 | 17 | 9 | 7 | 1 |
| Urticaceae | *Boehmeria spicata* (Thunb.) Thunb. 좀깨잎나무 |  |  | OBU | 21 | 8 | 43 | 16 | 29 | 0 |
| Urticaceae | *Boehmeria tricuspis* (Hance) Makino 거북꼬리 |  |  | FACW | 7 | 10 | 1 | 8 | 15 | 0 |
| Urticaceae | *Boehmeria tricuspis* (Hance) Makino var. *unicuspis* Makino ex Ohwi 풀거북꼬리 |  |  | OBU | 4 | 1 | 4 | 2 | 7 | 0 |
| Urticaceae | *Laportea bulbifera* (Siebold & Zucc.) Wedd. 혹쐐기풀 |  |  | OBU | 6 | 0 | 0 | 0 | 1 | 0 |
| Urticaceae | *Pilea oligantha* Nakai 강계큰물통이 |  |  | FAC | 2 | 0 | 0 | 1 | 0 | 0 |
| Urticaceae | *Pilea peploides* (Gaudich.) Hook. & Arn. 물통이 |  |  | FACU | 15 | 13 | 8 | 1 | 5 | 0 |
| Urticaceae | *Pilea pumila* (L.) A. Gray 모시물통이 |  |  | FACU | 53 | 13 | 37 | 23 | 13 | 0 |
| Urticaceae | *Pilea pumila* (L.) A. Gray var. *hamaoi* (Makino) C. J. Chen 큰물통이 |  |  | FAC | 7 | 0 | 6 | 0 | 1 | 0 |
| Urticaceae | *Urtica angustifolia* Fisch. ex Hornem. 가는잎쐐기풀 | ○ | Ⅲ | FACU | 1 | 0 | 0 | 0 | 0 | 0 |
| Urticaceae | *Urtica laetevirens* Maxim. 애기쐐기풀 | ○ |  | FACU | 1 | 0 | 1 | 0 | 0 | 0 |
| Urticaceae | *Urtica thunbergiana* Siebold & Zucc. 쐐기풀 |  | Ⅲ | OBW | 2 | 0 | 0 | 1 | 2 | 0 |
| Santalaceae | *Thesium chinense* Turcz. 제비꿀 |  |  | OBU | 2 | 0 | 2 | 0 | 1 | 0 |
| Polygonaceae | *Aconogonon alpinum* (All.) Schur 싱아 |  |  | FAC | 0 | 0 | 0 | 2 | 2 | 0 |
| Polygonaceae | *Aconogonon divaricatum* (L.) Nakai ex T. Mori 왜개싱아 |  |  | OBU | 1 | 0 | 0 | 0 | 0 | 0 |
| Polygonaceae | *Aconogonon microcarpum* (Kitag.) H. Hara 참개싱아 |  | Ⅳ | OBU | 1 | 0 | 0 | 0 | 0 | 0 |
| Polygonaceae | *Bistorta incana* (Nakai) Nakai ex T. Mori 흰범꼬리 |  |  | FACW | 1 | 0 | 0 | 0 | 0 | 0 |
| Polygonaceae | *Bistorta officinalis* Delarbre subsp. *japonica* (H. Hara) Yonek. 범꼬리 |  | Ⅱ | OBU | 2 | 0 | 0 | 1 | 0 | 0 |
| Polygonaceae | *Fallopia ciliinervis* (Nakai) K. Hammer 나도하수오 |  |  | OBU | 1 | 0 | 0 | 0 | 0 | 0 |
| Polygonaceae | *Fallopia dentatoalata* (F. Schmidt) Holub 큰닭의덩굴 |  |  | FACU | 3 | 0 | 1 | 1 | 0 | 0 |
| Polygonaceae | *Fallopia dumetorum* (L.) Holub 닭의덩굴 |  |  | FACU | 9 | 2 | 5 | 3 | 4 | 0 |
| Polygonaceae | *Fallopia forbesii* (Hance) Yonek. & H. Ohashi 감절대 |  |  | OBU | 0 | 0 | 0 | 1 | 0 | 0 |
| Polygonaceae | *Fallopia japonica* (Houtt.) Ronse Decr. 호장근 |  |  | OBU | 0 | 0 | 1 | 2 | 0 | 1 |
| Polygonaceae | *Persicaria breviochreata* (Makino) Ohki 긴화살여뀌 |  | Ⅰ | OBU | 0 | 0 | 0 | 0 | 1 | 0 |
| Polygonaceae | *Persicaria dissitiflora* (Hemsl.) H. Gross ex T. Mor 가시여뀌 |  |  | OBU | 0 | 0 | 1 | 1 | 2 | 0 |
| Polygonaceae | *Persicaria erectominor* (Makino) Nakai var. *koreensis* (Nakai) I. Ito 대동여뀌 |  | Ⅲ | OBU | 0 | 0 | 2 | 0 | 0 | 0 |
| Polygonaceae | *Persicaria filiformis* (Thunb.) Nakai ex T. Mori 이삭여뀌 |  |  | FACU | 19 | 19 | 20 | 23 | 36 | 0 |
| Polygonaceae | *Persicaria hastatosagittata* (Makino) Nakai ex T. Mori 긴미꾸리낚시 |  |  | FAC | 0 | 0 | 3 | 0 | 1 | 0 |
| Polygonaceae | *Persicaria hydropiper* (L.) Delarbre 여뀌 |  |  | OBU | 21 | 14 | 26 | 3 | 13 | 2 |
| Polygonaceae | *Persicaria japonica* (Meisn.) Nakai 흰꽃여뀌 |  |  | FACW | 0 | 0 | 6 | 0 | 0 | 7 |
| Polygonaceae | *Persicaria lapathifolia* (L.) Delarbre 흰여뀌 |  |  | FACW | 9 | 7 | 7 | 2 | 4 | 2 |
| Polygonaceae | *Persicaria longiseta* (Bruijn) Kitag. 개여뀌 |  |  | OBU | 19 | 12 | 25 | 18 | 11 | 0 |
| Polygonaceae | *Persicaria maackiana* (Regel) Nakai ex T. Mori 나도미꾸리낚시 |  |  | FACU | 1 | 0 | 3 | 1 | 1 | 0 |
| Polygonaceae | *Persicaria muricata* (Meisn.) Nemoto 넓은잎미꾸리낚시 |  |  | OBU | 20 | 12 | 20 | 11 | 21 | 1 |
| Polygonaceae | *Persicaria nepalensis* (Meisn.) H. Gross 산여뀌 |  |  | FACU | 6 | 2 | 1 | 2 | 3 | 0 |
| Polygonaceae | *Persicaria orientalis* (L.) Spach 털여뀌 |  |  | FACU | 0 | 0 | 1 | 0 | 0 | 1 |
| Polygonaceae | *Persicaria perfoliata* (L.) H. Gross 며느리배꼽 |  |  | FAC | 12 | 9 | 20 | 13 | 11 | 0 |
| Polygonaceae | *Persicaria posumbu* (Buch.-Ham. ex D. Don) H. Gross 장대여뀌 |  |  | FAC | 2 | 1 | 2 | 5 | 1 | 0 |
| Polygonaceae | *Persicaria praetermissa* (Hook. f.) H. Hara 좁은잎미꾸리낚시 |  |  | OBU | 0 | 1 | 3 | 0 | 0 | 15 |
| Polygonaceae | *Persicaria pubescens* (Blume) H. Hara 바보여뀌 |  |  | OBU | 6 | 3 | 5 | 2 | 4 | 0 |
| Polygonaceae | *Persicaria sagittata* (L.) H. Gross 미꾸리낚시 |  |  | OBW | 60 | 17 | 62 | 18 | 22 | 7 |
| Polygonaceae | *Persicaria senticosa* (Meisn.) H. Gross ex Nakai 며느리밑씻개 |  |  | FACU | 23 | 8 | 30 | 11 | 7 | 0 |
| Polygonaceae | *Persicaria taquetii* (H. Lév.) Koidz. 겨이삭여뀌 |  | Ⅳ | OBW | 0 | 0 | 0 | 0 | 0 | 1 |
| Polygonaceae | *Persicaria thunbergii* (Siebold & Zucc.) H. Gross 고마리 |  |  | OBW | 114 | 29 | 79 | 40 | 49 | 4 |
| Polygonaceae | *Persicaria viscofera* (Makino) H. Gross 끈끈이여뀌 |  |  | OBW | 2 | 0 | 0 | 0 | 1 | 0 |
| Polygonaceae | *Polygonum aviculare* L. 마디풀 |  |  | OBW | 1 | 0 | 0 | 0 | 1 | 0 |
| Polygonaceae | *Rumex acetosa* L. 수영 |  |  | OBU | 4 | 0 | 1 | 2 | 0 | 0 |
| Polygonaceae | *Rumex acetosella* L. 애기수영 |  |  | FAC | 2 | 0 | 4 | 0 | 0 | 1 |
| Polygonaceae | *Rumex crispus* L. 소리쟁이 |  |  | OBU | 0 | 1 | 3 | 2 | 3 | 0 |
| Polygonaceae | *Rumex longifolius* DC. 개대황 |  |  | OBU | 0 | 0 | 0 | 1 | 0 | 0 |
| Polygonaceae | *Rumex obtusifolius* L. 돌소리쟁이 |  |  | OBU | 0 | 0 | 1 | 1 | 0 | 0 |
| Phytolaccaceae | *Phytolacca acinosa* Roxb. 자리공 |  | Ⅳ | OBU | 0 | 0 | 1 | 2 | 0 | 0 |
| Phytolaccaceae | *Phytolacca americana* L. 미국자리공 |  |  | FACW | 10 | 10 | 14 | 10 | 17 | 0 |
| Molluginaceae | *Mollugo pentaphylla* L. 석류풀 |  |  | FAC | 0 | 1 | 0 | 1 | 0 | 0 |
| Portulacaceae | *Portulaca oleracea* L. 쇠비름 |  |  | OBU | 1 | 0 | 0 | 0 | 0 | 0 |
| Caryophyllaceae | *Arenaria serpyllifolia* L. 벼룩이자리 |  |  | OBU | 2 | 0 | 0 | 2 | 0 | 1 |
| Caryophyllaceae | *Cerastium glomeratum* Thuill. 유럽점나도나물 |  |  | OBU | 2 | 0 | 0 | 0 | 0 | 0 |
| Caryophyllaceae | *Cerastium holosteoides* Fr. subsp. *vulgare* (Hartm.) I. V. Sokolova 점나도나물 |  |  | FAC | 6 | 2 | 1 | 2 | 1 | 1 |
| Caryophyllaceae | *Dianthus chinensis* L. 패랭이꽃 |  |  | OBU | 0 | 0 | 2 | 0 | 0 | 1 |
| Caryophyllaceae | *Dianthus longicalyx* Miq. 술패랭이꽃 |  |  | OBU | 0 | 0 | 0 | 0 | 1 | 0 |
| Caryophyllaceae | *Lychnis cognata* Maxim. 동자꽃 | ○ | Ⅱ | OBU | 17 | 1 | 2 | 1 | 0 | 0 |
| Caryophyllaceae | *Pseudostellaria davidii* (Franch.) Pax ex Pax & Hoffm. 덩굴개별꽃 | ○ |  | FAC | 3 | 0 | 1 | 1 | 0 | 0 |
| Caryophyllaceae | *Pseudostellaria heterophylla* (Miq.) Pax 개별꽃 |  |  | OBU | 12 | 0 | 4 | 4 | 2 | 1 |
| Caryophyllaceae | *Pseudostellaria palibiniana* (Takeda) Ohwi 큰개별꽃 |  |  | OBU | 14 | 1 | 2 | 2 | 0 | 1 |
| Caryophyllaceae | *Pseudostellaria setulosa* Ohwi 숲개별꽃 |  | Ⅳ | FACU | 9 | 1 | 0 | 0 | 0 | 0 |
| Caryophyllaceae | *Silene baccifera* (L.) Roth 덩굴별꽃 |  | Ⅰ | OBW | 2 | 0 | 0 | 1 | 0 | 0 |
| Caryophyllaceae | *Silene firma* Siebold & Zucc. 장구채 |  |  | OBU | 5 | 2 | 6 | 2 | 1 | 0 |
| Caryophyllaceae | *Silene firma* Siebold & Zucc. f. *pubescens* (Makino) Ohwi & H. Ohashi 털장구채 |  |  | OBU | 0 | 0 | 0 | 1 | 0 | 0 |
| Caryophyllaceae | *Stellaria aquatica* (L.) Scop. 쇠별꽃 |  |  | OBU | 13 | 3 | 7 | 5 | 2 | 0 |
| Caryophyllaceae | *Stellaria media* (L.) Vill. 별꽃 |  |  | FACU | 0 | 0 | 1 | 3 | 2 | 0 |
| Caryophyllaceae | *Stellaria uliginosa* Murray 벼룩나물 |  |  | FACU | 7 | 2 | 2 | 2 | 2 | 0 |
| Chenopodiaceae | *Chenopodium album* L. 흰명아주 |  |  | FACW | 4 | 0 | 2 | 0 | 0 | 0 |
| Chenopodiaceae | *Chenopodium album* L. var. *centrorubrum* Makino 명아주 |  |  | OBU | 0 | 0 | 3 | 2 | 0 | 0 |
| Chenopodiaceae | *Chenopodium ficifolium* Sm. 좀명아주 |  |  | FAC | 2 | 0 | 3 | 0 | 0 | 0 |
| Chenopodiaceae | *Suaeda maritima* (L.) Dumort. 해홍나물 |  | Ⅱ | OBU | 0 | 0 | 0 | 0 | 1 | 0 |
| Amaranthaceae | *Achyranthes bidentata* Blume 털쇠무릎 |  |  | FACU | 7 | 0 | 0 | 2 | 0 | 0 |
| Amaranthaceae | *Achyranthes bidentata* Blume var. *japonica* Miq. 쇠무릎 |  |  | FACW | 32 | 13 | 34 | 22 | 31 | 0 |
| Amaranthaceae | *Amaranthus blitum* L. subsp. *oleraceus* (L.) Costea 개비름 |  |  | OBU | 0 | 1 | 1 | 0 | 0 | 0 |
| Magnoliaceae | *Liriodendron tulipifera* L. 백합나무 |  |  | OBU | 0 | 0 | 1 | 1 | 2 | 0 |
| Magnoliaceae | *Magnolia denudata* Desr. 백목련 |  |  | OBU | 1 | 0 | 0 | 0 | 0 | 0 |
| Magnoliaceae | *Magnolia kobus* DC. 목련 |  | Ⅴ | OBU | 1 | 0 | 0 | 0 | 1 | 0 |
| Magnoliaceae | *Magnolia obovata* Thunb. 일본목련 |  |  | OBU | 0 | 2 | 0 | 0 | 0 | 0 |
| Magnoliaceae | *Magnolia sieboldii* K. Koch 함박꽃나무 |  | Ⅱ | OBU | 19 | 1 | 3 | 3 | 2 | 0 |
| Schisandraceae | *Schisandra chinensis* (Turcz.) Baill. 오미자 | ○ | Ⅱ | OBW | 20 | 3 | 2 | 4 | 2 | 0 |
| Lauraceae | *Lindera erythrocarpa* Makino 비목나무 |  | Ⅰ | FAC | 1 | 1 | 28 | 17 | 39 | 2 |
| Lauraceae | *Lindera glauca* (Siebold & Zucc.) Blume 감태나무 |  | Ⅰ | OBU | 0 | 0 | 20 | 2 | 26 | 0 |
| Lauraceae | *Lindera obtusiloba* Blume 생강나무 |  |  | OBU | 63 | 16 | 42 | 17 | 18 | 2 |
| Lauraceae | *Lindera sericea* (Siebold & Zucc.) Blume 털조장나무 |  | Ⅳ | OBU | 0 | 0 | 0 | 0 | 1 | 0 |
| Lauraceae | *Litsea japonica* (Thunb.) Juss. 까마귀쪽나무 |  | Ⅲ | FACU | 0 | 0 | 0 | 0 | 0 | 1 |
| Ranunculaceae | *Aconitum ciliare* DC. 놋젓가락나물 |  |  | OBU | 3 | 0 | 0 | 0 | 1 | 0 |
| Ranunculaceae | *Aconitum jaluense* Kom. 투구꽃 |  | Ⅰ | OBU | 21 | 3 | 4 | 2 | 3 | 0 |
| Ranunculaceae | *Aconitum longecassidatum* Nakai 흰진범 |  | Ⅰ | FACW | 5 | 1 | 0 | 1 | 0 | 0 |
| Ranunculaceae | *Aconitum pseudolaeve* Nakai 진범 |  |  | FACW | 2 | 1 | 0 | 1 | 0 | 0 |
| Ranunculaceae | *Actaea asiatica* H. Hara 노루삼 |  | Ⅲ | OBU | 6 | 0 | 0 | 2 | 1 | 0 |
| Ranunculaceae | *Megaleranthis saniculifolia* Ohwi 모데미풀 |  | Ⅲ | OBU | 1 | 0 | 0 | 0 | 0 | 0 |
| Ranunculaceae | *Actaea biternata* (Siebold & Zucc.) Prantl 개승마 |  |  | OBU | 1 | 3 | 0 | 0 | 0 | 0 |
| Ranunculaceae | *Actaea dahurica* (Turcz. ex Fisch. & C. A. Mey.) Franch. 눈빛승마 |  |  | OBW | 11 | 0 | 3 | 1 | 0 | 0 |
| Ranunculaceae | *Actaea heracleifolia* (Kom.) J. Compton 승마 |  | Ⅴ | FACU | 3 | 5 | 2 | 0 | 1 | 0 |
| Ranunculaceae | *Actaea simplex* (DC.) Wormsk. ex Prantl 촛대승마 |  | Ⅰ | FACW | 17 | 1 | 0 | 3 | 0 | 0 |
| Ranunculaceae | *Anemone raddeana* Regel 꿩의바람꽃 |  |  | OBU | 0 | 0 | 0 | 0 | 0 | 1 |
| Ranunculaceae | *Aquilegia buergeriana* Siebold & Zucc. var. *oxysepala* (Trautv. & C. A. Mey.) Kitam. 매발톱 |  | Ⅲ | OBU | 2 | 0 | 0 | 0 | 0 | 0 |
| Ranunculaceae | *Caltha palustris* L. 동의나물 | ○ | Ⅱ | FAC | 34 | 2 | 3 | 0 | 4 | 1 |
| Ranunculaceae | *Clematis apiifolia* DC. 사위질빵 |  |  | OBU | 50 | 28 | 68 | 36 | 48 | 0 |
| Ranunculaceae | *Clematis brachyura* Maxim. 외대으아리 |  | Ⅲ | FACU | 0 | 1 | 0 | 0 | 0 | 0 |
| Ranunculaceae | *Clematis fusca* Turcz. var. *flabellata* (Nakai) J.S.Kim 요강나물 |  | Ⅲ | FAC | 2 | 0 | 0 | 0 | 0 | 0 |
| Ranunculaceae | *Clematis fusca* Turcz. var. *violacea* Maxim. 종덩굴 |  | Ⅲ | FAC | 3 | 0 | 0 | 0 | 0 | 0 |
| Ranunculaceae | *Clematis urticifolia* Nakai ex Kitag. 병조희풀 |  | Ⅲ | OBU | 2 | 0 | 1 | 0 | 0 | 0 |
| Ranunculaceae | *Clematis patens* C. Morren & Decne. 큰꽃으아리 |  | Ⅰ | FACU | 4 | 1 | 0 | 1 | 2 | 0 |
| Ranunculaceae | *Clematis terniflora* DC. 참으아리 |  |  | OBU | 0 | 0 | 0 | 0 | 1 | 0 |
| Ranunculaceae | *Clematis terniflora* DC. var. *mandshurica* (Rupr.) Ohwi 으아리 |  |  | OBU | 11 | 10 | 8 | 4 | 6 | 0 |
| Ranunculaceae | *Clematis trichotoma* Nakai 할미밀망 |  |  | OBU | 12 | 1 | 3 | 5 | 8 | 0 |
| Ranunculaceae | *Hepatica asiatica* Nakai 노루귀 |  | Ⅰ | OBU | 2 | 0 | 0 | 0 | 2 | 0 |
| Ranunculaceae | *Pulsatilla koreana* (Y. Yabe ex Nakai) Nakai ex T. Mori 할미꽃 |  |  | OBU | 1 | 0 | 1 | 0 | 0 | 0 |
| Ranunculaceae | *Ranunculus cantoniensis* DC. 털개구리미나리 |  |  | OBU | 4 | 1 | 8 | 0 | 1 | 0 |
| Ranunculaceae | *Ranunculus chinensis* Bunge 젓가락나물 | ○ |  | OBU | 3 | 0 | 7 | 3 | 7 | 2 |
| Ranunculaceae | *Ranunculus crucilobus* H. Lév. 바위미나리아재비 |  | Ⅳ | OBU | 0 | 0 | 0 | 0 | 0 | 1 |
| Ranunculaceae | *Ranunculus japonicus* Thunb. 미나리아재비 |  |  | FAC | 5 | 0 | 8 | 0 | 5 | 2 |
| Ranunculaceae | *Ranunculus natans* C. A. Mey.북미나리아재비 |  |  | FACW | 0 | 0 | 0 | 0 | 0 | 1 |
| Ranunculaceae | *Ranunculus sceleratus* L. 개구리자리 |  |  | FAC | 4 | 4 | 3 | 0 | 3 | 1 |
| Ranunculaceae | *Ranunculus tachiroei* Franch. & Sav. 개구리미나리 | ○ |  | OBW | 1 | 0 | 11 | 0 | 3 | 0 |
| Ranunculaceae | *Thalictrum actaeifolium* Siebold & Zucc. 은꿩의다리 |  |  | OBU | 0 | 0 | 0 | 1 | 7 | 0 |
| Ranunculaceae | *Thalictrum aquilegiifolium* L. var. *sibiricum* Regel & Tiling 꿩의다리 | ○ |  | OBU | 4 | 1 | 5 | 0 | 2 | 0 |
| Ranunculaceae | *Thalictrum minus* (Pamp.) Pamp. var. *hypoleucum* (Siebold & Zucc.) Miq. 좀꿩의다리 |  |  | OBW | 3 | 1 | 3 | 1 | 0 | 0 |
| Ranunculaceae | *Thalictrum rochebrunnianum* Franch. & Sav. 금꿩의다리 |  | Ⅲ | OBU | 6 | 1 | 0 | 0 | 0 | 0 |
| Ranunculaceae | *Thalictrum tuberiferum* Maxim. 산꿩의다리 |  |  | OBU | 6 | 0 | 2 | 0 | 2 | 0 |
| Berberidaceae | *Berberis amurensis* Rupr. 매발톱나무 | ○ | Ⅱ | OBW | 0 | 1 | 0 | 0 | 0 | 0 |
| Berberidaceae | *Caulophyllum robustum* Maxim. 꿩의다리아재비 | ○ | Ⅱ | OBU | 3 | 0 | 2 | 0 | 0 | 0 |
| Berberidaceae | *Epimedium koreanum* Nakai 삼지구엽초 | ○ | Ⅳ | OBU | 0 | 1 | 0 | 0 | 0 | 0 |
| Lardizabalaceae | *Akebia quinata* (Houtt.) Decne. 으름덩굴 |  |  | OBU | 9 | 7 | 22 | 24 | 32 | 0 |
| Menispermaceae | *Cocculus orbiculatus* (L.) DC. 댕댕이덩굴 |  |  | OBU | 19 | 3 | 40 | 17 | 36 | 0 |
| Menispermaceae | *Menispermum dauricum* DC. 새모래덩굴 | ○ |  | OBU | 24 | 17 | 8 | 7 | 0 | 0 |
| Menispermaceae | *Sinomenium acutum* (Thunb.) Rehder & E. H. Wilson 방기 |  | Ⅲ | FAC | 1 | 0 | 0 | 0 | 0 | 0 |
| Nymphaeaceae | *Nymphaea tetragona* Georgi 수련 | ○ |  | OBU | 0 | 0 | 0 | 0 | 0 | 1 |
| Ceratophyllaceae | *Ceratophyllum demersum* L. 붕어마름 |  |  | OBW | 0 | 0 | 1 | 0 | 0 | 0 |
| Chloranthaceae | *Chloranthus fortunei* (A. Gray) Solms 옥녀꽃대 |  | Ⅰ | OBU | 0 | 0 | 1 | 0 | 2 | 0 |
| Chloranthaceae | *Chloranthus japonicus* Siebold 홀아비꽃대 | ○ | Ⅰ | OBU | 4 | 0 | 2 | 1 | 3 | 0 |
| Menyanthaceae | *Menyanthes trifoliata* L. 조름나물 |  | Ⅴ | OBU | 2 | 0 | 0 | 0 | 0 | 0 |
| Menyanthaceae | *Nymphoides coreana* (H. Lév.) H. Hara 좀어리연꽃 |  |  | OBU | 0 | 0 | 0 | 0 | 0 | 1 |
| Menyanthaceae | *Nymphoides indica* (L.) Kuntze 어리연꽃 |  |  | OBU | 0 | 0 | 0 | 0 | 0 | 5 |
| Actinidiaceae | *Actinidia arguta* (Siebold & Zucc.) Planch. ex Miq. 다래 |  |  | OBU | 62 | 20 | 18 | 16 | 20 | 0 |
| Actinidiaceae | *Actinidia kolomikta* (Maxim. & Rupr.) Maxim. 쥐다래 |  | Ⅲ | OBU | 5 | 0 | 1 | 1 | 0 | 0 |
| Actinidiaceae | *Actinidia polygama* (Siebold & Zucc.) Planch. ex Maxim. 개다래 |  |  | OBU | 14 | 0 | 2 | 4 | 1 | 0 |
| Theaceae | *Camellia japonica* L. 동백나무 |  | Ⅰ | OBW | 0 | 0 | 0 | 0 | 0 | 2 |
| Theaceae | *Camellia sinensis* (L.) Kuntze 차나무 |  |  | OBU | 0 | 0 | 1 | 0 | 0 | 0 |
| Theaceae | *Eurya japonica* Thunb. 사스레피나무 |  | Ⅰ | OBU | 0 | 0 | 1 | 0 | 11 | 1 |
| Theaceae | *Stewartia koreana* Nakai ex Rehder 노각나무 |  | Ⅲ | OBU | 0 | 0 | 1 | 1 | 1 | 0 |
| Clusiaceae | *Hypericum ascyron* L. 물레나물 | ○ |  | FACW | 39 | 10 | 21 | 8 | 7 | 0 |
| Clusiaceae | *Hypericum attenuatum* Fisch. ex Choisy 채고추나물 | ○ | Ⅱ | FAC | 0 | 0 | 0 | 0 | 5 | 0 |
| Clusiaceae | *Hypericum erectum* Thunb. 고추나물 | ○ |  | FAC | 32 | 4 | 21 | 7 | 9 | 1 |
| Clusiaceae | *Hypericum japonicum* Thunb. 애기고추나물 |  |  | OBW | 7 | 1 | 6 | 1 | 7 | 1 |
| Clusiaceae | *Hypericum laxum* (Blume) Koidz. 좀고추나물 |  |  | OBU | 17 | 3 | 12 | 5 | 21 | 4 |
| Clusiaceae | *Triadenum japonicum* (Blume) Makino 물고추나물 | ○ | Ⅲ | OBW | 1 | 0 | 2 | 0 | 0 | 7 |
| Papaveraceae | *Chelidonium majus* L. subsp. *asiaticum* H. Hara 애기똥풀 |  |  | OBW | 19 | 9 | 5 | 6 | 0 | 0 |
| Papaveraceae | *Coreanomecon hylomeconoides* Nakai 매미꽃 |  | Ⅳ | OBU | 0 | 0 | 0 | 0 | 1 | 0 |
| Papaveraceae | *Corydalis ochotensis* Turcz. 눈괴불주머니 | ○ |  | OBW | 0 | 0 | 0 | 4 | 3 | 0 |
| Papaveraceae | *Corydalis pauciovulata* Ohwi 선괴불주머니 |  |  | FACW | 7 | 2 | 0 | 5 | 3 | 0 |
| Papaveraceae | *Corydalis remota* Fisch. ex Maxim. 현호색 |  |  | OBU | 0 | 0 | 0 | 0 | 0 | 1 |
| Papaveraceae | *Corydalis speciosa* Maxim. 산괴불주머니 | ○ |  | OBU | 6 | 3 | 1 | 0 | 1 | 0 |
| Papaveraceae | *Dicentra spectabilis* (L.) Lem. 금낭화 |  | Ⅲ | OBU | 2 | 0 | 0 | 0 | 0 | 0 |
| Papaveraceae | *Hylomecon vernalis* Maxim. 피나물 |  | Ⅱ | FAC | 1 | 1 | 0 | 0 | 1 | 0 |
| Penthoraceae | *Penthorum chinense* Pursh 낙지다리 |  | Ⅱ | OBU | 4 | 0 | 4 | 0 | 0 | 0 |
| Brassicaceae | *Arabis hirsuta* (L.) Scop. 털장대 |  |  | OBU | 0 | 1 | 0 | 0 | 0 | 0 |
| Brassicaceae | *Barbarea orthoceras* Ledeb. 나도냉이 | ○ |  | FACW | 1 | 0 | 0 | 0 | 0 | 0 |
| Brassicaceae | *Barbarea vulgaris* R.Br. 유럽나도냉이 |  |  | OBU | 6 | 0 | 0 | 1 | 0 | 0 |
| Brassicaceae | *Berteroella maximowiczii* (Palib.) O. E. Schulz장대냉이 |  |  | OBU | 0 | 0 | 1 | 0 | 0 | 0 |
| Brassicaceae | *Capsella bursa-pastoris* (L.) Medik. 냉이 |  |  | FACW | 5 | 0 | 0 | 0 | 0 | 0 |
| Brassicaceae | *Cardamine fallax* (O. E. Schulz) Nakai 좁쌀냉이 |  |  | FACW | 2 | 0 | 0 | 0 | 0 | 0 |
| Brassicaceae | *Cardamine flexuosa* With. 황새냉이 |  |  | FACW | 11 | 1 | 1 | 2 | 4 | 0 |
| Brassicaceae | *Cardamine impatiens* L. 싸리냉이 |  |  | FACU | 3 | 0 | 0 | 1 | 0 | 0 |
| Brassicaceae | *Cardamine komarovii* Nakai 는쟁이냉이 |  | Ⅲ | FACW | 6 | 1 | 2 | 0 | 0 | 0 |
| Brassicaceae | *Cardamine leucantha* (Tausch) O. E. Schulz 미나리냉이 |  |  | FAC | 24 | 7 | 2 | 4 | 4 | 0 |
| Brassicaceae | *Cardamine lyrata* Bunge 논냉이 |  |  | FAC | 0 | 0 | 0 | 0 | 2 | 0 |
| Brassicaceae | *Cardamine scutata* Thunb. 큰황새냉이 |  |  | OBU | 3 | 0 | 0 | 0 | 0 | 0 |
| Brassicaceae | *Cardamine yezoensis* Maxim. 왜갓냉이 | ○ | Ⅳ | FAC | 0 | 0 | 2 | 0 | 0 | 0 |
| Brassicaceae | *Catolobus pendulus* (L.) Al-Shehbaz 느러진장대 | ○ | Ⅲ | OBU | 0 | 0 | 0 | 1 | 0 | 0 |
| Brassicaceae | *Lepidium virginicum* L. 콩다닥냉이 |  |  | FAC | 0 | 0 | 1 | 0 | 0 | 0 |
| Brassicaceae | *Rorippa indica* (L.) Hiern 개갓냉이 |  |  | OBU | 1 | 0 | 0 | 0 | 1 | 0 |
| Brassicaceae | *Rorippa palustris* (L.) Besser 속속이풀 |  |  | OBU | 0 | 0 | 3 | 1 | 0 | 0 |
| Brassicaceae | *Sisymbrium luteum* (Maxim.) O. E. Schulz 노란장대 | ○ | Ⅰ | OBU | 2 | 0 | 0 | 0 | 0 | 0 |
| Brassicaceae | *Turritis glabra* L. 장대나물 |  |  | FACU | 3 | 0 | 0 | 1 | 0 | 0 |
| Platanaceae | *Platanus orientalis* L. 버즘나무 |  |  | OBU | 0 | 0 | 0 | 1 | 0 | 0 |
| Crassulaceae | *Hylotelephium erythrostictum* (Miq.) H. Ohba 꿩의비름 |  |  | FAC | 1 | 0 | 0 | 0 | 0 | 0 |
| Crassulaceae | *Phedimus aizoon* (L.) 't Hart 가는기린초 | ○ |  | OBU | 2 | 1 | 1 | 0 | 0 | 0 |
| Crassulaceae | *Phedimus kamtschaticus* (Fisch. & C. A. Mey.) 't Hart 기린초 |  |  | OBW | 6 | 0 | 8 | 2 | 0 | 0 |
| Crassulaceae | *Sedum bulbiferum* Makino 말똥비름 |  |  | FACU | 0 | 0 | 0 | 0 | 2 | 0 |
| Crassulaceae | *Sedum oryzifolium* Makino 땅채송화 |  |  | OBU | 0 | 0 | 0 | 0 | 1 | 0 |
| Crassulaceae | *Sedum polytrichoides* Hemsl. 바위채송화 |  |  | OBU | 0 | 0 | 0 | 0 | 1 | 0 |
| Crassulaceae | *Sedum sarmentosum* Bunge 돌나물 |  |  | FAC | 6 | 1 | 10 | 2 | 1 | 0 |
| Saxifragaceae | *Astilbe chinensis* (Maxim.) Franch. & Sav. 노루오줌 |  |  | FACU | 71 | 13 | 24 | 15 | 24 | 0 |
| Saxifragaceae | *Astilbe koreana* (Kom.) Nakai 숙은노루오줌 |  |  | OBW | 11 | 6 | 3 | 0 | 1 | 0 |
| Saxifragaceae | *Chrysosplenium barbatum* Nakai 흰털괭이눈 |  |  | OBU | 9 | 1 | 0 | 3 | 2 | 0 |
| Saxifragaceae | *Chrysosplenium flagelliferum* F. Schmidt 애기괭이눈 | ○ |  | OBU | 9 | 1 | 0 | 2 | 0 | 0 |
| Saxifragaceae | *Chrysosplenium grayanum* Maxim. 괭이눈 |  |  | FACW | 1 | 0 | 0 | 0 | 1 | 0 |
| Saxifragaceae | *Chrysosplenium pilosum* Maxim. 털괭이눈 | ○ | Ⅱ | OBU | 1 | 0 | 0 | 0 | 0 | 0 |
| Saxifragaceae | *Chrysosplenium ramosum* Maxim. 가지괭이눈 | ○ | Ⅳ | OBU | 1 | 0 | 0 | 0 | 0 | 0 |
| Saxifragaceae | *Chrysosplenium sinicum* Maxim. 선괭이눈 |  | Ⅰ | FAC | 2 | 0 | 0 | 0 | 0 | 0 |
| Saxifragaceae | *Micranthes octopetala* (Nakai) Y. I. Kim & Y. D. Kim 구실바위취 |  | Ⅳ | FACW | 5 | 0 | 0 | 0 | 0 | 0 |
| Saxifragaceae | *Mukdenia rossii* (Oliv.) Koidz. 돌단풍 |  | Ⅱ | FACW | 0 | 1 | 0 | 0 | 0 | 0 |
| Saxifragaceae | *Rodgersia podophylla* A. Gray 도깨비부채 |  | Ⅳ | OBW | 0 | 0 | 0 | 1 | 0 | 0 |
| Saxifragaceae | *Saxifraga fortunei* Hook. 바위떡풀 |  |  | OBU | 4 | 0 | 0 | 0 | 0 | 0 |
| Saxifragaceae | *Saxifraga stolonifera* Curtis 바위취 |  |  | OBU | 3 | 0 | 0 | 0 | 0 | 0 |
| Hydrangeaceae | *Deutzia glabrata* Kom. 물참대 | ○ | Ⅰ | FACW | 4 | 0 | 0 | 1 | 0 | 0 |
| Hydrangeaceae | *Deutzia parviflora* Bunge 말발도리 | ○ | Ⅰ | OBU | 2 | 0 | 1 | 1 | 0 | 0 |
| Hydrangeaceae | *Deutzia uniflora* Shirai 매화말발도리 |  | Ⅰ | OBU | 1 | 0 | 1 | 1 | 0 | 0 |
| Hydrangeaceae | *Hydrangea macrophylla* (Thunb.) Ser. 수국 |  |  | FACU | 0 | 0 | 0 | 1 | 1 | 0 |
| Hydrangeaceae | *Hydrangea macrophylla* (Thunb.) Ser. subsp. *serrata* (Thunb.) Makino 산수국 |  |  | OBU | 2 | 2 | 8 | 8 | 3 | 1 |
| Hydrangeaceae | *Hydrangea petiolaris* Siebold & Zucc. 등수국 |  | Ⅲ | FACU | 0 | 0 | 0 | 0 | 0 | 1 |
| Hydrangeaceae | *Philadelphus schrenkii* Rupr. 고광나무 |  | Ⅲ | OBU | 9 | 0 | 3 | 0 | 3 | 0 |
| Hydrangeaceae | *Philadelphus tenuifolius* Rupr. & Maxim. 얇은잎고광나무 | ○ |  | FACW | 14 | 2 | 3 | 5 | 3 | 0 |
| Hydrangeaceae | *Schizophragma hydrangeoides* Siebold & Zucc. 바위수국 |  | Ⅳ | OBU | 0 | 0 | 0 | 0 | 0 | 1 |
| Parnassiaceae | *Parnassia palustris* L. 물매화 | ○ |  | OBU | 3 | 0 | 1 | 0 | 6 | 0 |
| Grossulariaceae | *Ribes fasciculatum* Siebold & Zucc. var. *chinense* Maxim. 까마귀밥나무 |  |  | OBU | 1 | 1 | 4 | 2 | 1 | 0 |
| Grossulariaceae | *Ribes mandshuricum* (Maxim.) Kom. 까치밥나무 | ○ | Ⅲ | OBU | 0 | 2 | 0 | 1 | 0 | 0 |
| Rosaceae | *Agrimonia coreana* Nakai 산짚신나물 | ○ |  | OBU | 18 | 5 | 12 | 5 | 5 | 0 |
| Rosaceae | *Agrimonia pilosa* Ledeb. 짚신나물 |  |  | OBU | 44 | 19 | 37 | 21 | 24 | 1 |
| Rosaceae | *Aruncus dioicus* (Walter) Fernald 눈개승마 |  | Ⅲ | FAC | 4 | 0 | 0 | 0 | 0 | 0 |
| Rosaceae | *Crataegus pinnatifida* Bunge 산사나무 |  |  | OBW | 3 | 2 | 1 | 1 | 1 | 0 |
| Rosaceae | *Duchesnea chrysantha* (Zoll. & Moritzi) Miq. 산뱀딸기 |  |  | OBU | 2 | 2 | 1 | 0 | 3 | 0 |
| Rosaceae | *Duchesnea indica* (Andrews) Teschem. 뱀딸기 |  |  | FAC | 30 | 10 | 28 | 20 | 9 | 0 |
| Rosaceae | *Filipendula formosa* Nakai 지리터리풀 |  | Ⅳ | OBU | 0 | 0 | 1 | 0 | 0 | 0 |
| Rosaceae | *Filipendula glaberrima* Nakai 터리풀 | ○ | Ⅰ | OBU | 20 | 0 | 0 | 1 | 1 | 0 |
| Rosaceae | *Geum aleppicum* Jacq. 큰뱀무 |  |  | OBU | 9 | 1 | 4 | 1 | 0 | 0 |
| Rosaceae | *Geum japonicum* Thunb. 뱀무 |  |  | FACW | 3 | 0 | 2 | 2 | 0 | 0 |
| Rosaceae | *Malus baccata* (L.) Borkh. 야광나무 | ○ | Ⅰ | OBU | 9 | 1 | 1 | 1 | 1 | 0 |
| Rosaceae | *Malus mandshurica* (Maxim.) Kom. ex Skvortsov 털야광나무 |  | Ⅰ | OBU | 2 | 0 | 0 | 0 | 0 | 0 |
| Rosaceae | *Malus toringo* (Siebold) de Vriese 아그배나무 |  |  | OBU | 0 | 0 | 0 | 0 | 1 | 5 |
| Rosaceae | *Potentilla anemonifolia* Lehm. 가락지나물 |  |  | FAC | 2 | 3 | 3 | 0 | 0 | 7 |
| Rosaceae | *Potentilla centigrana* Maxim. 좀딸기 | ○ | Ⅲ | OBU | 3 | 1 | 0 | 0 | 0 | 0 |
| Rosaceae | *Potentilla cryptotaeniae* Maxim. 물양지꽃 | ○ | Ⅲ | FACW | 10 | 0 | 0 | 5 | 0 | 0 |
| Rosaceae | *Potentilla fragarioides* L. 양지꽃 | ○ |  | FAC | 10 | 2 | 12 | 10 | 5 | 2 |
| Rosaceae | *Potentilla freyniana* Bornm. 세잎양지꽃 |  |  | OBU | 22 | 9 | 14 | 8 | 21 | 0 |
| Rosaceae | *Potentilla rosulifera* H. Lév. 민눈양지꽃 |  | Ⅱ | OBU | 1 | 0 | 0 | 0 | 0 | 2 |
| Rosaceae | *Pourthiaea villosa* (Thunb.) Decne. 윤노리나무 |  | Ⅰ | OBU | 0 | 0 | 2 | 0 | 4 | 3 |
| Rosaceae | *Prunus armeniaca* L. 살구나무 |  |  | FACU | 1 | 0 | 0 | 0 | 0 | 0 |
| Rosaceae | *Prunus davidiana* (Carrière) Franch. 산복사나무 |  |  | OBU | 1 | 0 | 0 | 0 | 0 | 0 |
| Rosaceae | *Prunus japonica* Thunb. 산이스라지 |  |  | OBU | 0 | 2 | 0 | 0 | 0 | 0 |
| Rosaceae | *Prunus japonica* Thunb. var. *nakaii* (H. Lév.) Rehder 이스라지 |  |  | FACW | 1 | 0 | 3 | 0 | 1 | 0 |
| Rosaceae | *Prunus maackii* Rupr. 개벚지나무 | ○ | Ⅲ | OBU | 0 | 0 | 0 | 0 | 0 | 1 |
| Rosaceae | *Prunus mandshurica* (Maxim.) Koehne 개살구나무 | ○ | Ⅲ | OBU | 0 | 0 | 1 | 0 | 0 | 0 |
| Rosaceae | *Prunus maximowiczii* Rupr. 산개벚지나무 | ○ | Ⅱ | OBU | 1 | 0 | 0 | 0 | 0 | 1 |
| Rosaceae | *Prunus padus* L. 귀룽나무 |  |  | OBW | 24 | 2 | 2 | 0 | 2 | 0 |
| Rosaceae | *Prunus persica* (L.) Batsch. 복사나무 |  |  | OBU | 8 | 4 | 9 | 3 | 0 | 0 |
| Rosaceae | *Prunus sargentii* Rehder 산벚나무 | ○ | Ⅲ | OBU | 9 | 3 | 12 | 3 | 24 | 0 |
| Rosaceae | *Prunus sargentii* Rehder var. *verecunda* (Koidz.) Chin S. Chang 분홍벚나무 |  |  | OBU | 8 | 3 | 2 | 7 | 0 | 0 |
| Rosaceae | *Prunus serrulata* Lindl. var. *pubescens* (Makino) Nakai 잔털벚나무 |  |  | FACW | 17 | 4 | 5 | 2 | 2 | 0 |
| Rosaceae | *Prunus serrulata* Lindl. f. *spontanea* (E. H. Wilson) Chin S. Chang 벚나무 |  |  | OBU | 0 | 3 | 8 | 7 | 9 | 0 |
| Rosaceae | *Prunus tomentosa* Thunb. 앵도나무 |  |  | FACU | 0 | 0 | 1 | 0 | 0 | 0 |
| Rosaceae | *Prunus* ⨉ *yedoensis* Matsum. 왕벚나무 |  | Ⅳ | OBW | 0 | 0 | 0 | 1 | 1 | 0 |
| Rosaceae | *Pyrus calleryana* Decne. var. *fauriei* (C. K. Schneid.) Rehder 콩배나무 |  | Ⅰ | OBU | 2 | 2 | 1 | 0 | 8 | 0 |
| Rosaceae | *Pyrus pyrifolia* (Burm.f.) Nakai 돌배나무 |  |  | OBU | 9 | 0 | 2 | 2 | 0 | 0 |
| Rosaceae | *Pyrus ussuriensis* Maxim. ex Rupr. 산돌배 | ○ |  | OBU | 8 | 0 | 1 | 0 | 0 | 0 |
| Rosaceae | *Rosa davurica* Pall. 생열귀나무 | ○ | Ⅳ | OBU | 1 | 0 | 0 | 0 | 0 | 0 |
| Rosaceae | *Rosa lucieae* Franch. & Rochebr. ex Crép. 돌가시나무 |  | Ⅰ | FACU | 0 | 0 | 7 | 0 | 13 | 3 |
| Rosaceae | *Rosa maximowicziana* Regel 용가시나무 | ○ |  | OBU | 0 | 0 | 1 | 0 | 1 | 0 |
| Rosaceae | *Rosa multiflora* Thunb. 찔레꽃 |  |  | FAC | 53 | 22 | 64 | 29 | 57 | 14 |
| Rosaceae | *Rubus buergeri* Miq. 겨울딸기 |  | Ⅲ | OBU | 0 | 0 | 0 | 0 | 0 | 1 |
| Rosaceae | *Rubus corchorifolius* L.f. 수리딸기 |  | Ⅰ | FAC | 0 | 0 | 0 | 1 | 31 | 0 |
| Rosaceae | *Rubus coreanus* Miq. 복분자딸기 |  |  | FACU | 10 | 2 | 1 | 5 | 4 | 0 |
| Rosaceae | *Rubus crataegifolius* Bunge 산딸기 | ○ |  | OBU | 58 | 11 | 40 | 21 | 26 | 0 |
| Rosaceae | *Rubus parvifolius* L. 멍석딸기 |  |  | FACU | 17 | 10 | 29 | 12 | 15 | 0 |
| Rosaceae | *Rubus phoenicolasius* Maxim. 곰딸기 |  |  | OBU | 23 | 10 | 15 | 6 | 2 | 0 |
| Rosaceae | *Rubus pungens* Cambess. 줄딸기 |  |  | OBU | 65 | 3 | 42 | 10 | 3 | 1 |
| Rosaceae | *Sanguisorba longifolia* Bertol. 긴오이풀 |  | Ⅳ | OBU | 0 | 0 | 1 | 0 | 0 | 0 |
| Rosaceae | *Sanguisorba officinalis* L. 오이풀 |  |  | OBU | 5 | 3 | 14 | 2 | 16 | 3 |
| Rosaceae | *Sanguisorba* ⨉ *tenuifolia* Fisch. ex Link가는오이풀 | ○ | Ⅰ | FACW | 0 | 1 | 9 | 1 | 14 | 1 |
| Rosaceae | *Sorbaria sorbifolia* (L.) A. Braun var. *stellipila* Maxim. 쉬땅나무 | ○ | Ⅲ | OBU | 6 | 0 | 0 | 0 | 0 | 0 |
| Rosaceae | *Aria alnifolia* (Siebold & Zucc.) Decne. 팥배나무 |  |  | OBU | 7 | 3 | 1 | 2 | 5 | 1 |
| Rosaceae | *Sorbus commixta* Hedl. 마가목 |  | Ⅱ | FACU | 1 | 0 | 0 | 0 | 0 | 0 |
| Rosaceae | *Spiraea blumei* G. Don 산조팝나무 |  | Ⅰ | OBU | 3 | 0 | 1 | 0 | 1 | 0 |
| Rosaceae | *Spiraea fritschiana* C. K. Schneid. 참조팝나무 |  | Ⅲ | OBU | 21 | 0 | 1 | 0 | 0 | 0 |
| Rosaceae | *Spiraea microgyna* Nakai 좀조팝나무 |  | Ⅲ | FACU | 0 | 0 | 0 | 1 | 0 | 0 |
| Rosaceae | *Spiraea prunifolia* Siebold & Zucc. f. *simpliciflora* Nakai 조팝나무 |  |  | OBU | 37 | 20 | 40 | 30 | 20 | 0 |
| Rosaceae | *Spiraea salicifolia* L. 꼬리조팝나무 | ○ | Ⅱ | OBW | 23 | 10 | 7 | 5 | 2 | 0 |
| Rosaceae | *Spiraea trichocarpa* Nakai 갈기조팝나무 |  | Ⅳ | OBW | 1 | 0 | 0 | 0 | 0 | 0 |
| Rosaceae | *Stephanandra incisa* (Thunb.) Zabel 국수나무 |  |  | OBU | 61 | 16 | 25 | 18 | 30 | 0 |
| Rosaceae | *Waldsteinia ternata* (Stephan) Fritsch 나도양지꽃 | ○ | Ⅳ | FAC | 1 | 0 | 0 | 0 | 0 | 0 |
| Fabaceae | *Aeschynomene indica* L. 자귀풀 |  |  | OBU | 0 | 0 | 1 | 1 | 0 | 0 |
| Fabaceae | *Albizia julibrissin* Durazz. 자귀나무 |  |  | OBU | 0 | 1 | 14 | 6 | 23 | 0 |
| Fabaceae | *Amorpha fruticosa* L. 족제비싸리 |  |  | OBU | 18 | 2 | 14 | 6 | 0 | 0 |
| Fabaceae | *Amphicarpaea bracteata* (L.) Fernald subsp. *edgeworthii* (Benth.) H. Ohashi 새콩 |  |  | FAC | 60 | 28 | 58 | 23 | 33 | 0 |
| Fabaceae | *Astragalus penduliflorus* Lam. var. *dahuricus* (DC.) X. Y. Zhu 황기 |  |  | OBU | 0 | 0 | 1 | 2 | 0 | 0 |
| Fabaceae | *Chamaecrista nomame* (Makino) H. Ohashi 차풀 |  |  | OBU | 2 | 2 | 0 | 1 | 1 | 0 |
| Fabaceae | *Dunbaria villosa* (Thunb.) Makino 여우팥 |  |  | OBW | 4 | 1 | 0 | 4 | 3 | 0 |
| Fabaceae | *Glycine max* (L.) Merr. subsp. *soja* (Siebold & Zucc.) H. Ohashi 돌콩 |  |  | FACW | 19 | 4 | 24 | 17 | 6 | 0 |
| Fabaceae | *Hylodesmum oldhamii* (Oliv.) H. Ohashi & R. R. Mill  큰도둑놈의갈고리 |  |  | OBU | 0 | 0 | 2 | 0 | 3 | 0 |
| Fabaceae | *Hylodesmum podocarpum* (DC.) H. Ohashi & R. R. Mill 개도둑놈의갈고리 |  |  | OBU | 1 | 0 | 4 | 0 | 1 | 0 |
| Fabaceae | *Hylodesmum podocarpum* (DC.) H. Ohashi & R. R. Mill subsp. *oxyphyllum* (DC.) H. Ohashi & R. R. Mill 도둑놈의갈고리 |  |  | OBU | 29 | 11 | 20 | 1 | 15 | 0 |
| Fabaceae | *Hylodesmum podocarpum* (DC.) H. Ohashi & R. R. Mill var. *mandshuricum* (Maxim.) H. Ohashi & R. R. Mill 애기도둑놈의갈고리 |  |  | OBU | 0 | 0 | 0 | 1 | 5 | 0 |
| Fabaceae | *Indigofera kirilowii* Maxim. ex Palib. 땅비싸리 |  |  | FACU | 2 | 1 | 3 | 4 | 22 | 0 |
| Fabaceae | *Indigofera koreana* Ohwi 좀땅비싸리 |  |  | FACW | 0 | 0 | 0 | 0 | 1 | 0 |
| Fabaceae | *Indigofera pseudotinctoria* Matsum. 낭아초 |  | Ⅲ | FAC | 0 | 0 | 0 | 2 | 0 | 0 |
| Fabaceae | *Kummerowia stipulacea* (Maxim.) Makino 둥근매듭풀 |  |  | OBU | 0 | 0 | 0 | 2 | 1 | 0 |
| Fabaceae | *Kummerowia striata* (Thunb.) Schindl. 매듭풀 |  |  | OBU | 12 | 6 | 15 | 4 | 4 | 0 |
| Fabaceae | *Lathyrus davidii* Hance 활량나물 |  |  | FAC | 4 | 0 | 1 | 1 | 0 | 0 |
| Fabaceae | *Lespedeza bicolor* Turcz. 싸리 |  |  | FACU | 31 | 9 | 21 | 8 | 26 | 0 |
| Fabaceae | *Lespedeza cuneata* (Dum. Cours.) G. Don 비수리 |  |  | OBU | 12 | 1 | 20 | 6 | 7 | 3 |
| Fabaceae | *Lespedeza cyrtobotrya* Miq. 참싸리 |  |  | OBU | 16 | 2 | 11 | 7 | 6 | 0 |
| Fabaceae | *Lespedeza davurica* (Laxm.) Schindl. 호비수리 | ○ | Ⅲ | OBU | 0 | 0 | 1 | 0 | 0 | 0 |
| Fabaceae | *Lespedeza maritima* Nakai 해변싸리 |  | Ⅰ | FACU | 0 | 0 | 3 | 0 | 0 | 0 |
| Fabaceae | *Lespedeza maximowiczii* C. K. Schneid. 조록싸리 |  |  | OBU | 49 | 2 | 28 | 15 | 23 | 0 |
| Fabaceae | *Lespedeza maximowiczii* C. K. Schneid. var. *tomentella* (Nakai) Nakai 털조록싸리 |  |  | OBU | 15 | 0 | 2 | 2 | 6 | 0 |
| Fabaceae | *Lespedeza pilosa* (Thunb.) Siebold & Zucc 괭이싸리 |  |  | OBU | 0 | 0 | 0 | 0 | 2 | 0 |
| Fabaceae | *Lespedeza thunbergii* (DC.) Nakai 풀싸리 |  |  | FACU | 0 | 0 | 0 | 0 | 1 | 0 |
| Fabaceae | *Lespedeza tomentosa* (Thunb.) Siebold ex Maxim. 개싸리 |  |  | OBU | 0 | 0 | 2 | 1 | 0 | 0 |
| Fabaceae | *Lespedeza virgata* (Thunb.) DC. 좀싸리 |  |  | OBU | 0 | 0 | 2 | 0 | 0 | 0 |
| Fabaceae | *Lotus corniculatus* L. var. *japonica* Regel 벌노랑이 |  |  | FACU | 1 | 0 | 0 | 0 | 0 | 0 |
| Fabaceae | *Maackia amurensis* Rupr. 다릅나무 | ○ |  | FACW | 30 | 3 | 9 | 3 | 6 | 0 |
| Fabaceae | *Maackia fauriei* (H. Lév.) Takeda 솔비나무 |  | Ⅳ | OBW | 0 | 0 | 0 | 0 | 0 | 5 |
| Fabaceae | *Medicago polymorpha* L. 개자리 |  |  | FAC | 0 | 0 | 1 | 0 | 0 | 0 |
| Fabaceae | *Pueraria lobata* (Willd.) Ohwi 칡 |  |  | OBU | 29 | 17 | 35 | 24 | 27 | 0 |
| Fabaceae | *Robinia pseudoacacia* L. 아까시나무 |  |  | OBU | 41 | 10 | 29 | 19 | 12 | 0 |
| Fabaceae | *Sophora flavescens* Aiton 고삼 |  |  | OBU | 15 | 3 | 8 | 2 | 5 | 0 |
| Fabaceae | *Trifolium pratense* L. 붉은토끼풀 |  |  | FAC | 1 | 0 | 0 | 1 | 1 | 0 |
| Fabaceae | *Trifolium repens* L. 토끼풀 |  |  | FACW | 13 | 1 | 7 | 4 | 1 | 2 |
| Fabaceae | *Vicia amoena* Fisch. ex Ser. 갈퀴나물 | ○ |  | OBU | 8 | 0 | 9 | 4 | 3 | 0 |
| Fabaceae | *Vicia amurensis* Oett. 벌완두 | ○ |  | OBU | 2 | 2 | 0 | 0 | 0 | 0 |
| Fabaceae | *Vicia angustifolia* L. var. *minor* (Bertol.) Ohwi 가는갈퀴 |  |  | FACU | 1 | 0 | 0 | 0 | 0 | 0 |
| Fabaceae | *Vicia chosenensis* Ohwi 노랑갈퀴 |  | Ⅲ | OBW | 1 | 0 | 1 | 0 | 0 | 0 |
| Fabaceae | *Vicia cracca* L. 등갈퀴나물 |  |  | FAC | 2 | 0 | 0 | 0 | 0 | 0 |
| Fabaceae | *Vicia nipponica* Matsum. 네잎갈퀴나물 |  |  | OBU | 2 | 1 | 0 | 0 | 2 | 0 |
| Fabaceae | *Vicia pseudoorobus* Fisch. & C. A. Mey.큰등갈퀴 | ○ | Ⅰ | OBW | 0 | 0 | 1 | 0 | 0 | 0 |
| Fabaceae | *Vicia unijuga* A. Braun 나비나물 | ○ |  | OBW | 10 | 3 | 5 | 0 | 3 | 0 |
| Fabaceae | *Vicia unijuga* A. Braun var. *ouensanensis* H. Lév. 큰나비나물 |  |  | OBU | 0 | 0 | 0 | 1 | 0 | 0 |
| Fabaceae | *Vicia venosa* (Link) Maxim. 연리갈퀴 | ○ |  | OBU | 1 | 0 | 1 | 0 | 0 | 0 |
| Fabaceae | *Vicia venosa* (Link) Maxim. var. *cuspidata* Maxim 광릉갈퀴 |  |  | OBU | 19 | 1 | 1 | 3 | 0 | 0 |
| Fabaceae | *Vicia villosa* Roth 벳지 |  |  | FAC | 0 | 0 | 1 | 0 | 1 | 0 |
| Fabaceae | *Vigna angularis* (Willd.) Ohwi & H. Ohashi var. *nipponensis* (Ohwi) Ohwi & H. Ohashi 새팥 |  |  | OBU | 5 | 0 | 11 | 8 | 5 | 0 |
| Fabaceae | *Wisteria floribunda* (Willd.) DC. 등 |  | Ⅳ | FACU | 2 | 0 | 4 | 1 | 0 | 0 |
| Oxalidaceae | *Oxalis acetosella* L. 애기괭이밥 |  | Ⅲ | OBU | 0 | 0 | 0 | 0 | 0 | 1 |
| Oxalidaceae | *Oxalis corniculata* L. 괭이밥 |  |  | FACU | 26 | 10 | 19 | 11 | 6 | 0 |
| Oxalidaceae | *Oxalis obtriangulata* Maxim. 큰괭이밥 | ○ |  | OBU | 3 | 0 | 0 | 0 | 0 | 0 |
| Oxalidaceae | *Oxalis stricta* L. 선괭이밥 |  |  | OBU | 6 | 1 | 2 | 3 | 0 | 1 |
| Geraniaceae | *Geranium krameri* Franch. & Sav. 선이질풀 | ○ |  | FAC | 0 | 0 | 3 | 0 | 0 | 0 |
| Geraniaceae | *Geranium sibiricum* L. 쥐손이풀 |  |  | FACU | 3 | 3 | 0 | 5 | 0 | 0 |
| Geraniaceae | *Geranium thunbergii* Siebold ex Lindl. & Paxton 이질풀 |  |  | OBU | 11 | 2 | 20 | 8 | 2 | 0 |
| Geraniaceae | *Geranium thunbergii* Siebold ex Lindl. & Paxton f. *pallidum* (Nakai ex H. Hara) Murata 흰이질풀 |  |  | FAC | 2 | 0 | 0 | 0 | 1 | 0 |
| Geraniaceae | *Geranium wilfordii* Maxim. 세잎쥐손이 |  |  | FACU | 2 | 0 | 0 | 2 | 1 | 0 |
| Euphorbiaceae | *Acalypha australis* L. 깨풀 |  |  | OBU | 3 | 0 | 6 | 4 | 3 | 1 |
| Euphorbiaceae | *Euphorbia humifusa* Willd. ex Schltdl. 땅빈대 |  |  | OBU | 0 | 0 | 2 | 0 | 0 | 0 |
| Euphorbiaceae | *Euphorbia pekinensis* Rupr. 대극 |  | Ⅰ | FACW | 0 | 0 | 1 | 0 | 3 | 0 |
| Euphorbiaceae | *Euphorbia sieboldiana* Morren & Decne. 개감수 |  |  | FAC | 0 | 1 | 0 | 0 | 1 | 0 |
| Euphorbiaceae | *Mallotus japonicus* (L.f.) Müll. Arg. 예덕나무 |  | Ⅰ | OBU | 0 | 0 | 0 | 0 | 5 | 1 |
| Euphorbiaceae | *Neoshirakia japonica* (Siebold & Zucc.) Esser 사람주나무 |  | Ⅰ | OBW | 0 | 0 | 0 | 0 | 2 | 0 |
| Euphorbiaceae | *Phyllanthus urinaria* L. 여우구슬 |  |  | FACU | 0 | 1 | 3 | 0 | 0 | 1 |
| Euphorbiaceae | *Phyllanthus ussuriensis* Rupr. & Maxim. 여우주머니 |  |  | OBU | 0 | 0 | 3 | 0 | 1 | 0 |
| Euphorbiaceae | *Securinega suffruticosa* (Pall.) Rehder 광대싸리 |  |  | FACU | 21 | 16 | 17 | 14 | 3 | 0 |
| Daphniphyllaceae | *Daphniphyllum macropodum* Miq. 굴거리나무 |  | Ⅲ | OBU | 0 | 0 | 0 | 0 | 0 | 2 |
| Rutaceae | *Dictamnus dasycarpus* Turcz. 백선 | ○ | Ⅰ | OBU | 1 | 2 | 2 | 2 | 0 | 0 |
| Rutaceae | *Orixa japonica* Thunb. 상산 |  | Ⅰ | OBU | 0 | 0 | 0 | 0 | 1 | 0 |
| Rutaceae | *Phellodendron amurense* Rupr. 황벽나무 | ○ | Ⅱ | FAC | 1 | 0 | 0 | 0 | 0 | 0 |
| Rutaceae | *Tetradium daniellii* (Benn.) T. G. Hartley 쉬나무 |  |  | OBW | 0 | 0 | 0 | 1 | 0 | 0 |
| Rutaceae | *Zanthoxylum armatum* DC. 개산초 |  | Ⅰ | OBU | 0 | 0 | 0 | 0 | 1 | 0 |
| Rutaceae | *Zanthoxylum piperitum* (L.) DC. 초피나무 |  |  | OBU | 9 | 1 | 20 | 4 | 8 | 0 |
| Rutaceae | *Zanthoxylum schinifolium* Siebold & Zucc. 산초나무 |  |  | FACU | 62 | 24 | 61 | 34 | 43 | 0 |
| Simaroubaceae | *Ailanthus altissima* (Mill.) Swingle 가죽나무 |  |  | FAC | 10 | 2 | 3 | 7 | 0 | 0 |
| Simaroubaceae | *Picrasma quassioides* (D. Don) Benn. 소태나무 |  |  | FACW | 0 | 0 | 2 | 3 | 3 | 0 |
| Polygalaceae | *Polygala japonica* Houtt. 애기풀 |  |  | OBU | 0 | 1 | 1 | 0 | 0 | 0 |
| Anacardiaceae | *Rhus chinensis* Mill. 붉나무 |  |  | OBU | 18 | 8 | 12 | 15 | 16 | 0 |
| Anacardiaceae | *Toxicodendron succedaneum* (L.) Kuntze 검양옻나무 |  | Ⅳ | OBW | 2 | 0 | 2 | 1 | 17 | 0 |
| Anacardiaceae | *Toxicodendron sylvestre* (Siebold & Zucc.) Kuntze  산검양옻나무 |  | Ⅰ | OBU | 0 | 0 | 1 | 0 | 7 | 0 |
| Anacardiaceae | *Toxicodendron trichocarpum* (Miq.) Kuntze 개옻나무 |  |  | FACU | 27 | 7 | 22 | 10 | 15 | 0 |
| Anacardiaceae | *Toxicodendron vernicifluum* (Stokes) F. A. Barkley 옻나무 |  |  | OBU | 1 | 2 | 3 | 1 | 1 | 0 |
| Aceraceae | *Acer barbinerve* Maxim. 청시닥나무 | ○ | Ⅲ | OBU | 10 | 0 | 0 | 0 | 0 | 0 |
| Aceraceae | *Acer komarovii* Pojark. 시닥나무 | ○ | Ⅲ | FAC | 7 | 2 | 1 | 0 | 0 | 0 |
| Aceraceae | *Acer mandshuricum* Maxim. 복장나무 | ○ | Ⅲ | OBU | 7 | 0 | 0 | 0 | 0 | 0 |
| Aceraceae | *Acer palmatum* Thunb. 단풍나무 |  | Ⅲ | OBU | 3 | 2 | 3 | 0 | 2 | 0 |
| Aceraceae | *Acer pictum* Thunb. var. *mono* (Maxim.) Maxim. ex Franch. 고로쇠나무 |  |  | OBU | 29 | 1 | 3 | 7 | 3 | 0 |
| Aceraceae | *Acer pseudosieboldianum* (Pax) Kom. 당단풍나무 |  |  | OBU | 47 | 2 | 8 | 6 | 10 | 1 |
| Aceraceae | *Acer saccharinum* L. 은단풍 |  |  | OBU | 0 | 0 | 0 | 2 | 0 | 0 |
| Aceraceae | *Acer tataricum* L. subsp. *ginnala* (Maxim.) Wesm. 신나무 |  |  | OBW | 77 | 30 | 39 | 26 | 20 | 0 |
| Aceraceae | *Acer tegmentosum* Maxim. 산겨릅나무 | ○ | Ⅳ | OBU | 3 | 0 | 0 | 0 | 0 | 0 |
| Aceraceae | *Acer triflorum* Kom. 복자기 |  | Ⅲ | OBU | 8 | 0 | 0 | 1 | 0 | 0 |
| Aceraceae | *Acer truncatum* Bunge 만주고로쇠 |  |  | OBU | 5 | 1 | 0 | 0 | 0 | 0 |
| Aceraceae | *Acer ukurunduense* Trautv. & C. A. Mey.부게꽃나무 |  | Ⅲ | OBU | 1 | 0 | 0 | 0 | 0 | 0 |
| Sabiaceae | *Meliosma myriantha* Siebold & Zucc. 나도밤나무 |  | Ⅰ | FACU | 0 | 0 | 1 | 0 | 0 | 0 |
| Sabiaceae | *Meliosma pinnata* (Roxb.) Maxim. var. *oldhamii* (Miq. ex Maxim.) Beusekom 합다리나무 |  | Ⅰ | FAC | 0 | 0 | 0 | 0 | 2 | 0 |
| Balsaminaceae | *Impatiens balsamina* L. 봉선화 |  |  | FACU | 0 | 0 | 2 | 0 | 0 | 0 |
| Balsaminaceae | *Impatiens noli-tangere* L. 노랑물봉선 |  | Ⅰ | OBU | 20 | 6 | 2 | 2 | 1 | 0 |
| Balsaminaceae | *Impatiens textorii* Miq. 물봉선 |  |  | FACW | 85 | 22 | 52 | 31 | 45 | 0 |
| Balsaminaceae | *Impatiens textori* Miq. var. *koreana* (Nakai) Naka 흰물봉선 |  |  | OBU | 9 | 0 | 0 | 0 | 0 | 0 |
| Aquifoliaceae | *Ilex crenata* Thunb. 꽝꽝나무 |  | Ⅲ | OBU | 0 | 0 | 0 | 0 | 2 | 6 |
| Aquifoliaceae | *Ilex macropoda* Miq. 대팻집나무 |  | Ⅰ | FAC | 0 | 0 | 1 | 0 | 5 | 0 |
| Celastraceae | *Celastrus flagellaris* Rupr. 푼지나무 |  |  | FAC | 10 | 1 | 7 | 3 | 2 | 0 |
| Celastraceae | *Celastrus orbiculatus* Thunb. 노박덩굴 |  |  | OBU | 36 | 20 | 28 | 20 | 22 | 0 |
| Celastraceae | *Euonymus alatus* (Thunb.) Siebold 화살나무 |  |  | FACW | 4 | 0 | 9 | 0 | 3 | 0 |
| Celastraceae | *Euonymus alatus* (Thunb.) Siebold f. *ciliato-dentatus* (Franch. & Sav.) Hiyama 회잎나무 |  |  | OBU | 9 | 3 | 6 | 3 | 5 | 0 |
| Celastraceae | *Euonymus fortunei* (Turcz.) Hand.-Mazz. var. *radicans* (Siebold ex Miq.) Rehder 줄사철나무 |  |  | OBU | 0 | 0 | 2 | 0 | 1 | 1 |
| Celastraceae | *Euonymus hamiltonianus* Wall. 참빗살나무 |  |  | FACU | 1 | 0 | 1 | 0 | 0 | 1 |
| Celastraceae | *Euonymus hamiltonianus* Wall. var. *maackii* (Rupr.) Kom. 좀참빗살나무 |  |  | FAC | 0 | 0 | 3 | 0 | 0 | 0 |
| Celastraceae | *Euonymus macropterus* Rupr. 나래회나무 | ○ | Ⅱ | OBU | 3 | 0 | 0 | 0 | 0 | 0 |
| Celastraceae | *Euonymus oxyphyllus* Miq. 참회나무 |  |  | OBW | 5 | 0 | 1 | 2 | 2 | 0 |
| Celastraceae | *Euonymus pauciflorus* Maxim. 회목나무 |  | Ⅱ | OBW | 1 | 0 | 0 | 0 | 0 | 0 |
| Celastraceae | *Euonymus sachalinensis* (F. Schmidt) Maxim. 회나무 | ○ | Ⅰ | OBU | 5 | 1 | 0 | 2 | 3 | 0 |
| Celastraceae | *Tripterygium regelii* Sprague & Takeda 미역줄나무 |  | Ⅱ | OBU | 37 | 1 | 11 | 1 | 3 | 0 |
| Staphyleaceae | *Euscaphis japonica* (Thunb.) Kanitz 말오줌때 |  | Ⅰ | OBU | 0 | 0 | 0 | 0 | 3 | 0 |
| Staphyleaceae | *Staphylea bumalda* DC. 고추나무 |  |  | FACU | 24 | 5 | 5 | 9 | 7 | 0 |
| Rhamnaceae | *Hovenia dulcis* Thunb. 헛개나무 |  | Ⅰ | OBU | 0 | 0 | 1 | 0 | 0 | 0 |
| Rhamnaceae | *Rhamnella franguloides* (Maxim.) Weberb. 까마귀베개 |  | Ⅰ | OBU | 0 | 0 | 0 | 1 | 1 | 1 |
| Rhamnaceae | *Rhamnus davurica* Pall. 갈매나무 | ○ | Ⅳ | OBU | 1 | 2 | 5 | 0 | 1 | 0 |
| Rhamnaceae | *Rhamnus yoshinoi* Makino 짝자래나무 |  |  | FAC | 1 | 0 | 0 | 1 | 2 | 0 |
| Vitaceae | *Ampelopsis glandulosa* (Wall.) Momiy var. *brevipedunculata* (Maxim.) Momiy 개머루 |  |  | FAC | 22 | 6 | 22 | 6 | 22 | 1 |
| Vitaceae | *Cayratia japonica* (Thunb.) Gagnep. 거지덩굴 |  | Ⅰ | OBU | 0 | 5 | 1 | 0 | 10 | 0 |
| Vitaceae | *Parthenocissus tricuspidata* (Siebold & Zucc.) Planch. 담쟁이덩굴 |  |  | OBU | 22 | 12 | 20 | 20 | 27 | 1 |
| Vitaceae | *Vitis amurensis* Rupr. 왕머루 | ○ |  | OBW | 14 | 6 | 8 | 10 | 4 | 0 |
| Vitaceae | *Vitis coignetiae* Pulliat ex Planch. 머루 |  | Ⅲ | FAC | 6 | 8 | 8 | 2 | 4 | 0 |
| Vitaceae | *Vitis flexuosa* Thunb. 새머루 |  |  | OBU | 9 | 2 | 15 | 7 | 7 | 0 |
| Vitaceae | *Vitis heyneana* Roem. & Schult. subsp. *ficifolia* (Bunge) C.L.Li 까마귀머루 |  |  | OBU | 0 | 0 | 6 | 1 | 11 | 0 |
| Tiliaceae | *Corchoropsis tomentosa* (Thunb.) Makino 수까치깨 |  |  | FACU | 1 | 1 | 3 | 1 | 2 | 0 |
| Tiliaceae | *Grewia biloba* G. Don 장구밥나무 |  | Ⅰ | OBU | 0 | 0 | 0 | 1 | 2 | 0 |
| Tiliaceae | *Tilia amurensis* Rupr. 피나무 | ○ | Ⅱ | FAC | 18 | 0 | 0 | 0 | 0 | 0 |
| Tiliaceae | *Tilia mandshurica* Rupr. & Maxim. 찰피나무 | ○ | Ⅱ | OBU | 5 | 0 | 0 | 1 | 0 | 0 |
| Malvaceae | *Hibiscus trionum* L. 수박풀 |  |  | OBW | 0 | 0 | 1 | 0 | 0 | 0 |
| Violaceae | *Viola acuminata* Ledeb. 졸방제비꽃 | ○ |  | FAC | 37 | 6 | 7 | 9 | 3 | 0 |
| Violaceae | *Viola albida* Palib. 태백제비꽃 |  |  | OBW | 6 | 1 | 3 | 0 | 0 | 0 |
| Violaceae | *Viola albida* Palib. var. *chaerophylloides* (Regel)  F. Maek. ex H. Hara 남산제비꽃 |  |  | FACU | 9 | 6 | 4 | 3 | 6 | 0 |
| Violaceae | *Viola arcuata* Blume 콩제비꽃 |  |  | OBW | 55 | 19 | 34 | 9 | 8 | 8 |
| Violaceae | *Viola collina* Besser 둥근털제비꽃 |  |  | FACU | 14 | 7 | 3 | 5 | 1 | 0 |
| Violaceae | *Viola diamantiaca* Nakai 금강제비꽃 |  | Ⅲ | FAC | 3 | 0 | 0 | 0 | 0 | 0 |
| Violaceae | *Viola hirtipes* S. Moore 흰털제비꽃 | ○ |  | OBU | 0 | 1 | 0 | 0 | 0 | 0 |
| Violaceae | *Viola keiskei* Miq. 잔털제비꽃 |  |  | OBU | 3 | 1 | 3 | 1 | 1 | 0 |
| Violaceae | *Viola lactiflora* Nakai 흰젖제비꽃 |  |  | OBU | 0 | 0 | 0 | 0 | 0 | 6 |
| Violaceae | *Viola mandshurica* W. Becker 제비꽃 | ○ |  | OBU | 8 | 1 | 4 | 5 | 5 | 0 |
| Violaceae | *Viola orientalis* (Maxim.) W. Becker 노랑제비꽃 | ○ | Ⅱ | OBU | 3 | 0 | 0 | 0 | 0 | 0 |
| Violaceae | *Viola patrinii* DC. ex Ging. 흰제비꽃 | ○ |  | OBU | 1 | 0 | 0 | 0 | 0 | 0 |
| Violaceae | *Viola phalacrocarpa* Maxim. 털제비꽃 |  |  | OBU | 1 | 1 | 0 | 0 | 0 | 0 |
| Violaceae | *Viola philippica* Cav. 호제비꽃 |  |  | FAC | 0 | 0 | 0 | 0 | 0 | 1 |
| Violaceae | *Viola rossii* Hemsl. 고깔제비꽃 |  |  | OBU | 9 | 4 | 5 | 6 | 5 | 0 |
| Violaceae | *Viola selkirkii* Pursh ex Goldie 뫼제비꽃 | ○ |  | FAC | 4 | 0 | 3 | 0 | 0 | 0 |
| Violaceae | *Viola seoulensis* Nakai 서울제비꽃 |  |  | FAC | 1 | 0 | 2 | 1 | 1 | 0 |
| Violaceae | *Viola tokubuchiana* Makino var. *takedana* (Makino) F. Maek. 민둥뫼제비꽃 |  | Ⅱ | FACU | 1 | 0 | 1 | 1 | 0 | 0 |
| Violaceae | *Viola variegata* Fisch. ex Link 알록제비꽃 | ○ |  | OBU | 2 | 0 | 0 | 0 | 0 | 0 |
| Aristolochiaceae | *Aristolochia manshuriensis* Kom. 등칡 | ○ | Ⅱ | FAC | 6 | 0 | 0 | 0 | 0 | 0 |
| Aristolochiaceae | *Aristolochia contorta* Bunge 쥐방울덩굴 |  | Ⅰ | OBU | 6 | 3 | 3 | 6 | 5 | 0 |
| Aristolochiaceae | *Asarum sieboldii* Miq. 족도리풀 |  |  | OBU | 23 | 4 | 3 | 5 | 4 | 0 |
| Cucurbitaceae | *Actinostemma lobatum* (Maxim.) Maxim. ex Franch. & Sav. 뚜껑덩굴 |  | Ⅰ | OBU | 0 | 0 | 0 | 1 | 0 | 0 |
| Cucurbitaceae | *Gynostemma pentaphyllum* (Thunb.) Makino 돌외 |  | Ⅰ | FAC | 0 | 1 | 0 | 0 | 0 | 1 |
| Cucurbitaceae | *Melothria japonica* (Thunb.) Maxim. ex Cogn. 새박 |  | Ⅰ | OBU | 0 | 0 | 0 | 0 | 4 | 0 |
| Cucurbitaceae | *Schizopepon bryoniifolius* Maxim. 산외 |  | Ⅳ | OBU | 3 | 0 | 0 | 0 | 0 | 0 |
| Cucurbitaceae | *Sicyos angulatus* L. 가시박 |  |  | OBU | 1 | 0 | 0 | 0 | 0 | 0 |
| Cucurbitaceae | *Trichosanthes kirilowii* Maxim. 하늘타리 |  |  | OBU | 0 | 0 | 4 | 2 | 3 | 0 |
| Thymelaeaceae | *Edgeworthia chrysantha* Lindl. 삼지닥나무 |  |  | OBU | 0 | 0 | 1 | 0 | 0 | 0 |
| Elaeagnaceae | *Elaeagnus glabra* Thunb. 보리장나무 |  | Ⅲ | OBU | 0 | 3 | 0 | 0 | 0 | 0 |
| Elaeagnaceae | *Elaeagnus umbellata* Thunb. 보리수나무 |  |  | OBU | 10 | 3 | 22 | 11 | 9 | 1 |
| Lythraceae | *Lythrum salicaria* L. 털부처꽃 |  |  | OBU | 3 | 0 | 2 | 0 | 0 | 4 |
| Lythraceae | *Lythrum salicaria* L. subsp. *anceps* (Koehne) H. Hara 부처꽃 |  |  | OBW | 7 | 1 | 8 | 2 | 1 | 0 |
| Trapaceae | *Trapa japonica* Flerow 마름 |  |  | OBU | 0 | 0 | 2 | 0 | 0 | 9 |
| Onagraceae | *Circaea alpina* L. 쥐털이슬 |  | Ⅱ | OBU | 1 | 1 | 0 | 0 | 0 | 0 |
| Onagraceae | *Circaea cordata* Royle 쇠털이슬 |  | Ⅰ | OBU | 0 | 0 | 1 | 0 | 0 | 0 |
| Onagraceae | *Circaea lutetiana* L. subsp. *quadrisulcata* (Maxim.) Asch. & Magnus 말털이슬 |  | Ⅱ | OBU | 3 | 0 | 7 | 0 | 0 | 0 |
| Onagraceae | *Circaea mollis* Siebold & Zucc. 털이슬 |  |  | OBU | 6 | 3 | 4 | 4 | 3 | 0 |
| Onagraceae | *Epilobium amurense* Hausskn. subsp. *cephalostigma* (Hausskn.) C. J. Chen & Hoch & P. H. Raven 돌바늘꽃 |  |  | OBU | 5 | 0 | 1 | 1 | 1 | 0 |
| Onagraceae | *Epilobium palustre* L. 버들바늘꽃 | ○ | Ⅳ | OBU | 2 | 0 | 0 | 0 | 0 | 0 |
| Onagraceae | *Epilobium pyrricholophum* Franch. & Sav. 바늘꽃 |  |  | FACW | 5 | 0 | 5 | 2 | 10 | 0 |
| Onagraceae | *Ludwigia ovalis* Miq. 눈여뀌바늘 |  | Ⅲ | OBU | 0 | 0 | 0 | 0 | 0 | 6 |
| Onagraceae | *Ludwigia prostrata* Roxb. 여뀌바늘 |  |  | FACU | 1 | 1 | 1 | 2 | 2 | 0 |
| Onagraceae | *Oenothera biennis* L. 달맞이꽃 |  |  | OBW | 11 | 4 | 7 | 10 | 5 | 0 |
| Haloragaceae | *Haloragis micrantha* (Thunb.) R.Br. ex Siebold & Zucc. 개미탑 |  | Ⅰ | OBU | 0 | 1 | 2 | 0 | 13 | 3 |
| Alangiaceae | *Alangium platanifolium* (Siebold & Zucc.) Harms  단풍박쥐나무 |  |  | FACU | 0 | 0 | 1 | 0 | 0 | 0 |
| Alangiaceae | *Alangium platanifolium* (Siebold & Zucc.) Harms var. *trilobum* (Miq.) Ohwi 박쥐나무 |  |  | OBU | 3 | 2 | 1 | 6 | 3 | 0 |
| Cornaceae | *Cornus controversa* Hemsl. 층층나무 |  |  | OBU | 37 | 7 | 13 | 6 | 18 | 1 |
| Cornaceae | *Cornus kousa* Burger ex Hance 산딸나무 |  |  | FACW | 0 | 2 | 2 | 0 | 3 | 1 |
| Cornaceae | *Cornus macrophylla* Wall. 곰의말채나무 |  |  | OBU | 0 | 0 | 0 | 0 | 2 | 0 |
| Cornaceae | *Cornus officinalis* Siebold & Zucc. 산수유 |  |  | OBW | 2 | 3 | 3 | 0 | 1 | 0 |
| Cornaceae | *Cornus walteri* Wangerin 말채나무 |  |  | FACU | 0 | 1 | 4 | 1 | 2 | 0 |
| Araliaceae | *Aralia cordata* Thunb. var. *continentalis* (Kitag.) Y. C. Chu 독활 | ○ |  | OBU | 5 | 1 | 2 | 2 | 1 | 0 |
| Araliaceae | *Aralia elata* (Miq.) Seem. 두릅나무 |  |  | OBU | 27 | 11 | 13 | 6 | 12 | 0 |
| Araliaceae | *Dendropanax trifidus* (Thunb.) Makino ex H. Hara 황칠나무 |  | Ⅲ | OBU | 0 | 0 | 0 | 0 | 0 | 1 |
| Araliaceae | *Eleutherococcus divaricatus* (Siebold & Zucc.) S. Y. Hu var. *chiisanensis* (Nakai) C. H. Kim & B. Y. Sun 지리산오갈피 |  | Ⅲ | OBU | 6 | 0 | 0 | 2 | 0 | 0 |
| Araliaceae | *Eleutherococcus senticosus* (Rupr. & Maxim.) Maxim. 가시오갈피 | ○ |  | OBU | 1 | 0 | 1 | 0 | 0 | 0 |
| Araliaceae | *Eleutherococcus sessiliflorus* (Rupr. & Maxim.) S. Y. Hu 오갈피나무 | ○ | Ⅰ | FACW | 37 | 11 | 8 | 6 | 6 | 0 |
| Araliaceae | *Hedera rhombea* (Miq.) Siebold & Zucc. ex Bean 송악 |  | Ⅰ | OBU | 0 | 0 | 0 | 0 | 2 | 0 |
| Araliaceae | *Kalopanax septemlobus* (Thunb.) Koidz. 음나무 |  |  | FAC | 19 | 3 | 3 | 2 | 2 | 0 |
| Apiaceae | *Aegopodium alpestre* Ledeb. 왜방풍 | ○ | Ⅳ | OBU | 0 | 0 | 0 | 1 | 0 | 0 |
| Apiaceae | *Angelica anomala* Avé-Lall. 개구릿대 | ○ | Ⅰ | FAC | 4 | 0 | 0 | 0 | 0 | 0 |
| Apiaceae | *Angelica cartilaginomarginata* (Makino ex Y. Yabe) Nakai 처녀바디 |  |  | OBU | 0 | 0 | 4 | 0 | 1 | 0 |
| Apiaceae | *Angelica cartilaginomarginata* (Makino ex Y. Yabe) Nakai var. *distans* (Nakai) Kitag. 흰바디나물 |  |  | FACU | 3 | 0 | 0 | 0 | 0 | 0 |
| Apiaceae | *Angelica czernaevia* (Fisch. & C. A. Mey.) Kitag. 잔잎바디 |  |  | OBU | 0 | 0 | 1 | 0 | 0 | 0 |
| Apiaceae | *Angelica dahurica* (Fisch. ex Hoffm.) Benth. & Hook. f. ex Franch. & Sav. 구릿대 |  |  | FACW | 9 | 1 | 5 | 1 | 1 | 0 |
| Apiaceae | *Angelica decursiva* (Miq.) Franch. & Sav. 바디나물 |  |  | FACW | 44 | 12 | 13 | 15 | 19 | 0 |
| Apiaceae | *Angelica gigas* Nakai 참당귀 |  |  | OBU | 32 | 1 | 5 | 9 | 2 | 0 |
| Apiaceae | *Angelica polymorpha* Maxim. 궁궁이 |  |  | FACU | 20 | 1 | 9 | 4 | 6 | 0 |
| Apiaceae | *Angelica purpuraefolia* T. H. Chung 지리강활 |  |  | OBU | 8 | 0 | 3 | 0 | 1 | 0 |
| Apiaceae | *Angelica reflexa* B. Y. Lee 강활 |  |  | OBU | 14 | 1 | 0 | 0 | 0 | 0 |
| Apiaceae | *Anthriscus sylvestris* (L.) Hoffm. 전호 |  |  | OBU | 5 | 1 | 0 | 0 | 0 | 0 |
| Apiaceae | *Bupleurum longiradiatum* Turcz. 개시호 | ○ | Ⅱ | OBU | 2 | 0 | 1 | 0 | 1 | 0 |
| Apiaceae | *Centella asiatica* (L.) Urb. 병풀 |  | Ⅲ | FAC | 4 | 2 | 0 | 0 | 0 | 1 |
| Apiaceae | *Cicuta virosa* L. 독미나리 | ○ | Ⅴ | OBU | 5 | 0 | 0 | 0 | 0 | 0 |
| Apiaceae | *Cnidium japonicum* Miq. 갯사상자 |  | Ⅰ | OBU | 0 | 0 | 0 | 0 | 1 | 0 |
| Apiaceae | *Cryptotaenia japonica* Hassk. 파드득나물 |  |  | OBU | 5 | 2 | 0 | 0 | 4 | 0 |
| Apiaceae | *Cymopterus melanotilingia* (H. Boissieu) C. Y. Yoon 큰참나물 |  | Ⅱ | FAC | 4 | 1 | 2 | 0 | 0 | 0 |
| Apiaceae | *Heracleum moellendorffii* Hance 어수리 |  |  | OBU | 5 | 1 | 1 | 2 | 2 | 0 |
| Apiaceae | *Hydrocotyle maritima* Honda 선피막이 |  | Ⅰ | OBW | 0 | 0 | 0 | 0 | 0 | 1 |
| Apiaceae | *Hydrocotyle ramiflora* Maxim. 큰피막이 |  |  | FACW | 0 | 0 | 0 | 0 | 3 | 1 |
| Apiaceae | *Hydrocotyle sibthorpioides* Lam. 피막이 |  | Ⅳ | OBU | 5 | 0 | 2 | 0 | 4 | 7 |
| Apiaceae | *Hydrocotyle yabei* Makino 제주피막이 |  | Ⅳ | OBU | 0 | 0 | 0 | 0 | 0 | 1 |
| Apiaceae | *Oenanthe javanica* DC. 미나리 |  |  | FACW | 28 | 8 | 21 | 12 | 21 | 2 |
| Apiaceae | *Osmorhiza aristata* (Thunb.) Rydb. 긴사상자 |  |  | FAC | 0 | 0 | 1 | 0 | 2 | 0 |
| Apiaceae | *Ostericum grosseserratum* (Maxim.) Kitag. 신감채 |  |  | OBU | 3 | 1 | 0 | 4 | 2 | 0 |
| Apiaceae | *Ostericum maximowiczii* (F. Schmidt) Kitag. 가는바디 | ○ | Ⅳ | FAC | 1 | 0 | 0 | 0 | 0 | 0 |
| Apiaceae | *Ostericum sieboldii* (Miq.) Nakai 묏미나리 |  |  | FACW | 1 | 1 | 6 | 4 | 5 | 0 |
| Apiaceae | *Peucedanum terebinthaceum* (Fisch. ex Trevir.) Fisch. ex Turcz. 기름나물 | ○ |  | OBU | 8 | 0 | 2 | 1 | 2 | 0 |
| Apiaceae | *Pimpinella brachycarpa* (Kom.) Nakai 참나물 |  |  | FAC | 29 | 2 | 3 | 3 | 7 | 0 |
| Apiaceae | *Pimpinella komarovii* (Kitag.) R. H. Shan & F. T. Pu 노루참나물 |  |  | OBU | 0 | 0 | 1 | 0 | 0 | 0 |
| Apiaceae | *Sanicula chinensis* Bunge 참반디 |  |  | OBW | 16 | 6 | 8 | 3 | 6 | 0 |
| Apiaceae | *Sium ninsi* L. 감자개발나물 |  |  | OBU | 15 | 5 | 20 | 2 | 22 | 1 |
| Apiaceae | *Sium suave* Walter 개발나물 | ○ |  | OBU | 23 | 5 | 15 | 6 | 18 | 1 |
| Apiaceae | *Torilis japonica* (Houtt.) DC. 사상자 |  |  | OBU | 7 | 0 | 12 | 2 | 4 | 0 |
| Ericaceae | *Pyrola japonica* Klenze ex Alef. 노루발 | ○ |  | OBU | 5 | 0 | 7 | 3 | 7 | 0 |
| Ericaceae | *Rhododendron micranthum* Turcz. 꼬리진달래 |  | Ⅳ | FAC | 1 | 1 | 0 | 1 | 0 | 0 |
| Ericaceae | *Rhododendron mucronulatum* Turcz. 진달래 | ○ |  | OBW | 15 | 3 | 12 | 5 | 22 | 0 |
| Ericaceae | *Rhododendron mucronulatum* Turcz. var. *ciliatum* Nakai 털진달래 |  |  | FACU | 4 | 0 | 4 | 0 | 0 | 0 |
| Ericaceae | *Rhododendron schlippenbachii* Maxim. 철쭉 | ○ |  | OBU | 21 | 2 | 10 | 1 | 2 | 0 |
| Ericaceae | *Rhododendron yedoense* Maxim. f. *poukhanense* (H. Lév.) Sugim. ex T. Yamaz. 산철쭉 |  |  | FACU | 1 | 0 | 4 | 2 | 18 | 1 |
| Ericaceae | *Vaccinium oldhamii* Miq. 정금나무 |  | Ⅰ | FACU | 0 | 0 | 0 | 0 | 5 | 0 |
| Myrsinaceae | *Ardisia japonica* (Thunb.) Blume 자금우 |  | Ⅰ | OBU | 0 | 0 | 0 | 0 | 0 | 2 |
| Primulaceae | *Lysimachia barystachys* Bunge 까치수염 | ○ | Ⅰ | OBU | 2 | 0 | 2 | 0 | 2 | 0 |
| Primulaceae | *Lysimachia clethroides* Duby 큰까치수염 |  |  | OBU | 28 | 8 | 13 | 13 | 25 | 0 |
| Primulaceae | *Lysimachia coreana* Nakai 참좁쌀풀 |  | Ⅳ | OBU | 5 | 0 | 2 | 0 | 1 | 0 |
| Primulaceae | *Lysimachia japonica* Thunb. 좀가지풀 |  |  | FACW | 0 | 0 | 0 | 0 | 3 | 2 |
| Primulaceae | *Lysimachia vulgaris* L. var. *davurica* (Ledeb.) R. Knuth 좁쌀풀 |  | Ⅲ | OBU | 44 | 4 | 5 | 5 | 1 | 0 |
| Primulaceae | *Primula jesoana* Miq. 큰앵초 | ○ | Ⅱ | OBW | 5 | 0 | 1 | 0 | 0 | 0 |
| Primulaceae | *Primula sieboldii* E. Morren 앵초 | ○ | Ⅱ | FAC | 0 | 0 | 0 | 2 | 0 | 0 |
| Primulaceae | *Trientalis europaea* L. var. *arctica* (Fisch. ex Hook.) Ledeb. 기생꽃 |  | Ⅴ | OBW | 1 | 0 | 0 | 0 | 0 | 0 |
| Plumbaginaceae | *Limonium tetragonum* (Thunb.) Bullock 갯질경 |  |  | OBU | 0 | 0 | 0 | 0 | 1 | 0 |
| Ebenaceae | *Diospyros kaki* L.f. 감나무 |  |  | OBW | 5 | 0 | 4 | 0 | 1 | 0 |
| Ebenaceae | *Diospyros lotus* L. 고욤나무 |  |  | OBU | 12 | 5 | 23 | 10 | 6 | 0 |
| Styracaceae | *Styrax japonicus* Siebold & Zucc. 때죽나무 |  |  | OBU | 5 | 1 | 29 | 9 | 51 | 1 |
| Styracaceae | *Styrax obassis* Siebold & Zucc. 쪽동백나무 |  |  | OBU | 13 | 5 | 5 | 9 | 5 | 0 |
| Symplocaceae | *Symplocos sawafutagi* Nagam. 노린재나무 |  |  | OBU | 40 | 12 | 8 | 11 | 22 | 1 |
| Symplocaceae | *Symplocos tanakana* Nakai 검노린재나무 |  |  | OBU | 0 | 0 | 2 | 0 | 8 | 3 |
| Oleaceae | *Chionanthus retusus* Lindl. & Paxton 이팝나무 |  | Ⅲ | OBU | 0 | 0 | 0 | 0 | 1 | 0 |
| Oleaceae | *Forsythia koreana* (Rehder) Nakai 개나리 |  |  | FACU | 1 | 0 | 1 | 0 | 0 | 0 |
| Oleaceae | *Fraxinus chiisanensis* Nakai 물들메나무 |  | Ⅲ | FAC | 7 | 0 | 0 | 3 | 1 | 0 |
| Oleaceae | *Fraxinus mandshurica* Rupr. 들메나무 | ○ | Ⅰ | FAC | 43 | 1 | 3 | 3 | 1 | 0 |
| Oleaceae | *Fraxinus rhynchophylla* Hance 물푸레나무 |  |  | FACW | 83 | 13 | 32 | 19 | 23 | 0 |
| Oleaceae | *Fraxinus sieboldiana* Blume 쇠물푸레나무 |  |  | FAC | 7 | 0 | 9 | 4 | 12 | 0 |
| Oleaceae | *Ligustrum obtusifolium* Siebold & Zucc. 쥐똥나무 |  |  | FAC | 34 | 29 | 59 | 29 | 52 | 6 |
| Oleaceae | *Ligustrum ovalifolium* Hassk. 왕쥐똥나무 |  | Ⅲ | FAC | 0 | 1 | 0 | 1 | 1 | 0 |
| Oleaceae | *Ligustrum quihoui* Carrière var. *latifolium* Nakai  상동잎쥐똥나무 |  |  | OBU | 0 | 0 | 1 | 0 | 6 | 0 |
| Oleaceae | *Osmanthus heterophyllus* (G. Don) P. S. Green. 구골나무 |  | Ⅳ | OBU | 0 | 0 | 1 | 0 | 0 | 0 |
| Oleaceae | *Syringa pubescens* Turcz. subsp. *patula* (Palib.) M. C. Chang & X. L. Chen 털개회나무 |  | Ⅰ | FACW | 1 | 0 | 0 | 1 | 0 | 0 |
| Oleaceae | *Syringa reticulata* (Blume) H. Hara 개회나무 | ○ | Ⅲ | FACW | 10 | 0 | 0 | 0 | 0 | 0 |
| Oleaceae | *Syringa villosa* Vahl subsp. *wolfii* (C. K. Schneid.) Y. Chen & D. Y. Hong 꽃개회나무 | ○ | Ⅳ | FACU | 5 | 0 | 0 | 0 | 0 | 0 |
| Gentianaceae | *Gentiana scabra* Bunge 용담 | ○ |  | OBU | 0 | 0 | 4 | 1 | 3 | 0 |
| Gentianaceae | *Gentiana squarrosa* Ledeb. 구슬붕이 |  |  | OBU | 0 | 0 | 0 | 0 | 0 | 1 |
| Gentianaceae | *Gentiana triflora* Pall. var. *japonica* (Kusn.) H. Hara  과남풀 | ○ |  | FACW | 14 | 2 | 0 | 2 | 0 | 0 |
| Gentianaceae | *Swertia diluta* (Turcz.) Benth. & Hook. f. var. *tosaensis* (Makino) H. Hara 개쓴풀 |  | Ⅱ | OBU | 0 | 0 | 0 | 0 | 4 | 1 |
| Gentianaceae | *Swertia pseudochinensis* H. Hara 자주쓴풀 |  |  | FAC | 0 | 0 | 1 | 1 | 0 | 0 |
| Gentianaceae | *Tripterospermum japonicum* (Siebold & Zucc.) Maxim. 덩굴용담 |  | Ⅳ | FACU | 0 | 0 | 0 | 0 | 0 | 1 |
| Apocynaceae | *Apocynum cannabinum* L. 수궁초 |  |  | OBW | 0 | 0 | 0 | 0 | 0 | 1 |
| Apocynaceae | *Cynanchum amplexicaule* (Siebold & Zucc.) Hemsl. 솜아마존 |  | Ⅲ | OBW | 0 | 0 | 0 | 0 | 0 | 5 |
| Apocynaceae | *Cynanchum ascyrifolium* (Franch. & Sav.) Matsum. 민백미꽃 |  | Ⅰ | FAC | 2 | 0 | 1 | 2 | 0 | 0 |
| Apocynaceae | *Cynanchum nipponicum* Matsum. 덩굴박주가리 |  | Ⅲ | OBU | 13 | 4 | 2 | 2 | 0 | 0 |
| Apocynaceae | *Cynanchum nipponicum* Matsum. var. *glabrum* (Nakai) H. Hara 흑박주가리 |  | Ⅲ | OBW | 0 | 0 | 0 | 1 | 0 | 0 |
| Apocynaceae | *Cynanchum paniculatum* (Bunge) Kitag. ex H. Hara 산해박 |  |  | OBU | 2 | 0 | 2 | 1 | 0 | 0 |
| Apocynaceae | *Metaplexis japonica* (Thunb.) Makino 박주가리 |  |  | OBU | 9 | 1 | 10 | 5 | 5 | 1 |
| Apocynaceae | *Trachelospermum asiaticum* (Siebold & Zucc.) Nakai 마삭줄 |  |  | OBU | 0 | 0 | 1 | 0 | 5 | 1 |
| Apocynaceae | *Tylophora floribunda* Miq. 왜박주가리 |  | Ⅰ | OBU | 4 | 0 | 1 | 0 | 1 | 0 |
| Rubiaceae | *Asperula lasiantha* Nakai 갈퀴아재비 |  | Ⅴ | FACW | 4 | 0 | 1 | 1 | 0 | 0 |
| Rubiaceae | *Galium bungei* Steud. var. *trachyspermum* (A. Gray) Cufod. 네잎갈퀴 |  |  | OBU | 1 | 1 | 2 | 0 | 1 | 0 |
| Rubiaceae | *Galium dahuricum* Turcz. ex Ledeb. 큰잎갈퀴 | ○ |  | OBU | 2 | 1 | 0 | 0 | 2 | 0 |
| Rubiaceae | *Galium gracilens* (A. Gray) Makino 좀네잎갈퀴 |  |  | OBU | 0 | 0 | 1 | 0 | 1 | 1 |
| Rubiaceae | *Galium koreanum* (Nakai) Nakai 참갈퀴덩굴 |  |  | OBU | 0 | 0 | 0 | 1 | 0 | 0 |
| Rubiaceae | *Galium maximowiczii* (Kom.) Pobed. 개갈퀴 |  |  | OBU | 5 | 0 | 1 | 0 | 0 | 0 |
| Rubiaceae | *Galium odoratum* (L.) Scop. 선갈퀴 |  | Ⅲ | FACW | 3 | 2 | 0 | 1 | 0 | 0 |
| Rubiaceae | *Galium pogonanthum* Franch. & Sav. 산갈퀴 |  |  | FAC | 5 | 0 | 1 | 0 | 1 | 0 |
| Rubiaceae | *Galium spurium* L. 갈퀴덩굴 |  |  | FACW | 18 | 4 | 3 | 5 | 2 | 0 |
| Rubiaceae | *Galium tokyoense* Makino 흰갈퀴 |  |  | OBW | 0 | 2 | 0 | 0 | 0 | 0 |
| Rubiaceae | *Galium trifidum* L. 가는네잎갈퀴 |  |  | FAC | 3 | 0 | 3 | 0 | 1 | 1 |
| Rubiaceae | *Galium trifloriforme* Kom. 개선갈퀴 |  |  | FAC | 0 | 0 | 0 | 1 | 0 | 0 |
| Rubiaceae | *Galium verum* L. 솔나물 |  |  | OBW | 6 | 0 | 9 | 0 | 3 | 0 |
| Rubiaceae | *Neanotis hirsuta* (L.f.) W.H.Lewis 탐라풀 |  | Ⅲ | FACU | 0 | 0 | 0 | 0 | 0 | 1 |
| Rubiaceae | *Paederia foetida* L. 계요등 |  | Ⅰ | OBU | 1 | 0 | 15 | 4 | 45 | 1 |
| Rubiaceae | *Rubia argyi* (H. Lév. & Vaniot) H. Hara ex Lauener 꼭두서니 |  |  | FACW | 46 | 25 | 39 | 27 | 18 | 0 |
| Rubiaceae | *Rubia chinensis* Regel & Maack 큰꼭두서니 | ○ | Ⅱ | OBU | 4 | 2 | 2 | 0 | 1 | 0 |
| Rubiaceae | *Rubia cordifolia* L. 갈퀴꼭두서니 |  |  | FACU | 23 | 7 | 7 | 8 | 4 | 0 |
| Rubiaceae | *Rubia hexaphylla* (Makino) Makino 가지꼭두서니 |  |  | OBU | 0 | 0 | 1 | 0 | 1 | 0 |
| Convolvulaceae | *Calystegia pubescens* Lindl. 메꽃 |  |  | FACU | 2 | 0 | 3 | 0 | 0 | 0 |
| Convolvulaceae | *Calystegia sepium* (L.) R.Br. 큰메꽃 |  |  | FAC | 1 | 0 | 0 | 0 | 0 | 0 |
| Convolvulaceae | *Cuscuta australis* R.Br. 실새삼 |  |  | OBU | 1 | 0 | 4 | 1 | 0 | 0 |
| Convolvulaceae | *Cuscuta japonica* Choisy 새삼 |  |  | OBU | 3 | 0 | 0 | 0 | 2 | 0 |
| Convolvulaceae | *Cuscuta pentagona* Engelm. 미국실새삼 |  |  | FACU | 1 | 0 | 1 | 0 | 0 | 0 |
| Convolvulaceae | *Ipomoea nil* (L.) Roth 나팔꽃 |  |  | OBU | 0 | 0 | 0 | 0 | 1 | 0 |
| Convolvulaceae | *Quamoclit angulata* (Lam.) Bojer 둥근잎유홍초 |  |  | OBU | 0 | 0 | 0 | 0 | 1 | 0 |
| Boraginaceae | *Bothriospermum tenellum* (Hornem.) Fisch. & C. A. Mey.꽃받이 |  |  | OBU | 1 | 0 | 1 | 1 | 0 | 0 |
| Boraginaceae | *Brachybotrys paridiformis* Maxim. ex Oliv. 당개지치 | ○ | Ⅲ | OBU | 12 | 1 | 0 | 0 | 0 | 0 |
| Boraginaceae | *Symphytum officinale* L. 컴프리 |  |  | FAC | 0 | 0 | 1 | 0 | 0 | 0 |
| Boraginaceae | *Trigonotis icumae* (Maxim.) Makino 덩굴꽃마리 |  | Ⅰ | FACW | 3 | 1 | 3 | 1 | 2 | 0 |
| Boraginaceae | *Trigonotis peduncularis* (Trevis.) Benth. ex Baker & S. Moore 꽃마리 |  |  | OBU | 2 | 0 | 1 | 0 | 0 | 0 |
| Boraginaceae | *Trigonotis radicans* (Turcz.) Steven 거센털꽃마리 | ○ | Ⅲ | FACU | 0 | 0 | 1 | 0 | 0 | 0 |
| Boraginaceae | *Trigonotis radicans* (Turcz.) Steven var. *sericea* (Maxim.) H. Hara 참꽃마리 |  |  | FACU | 7 | 2 | 5 | 3 | 3 | 0 |
| Verbenaceae | *Callicarpa dichotoma* (Lour.) Raeusch. ex K. Koch 좀작살나무 |  | Ⅲ | OBU | 0 | 0 | 0 | 1 | 0 | 0 |
| Verbenaceae | *Callicarpa japonica* Thunb. 작살나무 |  |  | OBU | 28 | 17 | 18 | 18 | 32 | 0 |
| Verbenaceae | *Callicarpa japonica* Thunb. var. *luxurians* Rehder 왕작살나무 |  |  | FACU | 0 | 0 | 0 | 0 | 1 | 0 |
| Verbenaceae | *Callicarpa mollis* Siebold & Zucc. 새비나무 |  | Ⅲ | FAC | 0 | 0 | 0 | 0 | 1 | 0 |
| Verbenaceae | *Caryopteris incana* (Thunb. ex Houtt.) Miq. 층꽃나무 |  | Ⅰ | FACW | 0 | 0 | 0 | 0 | 1 | 0 |
| Verbenaceae | *Clerodendrum trichotomum* Thunb. 누리장나무 |  |  | OBU | 12 | 3 | 9 | 4 | 5 | 0 |
| Verbenaceae | *Tripora divaricata* (Maxim.) P. D. Cantino 누린내풀 |  |  | FACU | 0 | 0 | 0 | 1 | 0 | 0 |
| Verbenaceae | *Vitex rotundifolia* L.f. 순비기나무 |  | Ⅱ | OBU | 0 | 0 | 0 | 0 | 1 | 0 |
| Lamiaceae | *Agastache rugosa* (Fisch. & C. A. Mey.) Kuntze 배초향 |  |  | OBU | 3 | 1 | 0 | 2 | 0 | 0 |
| Lamiaceae | *Ajuga decumbens* Thunb. 금창초 |  | Ⅰ | OBU | 0 | 0 | 0 | 0 | 1 | 0 |
| Lamiaceae | *Clinopodium chinense* (Benth.) Kuntze var. *parviflorum* (Kudô) H. Hara 층층이꽃 |  |  | OBU | 2 | 0 | 2 | 1 | 8 | 0 |
| Lamiaceae | *Clinopodium chinense* (Benth.) Kuntze var. *shibetchense* (H. Lév.) Koidz. 산층층이 |  |  | OBU | 0 | 0 | 0 | 1 | 1 | 0 |
| Lamiaceae | *Clinopodium gracile* (Benth.) Kuntze 애기탑꽃 |  |  | FAC | 1 | 11 | 0 | 13 | 5 | 0 |
| Lamiaceae | *Clinopodium micranthum* (Regel) H. Hara 두메층층이 |  |  | FACU | 1 | 1 | 3 | 4 | 0 | 1 |
| Lamiaceae | *Clinopodium multicaule* (Maxim.) Kuntze 탑꽃 |  |  | OBU | 2 | 0 | 7 | 5 | 13 | 0 |
| Lamiaceae | *Clinopodium sachalinense* (F. Schmidt) Koidz. 두메탑풀 |  |  | FACU | 6 | 5 | 11 | 5 | 0 | 0 |
| Lamiaceae | *Elsholtzia ciliata* (Thunb.) Hyl. 향유 |  |  | OBU | 3 | 4 | 5 | 5 | 1 | 0 |
| Lamiaceae | *Elsholtzia splendens* Nakai ex Maekawa 꽃향유 |  |  | OBU | 5 | 1 | 8 | 2 | 2 | 0 |
| Lamiaceae | *Isodon excisus* (Maxim.) Kudô 오리방풀 | ○ |  | OBU | 61 | 12 | 22 | 6 | 6 | 0 |
| Lamiaceae | *Isodon inflexus* (Thunb.) Kudô 산박하 |  |  | FAC | 25 | 12 | 19 | 7 | 23 | 0 |
| Lamiaceae | *Isodon japonicus* (Burm.f.) H. Hara 방아풀 |  |  | OBW | 4 | 0 | 3 | 3 | 1 | 0 |
| Lamiaceae | *Lamium album* L. subsp. *barbatum* (Siebold & Zucc.) Mennema 광대수염 |  |  | FACU | 7 | 0 | 0 | 2 | 1 | 0 |
| Lamiaceae | *Leonurus japonicus* Houtt. 익모초 |  |  | OBU | 1 | 0 | 5 | 3 | 0 | 1 |
| Lamiaceae | *Leonurus macranthus* Maxim. 송장풀 | ○ |  | FAC | 0 | 0 | 2 | 0 | 2 | 0 |
| Lamiaceae | *Lycopus charkeviczii* Prob. 산쉽싸리 |  |  | FACU | 35 | 12 | 8 | 7 | 7 | 0 |
| Lamiaceae | *Lycopus coreanus* H. Lév. 개쉽싸리 |  | Ⅱ | OBU | 6 | 0 | 0 | 1 | 0 | 0 |
| Lamiaceae | *Lycopus lucidus* Turcz. ex Benth. 쉽싸리 |  |  | OBU | 62 | 14 | 39 | 17 | 22 | 0 |
| Lamiaceae | *Lycopus maackianus* (Maxim. ex Herder) Makino 애기쉽싸리 |  | Ⅰ | FAC | 14 | 2 | 12 | 2 | 5 | 0 |
| Lamiaceae | *Lycopus uniflorus* Michx. 털쉽싸리 |  | Ⅱ | FACU | 1 | 0 | 2 | 0 | 0 | 0 |
| Lamiaceae | *Meehania urticifolia* (Miq.) Makino 벌깨덩굴 |  |  | OBU | 26 | 4 | 5 | 4 | 2 | 0 |
| Lamiaceae | *Mentha arvensis* L. var. *piperascens* Malinv. ex Holmes 박하 |  |  | FACU | 3 | 0 | 4 | 1 | 0 | 0 |
| Lamiaceae | *Mosla dianthera* (Buch.-Ham. ex Roxb.) Maxim. 쥐깨풀 |  |  | OBU | 15 | 9 | 24 | 7 | 14 | 2 |
| Lamiaceae | *Mosla japonica* (Benth. ex Oliv.) Maxim. 산들깨 |  |  | OBU | 0 | 0 | 0 | 0 | 1 | 0 |
| Lamiaceae | *Mosla scabra* (Thunb.) C. Y. Wu & H. W. Li 들깨풀 |  |  | FAC | 9 | 2 | 6 | 3 | 5 | 0 |
| Lamiaceae | *Nepeta cataria* L. 개박하 |  |  | OBW | 0 | 0 | 1 | 0 | 2 | 0 |
| Lamiaceae | *Perilla frutescens* (L.) Britton 들깨 |  |  | FACU | 1 | 4 | 3 | 2 | 0 | 0 |
| Lamiaceae | *Phlomis umbrosa* Turcz. 속단 |  |  | FACW | 2 | 1 | 4 | 1 | 0 | 0 |
| Lamiaceae | *Prunella vulgaris* L. subsp. *asiatica* (Nakai) H. Hara 꿀풀 |  |  | FACW | 13 | 1 | 6 | 2 | 4 | 1 |
| Lamiaceae | *Salvia japonica* Thunb. 둥근배암차즈기 |  | Ⅲ | FAC | 0 | 0 | 1 | 0 | 2 | 0 |
| Lamiaceae | *Salvia plebeia* R.Br. 배암차즈기 |  |  | OBU | 0 | 0 | 3 | 1 | 1 | 0 |
| Lamiaceae | *Scutellaria dependens* Maxim. 애기골무꽃 | ○ | Ⅱ | FACW | 32 | 12 | 18 | 3 | 3 | 0 |
| Lamiaceae | *Scutellaria indica* L. 골무꽃 |  |  | OBU | 1 | 2 | 1 | 1 | 2 | 0 |
| Lamiaceae | *Scutellaria insignis Nakai* 광릉골무꽃 |  | Ⅲ | OBU | 0 | 0 | 0 | 1 | 3 | 0 |
| Lamiaceae | *Scutellaria pekinensis* Maxim. var. *transitra* (Makino) H. Hara 산골무꽃 |  |  | FACW | 2 | 2 | 2 | 1 | 1 | 0 |
| Lamiaceae | *Scutellaria strigillosa* Hemsl. 참골무꽃 | ○ | Ⅰ | FAC | 1 | 0 | 0 | 0 | 0 | 0 |
| Lamiaceae | *Stachys oblongifolia* Benth. 우단석잠풀 |  |  | FACW | 0 | 0 | 1 | 0 | 0 | 0 |
| Lamiaceae | *Stachys riederi* Cham. var. *japonica* (Miq.) H. Hara 석잠풀 |  |  | OBU | 4 | 1 | 3 | 0 | 0 | 0 |
| Lamiaceae | *Teucrium japonicum* Houtt. 개곽향 |  |  | FACW | 1 | 0 | 3 | 1 | 1 | 0 |
| Lamiaceae | *Teucrium veronicoides* Maxim. 곽향 |  | Ⅲ | FAC | 0 | 0 | 0 | 0 | 1 | 0 |
| Lamiaceae | *Teucrium viscidum* Blume var. *miquelianum* (Maxim.) H. Hara 덩굴곽향 |  |  | OBW | 0 | 1 | 1 | 0 | 1 | 0 |
| Solanaceae | *Lycium chinense* Mill. 구기자나무 |  |  | OBU | 0 | 1 | 0 | 1 | 0 | 0 |
| Solanaceae | *Physaliastrum echinatum* (Yatabe) Makino 가시꽈리 |  |  | FACU | 2 | 0 | 0 | 1 | 0 | 0 |
| Solanaceae | *Physalis alkekengi* L. 꽈리 |  |  | OBU | 1 | 1 | 0 | 1 | 0 | 0 |
| Solanaceae | *Solanum carolinense* L. 도깨비가지 |  |  | FACU | 0 | 0 | 0 | 0 | 0 | 1 |
| Solanaceae | *Solanum lyratum* Thunb. 배풍등 |  |  | OBU | 0 | 2 | 5 | 2 | 1 | 0 |
| Solanaceae | *Solanum nigrum* L. 까마중 |  |  | OBU | 1 | 3 | 1 | 3 | 1 | 0 |
| Scrophulariaceae | *Deinostema violacea* (Maxim.) T. Yamaz. 진땅고추풀 |  | Ⅱ | FACW | 0 | 1 | 0 | 0 | 0 | 1 |
| Scrophulariaceae | *Euphrasia maximowiczii* Wettst. ex Palib. 앉은좁쌀풀 |  | Ⅲ | OBW | 0 | 0 | 1 | 0 | 0 | 0 |
| Scrophulariaceae | *Limnophila sessiliflora* (Vahl) Blume 구와말 |  | Ⅱ | OBW | 0 | 0 | 0 | 0 | 0 | 1 |
| Scrophulariaceae | *Lindernia crustacea* (L.) F. Muell. 외풀 |  |  | FACU | 0 | 0 | 0 | 0 | 1 | 0 |
| Scrophulariaceae | *Lindernia micrantha* D. Don 논뚝외풀 |  |  | FACW | 2 | 0 | 1 | 1 | 1 | 0 |
| Scrophulariaceae | *Lindernia procumbens* (Krock.) Philcox 밭뚝외풀 |  |  | FAC | 2 | 0 | 1 | 1 | 0 | 0 |
| Scrophulariaceae | *Mazus pumilus* (Burm.f.) Steenis 주름잎 |  |  | FAC | 2 | 0 | 1 | 1 | 1 | 3 |
| Scrophulariaceae | *Melampyrum roseum* Maxim. 꽃며느리밥풀 | ○ |  | FACU | 2 | 0 | 0 | 0 | 1 | 0 |
| Scrophulariaceae | *Microcarpaea minima* (K. D. Koenig ex Retz.) Merr. 진흙풀 |  | Ⅱ | OBW | 0 | 0 | 0 | 0 | 0 | 1 |
| Scrophulariaceae | *Mimulus tenellus* Bunge 애기물꽈리아재비 |  | Ⅳ | OBW | 0 | 0 | 1 | 1 | 0 | 0 |
| Scrophulariaceae | *Mimulus tenellus* Bunge var. *nepalensis* (Benth.) P. C. Tsoong 물꽈리아재비 |  |  | OBW | 2 | 1 | 2 | 4 | 0 | 0 |
| Scrophulariaceae | *Paulownia coreana* Uyeki 오동나무 |  |  | OBU | 1 | 2 | 2 | 1 | 2 | 0 |
| Scrophulariaceae | *Paulownia tomentosa* (Thunb.) Steud. 참오동나무 |  |  | OBU | 2 | 1 | 0 | 1 | 1 | 0 |
| Scrophulariaceae | *Pedicularis resupinata* L. 송이풀 | ○ |  | OBU | 3 | 0 | 2 | 0 | 1 | 0 |
| Scrophulariaceae | *Pedicularis resupinata* L. var. *umbrosa* (Kom.) Nakai 그늘송이풀 |  |  | OBU | 1 | 0 | 0 | 0 | 0 | 0 |
| Scrophulariaceae | *Phtheirospermum japonicum* (Thunb.) Kanitz 나도송이풀 | ○ |  | FAC | 3 | 0 | 1 | 0 | 0 | 0 |
| Scrophulariaceae | *Pseudolysimachion rotundum* (Nakai) Holub var. *subintegrum* (Nakai) T. Yamaz. 산꼬리풀 |  |  | OBU | 2 | 0 | 0 | 0 | 0 | 0 |
| Scrophulariaceae | *Scrophularia kakudensis* Franch. 큰개현삼 |  | Ⅱ | OBW | 2 | 0 | 0 | 0 | 0 | 0 |
| Scrophulariaceae | *Scrophularia koraiensis* Nakai 토현삼 |  | Ⅳ | OBW | 6 | 1 | 1 | 0 | 2 | 0 |
| Scrophulariaceae | *Siphonostegia chinensis* Benth. 절국대 |  |  | OBU | 0 | 0 | 1 | 0 | 0 | 0 |
| Scrophulariaceae | *Veronica arvensis* L. 선개불알풀 |  |  | OBU | 2 | 0 | 0 | 1 | 0 | 0 |
| Acanthaceae | *Justicia procumbens* L. 쥐꼬리망초 |  |  | OBU | 1 | 0 | 5 | 2 | 5 | 2 |
| Phrymaceae | *Phryma leptostachya* L. var. *oblongifolia* (Koidz.) Honda 파리풀 |  |  | OBU | 38 | 20 | 33 | 19 | 31 | 0 |
| Plantaginaceae | *Plantago asiatica* L. 질경이 |  |  | OBU | 21 | 5 | 30 | 8 | 12 | 0 |
| Plantaginaceae | *Plantago depressa* Willd. 털질경이 | ○ |  | OBU | 1 | 0 | 1 | 0 | 0 | 0 |
| Plantaginaceae | *Plantago major* L. var. *japonica* (Franch. & Sav.) Kuntze 왕질경이 |  |  | FACW | 1 | 0 | 1 | 0 | 1 | 0 |
| Caprifoliaceae | *Lonicera caerulea* L. 댕댕이나무 | ○ | Ⅳ | OBU | 0 | 0 | 0 | 0 | 1 | 0 |
| Caprifoliaceae | *Lonicera harae* Makino 길마가지나무 |  |  | OBU | 0 | 0 | 0 | 1 | 0 | 0 |
| Caprifoliaceae | *Lonicera japonica* Thunb. 인동덩굴 |  |  | OBU | 43 | 11 | 49 | 17 | 27 | 2 |
| Caprifoliaceae | *Lonicera maackii* (Rupr.) Maxim. 괴불나무 | ○ | Ⅰ | OBU | 6 | 0 | 0 | 2 | 2 | 0 |
| Caprifoliaceae | *Lonicera nigra* L. var. *barbinervis* (Kom.) Nakai 암괴불나무 |  |  | FAC | 1 | 0 | 0 | 0 | 0 | 0 |
| Caprifoliaceae | *Lonicera praeflorens* Batalin 올괴불나무 | ○ |  | OBW | 13 | 6 | 3 | 3 | 1 | 0 |
| Caprifoliaceae | *Lonicera subhispida* Nakai 털괴불나무 |  |  | OBW | 0 | 0 | 3 | 0 | 6 | 0 |
| Caprifoliaceae | *Lonicera subsessilis* Rehder 청괴불나무 |  | Ⅱ | OBU | 3 | 0 | 0 | 0 | 1 | 0 |
| Caprifoliaceae | *Sambucus racemosa* L. subsp. *kamtschatica* (E. Wolf) Hultén 지렁쿠나무 |  |  | OBU | 3 | 0 | 0 | 4 | 1 | 0 |
| Caprifoliaceae | *Sambucus williamsii* Hance 딱총나무 | ○ |  | OBW | 22 | 7 | 12 | 6 | 6 | 0 |
| Caprifoliaceae | *Viburnum carlesii* Hemsl. 분꽃나무 |  |  | OBU | 0 | 0 | 3 | 0 | 0 | 0 |
| Caprifoliaceae | *Viburnum dilatatum* Thunb. 가막살나무 |  |  | OBU | 2 | 1 | 3 | 1 | 10 | 3 |
| Caprifoliaceae | *Viburnum erosum* Thunb. 덜꿩나무 |  |  | OBU | 1 | 5 | 2 | 1 | 11 | 1 |
| Caprifoliaceae | *Viburnum furcatum* Blume ex Maxim. 분단나무 |  | Ⅲ | OBU | 0 | 0 | 0 | 0 | 0 | 1 |
| Caprifoliaceae | *Viburnum opulus* L. var. *calvescens* (Rehder) H. Hara 백당나무 |  | Ⅰ | OBU | 22 | 5 | 5 | 5 | 3 | 0 |
| Caprifoliaceae | *Viburnum wrightii* Miq. 산가막살나무 |  |  | OBU | 2 | 0 | 0 | 0 | 3 | 0 |
| Caprifoliaceae | *Weigela florida* (Bunge) A. DC. 붉은병꽃나무 |  | Ⅱ | OBU | 14 | 0 | 4 | 0 | 0 | 0 |
| Caprifoliaceae | *Weigela praecox* (Lemoine) L. H. Bailey 소영도리나무 |  | Ⅱ | OBU | 8 | 0 | 0 | 1 | 0 | 0 |
| Caprifoliaceae | *Weigela subsessilis* (Nakai) L. H. Bailey 병꽃나무 |  |  | OBW | 32 | 14 | 18 | 20 | 27 | 0 |
| Valerianaceae | *Patrinia scabiosifolia* Fisch. ex Trevir. 마타리 |  |  | FAC | 3 | 1 | 4 | 5 | 3 | 0 |
| Valerianaceae | *Patrinia saniculifolia* Hemsl. 금마타리 |  | Ⅲ | FACW | 1 | 0 | 0 | 0 | 0 | 0 |
| Valerianaceae | *Patrinia villosa* (Thunb.) Juss. 뚝갈 |  |  | OBW | 3 | 0 | 1 | 0 | 1 | 0 |
| Valerianaceae | *Valeriana fauriei* Briq. 쥐오줌풀 |  |  | OBU | 14 | 0 | 3 | 0 | 2 | 0 |
| Campanulaceae | *Adenophora remotiflora* (Siebold & Zucc.) Miq. 모시대 |  |  | FACW | 1 | 1 | 0 | 0 | 0 | 1 |
| Campanulaceae | *Adenophora triphylla* (Thunb.) A. DC. 층층잔대 |  |  | FACU | 2 | 0 | 0 | 0 | 0 | 1 |
| Campanulaceae | *Adenophora triphylla* (Thunb.) A. DC. var. *japonica* (Regel) H. Hara 잔대 |  |  | FAC | 8 | 4 | 6 | 0 | 4 | 0 |
| Campanulaceae | *Asyneuma japonicum* (Miq.) Briq. 영아자 | ○ |  | OBU | 3 | 2 | 0 | 2 | 2 | 0 |
| Campanulaceae | *Campanula punctata* Lam. 초롱꽃 | ○ | Ⅰ | FACU | 9 | 2 | 1 | 0 | 0 | 0 |
| Campanulaceae | *Codonopsis lanceolata* (Siebold & Zucc.) Benth. & Hook. f. ex Trautv. 더덕 |  |  | FACU | 8 | 3 | 1 | 3 | 2 | 1 |
| Campanulaceae | *Hanabusaya asiatica* (Nakai) Nakai 금강초롱꽃 |  | Ⅳ | FAC | 1 | 0 | 0 | 0 | 0 | 0 |
| Ranunculaceae | *Actaea bifida* (Nakai) J. Compton 세잎승마 |  | Ⅳ | FACU | 2 | 1 | 1 | 0 | 0 | 0 |
| Campanulaceae | *Lobelia chinensis* Lour. 수염가래꽃 |  |  | OBW | 0 | 0 | 0 | 1 | 1 | 4 |
| Campanulaceae | *Lobelia sessilifolia* Lamb. 숫잔대 | ○ | Ⅱ | OBU | 0 | 0 | 4 | 0 | 8 | 0 |
| Campanulaceae | *Platycodon grandiflorus* (Jacq.) A. DC. 도라지 |  |  | OBU | 0 | 0 | 0 | 1 | 3 | 0 |
| Asteraceae | *Achillea alpina* L. 톱풀 |  | Ⅱ | OBW | 3 | 0 | 1 | 0 | 1 | 0 |
| Asteraceae | *Achillea alpina* L. var. *discoidea* (Regel) Kitam. 산톱풀 | ○ |  | OBU | 1 | 0 | 0 | 0 | 0 | 0 |
| Asteraceae | *Achillea millefolium* L. 서양톱풀 |  |  | OBU | 0 | 0 | 0 | 0 | 1 | 0 |
| Asteraceae | *Adenocaulon himalaicum* Edgew. 멸가치 |  |  | FACU | 6 | 4 | 2 | 0 | 2 | 0 |
| Asteraceae | *Ageratina altissima* (L.) R. M. King & H. Rob. 서양등골나물 |  |  | OBU | 1 | 4 | 0 | 0 | 0 | 0 |
| Asteraceae | *Ainsliaea acerifolia* Sch. Bip. 단풍취 |  |  | OBU | 15 | 1 | 1 | 1 | 2 | 0 |
| Asteraceae | *Ainsliaea apiculata* Sch. Bip. 좀딱취 |  | Ⅰ | OBU | 0 | 0 | 0 | 0 | 0 | 1 |
| Asteraceae | *Ambrosia artemisiifolia* L. 돼지풀 |  |  | OBU | 3 | 6 | 9 | 1 | 5 | 1 |
| Asteraceae | *Ambrosia trifida* L. 단풍잎돼지풀 |  |  | OBU | 2 | 0 | 1 | 0 | 0 | 0 |
| Asteraceae | *Artemisia angustissima* Nakai 실제비쑥 |  |  | FAC | 0 | 0 | 0 | 0 | 1 | 0 |
| Asteraceae | *Artemisia capillaris* Thunb. 사철쑥 |  |  | OBU | 0 | 0 | 2 | 0 | 0 | 0 |
| Asteraceae | *Artemisia codonocephala* Diels 참쑥 |  |  | OBU | 0 | 0 | 2 | 2 | 2 | 0 |
| Asteraceae | *Artemisia indica* Willd. 쑥 |  |  | OBU | 63 | 25 | 73 | 41 | 45 | 2 |
| Asteraceae | *Artemisia japonica* Thunb. 제비쑥 |  |  | OBU | 8 | 0 | 8 | 6 | 1 | 0 |
| Asteraceae | *Artemisia keiskeana* Miq. 맑은대쑥 |  |  | FACU | 8 | 5 | 8 | 4 | 4 | 0 |
| Asteraceae | *Artemisia koidzumii* Nakai 율무쑥 |  |  | OBU | 0 | 0 | 1 | 0 | 0 | 0 |
| Asteraceae | *Artemisia lancea* Vaniot 뺑쑥 | ○ |  | FACW | 4 | 4 | 5 | 3 | 2 | 0 |
| Asteraceae | *Artemisia montana* (Nakai) Pamp. 산쑥 |  |  | OBU | 5 | 3 | 7 | 0 | 9 | 0 |
| Asteraceae | *Artemisia rubripes* Nakai 덤불쑥 | ○ | Ⅰ | OBU | 0 | 0 | 3 | 4 | 0 | 0 |
| Asteraceae | *Artemisia sacrorum* Ledeb. 털산쑥 |  |  | OBU | 1 | 0 | 0 | 0 | 0 | 0 |
| Asteraceae | *Artemisia sacrorum* Ledeb. var. *iwayomogi* (Kitam.) M. S. Park & G. Y. Chung 더위지기 |  |  | OBU | 2 | 0 | 6 | 0 | 0 | 0 |
| Asteraceae | *Artemisia selengensis* Turcz. ex Besser 물쑥 |  |  | FAC | 8 | 0 | 13 | 11 | 9 | 0 |
| Asteraceae | *Artemisia stolonifera* (Maxim.) Kom. 넓은잎외잎쑥 |  |  | OBW | 43 | 7 | 6 | 2 | 5 | 0 |
| Asteraceae | *Artemisia sylvatica* Maxim. 그늘쑥 | ○ |  | OBU | 10 | 2 | 0 | 0 | 1 | 0 |
| Asteraceae | *Aster ageratoides* Turcz. 까실쑥부쟁이 |  |  | OBU | 8 | 4 | 6 | 4 | 4 | 0 |
| Asteraceae | *Aster fastigiatus* Fisch. 옹굿나물 |  |  | OBU | 0 | 0 | 2 | 0 | 0 | 0 |
| Asteraceae | *Aster incisus* Fisch. 가새쑥부쟁이 |  |  | FACW | 8 | 3 | 1 | 8 | 6 | 0 |
| Asteraceae | *Aster koraiensis* Nakai 벌개미취 |  |  | FAC | 0 | 1 | 3 | 2 | 10 | 0 |
| Asteraceae | *Aster maackii* Regel 좀개미취 | ○ | Ⅲ | OBW | 0 | 0 | 0 | 0 | 1 | 0 |
| Asteraceae | *Aster meyendorffii* (Regel & Maack) Voss 개쑥부쟁이 |  |  | OBW | 1 | 3 | 1 | 2 | 2 | 0 |
| Asteraceae | *Aster scaber* Thunb. 참취 |  |  | OBU | 40 | 7 | 27 | 15 | 33 | 0 |
| Asteraceae | *Aster tataricus* L.f. 개미취 |  |  | FACU | 24 | 6 | 0 | 1 | 1 | 0 |
| Asteraceae | *Aster yomena* (Kitam.) Honda 쑥부쟁이 |  |  | OBU | 1 | 0 | 6 | 4 | 8 | 0 |
| Asteraceae | *Atractylodes* ovata (Thunb.) DC. 삽주 |  |  | OBW | 3 | 1 | 0 | 1 | 3 | 0 |
| Asteraceae | *Bidens bipinnata* L. 도깨비바늘 |  |  | OBU | 5 | 0 | 2 | 0 | 2 | 2 |
| Asteraceae | *Bidens frondosa* L. 미국가막사리 |  |  | FACU | 48 | 18 | 48 | 15 | 24 | 0 |
| Asteraceae | *Bidens parviflora* Willd. 까치발 |  |  | OBU | 0 | 0 | 0 | 0 | 0 | 1 |
| Asteraceae | *Bidens pilosa* L. 울산도깨비바늘 |  |  | FAC | 0 | 0 | 2 | 0 | 0 | 0 |
| Asteraceae | *Bidens tripartita* L. 가막사리 |  |  | FACW | 8 | 4 | 16 | 5 | 17 | 6 |
| Asteraceae | *Breea segeta* (Bunge) Kitam. 조뱅이 |  |  | FACU | 1 | 0 | 1 | 0 | 0 | 0 |
| Asteraceae | *Carduus crispus* L. 지느러미엉겅퀴 |  |  | OBU | 6 | 1 | 0 | 1 | 0 | 0 |
| Asteraceae | *Carpesium abrotanoides* L. 담배풀 |  |  | OBU | 8 | 3 | 9 | 4 | 7 | 0 |
| Asteraceae | *Carpesium cernuum* L. 좀담배풀 |  |  | OBU | 0 | 0 | 0 | 0 | 1 | 0 |
| Asteraceae | *Carpesium divaricatum* Siebold & Zucc. 긴담배풀 |  |  | FACW | 1 | 0 | 9 | 0 | 4 | 0 |
| Asteraceae | *Carpesium macrocephalum* Franch. & Sav. 여우오줌 | ○ | Ⅰ | OBU | 10 | 2 | 1 | 1 | 2 | 0 |
| Asteraceae | *Carpesium triste* Maxim. 두메담배풀 |  |  | FACU | 5 | 0 | 0 | 0 | 0 | 0 |
| Asteraceae | *Centipeda minima* (L.) A. Braun & Asch. 중대가리풀 |  |  | OBU | 1 | 1 | 2 | 2 | 0 | 0 |
| Asteraceae | *Chrysanthemum boreale* (Makino) Makino 산국 |  |  | OBU | 3 | 1 | 12 | 3 | 2 | 0 |
| Asteraceae | *Chrysanthemum indicum* L. 감국 |  |  | OBU | 0 | 0 | 0 | 0 | 1 | 0 |
| Asteraceae | *Cirsium japonicum* Fisch. ex DC. var. *maackii* (Maxim.) Matsum. 엉겅퀴 |  |  | OBW | 11 | 5 | 13 | 5 | 13 | 0 |
| Asteraceae | *Cirsium japonicum* Fisch. ex DC. var. *spinossimum* Kitam. 가시엉겅퀴 |  |  | OBU | 0 | 0 | 0 | 0 | 0 | 1 |
| Asteraceae | *Cirsium pendulum* Fisch. ex DC. 큰엉겅퀴 | ○ | Ⅰ | FACU | 5 | 0 | 1 | 1 | 1 | 0 |
| Asteraceae | *Cirsium rhinoceros* (H. Lév. & Vaniot) Nakai 바늘엉겅퀴 |  | Ⅲ | FAC | 0 | 0 | 0 | 0 | 0 | 4 |
| Asteraceae | *Cirsium schantarense* Trautv. & C. A. Mey.도깨비엉겅퀴 | ○ | Ⅲ | OBU | 2 | 0 | 0 | 0 | 0 | 0 |
| Asteraceae | *Cirsium setidens* (Dunn) Nakai 고려엉겅퀴 |  | Ⅰ | FAC | 5 | 0 | 0 | 1 | 1 | 0 |
| Asteraceae | *Conyza bonariensis* (L.) Cronquist 실망초 |  |  | OBU | 0 | 0 | 0 | 0 | 1 | 0 |
| Asteraceae | *Conyza canadensis* (L.) Cronquist 망초 |  |  | FACU | 11 | 9 | 10 | 12 | 12 | 0 |
| Asteraceae | *Coreopsis lanceolata* L. 큰금계국 |  |  | OBU | 0 | 0 | 1 | 0 | 2 | 0 |
| Asteraceae | *Cosmos bipinnatus* Cav. 코스모스 |  |  | FACU | 0 | 0 | 1 | 0 | 0 | 0 |
| Asteraceae | *Crassocephalum crepidioides* (Benth.) S. Moore 주홍서나물 |  |  | OBW | 1 | 0 | 1 | 0 | 3 | 0 |
| Asteraceae | *Crepidiastrum chelidoniifolium* (Makino) J. H. Pak & Kawano 까치고들빼기 |  |  | OBU | 1 | 0 | 0 | 0 | 0 | 0 |
| Asteraceae | *Crepidiastrum denticulatum* (Houtt.) J. H. Pak & Kawano 이고들빼기 |  |  | OBU | 6 | 3 | 3 | 6 | 4 | 0 |
| Asteraceae | *Crepidiastrum sonchifolium* (Maxim.) J. H. Pak & Kawano 고들빼기 |  |  | FACU | 2 | 0 | 0 | 1 | 2 | 1 |
| Asteraceae | *Dendranthema oreastrum* (Hance) Y. Ling 바위구절초 |  |  | OBU | 1 | 0 | 0 | 0 | 0 | 0 |
| Asteraceae | *Dendranthema zawadskii* (Herbich) Tzvelev 산구절초 | ○ |  | FACU | 0 | 1 | 0 | 1 | 1 | 0 |
| Asteraceae | *Dendranthema zawadskii* (Herbich) Tzvelev var. *leiophyllum* (Nakai) M. Kim 구절초 |  |  | OBU | 1 | 0 | 6 | 2 | 3 | 0 |
| Asteraceae | *Eclipta prostrata* (L.) L. 한련초 |  |  | OBU | 0 | 0 | 0 | 0 | 1 | 0 |
| Asteraceae | *Erechtites hieraciifolius* (L.) Raf. ex DC. 붉은서나물 |  |  | FAC | 3 | 9 | 9 | 5 | 7 | 0 |
| Asteraceae | *Erigeron annuus* (L.) Pers. 개망초 |  |  | OBU | 63 | 17 | 65 | 26 | 30 | 1 |
| Asteraceae | *Erigeron floribundus* (Kunth) Sch. Bip. 큰망초 |  |  | FACU | 1 | 0 | 0 | 0 | 0 | 0 |
| Asteraceae | *Erigeron philadelphicus* L. 봄망초 |  |  | FACU | 2 | 0 | 0 | 0 | 0 | 0 |
| Asteraceae | *Erigeron strigosus* Muhl. ex Willd. 주걱개망초 |  |  | FAC | 6 | 0 | 1 | 2 | 0 | 0 |
| Asteraceae | *Euchiton japonicus* (Thunb.) Holub 풀솜나물 |  |  | OBU | 0 | 0 | 0 | 0 | 1 | 1 |
| Asteraceae | *Eupatorium japonicum* Thunb. 등골나물 |  |  | OBU | 32 | 9 | 27 | 14 | 23 | 0 |
| Asteraceae | *Eupatorium lindleyanum* DC. 골등골나물 |  |  | FACW | 10 | 1 | 20 | 1 | 13 | 0 |
| Asteraceae | *Eupatorium makinoi* Kawah. & Yahara var. *oppositifolium* (Koidz.) Kawah. & Yahara 벌등골나물 |  |  | FAC | 11 | 2 | 2 | 2 | 9 | 0 |
| Asteraceae | *Eupatorium tripartitum* (Makino) Murata & H. Koyama 향등골나물 |  |  | FACU | 0 | 0 | 0 | 0 | 1 | 0 |
| Asteraceae | *Galinsoga quadriradiata* Ruiz & Pav. 털별꽃아재비 |  |  | OBU | 3 | 0 | 0 | 0 | 0 | 0 |
| Asteraceae | *Helianthus tuberosus* L. 뚱딴지 |  |  | FAC | 1 | 0 | 1 | 2 | 2 | 0 |
| Asteraceae | *Hemistepta lyrata* (Bunge) Bunge 지칭개 |  |  | OBU | 2 | 0 | 1 | 1 | 0 | 0 |
| Asteraceae | *Hieracium umbellatum* L. 조밥나물 |  |  | FAC | 1 | 0 | 3 | 1 | 0 | 0 |
| Asteraceae | *Hololeion maximowiczii* Kitam. 께묵 |  |  | FACU | 0 | 0 | 3 | 0 | 3 | 0 |
| Asteraceae | *Inula japonica* Thunb. 금불초 |  |  | OBW | 1 | 0 | 2 | 0 | 0 | 0 |
| Asteraceae | *Inula linariifolia* Turcz. 가는금불초 |  |  | FAC | 2 | 0 | 3 | 0 | 0 | 0 |
| Asteraceae | *Inula salicina* L. 버들금불초 | ○ |  | OBU | 0 | 0 | 2 | 0 | 0 | 0 |
| Asteraceae | *Ixeridium dentatum* (Thunb.) Tzvelev 씀바귀 |  |  | FACU | 2 | 1 | 2 | 2 | 7 | 2 |
| Asteraceae | *Ixeridium dentatum* (Thunb.) Tzvelev f. *albiflora* (Makino) H. Hara 흰씀바귀 |  |  | FACU | 0 | 0 | 0 | 0 | 1 | 0 |
| Asteraceae | *Ixeris chinensis* (Thunb.) Nakai 노랑선씀바귀 |  |  | OBU | 0 | 0 | 0 | 0 | 1 | 0 |
| Asteraceae | *Ixeris debilis* (Thunb.) A. Gray 벋음씀바귀 |  |  | FAC | 0 | 0 | 0 | 1 | 0 | 0 |
| Asteraceae | *Ixeris polycephala* Cass. 벌씀바귀 |  |  | FAC | 4 | 0 | 0 | 1 | 0 | 0 |
| Asteraceae | *Ixeris stolonifera* A. Gray 좀씀바귀 |  |  | OBW | 0 | 0 | 0 | 0 | 0 | 2 |
| Asteraceae | *Ixeris strigosa* (H. Lév. & Vaniot) J. H. Pak & Kawano 선씀바귀 |  |  | FAC | 1 | 0 | 0 | 0 | 0 | 0 |
| Asteraceae | *Lactuca indica* L. 왕고들빼기 |  |  | OBU | 5 | 1 | 14 | 5 | 10 | 0 |
| Asteraceae | *Lactuca indica* L. var. *laciniata* (Houtt.) H. Hara f. *indivisa* (Maxim.) H. Hara 가는잎왕고들빼기 |  |  | OBU | 0 | 0 | 0 | 0 | 1 | 0 |
| Asteraceae | *Lactuca raddeana* Maxim. 산씀바귀 |  |  | FAC | 4 | 0 | 2 | 1 | 4 | 0 |
| Asteraceae | *Lactuca triangulata* Maxim. 두메고들빼기 |  |  | OBU | 2 | 0 | 0 | 0 | 0 | 0 |
| Asteraceae | *Lapsanastrum apogonoides* (Maxim.) J. H. Pak & K. Bremer 개보리뺑이 |  | Ⅰ | OBU | 0 | 0 | 0 | 0 | 1 | 0 |
| Asteraceae | *Leibnitzia anandria* (L.) Turcz. 솜나물 |  |  | OBU | 0 | 0 | 2 | 0 | 0 | 0 |
| Asteraceae | *Ligularia fischeri* (Ledeb.) Turcz. 곰취 |  | Ⅱ | FAC | 25 | 2 | 2 | 2 | 3 | 0 |
| Asteraceae | *Ligularia intermedia* Nakai 어리곤달비 |  |  | FACU | 1 | 0 | 0 | 0 | 0 | 0 |
| Asteraceae | *Parasenecio auriculatus* (DC.) J. R. Grant 귀박쥐나물 | ○ | Ⅳ | OBU | 1 | 0 | 0 | 0 | 0 | 0 |
| Asteraceae | *Parasenecio auriculatus* (DC.) J. R. Grant var. *kamtschatica* (Maxim.) H. Koyama 나래박쥐나물 |  | Ⅲ | OBU | 3 | 0 | 0 | 0 | 0 | 0 |
| Asteraceae | *Parasenecio auriculatus* (DC.) J. R. Grant var. *matsumurana* (Nakai) M. Kim 박쥐나물 |  |  | FACW | 3 | 1 | 0 | 0 | 1 | 0 |
| Asteraceae | *Parasenecio hastatus* (L.) H. Koyama var. *orientalis* (Kitam.) H. Koyama 민박쥐나물 |  | Ⅲ | OBU | 3 | 0 | 0 | 0 | 0 | 0 |
| Asteraceae | *Petasites japonicus* (Siebold & Zucc.) Maxim. 머위 |  |  | OBU | 12 | 5 | 14 | 8 | 8 | 0 |
| Asteraceae | *Picris hieracioides* L. subsp. *japonica* (Thunb.) Hand.-Mazz. 쇠서나물 |  |  | FACW | 1 | 0 | 3 | 1 | 3 | 0 |
| Asteraceae | *Prenanthes ochroleuca* (Maxim.) Hemsl. 왕씀배 |  |  | OBU | 0 | 0 | 0 | 0 | 1 | 0 |
| Asteraceae | *Pseudognaphalium* affine (D. Don) Anderb. 떡쑥 |  |  | OBW | 1 | 0 | 2 | 1 | 0 | 0 |
| Asteraceae | *Rudbeckia bicolor* Nutt. 원추천인국 |  |  | OBU | 0 | 0 | 1 | 0 | 0 | 0 |
| Asteraceae | *Rudbeckia laciniata* L. 삼잎국화 |  |  | OBU | 1 | 0 | 0 | 0 | 0 | 0 |
| Asteraceae | *Saussurea gracilis* Maxim. 은분취 |  |  | OBU | 1 | 0 | 0 | 0 | 0 | 0 |
| Asteraceae | *Saussurea grandicapitula* W. T. Lee & H. T. Im 태백취 |  | Ⅳ | FACU | 1 | 0 | 0 | 0 | 0 | 0 |
| Asteraceae | *Saussurea grandifolia* Maxim. 서덜취 | ○ |  | OBU | 4 | 0 | 0 | 0 | 0 | 0 |
| Asteraceae | *Saussurea macrolepis* (Nakai) Kitam. 각시서덜취 |  |  | OBU | 4 | 0 | 0 | 0 | 0 | 0 |
| Asteraceae | *Saussurea maximowiczii* Herder 버들분취 | ○ |  | OBU | 2 | 1 | 2 | 1 | 11 | 1 |
| Asteraceae | *Saussurea mongolica* (Franch.) Franch. 북분취 |  |  | OBU | 0 | 0 | 1 | 0 | 0 | 0 |
| Asteraceae | *Saussurea seoulensis* Nakai 분취 |  |  | OBU | 0 | 0 | 0 | 1 | 0 | 0 |
| Asteraceae | *Saussurea tanakae* Franch. & Sav. ex Maxim. 당분취 |  |  | OBU | 1 | 0 | 0 | 0 | 0 | 0 |
| Asteraceae | *Scorzonera albicaulis* Bunge 쇠채 | ○ |  | OBW | 1 | 0 | 0 | 0 | 0 | 0 |
| Asteraceae | *Senecio argunensis* Turcz. 쑥방망이 |  | Ⅰ | FACU | 0 | 0 | 2 | 0 | 0 | 0 |
| Asteraceae | *Serratula coronata* L. subsp. *insularis* (Iljin) Kitam. 산비장이 | ○ |  | OBU | 3 | 0 | 1 | 0 | 5 | 0 |
| Asteraceae | *Sigesbeckia glabrescens* (Makino) Makino 진득찰 |  |  | OBW | 10 | 9 | 13 | 2 | 3 | 0 |
| Asteraceae | *Sigesbeckia orientalis* L. subsp. *pubescens* (Makino) H. Koyama 털진득찰 |  |  | FAC | 1 | 4 | 6 | 3 | 0 | 0 |
| Asteraceae | *Solidago gigantea* Aiton 미국미역취 |  |  | FACU | 0 | 0 | 0 | 1 | 0 | 0 |
| Asteraceae | *Solidago virgaurea* L. subsp. *asiatica* Kitam. ex H. Hara 미역취 |  |  | OBU | 23 | 11 | 9 | 6 | 13 | 1 |
| Asteraceae | *Sonchus brachyotus* DC. 사데풀 | ○ |  | OBU | 0 | 0 | 1 | 0 | 0 | 0 |
| Asteraceae | *Stemmacantha uniflora* (L.) Dittrich 뻐꾹채 |  | Ⅰ | FACU | 0 | 0 | 1 | 0 | 0 | 0 |
| Asteraceae | *Symphyotrichum pilosum* (Willd.) G. L. Nesom 미국쑥부쟁이 |  |  | FACU | 10 | 5 | 14 | 7 | 0 | 0 |
| Asteraceae | *Syneilesis palmata* (Thunb.) Maxim. 우산나물 |  |  | OBU | 11 | 4 | 4 | 1 | 1 | 0 |
| Asteraceae | *Synurus deltoides* (Aiton) Nakai 수리취 | ○ |  | FAC | 2 | 0 | 1 | 0 | 1 | 0 |
| Asteraceae | *Synurus excelsus* (Makino) Kitam. 큰수리취 |  |  | FAC | 4 | 0 | 0 | 0 | 0 | 0 |
| Asteraceae | *Tagetes minuta* L. 만수국아재비 |  |  | OBU | 0 | 0 | 0 | 0 | 1 | 0 |
| Asteraceae | *Taraxacum officinale* F. H. Wigg. 서양민들레 |  |  | OBU | 8 | 0 | 0 | 2 | 0 | 1 |
| Asteraceae | *Taraxacum ohwianum* Kitam. 산민들레 |  |  | OBU | 2 | 0 | 1 | 0 | 1 | 0 |
| Asteraceae | *Tephroseris pseudosonchus* (Vaniot) C. Jeffrey & Y. L. Chen 물솜방망이 |  | Ⅱ | FACW | 5 | 0 | 12 | 0 | 1 | 0 |
| Asteraceae | *Xanthium strumarium* L. 도꼬마리 |  |  | OBU | 0 | 0 | 0 | 0 | 0 | 1 |
| Asteraceae | *Youngia japonica* (L.) DC. 뽀리뱅이 |  |  | FACU | 6 | 0 | 2 | 3 | 3 | 0 |
| Hydrocharitaceae | *Ottelia alismoides* (L.) Pers. 물질경이 |  | Ⅱ | OBU | 1 | 0 | 2 | 0 | 0 | 0 |
| Cabombaceae | *Brasenia schreberi* J. F. Gmel. 순채 |  | Ⅴ | OBW | 1 | 0 | 0 | 0 | 0 | 7 |
| Paeoniaceae | *Paeonia japonica* (Makino) Miyabe & Takeda 백작약 |  | Ⅱ | OBU | 1 | 0 | 0 | 0 | 0 | 0 |
| Droseraceae | *Drosera rotundifolia* L. 끈끈이주걱 | ○ | Ⅲ | OBU | 0 | 0 | 5 | 0 | 9 | 0 |
| Alismataceae | *Alisma canaliculatum* A. Braun & C. D. Bouché 택사 |  | Ⅱ | FACW | 2 | 0 | 1 | 1 | 1 | 5 |
| Alismataceae | *Alisma orientale* (Sam.) Juz. 질경이택사 |  | Ⅱ | OBU | 0 | 0 | 2 | 0 | 0 | 0 |
| Alismataceae | *Caldesia parnassifolia* (Bassi ex L.) Parl. 둥근잎택사 |  | Ⅳ | OBU | 0 | 0 | 0 | 0 | 0 | 2 |
| Alismataceae | *Sagittaria aginashii* Makino 보풀 |  | Ⅰ | OBW | 2 | 0 | 5 | 3 | 0 | 0 |
| Alismataceae | *Sagittaria trifolia* L. 벗풀 |  |  | OBW | 5 | 0 | 5 | 1 | 2 | 0 |
| Juncaginaceae | *Triglochin maritima* L. 지채 |  | Ⅰ | OBU | 0 | 0 | 0 | 0 | 1 | 0 |
| Potamogetonaceae | *Potamogeton cristatus* Regel & Maack 가는가래 |  | Ⅰ | OBW | 0 | 0 | 0 | 0 | 0 | 3 |
| Potamogetonaceae | *Potamogeton distinctus* A. Benn. 가래 |  |  | OBW | 7 | 1 | 6 | 0 | 1 | 6 |
| Potamogetonaceae | *Potamogeton pusillus* L. 실말 |  |  | OBU | 0 | 0 | 0 | 0 | 1 | 0 |
| Najadaceae | *Najas graminea* Delile 나자스말 |  |  | FACU | 0 | 0 | 0 | 1 | 1 | 1 |
| Najadaceae | *Najas minor* All. 톱니나자스말 |  |  | FACU | 0 | 0 | 1 | 1 | 0 | 0 |
| Liliaceae | *Allium macrostemon* Bunge 산달래 |  |  | OBU | 0 | 0 | 0 | 3 | 1 | 1 |
| Liliaceae | *Allium monanthum* Maxim. 달래 | ○ |  | FACU | 0 | 0 | 0 | 0 | 0 | 1 |
| Liliaceae | *Allium taquetii* H. Lév. & Vaniot 한라부추 |  |  | FACW | 0 | 0 | 0 | 0 | 0 | 3 |
| Liliaceae | *Allium thunbergii* G. Don 산부추 | ○ |  | OBU | 4 | 1 | 4 | 2 | 9 | 1 |
| Liliaceae | *Asparagus oligoclonos* Maxim. 방울비짜루 |  | Ⅰ | OBU | 1 | 0 | 0 | 0 | 0 | 0 |
| Liliaceae | *Asparagus schoberioides* Kunth 비짜루 |  |  | FACU | 1 | 2 | 3 | 2 | 4 | 0 |
| Liliaceae | *Barnardia japonica* (Thunb.) Schult. f. 무릇 |  |  | FACU | 1 | 1 | 6 | 1 | 1 | 2 |
| Liliaceae | *Convallaria keiskei* Miq. 은방울꽃 |  |  | OBU | 18 | 3 | 3 | 1 | 0 | 0 |
| Liliaceae | *Disporum smilacinum* A. Gray 애기나리 | ○ |  | FACU | 18 | 3 | 7 | 5 | 18 | 1 |
| Liliaceae | *Disporum uniflorum* Baker 윤판나물 |  |  | OBW | 0 | 4 | 0 | 0 | 1 | 0 |
| Liliaceae | *Disporum viridescens* (Maxim.) Nakai 큰애기나리 | ○ |  | FACU | 8 | 0 | 0 | 1 | 2 | 0 |
| Liliaceae | *Erythronium japonicum* Decne. 얼레지 | ○ |  | FACW | 0 | 0 | 1 | 0 | 1 | 0 |
| Liliaceae | *Heloniopsis koreana* Fuse, N. S. Lee & M. N. Tamura 처녀치마 |  | Ⅱ | OBW | 3 | 0 | 1 | 0 | 0 | 0 |
| Liliaceae | *Hemerocallis dumortieri* C. Morren 각시원추리 |  |  | FACU | 0 | 0 | 1 | 0 | 0 | 0 |
| Liliaceae | *Hemerocallis fulva* (L.) L. 원추리 |  |  | FACW | 1 | 0 | 3 | 0 | 3 | 3 |
| Liliaceae | *Hemerocallis hakuunensis* Nakai 백운산원추리 |  |  | OBU | 3 | 4 | 1 | 3 | 4 | 1 |
| Liliaceae | *Hemerocallis middendorffii* Trautv. & C. A. Mey.큰원추리 | ○ | Ⅳ | OBW | 0 | 1 | 0 | 0 | 1 | 0 |
| Liliaceae | *Hemerocallis minor* Mill. 애기원추리 | ○ |  | FACU | 0 | 1 | 0 | 0 | 0 | 0 |
| Liliaceae | *Hemerocallis thunbergii* Baker 노랑원추리 |  |  | OBU | 0 | 0 | 0 | 0 | 5 | 0 |
| Liliaceae | *Hosta capitata* (Koidz.) Nakai 일월비비추 |  | Ⅰ | FACW | 2 | 1 | 1 | 0 | 4 | 0 |
| Liliaceae | *Hosta clausa* Nakai 주걱비비추 |  | Ⅲ | FAC | 0 | 1 | 0 | 0 | 0 | 0 |
| Liliaceae | *Hosta longipes* (Franch. & Sav.) Matsum. 비비추 |  |  | OBU | 1 | 0 | 1 | 0 | 4 | 0 |
| Liliaceae | *Hosta minor* (Baker) Nakai 좀비비추 |  | Ⅰ | OBU | 0 | 0 | 3 | 0 | 3 | 0 |
| Liliaceae | *Lilium amabile* Palib. 털중나리 |  |  | OBU | 1 | 0 | 0 | 1 | 1 | 0 |
| Liliaceae | *Lilium distichum* Nakai ex Kamib. 말나리 | ○ | Ⅲ | FAC | 5 | 0 | 0 | 0 | 0 | 0 |
| Liliaceae | *Lilium lancifolium* Thunb. 참나리 |  |  | FAC | 2 | 0 | 0 | 0 | 0 | 0 |
| Liliaceae | *Lilium leichtlinii* Hook. f. var. *maximowiczii* (Regel) Baker 중나리 | ○ | Ⅳ | FACU | 0 | 1 | 1 | 0 | 0 | 0 |
| Liliaceae | *Lilium tsingtauense* Gilg 하늘말나리 |  |  | FACW | 6 | 2 | 0 | 0 | 1 | 0 |
| Liliaceae | *Liriope muscari* (Decne.) L. H. Bailey 맥문동 |  |  | FACU | 0 | 1 | 4 | 1 | 10 | 3 |
| Liliaceae | *Liriope spicata* (Thunb.) Lour. 개맥문동 |  |  | FACU | 0 | 0 | 0 | 2 | 11 | 0 |
| Liliaceae | *Maianthemum bifolium* (L.) F. W. Schmidt 두루미꽃 | ○ | Ⅱ | FAC | 3 | 0 | 0 | 0 | 0 | 0 |
| Liliaceae | *Maianthemum japonicum* (A. Gray) LaFrankie 풀솜대 |  |  | OBU | 6 | 1 | 0 | 2 | 1 | 0 |
| Liliaceae | *Ophiopogon japonicus* (Thunb.) Ker Gawl. 소엽맥문동 |  | Ⅰ | OBU | 0 | 0 | 0 | 1 | 1 | 1 |
| Liliaceae | *Paris verticillata* M. Bieb. 삿갓나물 | ○ |  | OBU | 6 | 1 | 3 | 0 | 1 | 0 |
| Liliaceae | *Polygonatum falcatum* A. Gray 진황정 |  | Ⅲ | FACU | 0 | 0 | 0 | 0 | 1 | 0 |
| Liliaceae | *Polygonatum humile* Fisch. ex Maxim. 각시둥굴레 | ○ |  | OBU | 2 | 0 | 0 | 0 | 0 | 0 |
| Liliaceae | *Polygonatum inflatum* Kom. 퉁둥굴레 | ○ |  | FACW | 7 | 1 | 3 | 1 | 1 | 0 |
| Liliaceae | *Polygonatum infundiflorum* Y. S. Kim, B. U. Oh & C. G. Jang 늦둥굴레 |  |  | FAC | 0 | 1 | 3 | 3 | 0 | 0 |
| Liliaceae | *Polygonatum involucratum* (Franch. & Sav.) Maxim. 용둥굴레 | ○ |  | FACW | 7 | 2 | 0 | 3 | 0 | 0 |
| Liliaceae | *Polygonatum lasianthum* Maxim. 죽대 |  |  | OBU | 0 | 0 | 0 | 0 | 1 | 0 |
| Liliaceae | *Polygonatum odoratum* (Mill.) Druce 풍도둥굴레 | ○ |  | FACW | 0 | 0 | 0 | 1 | 0 | 0 |
| Liliaceae | *Polygonatum odoratum* (Mill.) Druce var. *pluriflorum* (Miq.) Ohwi 둥굴레 |  |  | OBU | 27 | 9 | 8 | 7 | 8 | 0 |
| Liliaceae | *Polygonatum thunbergii* C. Morren & Decne. 산둥굴레 |  |  | OBU | 0 | 0 | 1 | 0 | 0 | 0 |
| Liliaceae | *Polygonatum* ⨉ *desoulavyi* Kom. 안면용둥굴레 | ○ |  | OBU | 1 | 0 | 0 | 0 | 0 | 0 |
| Liliaceae | *Smilax china* L. 청미래덩굴 |  |  | OBU | 23 | 5 | 25 | 18 | 46 | 3 |
| Liliaceae | *Smilax nipponica* Miq. 선밀나물 |  |  | OBU | 23 | 14 | 4 | 8 | 9 | 0 |
| Liliaceae | *Smilax riparia* A. DC. 밀나물 |  |  | OBU | 7 | 2 | 5 | 3 | 8 | 1 |
| Liliaceae | *Smilax sieboldii* Miq. 청가시덩굴 |  |  | FAC | 36 | 20 | 33 | 23 | 11 | 1 |
| Liliaceae | *Smilax sieboldii* Miq. f. *inermis* (Nakai ex T.Mori)  H. Hara 민청가시덩굴 |  |  | FACW | 0 | 1 | 0 | 1 | 1 | 0 |
| Liliaceae | *Streptopus ovalis* (Ohwi) F. T. Wang & Y. C. Tang  금강애기나리 |  |  | FACW | 3 | 0 | 0 | 0 | 0 | 0 |
| Liliaceae | *Tricyrtis macropod*a Miq. 뻐꾹나리 |  | Ⅰ | OBU | 0 | 0 | 0 | 2 | 11 | 0 |
| Liliaceae | *Trillium camschatcense* Ker Gawl. 연영초 | ○ | Ⅳ | FACU | 4 | 0 | 0 | 0 | 0 | 0 |
| Liliaceae | *Trillium tschonoskii* Maxim. 큰연영초 |  | Ⅳ | FAC | 1 | 0 | 0 | 0 | 0 | 0 |
| Liliaceae | *Tulipa edulis* (Miq.) Baker 산자고 |  |  | FAC | 0 | 0 | 0 | 0 | 0 | 1 |
| Liliaceae | *Veratrum dolichopetalum* O. Loes. 푸른박새 |  |  | FACW | 2 | 0 | 0 | 0 | 0 | 0 |
| Liliaceae | *Veratrum maackii* Regel 긴잎여로 | ○ | Ⅲ | OBU | 7 | 0 | 0 | 1 | 0 | 0 |
| Liliaceae | *Veratrum maackii* Regel var. *japonicum* (Baker) Shimizu 여로 |  | Ⅲ | OBU | 3 | 0 | 6 | 2 | 2 | 0 |
| Liliaceae | *Veratrum maackii* Regel var. *parviflorum* (Maxim. ex Miq.) H. Hara 파란여로 |  | Ⅳ | OBU | 0 | 1 | 0 | 0 | 0 | 0 |
| Liliaceae | *Veratrum nigrum* L. var. *ussuriense* O. Loes. 참여로 |  | Ⅲ | OBU | 1 | 0 | 0 | 0 | 0 | 0 |
| Liliaceae | *Veratrum oxysepalum* Turcz. 박새 | ○ | Ⅰ | OBU | 22 | 0 | 2 | 0 | 0 | 1 |
| Liliaceae | *Veratrum versicolor* Nakai 흰여로 |  |  | OBU | 0 | 0 | 0 | 0 | 1 | 0 |
| Lentibulariaceae | *Utricularia aurea* Lour. 들통발 |  | Ⅴ | OBU | 0 | 0 | 0 | 0 | 1 | 0 |
| Lentibulariaceae | *Utricularia bifida* L. 땅귀개 |  | Ⅳ | OBU | 0 | 1 | 3 | 0 | 9 | 0 |
| Lentibulariaceae | *Utricularia caerulea* L. 이삭귀개 |  | Ⅳ | OBU | 0 | 1 | 5 | 0 | 10 | 0 |
| Lentibulariaceae | *Utricularia japonica* Makino 통발 |  | Ⅴ | OBU | 0 | 0 | 0 | 1 | 0 | 4 |
| Lentibulariaceae | *Utricularia uliginosa* Vahl 자주땅귀개 |  | Ⅴ | OBU | 0 | 0 | 2 | 0 | 2 | 2 |
| Dioscoreaceae | *Dioscorea bulbifera* L. 둥근마 |  |  | FACU | 1 | 0 | 0 | 0 | 1 | 0 |
| Dioscoreaceae | *Dioscorea japonica* Thunb. 참마 |  |  | OBU | 16 | 1 | 4 | 3 | 3 | 0 |
| Dioscoreaceae | *Dioscorea nipponica* Makino 부채마 |  |  | OBU | 20 | 3 | 1 | 3 | 2 | 0 |
| Dioscoreaceae | *Dioscorea polystachya* Turcz. 마 |  |  | OBU | 30 | 18 | 31 | 18 | 28 | 0 |
| Dioscoreaceae | *Dioscorea quinquelobata* Thunb. 단풍마 |  |  | FACW | 9 | 0 | 4 | 2 | 4 | 0 |
| Dioscoreaceae | *Dioscorea septemloba* Thunb. 국화마 |  |  | OBU | 4 | 0 | 1 | 0 | 0 | 0 |
| Dioscoreaceae | *Dioscorea tenuipes* Franch. & Sav. 각시마 |  |  | OBU | 0 | 0 | 0 | 1 | 5 | 0 |
| Dioscoreaceae | *Dioscorea tokoro* Makino ex Miyabe 도꼬로마 |  |  | OBU | 1 | 1 | 1 | 0 | 3 | 0 |
| Pontederiaceae | *Monochoria korsakowii* Regel & Maack 물옥잠 | ○ | Ⅱ | OBU | 2 | 0 | 1 | 1 | 0 | 0 |
| Pontederiaceae | *Monochoria vaginalis* (Burm.f.) C. Presl var. *plantaginea* (Roxb.) Solms 물달개비 |  |  | FACW | 4 | 0 | 2 | 0 | 1 | 1 |
| Iridaceae | *Iris ensata* Thunb. var. *spontanea* (Makino) Nakai 꽃창포 | ○ | Ⅱ | FAC | 27 | 2 | 17 | 4 | 17 | 0 |
| Iridaceae | *Iris minutoaurea* Makino 금붓꽃 |  | Ⅰ | FACU | 0 | 1 | 0 | 1 | 0 | 0 |
| Iridaceae | *Iris rossii* Baker 각시붓꽃 |  |  | OBU | 2 | 4 | 2 | 1 | 3 | 0 |
| Iridaceae | *Iris rossii* Baker var. *latifolia* J. K. Sim & Y. S. Kim 넓은잎각시붓꽃 |  |  | FACW | 0 | 0 | 0 | 1 | 1 | 0 |
| Iridaceae | *Iris pseudacorus* L. 노랑꽃창포 |  |  | FACW | 0 | 0 | 0 | 1 | 0 | 0 |
| Iridaceae | *Iris sanguinea* Donn ex Hornem. 붓꽃 | ○ |  | OBW | 5 | 4 | 0 | 1 | 0 | 0 |
| Iridaceae | *Sisyrinchium rosulatum* E. P. Bicknell 등심붓꽃 |  |  | OBW | 0 | 0 | 0 | 0 | 0 | 1 |
| Juncaceae | *Juncus alatus* Franch. & Sav. 날개골풀 |  |  | OBW | 0 | 0 | 0 | 0 | 2 | 0 |
| Juncaceae | *Juncus bufonius* L. 애기골풀 |  |  | OBU | 0 | 1 | 0 | 0 | 0 | 0 |
| Juncaceae | *Juncus decipiens* (Buchenau) Nakai 골풀 |  |  | OBW | 58 | 18 | 72 | 20 | 48 | 10 |
| Juncaceae | *Juncus diastrophanthus* Buchenau 별날개골풀 |  |  | OBW | 2 | 1 | 0 | 1 | 2 | 3 |
| Juncaceae | *Juncus filiformis* L. 참골풀 | ○ |  | OBU | 0 | 0 | 0 | 0 | 1 | 0 |
| Juncaceae | *Juncus krameri* Franch. & Sav. 비녀골풀 | ○ |  | OBU | 2 | 0 | 1 | 1 | 1 | 1 |
| Juncaceae | *Juncus papillosus* Franch. & Sav. 청비녀골풀 | ○ |  | OBU | 8 | 3 | 9 | 2 | 14 | 0 |
| Juncaceae | *Juncus prismatocarpus* R.Br. subsp. *leschenaultii* (Gay ex Laharpe) Kirschner 참비녀골풀 |  |  | OBW | 1 | 0 | 1 | 0 | 0 | 0 |
| Juncaceae | *Juncus tenuis* Willd. 길골풀 |  |  | FACU | 0 | 1 | 3 | 0 | 0 | 0 |
| Juncaceae | *Juncus wallichianus* J. Gay ex Laharpe 눈비녀골풀 |  |  | OBU | 3 | 2 | 1 | 0 | 6 | 3 |
| Juncaceae | *Luzula capitata* (Miq. ex Franch. & Sav.) Kom. 꿩의밥 |  |  | OBU | 1 | 0 | 3 | 1 | 0 | 2 |
| Juncaceae | *Luzula multiflora* (Ehrh.) Lej. 산꿩의밥 |  | Ⅱ | OBU | 0 | 0 | 0 | 1 | 0 | 1 |
| Commelinaceae | *Aneilema keisak* Hassk. 사마귀풀 |  |  | FACW | 35 | 10 | 33 | 13 | 27 | 6 |
| Commelinaceae | *Commelina communis* L. 닭의장풀 |  |  | OBU | 42 | 16 | 49 | 28 | 25 | 1 |
| Commelinaceae | *Commelina communis* L. . var. *angustifolia* Nakai 좀닭의장풀 |  |  | OBU | 1 | 0 | 1 | 0 | 0 | 0 |
| Commelinaceae | *Streptolirion volubile* Edgew. 덩굴닭의장풀 |  |  | OBU | 0 | 1 | 0 | 1 | 0 | 0 |
| Eriocaulaceae | *Eriocaulon cinereum* R.Br. 곡정초 |  |  | OBW | 0 | 0 | 7 | 0 | 1 | 0 |
| Eriocaulaceae | *Eriocaulon decemflorum* Maxim. 좀개수염 |  |  | OBW | 0 | 0 | 0 | 0 | 0 | 2 |
| Eriocaulaceae | *Eriocaulon miquelianum* Körn. 개수염 |  |  | OBW | 3 | 2 | 2 | 0 | 9 | 1 |
| Eriocaulaceae | *Eriocaulon parvum* Körn. 검은개수염 |  |  | FACW | 0 | 0 | 1 | 0 | 0 | 0 |
| Eriocaulaceae | *Eriocaulon taquetii* Lecomte 큰개수염 |  | Ⅳ | OBW | 2 | 0 | 0 | 0 | 2 | 0 |
| Poaceae | *Achnatherum pekinense* (Hance) Ohwi 나래새 |  |  | OBU | 4 | 0 | 0 | 0 | 1 | 0 |
| Poaceae | *Agrostis canina* L. 검은겨이삭 |  |  | OBW | 0 | 0 | 0 | 0 | 3 | 0 |
| Poaceae | *Agrostis clavata* Trin. 산겨이삭 |  |  | OBU | 12 | 2 | 4 | 2 | 3 | 1 |
| Poaceae | *Agrostis clavata* Trin. var. *nukabo* Ohwi 겨이삭 |  |  | OBU | 5 | 0 | 2 | 0 | 0 | 2 |
| Poaceae | *Agrostis gigantea* Roth 흰겨이삭 |  |  | OBU | 7 | 3 | 4 | 2 | 0 | 0 |
| Poaceae | *Agrostis scabra* Willd. 긴겨이삭 |  | Ⅲ | OBU | 2 | 0 | 1 | 0 | 0 | 0 |
| Poaceae | *Alopecurus aequalis* Sobol. 뚝새풀 |  |  | FAC | 8 | 0 | 2 | 1 | 1 | 1 |
| Poaceae | *Arthraxon hispidus* (Thunb.) Makino 조개풀 |  |  | OBU | 17 | 4 | 22 | 12 | 15 | 0 |
| Poaceae | *Arundinaria munsuensis* Y.N.Lee 문수조릿대 |  | Ⅴ | OBU | 0 | 0 | 0 | 0 | 1 | 0 |
| Poaceae | *Arundinella hirta* (Thunb.) Tanaka 털새 |  |  | OBW | 8 | 1 | 7 | 3 | 9 | 1 |
| Poaceae | *Arundinella hirta* (Thunb.) Tanaka var. *ciliata* (Thunb.) Koidz. 새 |  |  | FAC | 7 | 0 | 8 | 0 | 18 | 2 |
| Poaceae | *Arundo donax* L. 물대 |  |  | OBU | 1 | 0 | 0 | 0 | 0 | 0 |
| Poaceae | *Avena fatua* L. 메귀리 |  |  | FACU | 0 | 0 | 0 | 0 | 1 | 0 |
| Poaceae | *Beckmannia syzigachne* (Steud.) Fernald 개피 | ○ |  | OBU | 5 | 0 | 2 | 3 | 0 | 0 |
| Poaceae | *Bothriochloa ischaemum* (L.) Keng 바랭이새 |  |  | FACW | 6 | 1 | 11 | 3 | 7 | 0 |
| Poaceae | *Brachypodium sylvaticum* (Huds.) P. Beauv. 숲개밀 |  |  | OBU | 1 | 0 | 3 | 0 | 1 | 0 |
| Poaceae | *Briza minor* L. 방울새풀 |  |  | FACW | 0 | 0 | 4 | 0 | 0 | 0 |
| Poaceae | *Bromus japonicus* Thunb. 참새귀리 |  |  | OBU | 1 | 1 | 0 | 1 | 0 | 0 |
| Poaceae | *Bromus remotiflorus* (Steud.) Ohwi 꼬리새 |  |  | FACW | 0 | 2 | 5 | 0 | 0 | 0 |
| Poaceae | *Bromus tectorum* L. 털빕새귀리 |  |  | OBU | 0 | 0 | 1 | 1 | 0 | 0 |
| Poaceae | *Calamagrostis arundinacea* (L.) Roth 실새풀 |  |  | OBU | 30 | 6 | 19 | 12 | 9 | 0 |
| Poaceae | *Calamagrostis epigejos* (L.) Roth 산조풀 |  |  | OBU | 14 | 4 | 29 | 0 | 7 | 0 |
| Poaceae | *Calamagrostis pseudophragmites* (Haller f.) Koeler 갯조풀 |  |  | OBW | 0 | 1 | 0 | 0 | 0 | 0 |
| Poaceae | *Calamagrostis purpurea* (Trin.) Trin. 산새풀 |  |  | FAC | 1 | 0 | 1 | 0 | 0 | 0 |
| Poaceae | *Cleistogenes hackelii* (Honda) Honda 대새풀 |  |  | OBU | 8 | 9 | 9 | 1 | 0 | 0 |
| Poaceae | *Cymbopogon goeringii* (Steud.) A. Camus 개솔새 |  |  | OBU | 2 | 0 | 0 | 1 | 3 | 0 |
| Poaceae | *Dactylis glomerata* L. 오리새 |  |  | FAC | 10 | 0 | 4 | 2 | 3 | 1 |
| Poaceae | *Diarrhena fauriei* (Hack.) Ohwi 광릉용수염 | ○ |  | OBU | 7 | 2 | 0 | 7 | 0 | 0 |
| Poaceae | *Diarrhena japonica* (Franch. & Sav.) Franch. & Sav. 용수염 |  |  | OBU | 2 | 1 | 2 | 2 | 4 | 0 |
| Poaceae | *Diarrhena mandshurica* Maxim. 껍질용수염 | ○ |  | OBW | 7 | 0 | 0 | 0 | 0 | 0 |
| Poaceae | *Digitaria ciliaris* (Retz.) Koeler 바랭이 |  |  | FACU | 1 | 3 | 3 | 2 | 1 | 1 |
| Poaceae | *Digitaria violascens* Link 민바랭이 |  |  | FACU | 0 | 0 | 0 | 1 | 0 | 0 |
| Poaceae | *Dimeria ornithopoda* Trin. 잔디바랭이 |  |  | FACW | 0 | 0 | 1 | 0 | 0 | 3 |
| Poaceae | *Echinochloa crus-galli* (L.) P. Beauv. 돌피 |  |  | OBU | 9 | 1 | 10 | 3 | 3 | 1 |
| Poaceae | *Echinochloa crus-galli* (L.) P. Beauv. var. *echinatum* (Willd.) Honda 물피 |  |  | FACU | 0 | 0 | 2 | 1 | 1 | 0 |
| Poaceae | *Echinochloa esculenta* (A. Braun) H. Scholz 피 |  |  | OBU | 0 | 0 | 0 | 0 | 2 | 0 |
| Poaceae | *Echinochloa oryzicola* (Vasinger) Vasinger 논피 |  |  | FACU | 0 | 1 | 0 | 0 | 0 | 0 |
| Poaceae | *Eleusine indica* (L.) Gaertn. 왕바랭이 |  |  | FACU | 0 | 0 | 2 | 0 | 0 | 0 |
| Poaceae | *Elymus ciliaris* (Trin. ex Bunge) Tzvelev 속털개밀 |  |  | OBW | 3 | 1 | 2 | 0 | 0 | 0 |
| Poaceae | *Elymus gmelinii* (Ledeb.) Tzvelev 털개밀 |  |  | FAC | 0 | 0 | 2 | 0 | 0 | 0 |
| Poaceae | *Elymus repens* (L.) Gould 구주개밀 |  |  | OBU | 0 | 0 | 1 | 0 | 0 | 0 |
| Poaceae | *Elymus tsukushiensis* Honda var. *transiens* (Hack.) K. Osada 개밀 |  |  | OBW | 7 | 2 | 15 | 3 | 1 | 0 |
| Poaceae | *Eragrostis ferruginea* (Thunb.) P. Beauv. 그령 |  |  | OBU | 3 | 2 | 6 | 2 | 4 | 0 |
| Poaceae | *Eriochloa villosa* (Thunb.) Kunth 나도개피 |  |  | FACU | 1 | 0 | 2 | 4 | 1 | 0 |
| Poaceae | *Eulalia speciosa* (Debeaux) Kuntze 개억새 |  |  | FACU | 0 | 0 | 0 | 0 | 1 | 0 |
| Poaceae | *Festuca arundinacea* Schreb. 큰김의털 |  |  | OBU | 2 | 0 | 5 | 4 | 1 | 0 |
| Poaceae | *Festuca extremiorientalis* Ohwi 왕김의털아재비 |  |  | OBU | 0 | 0 | 0 | 0 | 1 | 1 |
| Poaceae | *Festuca ovina* L. 김의털 |  |  | FAC | 1 | 0 | 1 | 1 | 1 | 1 |
| Poaceae | *Festuca ovina* L. var. *koreanoalpina* Ohwi 두메김의털 |  |  | FACU | 1 | 0 | 0 | 0 | 0 | 0 |
| Poaceae | *Festuca parvigluma* Steud. 김의털아재비 |  |  | OBW | 5 | 0 | 0 | 0 | 0 | 1 |
| Poaceae | *Festuca rubra* L. 왕김의털 |  | Ⅳ | OBU | 1 | 0 | 0 | 1 | 0 | 0 |
| Poaceae | *Glyceria ischyroneura* Steud. 진들피 |  |  | OBU | 0 | 0 | 0 | 0 | 1 | 0 |
| Poaceae | *Glyceria leptolepis* Ohwi 왕미꾸리광이 | ○ | Ⅱ | FACW | 37 | 5 | 20 | 9 | 13 | 0 |
| Poaceae | *Hemarthria sibirica* (Gand.) Ohwi 쇠치기풀 |  |  | FACU | 0 | 0 | 6 | 1 | 1 | 0 |
| Poaceae | *Hierochloe odorata* (L.) P. Beauv. 향모 |  |  | OBU | 0 | 0 | 0 | 0 | 0 | 1 |
| Poaceae | *Imperata cylindrica* (L.) Raeusch. 띠 |  |  | OBU | 0 | 0 | 6 | 0 | 3 | 1 |
| Poaceae | *Isachne globosa* (Thunb.) Kuntze 기장대풀 |  |  | OBU | 31 | 6 | 30 | 16 | 30 | 11 |
| Poaceae | Ischaemum anthephoroides (Steud.) Miq. 갯쇠보리 |  | Ⅰ | FACW | 0 | 0 | 0 | 0 | 4 | 0 |
| Poaceae | *Ischaemum aristatum* L. var. *glaucum* (Honda) T. Koyama 쇠보리 |  |  | FACU | 1 | 1 | 8 | 0 | 6 | 0 |
| Poaceae | *Koeleria macrantha* (Ledeb.) Schult. 도랭이피 |  |  | OBW | 0 | 0 | 0 | 1 | 0 | 0 |
| Poaceae | *Leersia japonica* (Makino ex Honda) Honda 나도겨풀 |  |  | OBW | 11 | 2 | 12 | 7 | 8 | 0 |
| Poaceae | *Leersia oryzoides* (L.) Sw. 좀겨풀 |  |  | FACW | 0 | 0 | 1 | 1 | 0 | 0 |
| Poaceae | *Lolium multiflorum* Lam. 쥐보리 |  |  | FACU | 0 | 0 | 0 | 0 | 0 | 1 |
| Poaceae | *Lolium perenne* L. 호밀풀 |  |  | OBU | 0 | 0 | 1 | 0 | 0 | 0 |
| Poaceae | *Lophatherum gracile* Brongn. 조릿대풀 |  | Ⅲ | OBU | 0 | 0 | 0 | 0 | 2 | 0 |
| Poaceae | *Melica nutans* L. 왕쌀새 | ○ | Ⅱ | OBU | 1 | 0 | 0 | 0 | 0 | 0 |
| Poaceae | *Melica onoei* Franch. & Sav. 쌀새 |  |  | OBU | 6 | 1 | 9 | 1 | 5 | 0 |
| Poaceae | *Microstegium japonicum* (Miq.) Koidz. 민바랭이새 |  |  | OBU | 4 | 0 | 0 | 0 | 0 | 0 |
| Poaceae | *Microstegium vimineum* (Trin.) A. Camus 나도바랭이새 |  |  | OBU | 46 | 24 | 53 | 17 | 12 | 0 |
| Poaceae | *Microstegium vimineum* (Trin.) A. Camus var. *polystachyum* (Franch. & Sav.) Ohwi 큰듬성이삭새 |  |  | FACU | 0 | 1 | 0 | 3 | 2 | 0 |
| Poaceae | *Milium effusum* L. 나도겨이삭 |  |  | OBW | 3 | 1 | 0 | 0 | 0 | 0 |
| Poaceae | *Miscanthus sacchariflorus* (Maxim.) Benth. & Hool. f. ex Franch. 물억새 | ○ |  | FACU | 31 | 5 | 55 | 11 | 34 | 0 |
| Poaceae | *Miscanthus sinensis* Andersson 참억새 |  |  | FACW | 18 | 4 | 13 | 11 | 19 | 2 |
| Poaceae | *Miscanthus sinensis* Andersson var. *purpurascens* (Andersson) Matsum. 억새 |  |  | OBU | 16 | 5 | 16 | 3 | 13 | 3 |
| Poaceae | *Molinia japonica* Hack. 진퍼리새 |  |  | OBW | 44 | 12 | 24 | 2 | 31 | 0 |
| Poaceae | *Muhlenbergia huegelii* Trin. 큰쥐꼬리새 |  |  | OBU | 0 | 1 | 1 | 3 | 0 | 0 |
| Poaceae | *Muhlenbergia japonica* Steud. 쥐꼬리새 |  |  | FACU | 10 | 2 | 8 | 2 | 1 | 0 |
| Poaceae | *Oplismenus burmanni* (Retz.) P. Beauv. 민주름조개풀 |  |  | OBU | 0 | 0 | 0 | 2 | 2 | 0 |
| Poaceae | *Oplismenus undulatifolius* (Ard.) P. Beauv. 주름조개풀 |  |  | FACU | 59 | 33 | 72 | 39 | 65 | 1 |
| Poaceae | *Panicum bisulcatum* Thunb. 개기장 |  |  | OBU | 4 | 4 | 7 | 4 | 7 | 1 |
| Poaceae | *Panicum dichotomiflorum* Michx. 미국개기장 |  |  | FAC | 3 | 0 | 3 | 3 | 0 | 0 |
| Poaceae | *Paspalum distichum* L. var. *indutum* Shinners 털물참새피 |  |  | OBW | 0 | 1 | 2 | 0 | 0 | 0 |
| Poaceae | *Paspalum thunbergii* Kunth ex Steud. 참새피 |  |  | OBU | 1 | 0 | 7 | 1 | 8 | 2 |
| Poaceae | *Pennisetum alopecuroides* (L.) Spreng. 수크령 |  |  | OBU | 5 | 1 | 14 | 5 | 9 | 1 |
| Poaceae | *Phacelurus latifolius* (Steud.) Ohwi 모새달 |  | Ⅰ | OBU | 0 | 0 | 0 | 0 | 1 | 0 |
| Poaceae | *Phalaris arundinacea* L. 갈풀 |  |  | OBU | 7 | 6 | 13 | 3 | 10 | 0 |
| Poaceae | *Phleum pratense* L. 큰조아재비 |  |  | OBU | 4 | 0 | 1 | 1 | 0 | 1 |
| Poaceae | *Phragmites australis* (Cav.) Trin. ex Steud. 갈대 |  |  | OBU | 36 | 6 | 39 | 6 | 15 | 2 |
| Poaceae | *Phragmites japonicus* Steud. 달뿌리풀 |  |  | OBW | 60 | 23 | 36 | 21 | 22 | 0 |
| Poaceae | *Phyllostachys nigra* (Lodd. ex Lindl.) Munro var. *henonis* (Mitford) Stapf ex Rendle 솜대 |  |  | OBU | 0 | 0 | 1 | 0 | 1 | 0 |
| Poaceae | *Phyllostachys reticulata* (Rupr.) K. Koch. 왕대 |  |  | OBU | 0 | 0 | 0 | 0 | 1 | 0 |
| Poaceae | *Poa annua* L. 새포아풀 |  |  | OBU | 0 | 0 | 0 | 1 | 0 | 0 |
| Poaceae | *Poa pratensis* L. 왕포아풀 |  |  | OBU | 3 | 0 | 4 | 1 | 0 | 0 |
| Poaceae | *Poa sphondylodes* Trin. 포아풀 |  |  | FAC | 1 | 0 | 2 | 0 | 1 | 1 |
| Poaceae | *Poa viridula* Palib. 청포아풀 |  |  | OBU | 1 | 1 | 0 | 0 | 0 | 0 |
| Poaceae | *Pseudosasa japonica* (Siebold & Zucc. ex Steud.) Makino ex Nakai 이대 |  |  | OBW | 0 | 0 | 3 | 0 | 1 | 0 |
| Poaceae | *Sacciolepis indica* (L.) Chase 좀물뚝새 |  |  | OBU | 5 | 1 | 2 | 2 | 4 | 0 |
| Poaceae | *Sasa borealis* (Hack.) Makino & Shibata 조릿대 |  |  | FACU | 19 | 0 | 2 | 0 | 10 | 1 |
| Poaceae | *Sasa quelpaertensis* Nakai 제주조릿대 |  |  | FACW | 0 | 0 | 0 | 0 | 0 | 3 |
| Poaceae | *Schizachne purpurascens* (Torr.) Swallen subsp. *callosa* (Turcz. ex Griseb.) T. Koyama & Kawano 호오리새 | ○ | Ⅱ | FACU | 0 | 0 | 1 | 0 | 0 | 0 |
| Poaceae | *Schizachyrium brevifolium* (Sw.) Nees ex Büse 쇠풀 |  |  | FACU | 0 | 0 | 0 | 1 | 0 | 0 |
| Poaceae | *Setaria chondrachne* (Steud.) Honda 조아재비 |  | Ⅰ | OBU | 0 | 0 | 2 | 0 | 0 | 0 |
| Poaceae | *Setaria faberi* R. A. W. Herrm. 가을강아지풀 |  |  | FACW | 3 | 1 | 0 | 1 | 1 | 0 |
| Poaceae | *Setaria pumila* (Poir.) Roem. & Schult. 금강아지풀 |  |  | OBW | 4 | 3 | 10 | 2 | 2 | 1 |
| Poaceae | *Setaria viridis* (L.) P. Beauv. 강아지풀 |  |  | FACU | 6 | 7 | 6 | 7 | 3 | 1 |
| Poaceae | *Setaria viridis* (L.) P. Beauv. subsp. *pycnocoma* (Steud.) Tzvelev 수강아지풀 |  |  | FACU | 1 | 1 | 1 | 0 | 0 | 0 |
| Poaceae | *Spodiopogon cotulifer* (Thunb.) Hack. 기름새 |  |  | OBU | 12 | 4 | 10 | 5 | 4 | 0 |
| Poaceae | *Spodiopogon sibiricus* Trin. 큰기름새 | ○ |  | OBU | 28 | 10 | 24 | 11 | 17 | 0 |
| Poaceae | *Sporobolus fertilis* (Steud.) Clayton 쥐꼬리새풀 |  |  | OBU | 0 | 0 | 1 | 1 | 2 | 0 |
| Poaceae | *Sporobolus piliferus* (Trin.) Kunth 나도잔디 |  |  | OBU | 0 | 0 | 0 | 1 | 0 | 0 |
| Poaceae | *Themeda triandra* Forssk. 솔새 |  |  | OBU | 0 | 0 | 0 | 1 | 0 | 0 |
| Poaceae | *Trisetum bifidum* (Thunb.) Ohwi 잠자리피 |  |  | OBU | 7 | 2 | 1 | 1 | 2 | 1 |
| Poaceae | *Trisetum sibiricum* Rupr. 시베리아잠자리피 | ○ | Ⅱ | FAC | 1 | 1 | 0 | 0 | 0 | 0 |
| Poaceae | *Vulpia myuros* (L.) C. C. Gmel. 들묵새 |  |  | OBU | 0 | 0 | 0 | 1 | 0 | 0 |
| Poaceae | *Zizania latifolia* (Griseb.) Turcz. ex Stapf 줄 |  |  | OBU | 3 | 4 | 2 | 1 | 0 | 0 |
| Poaceae | *Zoysia japonica* Steud. 잔디 |  |  | FACW | 0 | 1 | 3 | 2 | 2 | 2 |
| Poaceae | *Zoysia sinica* Hance 갯잔디 |  |  | FACW | 0 | 0 | 0 | 0 | 1 | 0 |
| Araceae | *Acorus calamus* L. 창포 |  | Ⅱ | FACW | 6 | 0 | 2 | 0 | 3 | 1 |
| Araceae | *Acorus gramineus* Aiton 석창포 |  | Ⅲ | FACU | 1 | 0 | 0 | 0 | 0 | 0 |
| Araceae | *Arisaema amurense* Maxim. 둥근잎천남성 |  |  | FAC | 14 | 0 | 0 | 3 | 1 | 0 |
| Araceae | *Arisaema amurense* Maxim. f. *serratum* (Nakai) Kitag. 천남성 |  |  | OBU | 13 | 4 | 4 | 5 | 2 | 1 |
| Araceae | *Arisaema heterophyllum* Blume 두루미천남성 |  | Ⅰ | OBU | 7 | 4 | 0 | 0 | 0 | 0 |
| Araceae | *Arisaema ringens* (Thunb.) Schott 큰천남성 |  | Ⅰ | FACW | 0 | 0 | 1 | 0 | 1 | 0 |
| Araceae | *Arisaema serratum* (Thunb.) Schott 점박이천남성 |  |  | OBU | 20 | 2 | 0 | 0 | 0 | 0 |
| Araceae | *Pinellia ternata* (Thunb.) Makino 반하 |  |  | OBU | 3 | 2 | 3 | 1 | 1 | 0 |
| Araceae | *Symplocarpus nipponicus* Makino 애기앉은부채 |  | Ⅲ | OBU | 4 | 0 | 0 | 0 | 0 | 0 |
| Lemnaceae | *Lemna perpusilla* Torr. 좀개구리밥 |  |  | OBW | 3 | 0 | 4 | 0 | 0 | 0 |
| Lemnaceae | *Spirodela polyrrhiza* (L.) Schleid. 개구리밥 |  |  | OBW | 3 | 0 | 4 | 0 | 0 | 2 |
| Lemnaceae | *Wolffia arrhiza* (L.) Horkel ex Wimm. 분개구리밥 |  |  | OBW | 0 | 0 | 0 | 0 | 0 | 2 |
| Typhaceae | *Sparganium japonicum* Rothert 긴흑삼릉 | ○ | Ⅳ | FAC | 1 | 0 | 0 | 0 | 0 | 0 |
| Typhaceae | *Sparganium stoloniferum* (Graebn.) Buch.-Ham. ex Juz. 흑삼릉 |  | Ⅲ | FAC | 1 | 0 | 0 | 0 | 1 | 0 |
| Typhaceae | *Typha angustifolia* L. 애기부들 |  |  | OBW | 3 | 2 | 10 | 0 | 2 | 1 |
| Typhaceae | *Typha latifolia* L. 큰잎부들 |  |  | FACU | 6 | 1 | 0 | 0 | 0 | 0 |
| Typhaceae | *Typha orientalis* C. Presl 부들 |  |  | OBW | 14 | 0 | 5 | 1 | 4 | 2 |
| Cyperaceae | *Bolboschoenus fluviatilis* (Torr.) Soják 큰매자기 |  |  | FACW | 1 | 0 | 0 | 0 | 0 | 0 |
| Cyperaceae | *Bolboschoenus maritimus* (L.) Palla 매자기 |  | Ⅱ | FACU | 1 | 0 | 0 | 0 | 0 | 0 |
| Cyperaceae | *Bulbostylis densa* (Wall.) Hand.-Mazz. 꽃하늘지기 |  |  | FACU | 0 | 0 | 0 | 0 | 0 | 1 |
| Cyperaceae | *Carex aphanolepis* Franch. & Sav. 골사초 |  |  | FAC | 2 | 2 | 3 | 1 | 0 | 0 |
| Cyperaceae | *Carex appendiculata* (Trautv. & C. A. Mey.) Kük. 뚝사초 | ○ | Ⅳ | OBW | 4 | 0 | 0 | 0 | 1 | 0 |
| Cyperaceae | *Carex arenicola* F. Schmidt 진퍼리사초 |  | Ⅳ | OBU | 1 | 0 | 0 | 0 | 0 | 0 |
| Cyperaceae | *Carex augustinowiczii* Meinsh. ex Korsh. 북사초 | ○ | Ⅱ | FACW | 3 | 0 | 1 | 0 | 0 | 0 |
| Cyperaceae | *Carex biwensis* Franch. 솔잎사초 |  |  | OBU | 1 | 0 | 0 | 0 | 1 | 1 |
| Cyperaceae | *Carex boottiana* Hook. & Arn. 밀사초 |  |  | OBW | 0 | 0 | 0 | 0 | 1 | 0 |
| Cyperaceae | *Carex bostrychostigma* Maxim. 길뚝사초 |  |  | OBU | 7 | 3 | 1 | 2 | 4 | 0 |
| Cyperaceae | *Carex breviculmis* R.Br. 청사초 |  |  | OBU | 3 | 1 | 1 | 0 | 0 | 1 |
| Cyperaceae | *Carex brownii* Tuck. 흰꼬리사초 |  | Ⅰ | OBU | 1 | 0 | 0 | 1 | 0 | 0 |
| Cyperaceae | *Carex canescens* L. 산사초 |  | Ⅳ | OBU | 1 | 0 | 0 | 0 | 0 | 0 |
| Cyperaceae | *Carex capillacea* Boott 잔솔잎사초 |  |  | OBU | 0 | 0 | 0 | 0 | 1 | 0 |
| Cyperaceae | *Carex capricornis* Meinsh. ex Maxim. 양뿔사초 | ○ | Ⅳ | OBU | 0 | 0 | 0 | 1 | 0 | 0 |
| Cyperaceae | *Carex ciliato-marginata* Nakai 털대사초 |  |  | OBU | 0 | 1 | 1 | 0 | 6 | 0 |
| Cyperaceae | *Carex cinerascens* Kük. 회색사초 |  | Ⅱ | OBU | 1 | 1 | 2 | 1 | 0 | 0 |
| Cyperaceae | *Carex dickinsii* Franch. & Sav. 도깨비사초 |  |  | OBU | 36 | 11 | 29 | 1 | 12 | 1 |
| Cyperaceae | *Carex dimorpholepis* Steud. 이삭사초 |  |  | OBU | 5 | 13 | 7 | 4 | 6 | 13 |
| Cyperaceae | *Carex dispalata* Boott 삿갓사초 |  | Ⅰ | OBU | 42 | 4 | 23 | 8 | 24 | 0 |
| Cyperaceae | *Carex doniana* Spreng. 흰사초 |  | Ⅰ | OBU | 0 | 0 | 1 | 0 | 1 | 0 |
| Cyperaceae | *Carex erythrobasis* H. Lév. & Vaniot 한라사초 | ○ | Ⅱ | FACW | 5 | 4 | 2 | 4 | 1 | 0 |
| Cyperaceae | *Carex fernaldiana* H. Lév. & Vaniot 실사초 |  |  | OBU | 2 | 0 | 0 | 0 | 0 | 0 |
| Cyperaceae | *Carex forficula* Franch. & Sav. 산뚝사초 |  |  | FACW | 61 | 12 | 30 | 7 | 21 | 0 |
| Cyperaceae | *Carex gibba* Wahlenb. 나도별사초 |  |  | OBU | 0 | 0 | 0 | 1 | 1 | 0 |
| Cyperaceae | *Carex gifuensis* Franch. 애기감둥사초 |  |  | FACW | 0 | 0 | 2 | 0 | 2 | 0 |
| Cyperaceae | *Carex glabrescens* (Kük.) Ohwi 곱슬사초 | ○ |  | FACW | 5 | 2 | 1 | 0 | 1 | 0 |
| Cyperaceae | *Carex hakonensis* Franch. & Sav. 애기바늘사초 |  |  | FACU | 3 | 0 | 0 | 0 | 0 | 0 |
| Cyperaceae | *Carex heterolepis* Bunge 산비늘사초 | ○ |  | OBU | 0 | 0 | 0 | 1 | 0 | 0 |
| Cyperaceae | *Carex humilis* Leyss. var. *nana* (H. Lév. & Vaniot) Ohwi 가는잎그늘사초 |  |  | OBU | 15 | 6 | 10 | 11 | 11 | 0 |
| Cyperaceae | *Carex idzuroei* Franch. & Sav. 좀도깨비사초 |  |  | FAC | 0 | 1 | 0 | 1 | 0 | 1 |
| Cyperaceae | *Carex jaluensis* Kom. 참삿갓사초 | ○ | Ⅱ | OBU | 7 | 2 | 8 | 1 | 0 | 0 |
| Cyperaceae | *Carex japonica* Thunb. 개찌버리사초 |  |  | FACW | 20 | 10 | 15 | 6 | 0 | 1 |
| Cyperaceae | *Carex laevissima* Nakai 애괭이사초 | ○ |  | FACW | 3 | 1 | 0 | 1 | 0 | 0 |
| Cyperaceae | *Carex lanceolata* Boott 그늘사초 |  |  | FACU | 19 | 6 | 10 | 9 | 12 | 0 |
| Cyperaceae | *Carex lasiocarpa* Ehrh. 벌사초 |  | Ⅳ | OBW | 1 | 0 | 0 | 0 | 0 | 0 |
| Cyperaceae | *Carex lasiolepis* Franch. 난사초 |  | Ⅲ | OBU | 1 | 0 | 0 | 0 | 0 | 0 |
| Cyperaceae | *Carex leiorhyncha* C. A. Mey.산괭이사초 | ○ |  | FACU | 2 | 0 | 1 | 1 | 0 | 0 |
| Cyperaceae | *Carex lenta* D. Don 줄사초 |  | Ⅰ | FAC | 1 | 0 | 0 | 0 | 0 | 0 |
| Cyperaceae | *Carex maculata* Boott 무늬사초 |  | Ⅰ | OBU | 0 | 0 | 0 | 0 | 0 | 1 |
| Cyperaceae | *Carex maximowiczii* Miq. 왕비늘사초 |  |  | OBU | 33 | 2 | 3 | 1 | 9 | 3 |
| Cyperaceae | *Carex mitrata* Franch. var. *aristata* Ohwi 까락겨사초 |  | Ⅰ | FAC | 0 | 0 | 0 | 0 | 0 | 1 |
| Cyperaceae | *Carex miyabei* Franch. 융단사초 |  |  | OBU | 17 | 3 | 10 | 6 | 5 | 0 |
| Cyperaceae | *Carex mollicula* Boott 애기흰사초 |  |  | FAC | 1 | 0 | 1 | 2 | 8 | 1 |
| Cyperaceae | *Carex nervata* Franch. & Sav. 양지사초 |  |  | OBW | 0 | 0 | 1 | 0 | 0 | 1 |
| Cyperaceae | *Carex neurocarpa* Maxim. 괭이사초 | ○ |  | FAC | 7 | 0 | 4 | 1 | 1 | 0 |
| Cyperaceae | *Carex okamotoi* Ohwi 지리대사초 |  | Ⅰ | OBU | 0 | 0 | 2 | 0 | 0 | 0 |
| Cyperaceae | *Carex onoei* Franch. & Sav. 바늘사초 | ○ | Ⅱ | OBU | 12 | 0 | 1 | 2 | 0 | 0 |
| Cyperaceae | *Carex phacota* Spreng. 비늘사초 |  |  | OBU | 1 | 0 | 4 | 1 | 0 | 1 |
| Cyperaceae | *Carex pilosa* Scop. 털사초 |  | Ⅲ | FACU | 4 | 0 | 0 | 0 | 0 | 0 |
| Cyperaceae | *Carex planiculmis* Kom. 그늘흰사초 | ○ | Ⅱ | FACU | 15 | 2 | 3 | 3 | 0 | 1 |
| Cyperaceae | *Carex polyschoena* H. Lév. & Vaniot 가지청사초 |  |  | OBU | 17 | 4 | 0 | 0 | 0 | 0 |
| Cyperaceae | *Carex pseudochinensis* H. Lév. & Vaniot 햇사초 |  |  | OBU | 0 | 0 | 0 | 0 | 1 | 0 |
| Cyperaceae | *Carex pumila* Thunb. 좀보리사초 |  |  | OBU | 1 | 0 | 0 | 0 | 0 | 0 |
| Cyperaceae | *Carex quadriflora* (Kük.) Ohwi 녹빛사초 |  | Ⅱ | OBW | 1 | 0 | 0 | 0 | 0 | 0 |
| Cyperaceae | *Carex remotiuscula* Wahlenb. 층실사초 |  | Ⅳ | OBU | 1 | 0 | 0 | 0 | 1 | 0 |
| Cyperaceae | *Carex rhynchophysa* Fisch., C. A. Mey.& Avé-Lall. 왕삿갓사초 | ○ | Ⅳ | OBW | 1 | 0 | 0 | 0 | 0 | 0 |
| Cyperaceae | *Carex sabynensis* Less. ex Kunth 실청사초 |  |  | OBU | 4 | 1 | 3 | 4 | 9 | 0 |
| Cyperaceae | *Carex sabynensis* Less. ex Kunth var. *leiosperma* Ohwi 지리실청사초 |  |  | OBW | 0 | 2 | 0 | 0 | 0 | 0 |
| Cyperaceae | *Carex scabrifolia* Steud. 천일사초 |  |  | OBU | 0 | 0 | 0 | 0 | 1 | 0 |
| Cyperaceae | *Carex schmidtii* Meinsh. 참뚝사초 | ○ |  | OBW | 6 | 0 | 3 | 0 | 1 | 0 |
| Cyperaceae | *Carex siderosticta* Hance 대사초 |  |  | OBU | 39 | 5 | 12 | 4 | 20 | 1 |
| Cyperaceae | *Carex stipata* Muhl. ex Willd. 양덕사초 |  | Ⅳ | FACU | 3 | 0 | 0 | 0 | 0 | 0 |
| Cyperaceae | *Carex suifunensis* Kom. 가는비늘사초 |  |  | OBU | 4 | 1 | 2 | 1 | 0 | 0 |
| Cyperaceae | *Carex tegulata* H. Lév. & Vaniot 구슬사초 |  | Ⅲ | FACW | 0 | 0 | 1 | 0 | 0 | 0 |
| Cyperaceae | *Carex tenuiflora* Wahlenb. 별사초 | ○ | Ⅳ | OBW | 2 | 0 | 0 | 0 | 0 | 0 |
| Cyperaceae | *Carex transversa* Boott 화살사초 |  |  | FAC | 0 | 0 | 0 | 0 | 0 | 1 |
| Cyperaceae | *Cladium chinense* Nees 층층고랭이 |  | Ⅳ | FACU | 0 | 0 | 0 | 0 | 1 | 0 |
| Cyperaceae | *Cyperus amuricus* Maxim. 방동사니 |  |  | FACW | 3 | 1 | 3 | 5 | 3 | 0 |
| Cyperaceae | *Cyperus difformis* L. 알방동사니 |  |  | FAC | 0 | 0 | 1 | 0 | 0 | 0 |
| Cyperaceae | *Cyperus exaltatus* Retz. var. *iwasakii* (Makino) T. Koyama 왕골 |  |  | FACU | 1 | 0 | 0 | 0 | 0 | 0 |
| Cyperaceae | *Cyperus hakonensis* Franch. & Sav. 병아리방동사니 |  |  | FACW | 0 | 0 | 0 | 0 | 1 | 0 |
| Cyperaceae | *Cyperus iria* L. 참방동사니 |  |  | OBU | 0 | 0 | 0 | 1 | 0 | 0 |
| Cyperaceae | *Cyperus microiria* Steud. 금방동사니 |  |  | FAC | 5 | 2 | 3 | 0 | 0 | 0 |
| Cyperaceae | *Cyperus nipponicus* Franch. & Sav. 푸른방동사니 |  |  | FACU | 1 | 0 | 0 | 0 | 0 | 0 |
| Cyperaceae | *Cyperus orthostachyus* Franch. & Sav. 쇠방동사니 |  |  | FAC | 3 | 0 | 1 | 1 | 0 | 0 |
| Cyperaceae | *Cyperus tenuispica* Steud. 우산방동사니 |  |  | OBU | 0 | 0 | 0 | 0 | 2 | 0 |
| Cyperaceae | *Eleocharis acicularis* (L.) Roem. & Schult. var. *longiseta* Svenson 쇠털골 |  |  | FAC | 1 | 0 | 0 | 0 | 2 | 2 |
| Cyperaceae | *Eleocharis attenuata* (Franch. & Sav.) Palla f. *laeviseta* (Nakai) H. Hara 참바늘골 |  |  | FAC | 5 | 0 | 1 | 0 | 1 | 0 |
| Cyperaceae | *Eleocharis congesta* D. Don 바늘골 |  |  | FACW | 4 | 0 | 3 | 0 | 1 | 2 |
| Cyperaceae | *Eleocharis dulcis* (Burm.f.) Trin. ex Hensch. 남방개 |  | Ⅳ | OBU | 1 | 0 | 0 | 0 | 0 | 1 |
| Cyperaceae | *Eleocharis equisetiformis* (Meinsh.) B. Fedtsch. 까락골 |  |  | FACU | 0 | 3 | 0 | 0 | 1 | 0 |
| Cyperaceae | *Eleocharis kamtschatica* (C. A. Mey.) Kom. 올방개아재비 |  |  | FACW | 0 | 0 | 0 | 1 | 5 | 0 |
| Cyperaceae | *Eleocharis kuroguwai* Ohwi 올방개 |  |  | OBW | 6 | 0 | 5 | 0 | 0 | 3 |
| Cyperaceae | *Eleocharis mamillata* L.f. var. *cyclocarpa* Kitag. 물꼬챙이골 | ○ |  | OBW | 9 | 1 | 15 | 0 | 5 | 7 |
| Cyperaceae | *Eleocharis tetraquetra* Nees 네모골 |  | Ⅱ | OBU | 1 | 0 | 0 | 0 | 2 | 2 |
| Cyperaceae | *Eleocharis wichurae* Boeckeler 좀네모골 | ○ |  | FACU | 0 | 0 | 2 | 0 | 0 | 1 |
| Cyperaceae | *Eriophorum gracile* Koch 작은황새풀 | ○ | Ⅴ | OBU | 1 | 0 | 1 | 0 | 0 | 0 |
| Cyperaceae | *Fimbristylis dichotoma* (L.) Vahl 하늘지기 |  |  | OBU | 0 | 1 | 2 | 0 | 0 | 0 |
| Cyperaceae | *Fimbristylis littoralis* Gaudich. 바람하늘지기 |  |  | FACU | 0 | 0 | 1 | 0 | 0 | 3 |
| Cyperaceae | *Fimbristylis squarrosa* Vahl 민하늘지기 |  |  | OBU | 0 | 0 | 0 | 1 | 0 | 0 |
| Cyperaceae | *Fimbristylis tristachya* R.Br. var. *subbispicata* (Nees & Meyen) T. Koyama 꼴하늘지기 |  |  | FACU | 0 | 0 | 0 | 0 | 2 | 1 |
| Cyperaceae | *Kyllinga brevifolia* Rottb. var. *leiolepis* (Franch. & Sav.) H. Hara 파대가리 |  |  | FACW | 7 | 0 | 10 | 1 | 2 | 0 |
| Cyperaceae | *Pycreus sanguinolentus* (Vahl) Nees 방동사니대가리 |  |  | OBU | 3 | 0 | 0 | 1 | 1 | 0 |
| Cyperaceae | *Rhynchospora alba* (L.) Vahl 흰고양이수염 |  |  | OBW | 0 | 0 | 0 | 1 | 0 | 0 |
| Cyperaceae | *Rhynchospora chinensis* Nees & Meyen ex Nees 고양이수염 |  |  | OBW | 0 | 1 | 1 | 0 | 11 | 1 |
| Cyperaceae | *Rhynchospora faberi* C. B. Clarke 골풀아재비 |  |  | OBW | 0 | 0 | 0 | 0 | 9 | 1 |
| Cyperaceae | *Rhynchospora fauriei* Franch. 큰고양이수염 |  |  | OBU | 14 | 0 | 3 | 3 | 2 | 0 |
| Cyperaceae | *Rhynchospora fujiana* Makino 좀고양이수염 |  |  | FAC | 0 | 0 | 0 | 1 | 6 | 1 |
| Cyperaceae | *Schoenoplectiella hotarui* (Ohwi) J. Jung & H. K. Choi 좀올챙이골 |  |  | OBU | 3 | 2 | 2 | 1 | 1 | 0 |
| Cyperaceae | *Schoenoplectiella triangulata* (Roxb.) J. D. Jung & H. K. Choi 송이고랭이 |  |  | OBU | 10 | 1 | 3 | 2 | 2 | 15 |
| Cyperaceae | *Schoenoplectiella wallichii* (Nees) Lye 수원고랭이 |  |  | OBU | 0 | 0 | 6 | 0 | 3 | 0 |
| Cyperaceae | *Schoenoplectus juncoides* (Roxb.) Palla 올챙이고랭이 |  |  | OBU | 11 | 0 | 3 | 1 | 7 | 3 |
| Cyperaceae | *Schoenoplectus nipponicus* (Makino) Soják 물고랭이 |  | Ⅱ | OBW | 0 | 0 | 0 | 0 | 1 | 0 |
| Cyperaceae | *Schoenoplectus tabernaemontani* (C. C. Gmel.) Palla  큰고랭이 |  |  | OBU | 7 | 1 | 3 | 0 | 0 | 6 |
| Cyperaceae | *Schoenoplectus triqueter* (L.) Palla 세모고랭이 |  |  | FACU | 7 | 0 | 6 | 1 | 0 | 1 |
| Cyperaceae | *Scirpus karuisawensis* Makino 솔방울고랭이 |  |  | OBU | 34 | 3 | 41 | 2 | 8 | 0 |
| Cyperaceae | *Scirpus mitsukurianus* Makino 솔방울골 |  |  | OBU | 0 | 0 | 1 | 0 | 0 | 0 |
| Cyperaceae | *Scirpus orientalis* Ohwi 검은도루박이 |  | Ⅳ | OBU | 0 | 1 | 0 | 0 | 0 | 0 |
| Cyperaceae | *Scirpus radicans* Schkuhr 도루박이 | ○ |  | OBU | 7 | 0 | 2 | 1 | 0 | 0 |
| Cyperaceae | *Scirpus wichurae* Boeck. 방울고랭이 |  |  | OBU | 13 | 3 | 11 | 5 | 18 | 0 |
| Cyperaceae | *Scleria parvula* Steud. 너도고랭이 |  |  | OBW | 0 | 0 | 0 | 0 | 5 | 0 |
| Cyperaceae | *Scleria rugosa* R.Br. 가시개올미 |  | Ⅳ | OBU | 0 | 0 | 1 | 0 | 3 | 0 |
| Orchidaceae | *Cephalanthera erecta* (Thunb.) Blume 은난초 |  |  | OBU | 3 | 0 | 3 | 0 | 2 | 0 |
| Orchidaceae | *Cephalanthera falcata* (Thunb.) Blume 금난초 |  | Ⅲ | FACU | 0 | 0 | 0 | 0 | 2 | 0 |
| Orchidaceae | *Cephalanthera longibracteata* Blume 은대난초 |  |  | OBU | 9 | 3 | 3 | 2 | 2 | 0 |
| Orchidaceae | *Epipactis thunbergii* A. Gray 닭의난초 |  | Ⅱ | FACU | 2 | 1 | 0 | 0 | 9 | 0 |
| Orchidaceae | *Cephalanthera erecta* (Thunb.) Blume f. *subaphylla* (Miyabe & Kudô) Hiroë 꼬마은난초 |  |  | OBU | 1 | 0 | 1 | 0 | 0 | 0 |
| Orchidaceae | *Galearis cyclochila* (Franch. & Sav.) Soó 나도제비란 |  | Ⅱ | OBU | 5 | 0 | 0 | 0 | 0 | 0 |
| Orchidaceae | *Goodyera schlechtendaliana* Rchb. f. 사철란 |  |  | FACU | 0 | 0 | 1 | 0 | 0 | 0 |
| Orchidaceae | *Habenaria linearifolia* Maxim. 잠자리난초 |  |  | OBU | 0 | 0 | 8 | 0 | 7 | 0 |
| Orchidaceae | *Habenaria radiata* (Thunb.) Spreng. 해오라비난초 |  | Ⅴ | OBW | 0 | 0 | 0 | 0 | 1 | 0 |
| Orchidaceae | *Liparis krameri* Franch. & Sav. 나나벌이난초 |  |  | OBU | 0 | 0 | 2 | 0 | 0 | 1 |
| Orchidaceae | *Liparis kumokiri* F. Maek. 옥잠난초 |  |  | OBW | 40 | 5 | 15 | 9 | 7 | 0 |
| Orchidaceae | *Liparis makinoana* Schltr. 나리난초 |  | Ⅰ | OBU | 2 | 0 | 0 | 0 | 0 | 0 |
| Orchidaceae | *Oreorchis patens* (Lindl.) Lindl. 감자난초 |  |  | FACU | 1 | 0 | 0 | 0 | 0 | 0 |
| Orchidaceae | *Platanthera hologlottis* Maxim. 흰제비란 | ○ | Ⅴ | FACW | 2 | 0 | 2 | 0 | 0 | 1 |
| Orchidaceae | *Platanthera sachalinensis* F. Schmidt 큰제비란 |  | Ⅳ | FACU | 2 | 0 | 0 | 0 | 0 | 0 |
| Orchidaceae | *Platanthera ussuriensis* (Regel & Maack) Maxim.  나도잠자리란 |  | Ⅱ | OBU | 1 | 1 | 0 | 0 | 0 | 0 |
| Orchidaceae | *Pogonia japonica* Rchb. f. 큰방울새란 | ○ | Ⅱ | OBU | 4 | 0 | 1 | 0 | 6 | 0 |
| Orchidaceae | *Pogonia minor* (Makino) Makino 방울새란 |  |  | OBU | 0 | 0 | 0 | 0 | 4 | 0 |
| Orchidaceae | *Spiranthes sinensis* (Pers.) Ames 타래난초 |  |  | FACU | 1 | 0 | 2 | 0 | 1 | 2 |

A: Northern lineage plants, B: Floristic target plants, C: Wetland preference of vascular plants, D: Frequency of Gangwon area, E: Frequency of Gyeonggi area, F: Frequency of Gyeongsang area, G: Frequency of Chungcheong area, H: Frequency of Jeonla area, I: Frequency of Jeju area
